# Synthesis of 2,2-Disubstituted Indolin-3-ones via Enolonium Species

**DOI:** 10.1021/acs.joc.5c00873

**Published:** 2025-07-01

**Authors:** Bat-El Oded, Subrata Maity, Haya Kornweitz, Alex M. Szpilman

**Affiliations:** Department of Chemical Sciences, 42732 Ariel University, 4070000 Ariel, Israel

## Abstract

Herein, we present an efficient and operationally simple one-pot synthesis of a broad range of 2,2-disubstituted indolin-3-ones via the double umpolung reaction of 2-aminophenyl-3-oxopropanoate. The 2-substituent introduced in the reaction may be hydroxy or acetamide. The products can then be functionalized further. The procedure has a broad scope and functional group tolerance. Importantly, density functional theory (DFT) calculations provide novel mechanistic insight into both this and related reactions and reveal that two C-enolonium species are key intermediates of this transformation.

## Graphical abstract

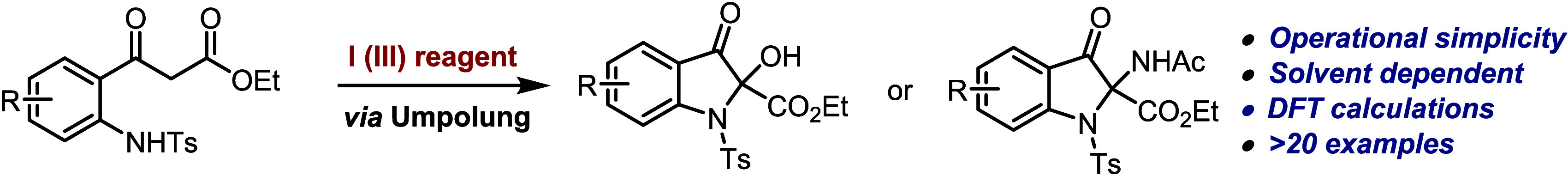

Functionalized oxindoles are an integral part of a wide spectrum of alkaloid natural products^1^ and serve as key building blocks for the synthesis of pharmaceutically relevant compounds.^2^ Particularly, 2,2-disubstituted indolin-3-ones are commonly encountered structural components in bioactive alkaloids. Notably, indolin-3-ones bearing a hydroxy or amine group at the C2-position, such as matemone,^3^ cephalinone B,^4^ secoleuconoxine,^5^ and melochicorin^6^ (Figure 1A), have been found to exhibit a broad range of biological activities.^3,4^

**Figure 1:**
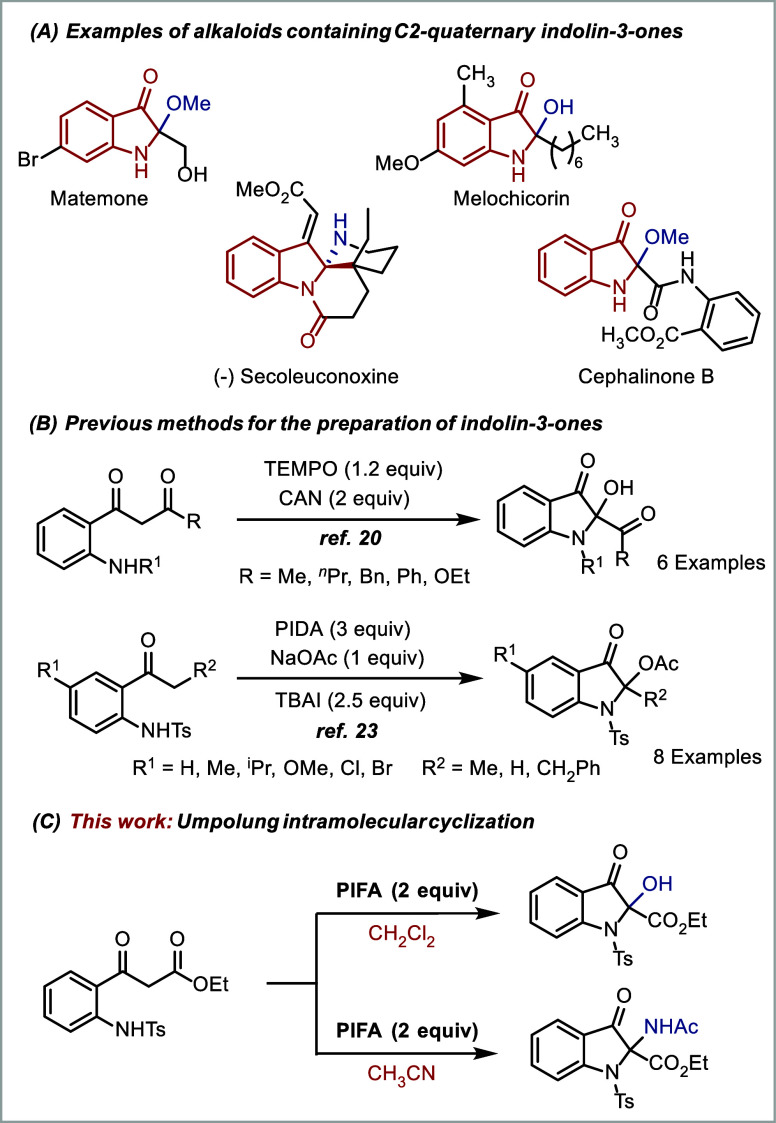
Selected examples of natural products containing indolin-3-ones and methods for their preparation.

Currently, the most used method for synthesizing 2-hydoxy-indolin-3-ones is the oxidation of indoles. This oxidation can be performed using various oxidants including dimethyldioxirane (DMDO),^7^ Pd­(acac)_2_/H_2_O_2_,^8^ and several others.^9–20^ Another reliable strategy involves the intramolecular cyclization of either amino acetophenone derivatives or nitrogen-containing phenyl acetylene derivatives to produce the corresponding 2-hydoxy-indolin-3-ones. Notably, Yang reported on the oxidative cyclization of 2-aminophenyl-1,3-dione using a combination of ceric ammonium nitrate (CAN) and TEMPO as oxidants for the construction of 2-hydroxy-indolinones^21^ (Figure 1B). Recently, Wu and co-workers demonstrated the copper-catalyzed oxidative cyclization of 2-arylethynylanilines for accessing 2-hydroxy indolinones.^22^ Of particular relevance to this work, Fan et al. reported on the cyclization of anilines using PIDA as an oxidant for the construction of indolinones (Figure 1B).^23^ Fan reported 8 examples of which one (R^1^, R^2^ = H) produced in 50% yield was converted into a variety of benzene derivatives via Friedel-Craft-type chemistry with the acetate function as the leaving group. Formation of quaternary centers from other products was not possible. While a mechanism was proposed, no supporting evidence was provided.

Our group has pioneered the preparation of semistable enolonium species from hypervalent iodine reagents and carbonyl enolates and studied their structure spectroscopically and mechanistically. We^24–32^ and other groups^33,34^ have also demonstrated the broad applicability of these in situ generated electrophilic enolonium species in various umpolung intermolecular α-functionalization reactions.^24–32^

We have previously shown that β-keto esters can form enolonium species when treated with hypervalent iodine reagents.^24,29^ We hypothesized that substrates such as 2-aminophenyl-3-oxopropanoate (Figure 1C), where β-keto ester and nucleophilic amino group are tethered in the same molecule, could engage in intramolecular cyclization via enolonium species to produce the indolin-3-one. This compound could then be oxidized in a second step, possibly again via an enolonium species, to afford the 2,2-disubstituted indolin-3-ones.

Thus, we commenced our studies with 2-aminophenyl-3-oxopropanoate **1a** as a model substrate. First, we screened various hypervalent iodine reagents in dichloromethane (DCM) as a solvent at room temperature (Table 1). Naturally at least two equivalents of hypervalent iodine reagent would be required to enable the two oxidation events. Surprisingly, commercially available PIDA did not produce product **2a** (entry 1). To our delight, PIFA produced the anticipated intramolecular cyclized product **2a** in an excellent isolated yield of 97% (entry 2). Commercially available Koser’s reagent and iodosobenzene delivered product **2a** in inferior yields when compared to PIFA (entries 3 and 4). Zhang’s reagent^31^ did not afford the desired product (entry 5). Either increasing or reducing the stoichiometry of PIFA reagent was found to be detrimental and delivered the product in low yields (entries 6 and 7). Using PIFA as an oxidant and screening other solvents such as DCE, THF, or methanol did not yield the product (entries 8–10). Testing other *N*-protecting groups such as *N*-Ac or *N*-Boc indicated that *N*-Ts protection was essential for successful oxidative cyclization.

**Table 1:**
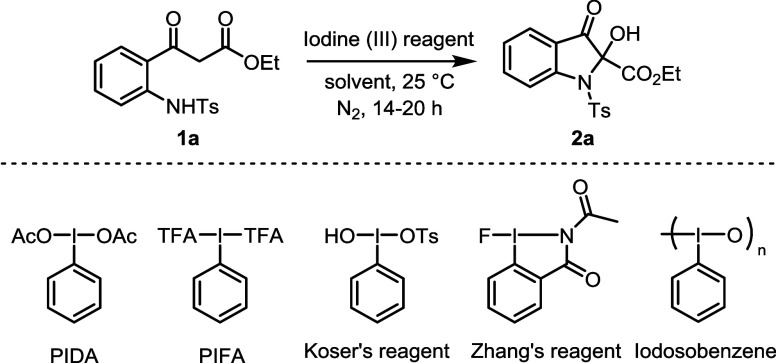
Optimization of Cyclization of 2-Aminophenyl-3-oxopropanoate^a^

entry	iodine(III) reagent (equiv)	solvent	% yield
1	PIDA (2.0)	CH_2_Cl_2_	
2	PIFA (2.0)	CH_2_Cl_2_	97^b^
3	Koser's reagent (2.0)	CH_2_Cl_2_	<10
4	Ph-I = O (2.0)	CH_2_Cl_2_	50
5	Zhang's reagent (2.0)	CH_2_Cl_2_	
6	PIFA (1.3)	CH_2_Cl_2_	59^b^
7	PIFA (2.5)	CH_2_Cl_2_	73^b^
8	Koser's reagent (2.0)	DCE	<30
9	PIFA (2.0)	THF	
10	PIFA (2.0)	MeOH	

a Conditions: 100 mg scale, 0.025 M of DCM as a solvent at 25 °C for 14–20 h under nitrogen atmosphere; yields were determined by ^1^H NMR with 1,3,5-trimethoxybenzene as an internal standard.

b Isolated yields.

With the optimized conditions in hand, the generality of the new protocol was explored, and the results are shown in Scheme 1. A variety of substituted 2-aminophenyl-3-oxopropanoate with a range of electron-deficient or electron-donating groups were successfully converted into the corresponding 2-hydroxy-indolin-3-ones **2a**–**n** in good yields. 2-Aminophenyl-3-oxopropanoate bearing a phenyl ring or a methyl group at the 4*-*position produced the corresponding cyclized product (**2b**, **2c**) in 80 and 62% yields, respectively. The series of halogen (F, Cl, Br, I)-substituted 2-aminophenyl-3-oxopropanoate at the 4*-*position were tolerated under the reaction conditions.

**Scheme 1:**
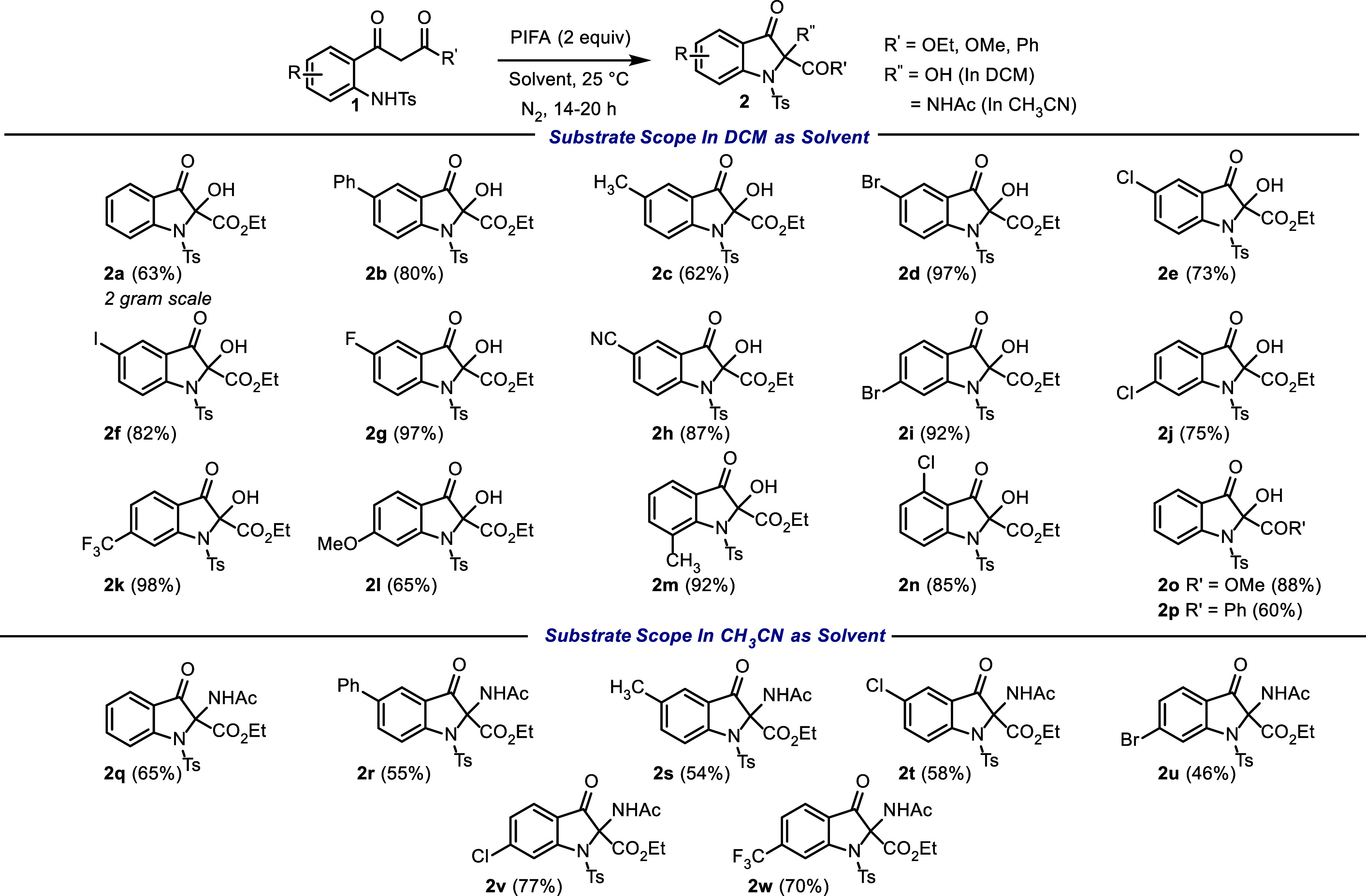
Umpolung Oxidative Cyclization: Scope of the Reaction

Especially remarkable are *p*-Br- and *p*-F-aminophenyl-3-oxopropanoate, delivering the corresponding products (**2d**–**g**) in 73–97% yield. Reactant **1** with an electron-withdrawing cyano group at the 4*-*position afforded the corresponding product **2h** in 87% yield. 5*-*Halogen substitutions on the phenyl ring were also well tolerated under the reaction conditions and delivered the desired products (**2i**, **2j**) in 92 and 75% yields, respectively. Having an electron-withdrawing CF_3_ substituent at the 5*-*position provided the cyclized product (**2k**) in 98% yield. Reactants with a 4*-*electron-donating methoxy group underwent the reaction, albeit in a reduced yield of 65% yield (**2l**). Reactant **1** with a methyl substituent at the 2*-*position also underwent the reaction, producing the corresponding product **2m** in 92% yield. 5-Chloro-substituted **1** furnished the corresponding indolin-3-one product (**2n**) in 85% yield. Interestingly, changing from ethyl ester to methyl ester produced the desired product (**2o**) in 88% yield. Additionally, β-diketone afforded 2-benzoyl-2-hydroxy-1-tosylindolin-3-one (**2p**) in 60% yield.

Next, we turned our focus to evaluate the feasibility of the reaction in acetonitrile as a solvent. We speculated that acetonitrile might act as a nucleophile to attack a second enolonium species (**Int6**, Figure 2), producing after aqueous workup hydrolysis, the 2-acetamido-indolin-3-one (Figure 1C). Using 2 equiv of PIFA and now using acetonitrile as a solvent, we expanded the scope, and the results are summarized in Scheme 1. Compound **1a** afforded the desired 2-amino-indolin-3-one **2q** in 65% yield. Compounds **1** with 4-phenyl and -methyl groups provided the corresponding products (**2r**, **2s**) in 55 and 54% yields, respectively. Reactant **1** with a chloride atom at the 4-position underwent the reaction and provided the corresponding product (**2t**) in 58% yield. Halogen substitution (Br or Cl) at the 5*-*position was tolerated under the reaction conditions and provided the corresponding indolin-3-one products (**2u**, **2v**) in 46 and 77% yield, respectively. The electron-withdrawing CF_3_ group at the 5*-*position afforded cyclized product **2w** in 70% yield.

**Figure 2:**
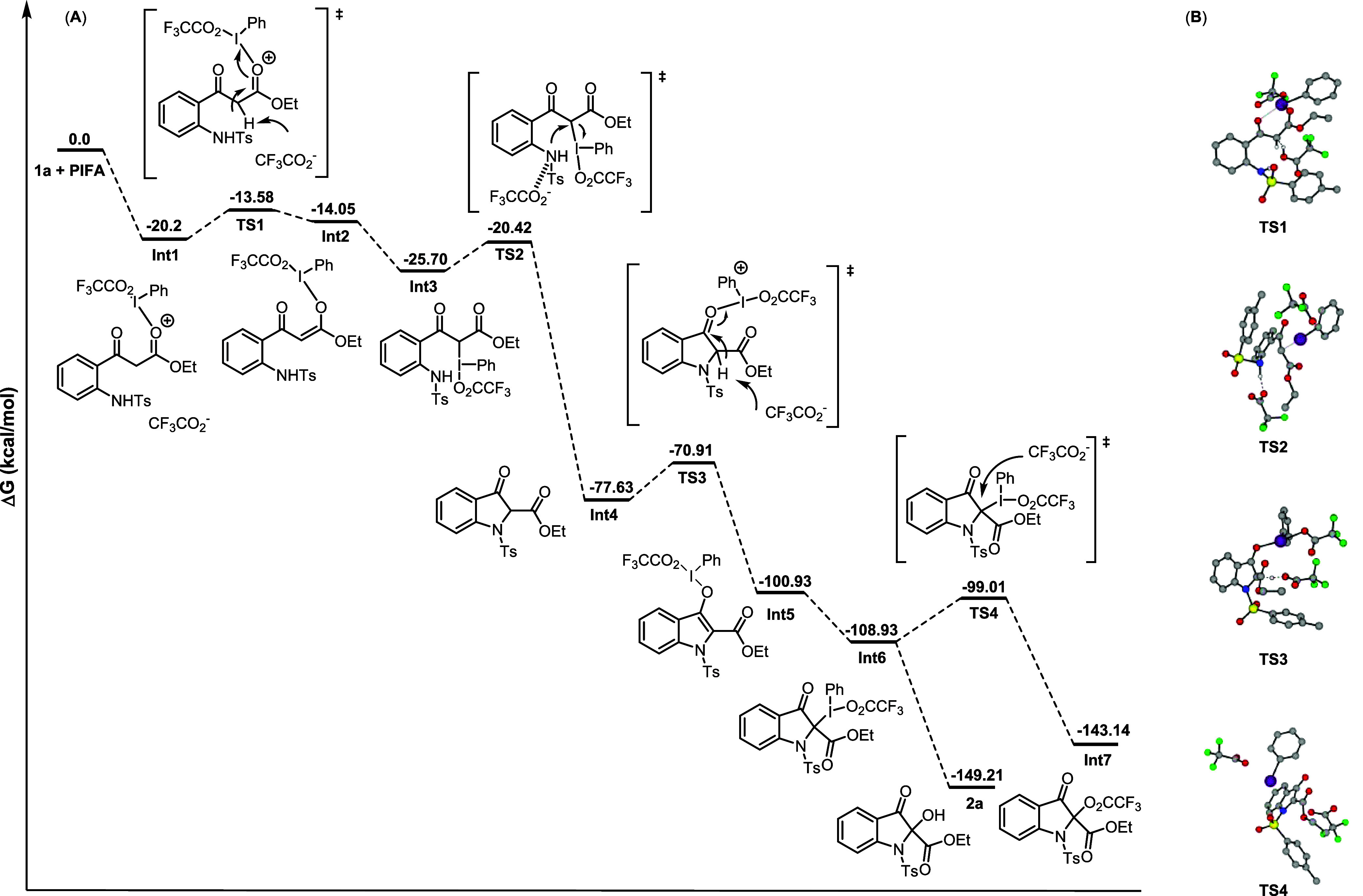
(A) Free energy (kcal/mol) profile for the formation of 2-trifluoroacetoxy-indolin-3-one (**Int7**) or product **2a** obtained at the SMD_(DCM)_/B3LYP/6-31G, LANL2DZ, level of theory. (B) Transition state structures (hydrogens are omitted for clarity).

To demonstrate the practicability of the newly developed approach, we carried out a gram-scale reaction (Scheme 1). Under the standard conditions, **2a** was isolated in 63%.

To illustrate the potential of 2-hydroxy-indolin-3-one to create diverse motifs, several downstream transformations of **2a** were carried out (Scheme 2). Esterification with acetyl chloride in reflux CH_2_Cl_2_ produced 2-acetoxy-indolin-3-one **3** in 69% yield. Chlorination with thionyl chloride and imidazole furnished 2-chloro-indolin-3-one **4** in 40% yield. Etherification with dimethylsulfate and cesium carbonate in a mixture of acetonitrile and DMF as solvent provided 2-methoxy-indolin-3-one **5** in 81% yield. Subsequent Corey-Chaykovsky reaction of **5** with TMSOI and sodium hydride in DMSO generated epoxide **6** at position 3 in 37% yield. *N*-Tosyl deprotection was performed using magnesium metal in methanol, and a subsequent third umpolung reaction employing PIFA as an oxidant and indole as the nucleophile provided C–C bond in the sterically hindered C2-position. This product (**7**) was obtained in an overall yield of 56%. Importantly, the yield of the third umpolung reaction furnished product **7** with a densely substituted quaternary center in a yield of 92%. Amide hydrolysis of **2q** with HCl produced 2-amino-indolin-3-one **8** in 61% yield.

**Scheme 2:**
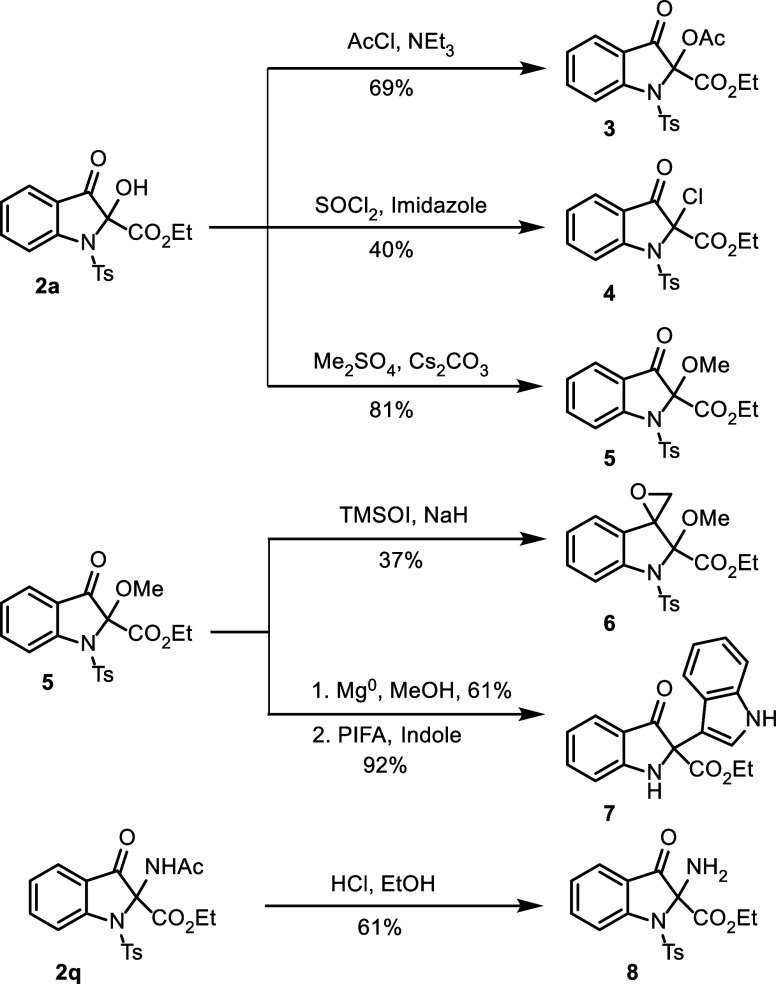
Synthetic Transformations

To shed light on the possible reaction mechanism, we performed DFT calculations using Gaussian16.^32,33^ For the standard reaction, computational studies were done at the SMD_(DCM)_/B3LYP/LANL2DZ, 6-31G level of theory (see the SI for computational details). The reaction can begin with a nucleophilic attack of the carbonyl oxygen of the ester moiety on the hypervalent iodine center, leading to **Int1** (Δ*G*° = −20.2 kcal/mol). Since most of the reactions with PhIL_2_ and a nucleophile follow a heterolytic pathway,^35^ and the bonding situation in neutral I­(III) compounds is accurately described as an ionic pair in two mesomeric species,^36,37^ PIFA was considered as an ion pair of [Ph-I-O_2_CCF_3_]^+^[O_2_CCF_3_]^−^. Natural Bond Orbital (NBO) analysis revealed a more negative charge on the carbonyl oxygen of the ester moiety (−0.598 au) than that of the ketone (−0.594 au). Thus, the carbonyl ester moiety attacks preferentially the [Ph-I-O_2_CCF_3_]^+^ species. The resulting adduct (**Int1**) is an iodinated intermediate interacting with trifluoroacetate (CF_3_COO^–^) through acidic methylene hydrogen. Ensuing deprotonation of the methylenic C–H of **Int1** by the CF_3_COO^–^ via a transition state barrier (**TS1**) of 6.62 kcal/mol leads to the formation of an O-iodoenolate **Int2** (Δ*G*° = 6.14 kcal/mol). Along this path, 1,3-migration of the [Ph-I-O_2_CCF_3_]^+^ fragment from the O-iodoenolate to the α C-iodoenolate generates a lower-energy C-iodoenolate species **Int3** without a barrier (Δ*G*° = −11.65 kcal/mol). **Int3** subsequently undergoes intramolecular cyclization by a nucleophilic attack of the *ortho*-NH group on the α C-iodoenolate, resulting in C–N bond formation. This exergonic step triggers the formation of intermediate **Int4** (Δ*G*° = −51.9 kcal/mol) with an energy barrier of 5.28 kcal/mol (**TS2**). The change in the oxidation state of iodine (III to I) and the expulsion of neutral species (PhI and CF_3_COOH) provide a noticeable thermodynamic drive for this reaction. **Int4** further interacts with a second equivalent of PIFA to form another O-iodoenolate species **Int5** (Δ*G*° = −23.3 kcal/mol) through **TS3**. The activation energy barrier for this step is 6.72 kcal/mol. Important to note, in this step, the enolonium species is formed with the carbonyl ketone and not with the carbonyl ester moiety since it forms a product (**Int5**) which is lower in energy (see the SI for comparison). In addition, this enolonium species (**Int5**) is an aromatic structure, while enolonium species of the carbonyl ester is not. Succeeding 1,3-migration of [Ph–I-O_2_CCF_3_]^+^ from the O-iodoenolate to the α C-iodoenolate atom generates a lower-energy C-iodoenolate **Int6** (Δ*G*° = −8.0 kcal/mol). This step is barrierless. Attack of the CF_3_COO^–^ ion on enolonium species **Int6** through **TS4** readily gives **Int7** (Δ*G*° = −34.21 kcal/mol). This step is quite fast, as it exhibits a low barrier of only 9.92 kcal/mol, and results in the formation of PhI, CF_3_COO^–^, and the final 2-trifluoroacetoxy-indolin-3-one (**Int7**). Although CF_3_COO^–^ is a weak nucleophile, since no other nucleophile exists in the reaction mixture, it is feasible to get **Int7** as an intermediate. **Int7** was not detected experimentally; it may be attributed to the fast reaction from **Int6** to **Int7**, but it cannot be excluded that **Int7** is not formed during the process. Upon water workup, hydrolysis of the trifluoroacetate moiety furnishes the final hydroxy product (**2a**). The second pathway is H_2_O attack on **Int6** during the workup process that will lead directly to product **2a**.

In summary, we have developed an efficient one-pot approach for the cyclization of 2-aminophenyl-3-oxopropanoate via an umpolung reaction, yielding synthetically valuable 2,2-disubstituted indolin-3-ones. In comparison to other methods, this protocol relies on simple substrates and has a broad substrate scope (23 examples). DFT calculations provided insight into the reaction mechanism and support the formation of key C-enolonium species.

## Experimental Section

### General Information

Unless otherwise noted, all reagents were purchased from commercial suppliers and used without further purification. All solvents were dried according to standard procedures and techniques before use. Column chromatography was performed on silica gel. ^1^H NMR spectroscopy measurements were carried out on Bruker 400 MHz NMR spectrometers with CDCl_3_ (δ 7.26) as an internal standard unless otherwise stated. The ^13^C NMR spectra were recorded on a 101 MHz NMR spectrometer with CDCl_3_ (δ 77.16) as an internal standard unless otherwise stated. HRMS spectra were acquired on an Xevo G2-XS QTof device mass spectrometer. IR spectra were obtained using an FT/IR-4700 type A with a resolution of 16 cm^–1^. Melting point was measured on Stuart SMP50 version 1.12.

**Caution!** PIFA is an oxidizing agent. While no data exist for PIFA, some hypervalent iodine reagents are potentially explosive.^38,39^ PIFA is a mild irritant. Appropriate precautions should be taken.

#### General Procedure A for the Umpolung Oxidative Cyclization in CH_2_Cl_2_

Compounds **1a**–**n** (1 equiv) were dissolved in 12 mL of dry DCM (0.02 M). PIFA (2 equiv) was added portion-wise to the reaction mixture at room temperature. The reaction was allowed to stir overnight. The mixture was extracted 3 times with 10 mL water and DCM (3 × 30 mL) and dried over sodium sulfate. The product was purified by silica gel column chromatography using EtOAc/Hexane as eluents.

#### General Procedure B for the Umpolung Oxidative Cyclization in CH_3_CN

Compounds **1a**–**c**, **1e**, and **1i**–**k** were dissolved in 12 mL of dry CH_3_CN (0.02–0.03 M). PIFA (2 equiv) was added portion-wise at room temperature. The reaction was allowed to stir overnight. Next, 10 mL of water was added to the mixture and allowed to stir for 30 min. The mixture was extracted 3 times with 10 mL of water and DCM (3 × 30 mL) and dried over sodium sulfate. The product was purified by column chromatography with ethyl acetate and hexane as the eluents. Ethyl 2-hydroxy-3-oxo-1-tosylindoline-2-carboxylate (**2a**). According to general procedure A, **1**(**a**) (100 mg, 0.27 mmol) and PIFA (238 mg, 0.54 mmol, 2 equiv) were used in 12 mL of dry DCM (0.02 M). The crude residue was purified by silica gel column chromatography using EtOAc:hexane (20:80, v:v) to afford a brown color solid, 101 mg, 97% yield. ^**1**^ **H NMR** (400 MHz, CDCl_3_) δ 7.98 (m, 2H), 7.70 (m, 1H), 7.63–7.51 (m, 2H), 7.31 (m, 2H), 7.17–7.11 (bs, 1H), 5.33 (s, 1H), 4.43 (m, 1H), 4.31 (m, 1H), 2.40 (s, 3H), 1.29 (t, *J* = 8 Hz, 3H). ^**13**^ **C­{** ^**1**^ **H} NMR** (101 MHz, CDCl_3_) δ: 191.2, 167.3, 152.4, 145.1, 138.3, 136.1, 129.9, 128.0, 125.9, 123.9, 120.3, 87.3, 64.5, 21.7, 14.0. **HRMS** (ESI) *m*/*z*: [M + Na]^+^ Calcd for C_18_H_17_NNaO_6_S 398.0674; found: 398.0668. **mp** 108–111 °C.

#### Ethyl 2-Hydroxy-3-oxo-5-phenyl-1-tosylindoline-2-carboxylate (**2b**)

According to general procedure A, **1**(**b**) (100 mg, 0.23 mmol) and PIFA (197 mg, 0.46 mmol, 2 equiv) were used in 12 mL of dry DCM (0.02 M). The crude residue was purified by silica gel column chromatography using EtOAc:hexane (20:80, v:v) to afford an amorphous yellow color solid, 82 mg, 80% yield. ^**1**^ **H NMR** (400 MHz, CDCl_3_) δ 8.01 (m, 2H), 7.90 (m, 1H), 7.85 (m, 1H), 7.64 (m, 1H), 7.53–7.49 (m, 2H), 7.46–7.41 (m, 2H), 7.39–7.31 (m, 3H), 5.42 (s, 1H), 4.45 (m, 1H), 4.33 (m, 1H), 2.41 (s, 3H), 1.31 (t, *J* = 8 Hz, 3H). ^**13**^ **C­{** ^**1**^ **H} NMR** (101 MHz, CDCl_3_) δ: 191.3, 167.2, 151.5, 145.2, 138.8, 137.5, 137.3, 136.1, 130.0, 129.1, 128.0, 126.8, 123.8, 120.8, 114.1, 87.8, 64.6, 21.7, 14.0. **HRMS** (ESI) *m*/*z*: [M + Na]^+^ Calcd for C_24_H_21_NNaO_6_S 474.0987; found: 474.0998. **IR** 3031, 1761, 1725, 1611, 1470, 1355, 1261, 1157, 1084, 958 cm^–1^.

#### Ethyl 2-Hydroxy-5-methyl-3-oxo-1-tosylindoline-2-carboxylate (**2c**)

According to general procedure A, **1**(**c**) (100 mg, 0.27 mmol) and PIFA (229 mg, 0.54 mmol, 2 equiv) were used in 12 mL of dry DCM (0.02 M). The crude residue was purified by silica gel column chromatography using EtOAc:hexane (15:85, v:v) to afford a yellow color solid, 64 mg, 62% yield. ^**1**^ **H NMR** (400 MHz, CDCl_3_) δ 7.96 (m, 2H), 7.44 (m, 3H), 7.30 (m, 2H), 5.33 (s, 1H), 4.41 (m, 1H), 4.30 (m, 1H), 2.39 (s, 3H), 2.32 (s, 3H), 1.28 (t, *J* = 8 Hz, 3H). ^**13**^ **C­{** ^**1**^ **H} NMR** (101 MHz, CDCl_3_) δ: 191.3, 167.3, 150.5, 145.0, 139.4, 136.2, 133.9, 129.9, 128.0, 125.5, 120.3, 113.6, 87.5, 64.4, 21.7, 20.6, 13.9. **HRMS** (ESI) *m*/*z*: [M + Na]^+^ Calcd for C_19_H_19_NNaO_6_S 412.0831; found: 412.0843 **IR** 2926, 2848, 2304, 1761, 1724, 1617, 1487, 1362, 1247, 1141, 916, 672 cm^–1^. **mp** 109–112 °C.

#### Ethyl 5-Bromo-2-hydroxy-3-oxo-1-tosylindoline-2-carboxylate (**2d**)

According to general procedure A, **1**(**d**) (100 mg, 0.23 mmol) and PIFA (195 mg, 0.46 mmol, 2 equiv) were used in 12 mL of dry DCM (0.02 M). The crude residue was purified by silica gel column chromatography using EtOAc:hexane (20:80, v:v) to afford a light orange color solid, 100 mg, 97% yield. ^**1**^ **H NMR** (400 MHz, CDCl_3_) δ 7.98–7.93 (m, 2H), 7.82–7.78 (m, 1H), 7.68 (m, 1H), 7.50–7.45 (m, 1H), 7.32 (m, 2H), 5.37 (s, 1H), 4.42 (m, 1H), 4.31 (m, 1H), 2.41 (s, 3H), 1.29 (t, *J* = 8 Hz, 3H). ^**13**^ **C­{** ^**1**^ **H} NMR** (101 MHz, CDCl_3_) δ: 190.1, 166.8, 151.2, 145.4, 140.8, 135.8, 130.0, 128.3, 128.0, 121.8, 116.8, 115.4, 87.6, 64.7, 21.7, 14.0. **HRMS** (ESI) *m*/*z*: [M + H]^+^ Calcd for C_18_H_17_BrNO_6_S 453.9960; found: 453.9954. **IR** 2926, 2853, 1759, 1730, 1593, 1453, 1364, 1247, 1150, 1128, 1079, 951 cm^–1^. **mp** 114–117 °C.

#### Ethyl 5-Chloro-2-hydroxy-3-oxo-1-tosylindoline-2-carboxylate (**2e**)

**1**(**e**) (100 mg, 0.25 mmol) and PIFA (218 mg, 0.5 mmol, 2 equiv) were used in 12 mL of dry DCM (0.02 M). The crude residue was purified by silica gel column chromatography using EtOAc:hexane (12:82, v:v) to afford an orange color solid, 76 mg, 73% yield. ^**1**^ **H NMR** (400 MHz, CDCl_3_) δ 7.95 (m, 2H), 7.64 (m, 1H), 7.57–7.51 (m, 2H), 7.31 (m, 2H), 5.40 (s, 1H), 4.42 (m, 1H), 4.31 (m, 1H), 2.40 (s, 3H), 1.29 (t, *J* = 8 Hz, 3H). ^**13**^ **C­{** ^**1**^ **H} NMR** (101 MHz, CDCl_3_) δ: 190.2, 166.8, 150.7, 145.4, 138.0, 135.8, 130.0, 129.7, 128.0, 125.2, 121.4, 115.1, 87.7, 64.7, 21.7, 13.9. **HRMS** (ESI) *m*/*z*: [M + Na]^+^ Calcd for C_18_H_16_ClNNaO_6_S 432.0284; found: 432.0286. **IR** 3358, 3009, 1729, 1738, 1364, 1150, and 963 cm^–1^. **mp** 117–120 °C.

#### Ethyl 2-Hydroxy-5-iodo-3-oxo-1-tosylindoline-2-carboxylate (**2f**)

According to general procedure A, **1**(**f**) (100 mg, 0.21 mmol) and PIFA (176 mg, 0.42 mmol, 2 equiv) were used in 12 mL of dry DCM (0.02 M). The crude residue was purified by silica gel column chromatography using EtOAc:hexane (20:80, v:v) to afford a yellow color solid, 85 mg, 82% yield. ^**1**^ **H NMR** (400 MHz, CDCl_3_) δ 7.97 (m, 3H), 7.85 (m, 1H), 7.38–7.28 (m, 3H), 5.34 (s, 1H), 4.42 (m, 1H), 4.31 (m, 1H), 2.41 (s, 3H), 1.29 (t, *J* = 8 Hz, 3H). ^**13**^ **C­{** ^**1**^ **H} NMR** (101 MHz, CDCl_3_) δ: 189.9, 166.8, 151.8, 146.4, 145.4, 135.8, 134.3, 130.0, 128.0, 122.3, 115.8, 87.3, 86.4, 64.7, 21.7, 14.0. **HRMS** (ESI) *m*/*z*: [M + H]^+^ Calcd for C_18_H_17_INO_6_S 501.9821; found: 501.9823 **IR** 3235, 3040, 1752, 1728, 1591, 1450, 1357, 1252, 1128, 1078, 1007, 949 cm^–1^. **mp** 125–128 °C.

#### Ethyl 5-Fluoro-2-hydroxy-3-oxo-1-tosylindoline-2-carboxylate (**2g**)

According to general procedure A, **1**(**g**) (100 mg, 0.26 mmol) and PIFA (226 mg, 0.52 mmol, 2 equiv) were used in 12 mL of dry DCM (0.02 M). The crude residue was purified by silica gel column chromatography using EtOAc:hexane (15:85, v:v) to afford a yellow color solid, 101 mg, 97% yield. ^**1**^ **H NMR** (400 MHz, CDCl_3_) δ 7.99–7.92 (m, 2H), 7.55 (m, 1H), 7.38–7.28 (m, 4H), 5.35 (s, 1H), 4.43 (m, 1H), 4.32 (m, 1H), 2.41 (s, 3H), 1.30 (t, *J* = 8 Hz, 3H). ^**13**^ **C­{** ^**1**^ **H} NMR** (101 MHz, CDCl_3_) δ: 190.7, 190.7, 166.9, 160.3, 157.8, 148.7, 148.7, 145.3, 135.9, 130.0, 128.0, 125.8, 125.6, 121.3, 121.3, 115.3, 115.2, 111.6, 111.3, 87.9, 64.6, 21.7, 14.0. ^**19**^ **F NMR** (376 MHz, CDCl_3_) δ −118.01. **HRMS** (ESI) *m*/*z*: [M + Na]^+^ Calcd for C_18_H_16_FNNaO_6_S 416.0580; found: 416.0589. **IR** 3372, 2928, 1739, 1729, 1477, 905 cm^–1^. **mp** 131–134 °C.

#### Ethyl 5-Cyano-2-hydroxy-3-oxo-1-tosylindoline-2-carboxylate (**2h**)

According to general procedure A, **1**(**h**) (100 mg, 0.26 mmol) and PIFA (222 mg, 0.52 mmol, 2 equiv) were used in 12 mL of dry DCM (0.02 M). The crude residue was purified by silica gel column chromatography using EtOAc:hexane (20:80, v:v) to afford an orange color oil, 90 mg, 87% yield. ^**1**^ **H NMR** (400 MHz, CDCl_3_) δ 8.00–7.93 (m, 3H), 7.84 (m, 1H), 7.68 (m, 1H), 7.36–7.31 (m, 2H), 4.44 (m, 1H), 4.31 (m, 1H), 2.41 (s, 3H), 1.29 (t, *J* = 8 Hz, 3H). ^**13**^ **C­{** ^**1**^ **H} NMR** (101 MHz, CDCl_3_) δ: 189.7, 166.3, 154.4, 145.9, 141.0, 135.2, 130.2, 130.1, 128.0, 120.6, 117.3, 114.6, 107.6, 87.8, 64.9, 21.7, 13.9. **HRMS** (ESI) *m*/*z*: [M + Na]^+^ Calcd for C_19_H_16_N_2_NaO_6_S 423.0627; found: 423.0621. **IR** 3312, 3028, 2233, 1741, 1616, 1580, and 900 cm^–1^.

#### Ethyl 6-Bromo-2-hydroxy-3-oxo-1-tosylindoline-2-carboxylate (**2i**)

According to general procedure A, **1**(**i**) (100 mg, 0.22 mmol) and PIFA (195 mg, 0.44 mmol, 2 equiv) were used in 12 mL of dry DCM (0.02 M). The crude residue was purified by silica gel column chromatography using EtOAc:hexane (25:75, v:v) to afford an orange color solid, 95 mg, 92% yield. ^**1**^ **H NMR** (400 MHz, CDCl_3_) δ 7.99–7.94 (m, 2H), 7.76 (m, 1H), 7.54 (m, 1H), 7.37–7.31 (m, 2H), 7.28 (m, 1H), 5.39 (bs, 1H), 4.42 (m, 1H), 4.30 (m, 1H), 2.41 (s, 3H), 1.29 (t, *J* = 8 Hz, 3H). ^**13**^ **C­{** ^**1**^ **H} NMR** (101 MHz, CDCl_3_) δ 190.2, 166.8, 152.8, 145.4, 135.7, 134.0, 130.1, 128.0, 127.5, 126.6, 119.1, 117.2, 87.6, 64.6, 21.7, 13.9. **HRMS** (ESI) *m*/*z*: [M + H]^+^ Calcd for C_18_H_17_BrNO_6_S 453.9960; found: 453.9954. **IR** 3434, 3109, 1722, 1589, 1153, 952 cm^–1^.

#### Ethyl 6-Chloro-2-hydroxy-3-oxo-1-tosylindoline-2-carboxylate (**2j**)

According to general procedure A, **1**(**j**) (100 mg, 0.25 mmol) and PIFA (218 mg, 0.5 mmol, 2 equiv) were used in 12 mL of dry DCM (0.02 M). The crude residue was purified by silica gel column chromatography using EtOAc:hexane (17:83, v:v) to afford an orange color solid, 78 mg, 75% yield. ^**1**^ **H NMR** (400 MHz, CDCl_3_) δ 7.97 (d, 2H), 7.64–7.60 (m, 1H), 7.59–7.57 (m, 1H), 7.34 (m, 2H), 7.12 (m, 1H), 4.43 (m, 1H), 4.31 (m, 1H), 2.42 (s, 3H), 1.29 (t, *J* = 8 Hz, 3H). ^**13**^ **C­{** ^**1**^ **H} NMR** (101 MHz, CDCl_3_) δ: 189.9, 166.9, 152.9, 145.5, 145.1, 135.7, 130.1, 128.0, 126.7, 124.6, 118.7, 114.3, 87.8, 64.7, 21.7, 14.0. **HRMS** (ESI) *m*/*z*: [M + H]^+^ Calcd for C_18_H_17_ClNO_6_S 410.0465; found: 410.0479. **IR** 3449, 3014, 1733, 1599, 1153, 955 cm^–1^. **mp** 122–125 °C.

#### Ethyl 2-Hydroxy-3-oxo-1-tosyl-6-(trifluoromethyl)­indoline-2-carboxylate (**2k**)

According to general procedure A, **1**(**k**) (100 mg, 0.23 mmol) and PIFA (200 mg, 0.46 mmol, 2 equiv) were used in 12 mL of dry DCM (0.02 M). The crude residue was purified by silica gel column chromatography using EtOAc:hexane (14:86, v:v) to afford an orange color solid, 102 mg, 98% yield. ^**1**^ **H NMR** (400 MHz, CDCl_3_) δ 8.00–7.95 (m, 2H), 7.85–7.78 (m, 2H), 7.39 (m, 1H), 7.34 (m, 2H), 5.46 (s, 1H), 4.44 (m, 1H), 4.32 (m, 1H), 2.42 (s, 3H), 1.30 (t, *J* = 8 Hz, 3H). ^**13**^ **C­{** ^**1**^ **H} NMR** (101 MHz, CDCl_3_) δ: 190.8, 166.7, 152.2, 145.7, 139.6, 139.3, 139.0, 138.6, 135.5, 130.1, 128.0, 126.5, 122.6, 121.6, 120.7, 120.7, 111.1, 111.0, 111.0, 111.0, 87.6, 64.8, 21.7, 14.0. ^**19**^ **F NMR** (376 MHz, CDCl_3_) δ −63.49. **HRMS** (ESI) *m*/*z*: [M + Na]^+^ Calcd for C_19_H_16_F_3_NNaO_6_S 466.0548; found: 466.0557. **IR** 3420, 2985, 1748, 1604, 1157, 972 cm^–1^. **mp** 125–128 °C.

#### Ethyl 2-Hydroxy-6-methoxy-3-oxo-1-tosylindoline-2-carboxylate (**2l**)

According to general procedure A, **1**(**l**) (100 mg, 0.25 mmol) and PIFA (220 mg, 0.5 mmol, 2 equiv) were used in 12 mL of dry DCM (0.02 M). The crude residue was purified by silica gel column chromatography using EtOAc:hexane (25:75, v:v) to afford a light yellow color solid, 67 mg, 65% yield. ^**1**^ **H NMR** (400 MHz, CDCl_3_) δ 7.97 (m, 2H), 7.61 (m, 1H), 7.31 (m, 2H), 7.00 (m, 1H), 6.65 (m, 1H), 5.34 (s, 1H), 4.42 (m, 1H), 4.30 (m, 1H), 3.87 (s, 3H), 2.40 (s, 3H), 1.29 (t, *J* = 8 Hz, 3H). ^**13**^ **C­{** ^**1**^ **H} NMR** (101 MHz, CDCl_3_) δ: 188.8, 168.2, 167.4, 154.6, 145.1, 136.1, 129.9, 128.0, 127.6, 113.4, 111.6, 98.5, 88.1, 64.4, 56.1, 21.7, 14.0. **HRMS** (ESI) *m*/*z*: [M + H]^+^ Calcd for C_19_H_20_NO_7_S 406.0960; found: 406.0956. **IR** 3396, 3034, 1724, 1604, 1585, 967 cm^–1^. **mp** 133–136 °C.

#### Ethyl 2-Hydroxy-7-methyl-3-oxo-1-tosylindoline-2-carboxylate (**2m**)

According to general procedure A, **1**(**m**) (100 mg, 0.26 mmol) and PIFA (229 mg, 0.52 mmol, 2 equiv) were used in 12 mL of dry DCM (0.02 M). The crude residue was purified by silica gel column chromatography using EtOAc:hexane (20:80, v:v) to afford a yellow color solid, 95 mg, 92% yield. ^**1**^ **H NMR** (400 MHz, CDCl_3_) δ 7.98–7.92 (m, 2H), 7.63 (m, 1H), 7.38–7.29 (m, 3H), 7.09 (m, 1H), 5.37 (s, 1H), 4.44 (m, 1H), 4.27 (m, 1H), 2.42 (s, 3H), 2.16 (s, 3H), 1.29 (t, *J* = 8 Hz, 3H). ^**13**^ **C­{** ^**1**^ **H} NMR** (101 MHz, CDCl_3_) δ: 191.7, 167.2, 151.4, 144.6, 142.6, 138.5, 130.0, 127.1, 125.5, 124.6, 123.7, 122.5, 88.6, 64.3, 21.7, 21.7, 13.9. **HRMS** (ESI) *m*/*z*: [M + H]^+^ Calcd for C_19_H_20_NO_6_S 390.1011; found: 390.1000. **IR** 3436, 3040,1753, 1722, 1148, 967 cm^–1^. **mp** 131–134 °C.

#### Ethyl 4-Chloro-2-hydroxy-3-oxo-1-tosylindoline-2-carboxylate (**2n**)

According to general procedure A, **1**(**n**) (100 mg, 0.24 mmol) and PIFA (218 mg, 0.48 mmol, 2 equiv) were used in 12 mL of dry DCM (0.02 M). The crude residue was purified by silica gel column chromatography using EtOAc:hexane (17:83, v:v) to afford a yellow color solid, 88 mg, 85% yield. ^**1**^ **H NMR** (400 MHz, CDCl_3_) δ 8.00–7.96 (m, 2H), 7.50–7.43 (m, 2H), 7.32 (m, 2H), 7.08 (m, 1H), 5.42 (s, 1H), 4.46 (m, 1H), 4.31 (m, 1H), 2.41 (s, 3H), 1.31 (t, *J* = 8 Hz, 3H). ^**13**^ **C­{** ^**1**^ **H} NMR** (101 MHz, CDCl_3_) δ: 188.5, 166.9, 153.5, 145.4, 138.2, 135.7, 134.0, 130.0, 128.1, 125.2, 117.1, 111.9, 87.3, 64.7, 21.7, 14.0. **HRMS** (ESI) *m*/*z*: [M + Na]^+^ Calcd for C_18_H_16_ClNNaO_6_S 432.0284; found: 432.0291. **IR** 3378, 2932, 1786, 1750, 1080, and 957 cm^–1^. **mp** 124–127 °C.

#### Methyl 2-Hydroxy-3-oxo-1-tosylindoline-2-carboxylate (**2o**)

According to general procedure A, **1**(**o**) (100 mg, 0.28 mmol) and PIFA (247 mg, 0.56 mmol, 2 equiv) were used in 12 mL of dry DCM (0.02 M). The crude residue was purified by silica gel column chromatography using EtOAc:hexane (35:65, v:v) to afford a yellow color solid, 92 mg, 88% yield. ^**1**^ **H NMR** (400 MHz, CDCl_3_) δ 7.98 (m, 2H), 7.71–7.55 (m, 3H), 7.31 (m, 2H), 7.18–7.11 (m, 1H), 5.35 (s, 1H), 3.87 (s, 3H), 2.39 (s, 3H). ^**13**^ **C­{** ^**1**^ **H} NMR** (101 MHz, CDCl_3_) δ: 191.0, 167.7, 152.3, 145.2, 138.4, 135.9, 129.9, 128.0, 125.9, 124.0, 120.2, 113.8, 87.3, 54.7, 21.7. **HRMS** (ESI) *m*/*z*: [M + Na]^+^ Calcd for C_17_H_15_NNaO_6_S 384.0518; found: 384.0507. **IR** 2925, 1749, 1593, 1246, and 951 cm^–1^. **mp** 149–152 °C.

#### 2-Benzoyl-2-hydroxy-1-tosylindolin-3-one (**2p**)

According to general procedure A, **1**(**p**) (26 mg, 0.07 mmol) and PIFA (58 mg, 0.14 mmol, 2 equiv) were used in 12 mL of dry DCM (6 mM). The crude residue was purified by silica gel column chromatography using EtOAc:hexane (15:85, v:v) to afford an amorphous yellow color solid, 16 mg, 60% yield. ^**1**^ **H NMR** (400 MHz, CDCl_3_) δ 8.02–7.95 (m, 2H), 7.86–7.76 (m, 3H), 7.73–7.68 (m, 2H), 7.60–7.54 (m, 1H), 7.40–7.33 (m, 2H), 7.29 (m, 2H), 7.25–7.21 (m, 1H), 6.47 (s, 1H), 2.40 (s, 3H). ^**13**^ **C­{** ^**1**^ **H} NMR** (101 MHz, CDCl_3_) δ: 191.7, 191.0, 152.1, 145.2, 138.5, 135.7, 134.7, 131.7, 129.8, 129.2, 129.1, 128.4, 126.3, 124.4, 120.8, 114.6, 90.4, 21.7. **HRMS** (ESI) *m*/*z*: [M + Na]^+^ Calcd for C_22_H_17_NO_5_SNa 430.0725; found: 430.0706. **IR** 3070, 2923, 1732, 1683, 1359, 753 cm^–1^.

#### Ethyl 2-Acetamido-3-oxo-1-tosylindoline-2-carboxylate (**2q**)

According to general procedure B, **1**(**a**) (140 mg, 0.39 mmol) and PIFA (333 mg, 0.78 mmol, 2 equiv) were used in 12 mL of dry DCM (0.03 M). The crude residue was purified by silica gel column chromatography using EtOAc:hexane (40:60, v:v) to afford a white color solid, 105 mg, 65% yield. ^**1**^ **H NMR** (400 MHz, CDCl_3_) δ 7.79–7.72 (m, 4H), 7.63 (m, 1H), 7.36 (s, 1H), 7.28 (m, 2H), 7.17 (m, 1H), 4.37 (m, 1H), 4.27–4.18 (m, 1H), 2.39 (s, 3H), 1.76 (s, 3H), 1.24 (t, *J* = 8 Hz, 3H). ^**13**^ **C­{** ^**1**^ **H} NMR** (101 MHz, CDCl_3_) δ: 188.9, 168.9, 164.9, 151.6, 144.9, 137.3, 137.2, 130.0, 126.6, 125.0, 123.6, 122.2, 113.6, 77.7, 64.3, 22.1, 21.7, 13.8. **HRMS** (ESI) *m*/*z*: [M + Na]^+^ Calcd for C_20_H_20_N_2_NaO_6_S 439.0940; found: 439.0948 **IR** 2249, 1759, 1728, 1666, 1602, 1458, 1357, 1214, 1153, 1081 cm^–1^. **mp** 209–212 °C.

#### Ethyl 2-Acetamido-3-oxo-5-phenyl-1-tosylindoline-2-carboxylate (**2r**)

According to general procedure B, **1**(**b**) (130 mg, 0.29 mmol) and PIFA (256 mg, 0.58 mmol, 2 equiv) were used in 12 mL of dry DCM (0.02 M). The crude residue was purified by silica gel column chromatography using EtOAc:hexane (40:60, v:v) to afford a yellow color solid, 81 mg, 55% yield. ^**1**^ **H NMR** (400 MHz, CDCl_3_) δ 7.94 (m, 1H), 7.86 (m, 2H), 7.80 (m, 2H), 7.57–7.53 (m, 2H), 7.49–7.41 (m, 3H), 7.38–7.33 (m, 1H), 7.31–7.28 (m, 2H), 4.39 (m, 1H), 4.25 (m, 1H), 2.39 (s, 3H), 1.77 (s, 3H), 1.27 (t, *J* = 8 Hz, 3H). ^**13**^ **C­{** ^**1**^ **H} NMR** (101 MHz, CDCl_3_) δ: 188.9, 169.0, 164.8, 150.7, 145.0, 139.2, 137.1, 137.1, 136.3, 130.0, 129.0, 127.8, 126.9, 126.6, 123.0, 122.7, 113.9, 78.1, 64.3, 22.0, 21.6, 13.8. **HRMS** (ESI) *m*/*z*: [M + H]^+^ Calcd for C_26_H_25_N_2_O_6_S 493.1433; found: 493.1473. **IR** 3365, 3180, 1755, 1738, 1654, 1144, and 961 cm^–1^. **mp** 180–183 °C.

#### Ethyl 2-Acetamido-5-methyl-3-oxo-1-tosylindoline-2-carboxylate (**2s**)

According to general procedure B, **1**(**c**) (130 mg, 0.35 mmol) and PIFA (298 mg, 0.7 mmol, 2 equiv) were used in 12 mL of dry DCM (0.03 M). The crude residue was purified by silica gel column chromatography using EtOAc:hexane (40:60, v:v) to afford a white color solid, 80 mg, 54% yield. ^**1**^ **H NMR** (400 MHz, CDCl_3_) δ 7.77–7.73 (m, 2H), 7.65 (m, 1H), 7.52 (m, 1H), 7.43 (m, 1H), 7.37 (s, 1H), 7.28–7.24 (m, 2H), 4.35 (m, 1H), 4.22 (m, 1H), 2.38 (s, 3H), 2.35 (s, 3H), 1.74 (s, 3H), 1.24 (t, *J* = 8 Hz, 3H). ^**13**^ **C­{** ^**1**^ **H} NMR** (101 MHz, CDCl_3_) δ: 188.9, 168.8, 165.0, 149.7, 144.7, 138.42, 137.37, 133.53, 129.92, 126.60, 124.82, 122.30, 113.41, 77.9, 64.2, 22.0, 21.6, 20.6, 13.8. **HRMS** (ESI) *m*/*z*: [M + H]^+^ Calcd for C_21_H_23_N_2_O_6_S 431.1277; found: 431.1290. **IR** 3309, 3034, 1753, 1662, 1620, 1150, and 965 cm^–1^. **mp** 210–213 °C.

#### Ethyl 2-Acetamido-5-chloro-3-oxo-1-tosylindoline-2-carboxylate (**2t**)

According to general procedure B, **1**(**e**) (140 mg, 0.35 mmol) and PIFA (304 mg, 0.7 mmol, 2 equiv) were used in 12 mL of dry DCM (0.03 M). The crude residue was purified by silica gel column chromatography using EtOAc:hexane (40:60, v:v) to afford a white color solid, 92 mg, 58% yield. ^**1**^ **H NMR** (400 MHz, CDCl_3_) δ 7.73–7.67 (m, 3H), 7.65–7.63 (m, 1H), 7.53 (m, 1H), 7.35 (s, 1H), 7.25 (m, 2H), 4.33 (m, 1H), 4.20 (m, 1H), 2.37 (s, 3H), 1.70 (s, 3H), 1.21 (t, *J* = 8 Hz, 3H). ^**13**^ **C­{** ^**1**^ **H} NMR** (101 MHz, CDCl_3_) δ: 187.8, 169.0, 164.4, 149.9, 145.2, 137.0, 136.9, 130.0, 129.3, 126.6, 124.5, 123.4, 114.9, 78.1, 64.4, 21.9, 21.7, 13.8. **HRMS** (ESI) *m*/*z*: [M + H]^+^ Calcd for C_20_H_20_ClN_2_O_6_S 451.0731; found: 451.0764. **IR** 3014, 2921, 1759, 1684, 1607, 1461, 1364, 1269, 1210, 1163, 1086, 1011, 886 cm^–1^. **mp** 192–195 °C.

#### Ethyl 2-Acetamido-6-bromo-3-oxo-1-tosylindoline-2-carboxylate (**2u**)

According to general procedure B, **1**(**i**) (130 mg, 0.29 mmol) and PIFA (254 mg, 0.58 mmol, 2 equiv) were used in 12 mL of dry DCM (0.02 M). The crude residue was purified by silica gel column chromatography using EtOAc:hexane (35:65, v:v) to afford a white color solid, 68 mg, 46% yield. ^**1**^ **H NMR** (400 MHz, CDCl_3_) δ 8.00 (m, 1H), 7.77–7.73 (m, 2H), 7.59–7.55 (m, 1H), 7.35–7.28 (m, 4H), 4.37 (m, 1H), 4.23 (m, 1H), 2.41 (s, 3H), 1.73 (s, 3H), 1.26 (d, *J* = 8 Hz, 3H). ^**13**^ **C­{** ^**1**^ **H} NMR** (101 MHz, CDCl_3_) δ: 187.9, 168.9, 164.5, 152.1, 145.2, 136.9, 132.7, 130.1, 127.2, 126.6, 125.9, 121.1, 117.0, 77.9, 64.5, 22.0, 21.7, 13.9. **HRMS** (ESI) *m*/*z*: [M + H]^+^ Calcd for C_20_H_20_BrN_2_O_6_S 495.0226; found: 495.0241. **IR** 3299, 3007, 1741, 1660, 1596, 955 cm^–1^. **mp** 229–232 °C.

#### Ethyl 2-Acetamido-6-chloro-3-oxo-1-tosylindoline-2-carboxylate (**2v**)

According to general procedure B, **1**(**j**) (130 mg, 0.33 mmol) and PIFA (282 mg, 0.66 mmol, 2 equiv) were used in 12 mL of dry DCM (0.03 M). The crude residue was purified by silica gel column chromatography using EtOAc:hexane (40:60, v:v) to afford a light orange color solid, 114 mg, 77% yield. ^**1**^ **H NMR** (400 MHz, CDCl_3_) δ 7.81 (m, 1H), 7.75 (m, 2H), 7.65 (m, 1H), 7.34 (s, 1H), 7.33–7.28 (m, 2H), 7.15 (m, 1H), 4.37 (m, 1H), 4.23 (m, 1H), 2.41 (s, 3H), 1.73 (s, 3H), 1.25 (t, *J* = 8 Hz, 3H). ^**13**^ **C­{** ^**1**^ **H} NMR** (101 MHz, CDCl_3_) δ: 187.6, 168.9, 164.5, 152.2, 145.2, 143.9, 136.9, 130.1, 126.6, 125.8, 124.3, 120.7, 114.1, 78.1, 64.4, 22.0, 21.7, 13.8. **HRMS** (ESI) *m*/*z*: [M + H]^+^ Calcd for C_20_H_20_ClN_2_O_6_S 451.0731; found: 451.0755. **IR** 3316, 3016, 1781, 1748, 1656, 1071, 967 cm^–1^. **mp** 216–219 °C.

#### Ethyl 2-Acetamido-3-oxo-1-tosyl-6-(trifluoromethyl)­indoline-2-carboxylate (**2w**)

According to general procedure B, **1**(**k**) (130 mg, 0.3 mmol) and PIFA (260 mg, 0.6 mmol, 2 equiv) were used in 12 mL of dry DCM (0.03 M). The crude residue was purified by silica gel column chromatography using EtOAc:hexane (30:70, v:v) to afford an orange color solid, 103 mg, 70% yield. ^**1**^ **H NMR** (400 MHz, CDCl_3_) δ 8.06 (s, 1H), 7.83 (m, 1H), 7.78–7.72 (m, 2H), 7.45–7.41 (m, 2H), 7.31 (m, 2H), 4.38 (m, 1H), 4.25 (m, 1H), 2.41 (s, 3H), 1.74 (s, 3H), 1.26 (t, *J* = 8 Hz, 3H). ^**13**^ **C­{** ^**1**^ **H} NMR** (101 MHz, CDCl_3_) δ: 188.2, 169.2, 164.3, 151.3, 145.4, 138.7, 138.3, 138.0, 137.7, 136.7, 130.1, 126.6, 125.5, 124.6, 124.5, 121.9, 120.5, 120.5, 110.9, 110.8, 110.8, 78.0, 64.6, 21.8, 21.7, 13.8. ^**19**^ **F NMR** (376 MHz, CDCl_3_) δ −63.16. **HRMS** (ESI) *m*/*z*: [M + H]^+^ Calcd for C_21_H_20_F_3_N_2_O_6_S 485.0994; found: 485.1038. **IR** 3288, 3019, 1744, 1709, 1654, 943 cm^–1^. **mp** 203–206 °C.

### Procedures for Synthetic Transformations

#### Ethyl 2-Acetoxy-3-oxo-1-tosylindoline-2-carboxylate (**3**)

Compound **2a** (300 mg, 0.8 mmol) was dissolved in 5 mL of dry DCM (0.16 M). Triethylamine (4 mL) was added, and the mixture was stirred at room temperature for 10 min. Acetyl chloride (1.17 mL) was added, and the mixture was stirred at reflux for 2 h. The crude mixture was extracted 3 times with water and DCM and dried over sodium sulfate. The product was purified by silica gel column chromatography using EtOAc/Hexane (25:75 v:v) to afford a yellow color solid, 233 mg, 69% yield. ^**1**^ **H NMR** (400 MHz, CDCl_3_) δ 7.86–7.81 (m, 3H), 7.71 (m, 1H), 7.65 (m, 1H), 7.31 (m, 2H), 7.19 (m, 1H), 4.33 (m, 1H), 4.22 (m, 1H), 2.41 (s, 3H), 1.88 (s, 3H), 1.25 (t, *J* = 8 Hz, 3H). ^**13**^ **C­{** ^**1**^ **H} NMR** (101 MHz, CDCl_3_) δ: 188.2, 168.0, 162.5, 151.5, 145.3, 137.6, 137.0, 130.1, 126.8, 125.1, 123.9, 121.7, 113.7, 87.9, 63.6, 21.7, 20.0, 13.8. **HRMS** (ESI) *m*/*z*: [M + Na]^+^ Calcd for C_20_H_19_NNaO_7_S 440.0780; found: 440.0781. **IR** 3115, 1764, 1642, 1603, 1087, and 950 cm^–1^. **mp** 131–134 °C.

#### Ethyl 2-Chloro-3-oxo-1-tosylindoline-2-carboxylate (**4**)

Imidazole (72 mg, 1 mmol, 8 equiv) was dissolved in 3 mL of dry DCM (0.33 M). Thionyl chloride (25 μL, 0.32 mmol, 2.5 equiv) was added at 0 °C. The mixture was stirred at that temperature for 10 min, and then compound **2a** (50 mg, 0.13 mmol) dissolved in 1 mL dry DCM was added dropwise. The mixture was stirred at 0 °C for 1 h. The crude mixture was extracted 3 times with water and DCM and dried over sodium sulfate. The product was purified by silica gel column chromatography using EtOAc/Hexane (18:82 v:v) to afford an amorphous yellow solid. 21 mg, 40% yield. ^**1**^ **H NMR** (400 MHz, CDCl_3_) δ 8.07–8.01 (m, 2H), 7.78 (m, 1H), 7.67 (m, 1H), 7.60–7.56 (m, 1H), 7.36 (m, 2H), 7.24–7.19 (m, 1H), 4.44 (m, 1H), 4.35 (m, 1H), 2.43 (s, 3H), 1.34 (t, *J* = 8 Hz, 3H). ^**13**^ **C­{** ^**1**^ **H} NMR** (101 MHz, CDCl_3_) δ: 187.2, 162.4, 151.7, 145.8, 138.5, 135.6, 130.1, 128.4, 126.7, 124.5, 119.6, 114.2, 79.8, 64.7. **HRMS** (ESI) *m*/*z*: [M + Na]^+^ Calcd for C_18_H_16_ClNNaO_5_S 416.0335; found: 416.0342. **IR** 2922, 1746, 1177, 950 cm^–1^.

#### Ethyl 2-Methoxy-3-oxo-1-tosylindoline-2-carboxylate (**5**)

Compound **2a** (0.8 g, 2.1 mmol) was dissolved in a CH_3_CN:DMF (2:1) mixture (0.21 M). Cs_2_CO_3_ (2.43 g, 7.4 mmol, 3.5 equiv) was added, followed by dropwise addition of dimethylsulfate (1.6 mL, 17 mmol, 8 equiv). The mixture was stirred at room temperature overnight. Solvents were evaporated, and the product was purified by silica gel column chromatography using EtOAc/hexane (20:80 v:v) to afford a yellow color solid, 678 mg, 81% yield. ^**1**^ **H NMR** (400 MHz, CDCl_3_) δ 8.03–7.97 (m, 2H), 7.84–7.79 (m, 1H), 7.71–7.64 (m, 2H), 7.34 (m, 2H), 7.16 (m, 1H), 4.37–4.29 (m, 1H), 4.29–4.21 (m, 1H), 2.94 (s, 3H), 2.42 (s, 3H), 1.26 (t, *J* = 8 Hz, 3H). ^**13**^ **C­{** ^**1**^ **H} NMR** (101 MHz, CDCl_3_) δ: 191.2, 164.1, 153.3, 145.2, 138.6, 136.5, 130.0, 127.7, 125.4, 123.8, 120.9, 113.9, 93.0, 63.2, 52.8, 21.7, 14.0. **HRMS** (ESI) *m*/*z*: [M + H]^+^ Calcd for C_19_H_20_NO_6_S 390.1011; found: 390.1000. **IR** 3028, 1731, 1587, 1157, and 950 cm^–1^. **mp** 112–115 °C.

#### Ethyl 2-Methoxy-1-tosylspiro­[indoline-3,2′-oxirane]-2-carboxylate (**6**)

TMSOI (367 mg, 1.6 mmol, 5 equiv) was dissolved in 3 mL of dry DMSO (0.5 M). NaH (48 mg, 2 mmol) was added, and the mixture was stirred at room temperature for 5 min. Compound **5** (130 mg, 0.3 mmol) dissolved in 1 mL dry DMSO was added dropwise. The mixture was stirred at room temperature for 3 h. The crude mixture was extracted 3 times with NH_4_Cl and ethyl acetate and dried over sodium sulfate. The product was purified by silica gel column chromatography using EtOAc/Hexane (40:60 v:v) to afford an amorphous yellow solid. 50 mg, 37% yield. ^**1**^ **H NMR** (400 MHz, CDCl_3_) δ 8.06–8.01 (m, 2H), 7.34 (m, 4H), 7.06–6.98 (m, 2H), 4.37–4.29 (m, 2H), 3.53 (m, 1H), 3.46 (m, 1H), 3.03 (s, 3H), 2.41 (s, 3H), 1.31 (t, *J* = 8 Hz, 3H). ^**13**^ **C­{** ^**1**^ **H} NMR** (101 MHz, CDCl_3_) δ: 165.0, 144.8, 144.4, 136.4, 131.4, 129.8, 128.1, 123.4, 123.2, 122.6, 112.2, 97.5, 63.8, 62.7, 51.8, 51.8, 41.0, 21.7, 14.1. **HRMS** (ESI) *m*/*z*: [M + H]^+^ Calcd for C_20_H_22_NO_6_S 404.1168; found: 404.1171. **IR** 2923, 1765, 1677, 1355, 1163, 1016 cm^–1^.

#### Ethyl 2-(1*H*-Indol-3-yl)-3-oxoindoline-2-carboxylate (**7**)

Compound **5** (188 mg, 0.48 mmol) and magnesium turnings (586 mg, 24 mmol, 50 equiv) were dissolved in 12 mL of dry MeOH (0.04 M) under N_2_ atmosphere. The suspension was placed in an ultrasonicator at room temperature and sonicated for 30 min, with the flask being manually shaken every minute for the first 5 min to ensure thorough mixing. The suspension was poured over ethyl acetate with vigorous shaking to prevent a thick gel from forming. The crude mixture was extracted 3 times with ammonium chloride solution and ethyl acetate and dried over sodium sulfate. The product was purified by silica gel column chromatography using EtOAc/Hexane (9:91 v:v) to afford a purple solid, 60 mg, 61% yield. ^**1**^ **H NMR** (400 MHz, CDCl_3_) δ 7.83 (s, 1H), 7.73 (m, 1H), 7.33 (m, 1H), 7.25 (m, 1H), 7.08 (m, 1H), 4.42 (q, *J* = 8 Hz, 2H), 1.41 (t, *J* = 8 Hz, 3H). ^**13**^ **C­{** ^**1**^ **H} NMR** (101 MHz, CDCl_3_) δ: 163.5, 147.5, 135.4, 127.3, 120.2, 119.8, 117.9, 112.0, 108.2, 60.8, 14.6. **HRMS** (ESI) *m*/*z*: [M + H]^+^ Calcd for C_11_H_12_NO_3_ 206.0817; found: 206.0825. **IR** 3507, 3337, 2920, 1674, 1617, 1327, 1232, 1012, 742 cm^–1^. **mp** 93–96 °C. The deprotected indole from the previous step (30 mg, 0.15 mmol) was dissolved in 12 mL of dry DCM (1.3 mM). PIFA (62 mg, 0.15 mmol, 1 equiv) was added. The solution was stirred at room temperature for 5 min and indole (42 mg, 0.36 mmol, 2.5 equiv) was added. The solution was stirred at room temperature for 1 h and extracted 3 times with water and DCM and dried over sodium sulfate. Product was purified by silica gel column chromatography using EtOAc/Hexane (20:80 v:v) to afford 43 mg of yellow solid, 43 mg, 92% yield. ^**1**^ **H NMR** (400 MHz, CDCl_3_) δ 8.40 (s, 1H), 7.69 (m, 1H), 7.62–7.57 (m, 1H), 7.52 (m, 1H), 7.33–7.28 (m, 2H), 7.17 (m, 1H), 7.08 (m, 1H), 6.99 (m, 1H), 6.95–6.90 (m, 1H), 5.73 (s, 1H), 4.27 (q, *J* = 8 Hz, 2H), 1.23 (t, *J* = 8 Hz, 3H). ^**13**^ **C­{** ^**1**^ **H} NMR** (101 MHz, CDCl_3_) δ: 195.0, 168.5, 161.2, 137.9, 136.6, 125.5, 125.4, 123.7, 122.6, 120.3, 120.3, 119.9, 119.6, 113.6, 111.7, 111.6, 72.7, 63.1, 14.1. **HRMS** (ESI) *m*/*z*: [M + H]^+^ Calcd for C_19_H_17_N_2_O_3_ 321.1239; found: 321.1257. **IR** 3390, 3348, 2917, 1726, 1676, 1232, and 744 cm^–1^. **mp** 189–192 °C.

#### Ethyl 2-Amino-3-oxo-1-tosylindoline-2-carboxylate (**8**)

**2q** (42 mg, 0.1 mmol) was dissolved in 10 mL of ethanol (0.01 M). 2 mL of conc. HCl was added, and the mixture was stirred at 100 °C overnight. Then, pH was adjusted to 7 by adding solid NaHCO_3_ and extracted 3 times with water and ethyl acetate. The organic layer was dried over sodium sulfate, and the product was purified by silica gel column chromatography using ethyl acetate and hexane as eluents (13:87, v:v). The desired product was obtained as a white solid, 61% yield. ^**1**^ **H NMR** (400 MHz, CDCl_3_) δ 8.00–7.94 (m, 2H), 7.70 (m, 1H), 7.61 (m, 1H), 7.54 (m, 1H), 7.31 (m, 2H), 7.14 (m, 1H), 5.35 (s, 1H), 4.43 (m, 1H), 4.31 (m, 1H), 2.40 (s, 3H), 1.63 (s, 1H), 1.29 (t, *J* = 8 Hz, 3H). ^**13**^ **C­{** ^**1**^ **H} NMR** (101 MHz, CDCl_3_) δ: 191.3, 167.2, 152.4, 145.1, 138.3, 136.1, 129.9, 128.0, 125.9, 123.9, 120.3, 113.8, 87.3, 64.5, 21.7, 14.0. **HRMS** (ESI) *m*/*z*: [M + H]^+^ Calcd for C_18_H_19_N_2_O_5_S 375.1015; found: 375.1009. **IR** 3435, 3410, 2988, 1599, 1456, 1249, and 743 cm^–1^. **mp** 132–135 °C.

## Supplementary Material



## Data Availability

The data underlying this study are available in the published article and its Supporting Information.

## Notes

Synthetic procedures and characterization data for all new compounds, computational details, and optimized geometries (PDF)

## References

[1] Egami H., Shimizu R., Sodeoka M. (2013). Concise Synthesis of Oxindole Derivatives
Bearing a 3-Trifluoroethyl Group: Copper-Catalyzed Trifluoromethylation
of Acryloanilides. J. Fluorine Chem..

[2] Zhang Y., Han J., Liu Zhen-Jiang Y. H. (2015). Tert-Butoxide-Mediated
Arylation
of 1-Acetylindolin-3-Ones with Diaryliodonium Salts. Synlett.

[3] Carletti I., Banaigs B., Amade P. (2000). Matemone,
a New Bioactive Bromine-Containing
Oxindole Alkaloid from the Indian Ocean Sponge Iotrochota Purpurea. J. Nat. Prod..

[4] Wu P.-L., Hsu Y.-L., Jao C.-W. (2006). Indole Alkaloids from Cephalanceropsis
Gracilis. J. Nat. Prod..

[5] Lim K.-H., Kam T.-S. (2007). Secoleuconoxine
and Oxopericine Derivatives from Kopsia. Helv.
Chim. Acta.

[6] Bhakuni R. S., Shukla Y. N., Thakur R. S. (1991). Melochicorine, a Pseudooxindole Alkaloid
from Melochia Corchorifolia. Phytochemistry.

[7] Zhang X., Foote C. S. (1993). Dimethyldioxirane
Oxidation of Indole Derivatives.
Formation of Novel Indole-2,3-Epoxides and a Versatile Synthetic Route
to Indolinones and Indolines. J. Am. Chem. Soc..

[8] Zhou X.-Y., Chen X., Wang L.-G. (2016). Palladium-Catalyzed
Wacker-Type Oxidation
of N-Boc Indoles under Mild Conditions. Synlett.

[9] Liu R.-R., Ye S.-C., Lu C.-J., Zhuang G.-L., Gao J.-R., Jia Y.-X. (2015). Dual Catalysis for
the Redox Annulation of Nitroalkynes
with Indoles: Enantioselective Construction of Indolin-3-Ones Bearing
Quaternary Stereocenters. Angew. Chem., Int.
Ed..

[10] Coldham I., Adams H., Ashweek N. J., Barker T. A., Reeder A. T., Skilbeck M. C. (2010). Synthesis of 2-Hydroxy-3-Indolinones and 3-Hydroxy-2-Indolinones
by Anionic Cyclization, in Situ Oxidation and Rearrangement. Tetrahedron Lett..

[11] Ma R., Gu Y., Wang Y.-E., Fei R., Xiong D., Mao J. (2024). One-Pot Synthesis
of Indolin-3-Ones Mediated by LiN­(SiMe_3_)_2_/CsF. Org. Lett..

[12] Shimizu M., Imazato H., Mizota I., Zhu Y. (2019). A Facile Approach
to
2-Alkoxyindolin-3-One and Its Application to the Synthesis of N-Benzyl
Matemone. RSC Adv..

[13] Mandal T., Chakraborti G., Maiti S., Dash J. (2019). Domino Grignard Addition
and Oxidation for the One-Pot Synthesis of C2-Quaternary 2-Hydroxyindoxyls. Org. Lett..

[14] Yamashiro T., Abe T., Sawada D. (2022). Synthesis of 2-Monosubstituted
Indolin-3-Ones by Cine-Substitution
of 3-Azido-2-Methoxyindolines. Org. Chem. Front..

[15] Yarlagadda S., Ramesh B., Ravikumar
Reddy C., Srinivas L., Sridhar B., Subba Reddy B. V. (2017). Organocatalytic
Enantioselective Amination of 2-Substituted
Indolin-3-Ones: A Strategy for the Synthesis of Chiral α-Hydrazino
Esters. Org. Lett..

[16] Guo J., Lin Z.-H., Chen K.-B., Xie Y., Chan A. S. C., Weng J., Lu G. (2017). Asymmetric Amination
of 2-Substituted
Indolin-3-Ones Catalyzed by Natural Cinchona Alkaloids. Org. Chem. Front..

[17] Zhang J., Torabi Kohlbouni S., Borhan B. (2019). Cu-Catalyzed Oxidation of C2 and
C3 Alkyl-Substituted Indole via Acyl Nitroso Reagents. Org. Lett..

[18] Bott T. M., Atienza B. J., West F. G. (2014). Azide Trapping of
Metallocarbenes:
Generation of Reactive C-Acylimines and Domino Trapping with Nucleophiles. RSC Adv..

[19] Chen G., Wang Y., Zhao J., Zhang X., Fan X. (2021). Synthesis
of Hydroxysuccinimide Substituted Indolin-3-Ones via One-Pot Cascade
Reaction of o-Alkynylnitrobenzenes with Maleimides under Au­(III)–Cu­(II)
Relay/Synergetic Catalysis. J. Org. Chem..

[20] Yang F., Luo S., Wang M., Fan B., Yao B. (2024). Enantioselective Synthesis
of C2-Quaternary Indolin-3-Ones by Pt-Catalyzed Alkynylation of 2-Aryl-3H-Indol-3-One
with Alkynylsilanes. J. Org. Chem..

[21] Wen S.-S., Zhou Z.-F., Xiao J.-A., Li J., Xiang H., Yang H. (2017). Facile Oxidative Cyclization to Access
C2-Quaternary 2-Hydroxy-Indolin-3-Ones:
Synthetic Studies towards Matemone. New J. Chem..

[22] Sun W., Cui X., Qu J., Cai X., Hu J., Xiong Z., Guo S., Xu J., Chen W.-H., Wu J.-Q. (2023). Facile Access to
2-Hydroxy-2-Substituted Indole-3-Ones via a Copper-Catalyzed Oxidative
Cyclization of 2-Arylethynylanilines. Chem.
Commun..

[23] Sun Y., Fan R. (2010). Construction of 3-Oxyindoles
via Hypervalent Iodine Mediated Tandem
Cyclization–Acetoxylation of o-Acyl Anilines. Chem. Commun..

[24] Arava S., Kumar J. N., Maksymenko S., Iron M. A., Parida K. N., Fristrup P., Szpilman A. M. (2017). Enolonium Species-Umpoled Enolates. Angew. Chem., Int. Ed..

[25] Shneider O. S., Pisarevsky E., Fristrup P., Szpilman A. M. (2015). Oxidative Umpolung
α-Alkylation of Ketones. Org. Lett..

[26] Maksymenko S., Parida K. N., Pathe G. K., More A. A., Lipisa Y. B., Szpilman A. M. (2017). Transition-Metal-Free
Intermolecular α-Arylation
of Ketones via Enolonium Species. Org. Lett..

[27] More A. A., Pathe G. K., Parida K. N., Maksymenko S., Lipisa Y. B., Szpilman A. M. (2018). α-N-Heteroarylation
and α-Azidation
of Ketones via Enolonium Species. J. Org. Chem..

[28] Ghosh A., Lipisa Y. B., Fridman N., Szpilman A. M. (2023). 2-Nitro-Cyclopropyl-1-Carbonyl
Compounds from Unsaturated Carbonyl Compounds and Nitromethane via
Enolonium Species. J. Org. Chem..

[29] Maity S., Szpilman A. M. (2023). 2-Fluoroenones via
an Umpolung Morita–Baylis–Hillman
Reaction of Enones. Org. Lett..

[30] Targel T. A., Kumar J. N., Shneider O. S., Bar S., Fridman N., Maximenko S., Szpilman A. M. (2015). Oxidative Asymmetric
Umpolung Alkylation
of Evans’ β-Ketoimides Using Dialkylzinc Nucleophiles. Org. Biomol. Chem..

[31] Arava S., Santra S. K., Pathe G. K., Kapanaiah R., Szpilman A. M. (2020). Direct Umpolung Morita–Baylis–Hillman
like α-Functionalization of Enones via Enolonium Species. Angew. Chem., Int. Ed..

[32] Kapanaiah R., Oded B.-E., Strugano R., Ghosh A., Maity S., Fridman N., Szpilman A. M. (2025). Unified Strategy for the α-Functionalization
of Esters, Imides, and Ketones via Enolonium Species. Cell Rep. Phys. Sci..

[33] Kiefl G. M., Gulder T. (2020). α-Functionalization of Ketones
via a Nitrogen
Directed Oxidative Umpolung. J. Am. Chem. Soc..

[34] Merritt E. A., Olofsson B. (2011). α-Functionalization of Carbonyl Compounds Using
Hypervalent Iodine Reagents. Synthesis.

[35] Hypervalent Iodine in Organic Synthesis; Varvoglis, A. , Ed.; Academic Press: London, 1997.

[36] Bauer A., Maulide N. (2021). Recent Discoveries
on the Structure of Iodine­(III)
Reagents and Their Use in Cross-Nucleophile Coupling. Chem. Sci..

[37] Koser G. F., Rebrovic L., Wettach R. H. (1981). Functionalization of Alkenes and
Alkynes with [Hydroxy­(Tosyloxy)­Iodo]­Benzene. Bis­(Tosyloxy)­Alkanes,
Vinylaryliodonium Tosylates, and Alkynylaryliodonium Tosylates. J. Org. Chem..

[38] Hari D. P., Caramenti P., Schouwey L., Chang M., Nicolai S., Bachert D., Wright T., Orella C., Wase J. (2020). One-Pot Synthesis
of 1-[(Triisopropylsilyl)­ethynyl]-1,2-benziodoxol-3­(1H)-one (TIPS-EBX):
Process Safety Assessment and Impact of Impurities on Product Stability. Org. Process Res. Dev..

[39] Obermüller R., Tobisch H., Stockhammer L., Waser M. (2024). Thermal Stability,
OREOS+ Explosive Hazard Assessment, and Maximum Safe Storage Temperature
of Cyclic Hypervalent Iodine (λ-3-Iodane)-Based Reagents. Org. Process Res. Dev..

