# Supplementary material for: Synthesis of 2,2-Disubstituted Indolin-3-ones via Enolonium Species
Source: J Org Chem. 2025 Jul 1;90(27):9649–57. doi: 10.1021/acs.joc.5c00873 (PMC12261313; doi:10.1021/acs.joc.5c00873)

Supporting Information

Synthesis of 2,2-Disubstituted-Indolin-3-ones via Enolonium Species

Bat-El Oded, Subrata Maity, Haya Kornweitz, Alex M. Szpilman*

Department of Chemical Sciences, Ariel University, 4070000 Ariel, Israel

Table of Contents

General Information S2
Optimization of the Reaction Conditions S2
General Procedures for the Synthesis of Substrates S3
Procedures for the Synthetic Transformations S18
Mechanistic Studies S21
Computational Details S21
References S43
NMR Spectra S45

1. General Information

Unless otherwise noted, all reagents were purchased from commercial suppliers and used without further purification. All solvents were dried according to standard procedures and techniques before use. Column chromatography was performed on silica gel. ^{1}H NMR spectroscopy measurements were carried out on Bruker 400 MHz NMR spectrometers with $CDCl_3$ (δ 7.26) as an internal standard unless otherwise stated. The ^{13}C{^{1}**H**} NMR spectra were recorded on 101 MHz NMR spectrometer with $CDCl_3$ (δ 77.16) as an internal standard unless otherwise stated. HRMS spectra were acquired on an Xevo G2-XS QTof device mass spectrometer. IR spectra were obtained using a FT/IR-4700 typeA with resolution of 16 cm^{-1}. Melting point was measured on Stuart SMP50 version 1.12.

2. Optimization of the Reaction Conditions

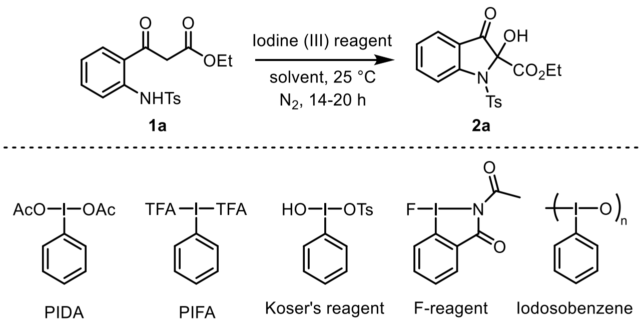

Entry	Iodine(III) Reagent (equiv.)	Solvent	% Yield
1	PIDA (2.0)	CH_2Cl_2	N.D.
2	PIFA (2.0)	CH_2Cl_2	97[b]
3	Koser's (2.0)	CH_2Cl_2	< 10
4	Ph-I=O (2.0)	CH_2Cl_2	50
5	F-reagent (2.0)	CH_2Cl_2	N.D.
6	PIFA (1.3)	CH_2Cl_2	59[b]
7	PIFA (2.5)	CH_2Cl_2	73[b]
8	Koser's (2.0)	DCE	< 30
9	PIFA (2.0)	THF	N.D.
10	PIFA (2.0)	MeOH	N.D.

Conditions: 100 mg scale, 0.025 M of DCM solvent at 25°C for 14-20 h; yields were determined by ^{1}H-NMR with trimethoxybenzene as an internal standard. [b]Isolated yields.

3. General Procedures for the Synthesis of Substrates

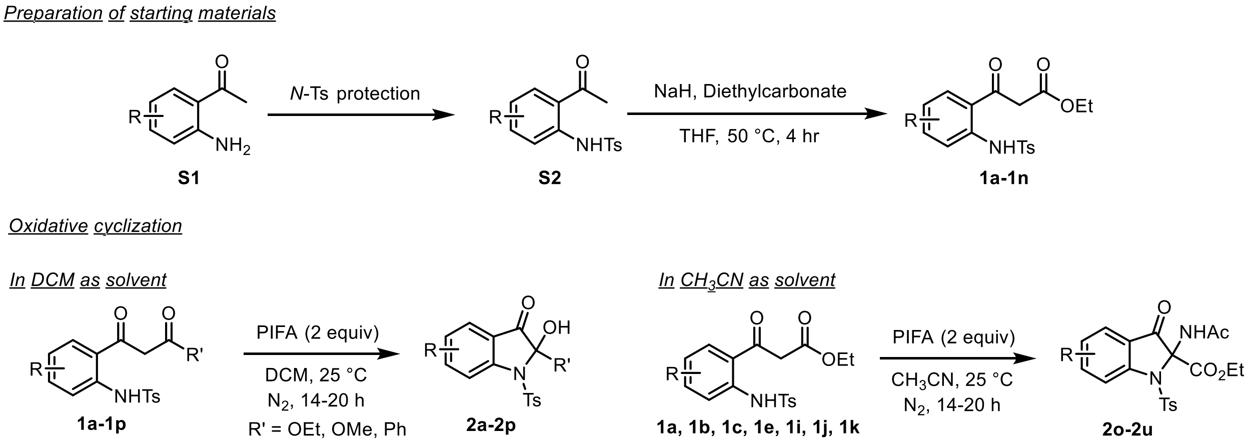

Starting materials **S1(b)**[1], **S1(c)**[2], **S1(d)**[1], **S1(e)**[3], **S1(f)**[4], **S1(g)**[5], **S1(i)**[6], **S1(j)**[7], **S1(k)**[5], **S1(l)**[8], **S1(m)**[9], **S1(n)**[10] and **1(p)**[11] are known compounds synthesized by literature procedures.

Synthesis of compounds **S2**

Compounds **S2** (**a-g**, **i-l**, **n**) were prepared according to the following procedure. Substituted aminoacetophenone was dissolved in 15 mL dry DCM. Pyridine (5 eq) was added dropwise at 0 °C. Then, *p*-toluenesulfonyl chloride (2 eq) was added portionwise. The reaction mixture was allowed to warm to room temperature and stirred overnight. The mixture was quenched with 1 M HCl solution and extracted 3 times with DCM. The organic phase was dried over sodium sulfate and purified by silica gel column chromatography using EtOAc/Hexane as eluents.

Synthesis of compound **S2(h)**: Compound **S2(d)** (2.63 g, 7.1 mmol) was dissolved in 8 mL dry DMF (0.9 M). CuCN (704 mg, 7.9 mmol, 1.1 eq) was added. The reaction was stirred at reflux overnight. The crude mixture was filtered over celite with chloroform and purified by silica gel column chromatography using EtOAc/Hexane as eluents.

Synthesis of compound **S2(m)**: **S1(m)** (389 mg, 1.6 mmol) was dissolved in 10 mL pyridine (0.16 M). TsCl (602 mg, 3.2 mmol) was added. The solution was stirred at 90 °C overnight. The mixture was extracted with 1 M HCl solution and ethyl acetate and purified by silica gel column chromatography using EtOAc/Hexane as eluents.

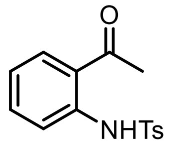

S2(a)

Following general procedure, aminoacetophenone (500 mg, 3.7 mmol), pyridine (1.8 mL, 18.5 mmol), and TsCl (1.2 g, 7.4 mmol) in dry DCM (0.25 M). The crude residue was purified by silica gel column chromatography using EtOAc:Hexane (20:80, v:v) to afford an orange color solid, 566 mg, 52% yield. **^{1}H NMR** (400 MHz, $CDCl_3$) δ 11.45 (s, 1H), 7.81 – 7.65 (m, 4H), 7.45 (m, 1H), 7.22 (m, 2H), 7.06 (m, 1H), 2.56 (s, 3H), 2.36 (s, 3H). **^{13}C{^{1}H} NMR** (101 MHz, $CDCl_3$) δ 202.4, 143.9, 140.2, 136.7, 135.0, 132.0, 129.7, 127.4, 122.6, 122.4, 119.1, 28.2, 21.6. **HRMS** (ESI) *m/z*: $[M+Na]^+$Calcd for $C_{15}H_{15}NNaO_3S$ 312.0670; found: 312.0682. **mp** 139-142 °C.

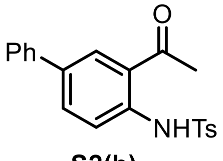

S2(b)

Following general procedure, **S1**(**b**) (315 mg, 1.5 mmol), pyridine (0.72 mL, 7.5 mmol), and TsCl (569 mg, 2 mmol) in dry DCM (0.1 M). The crude residue was purified by silica gel column chromatography using EtOAc:Hexane (20:80, v:v) to afford a yellow color solid, 283 mg, 52% yield. **^{1}H NMR** (400 MHz, $CDCl_3$) δ 11.43 (s, 1H), 7.96 (m, 1H), 7.77 (m, 3H), 7.67 (m, 1H), 7.53 – 7.33 (m, 5H), 7.24 (m, 2H), 2.62 (s, 3H), 2.37 (s, 3H). **^{13}C{^{1}H} NMR** (101 MHz, $CDCl_3$) δ 202.4, 144.0, 139.3, 136.8, 135.8, 133.6, 130.4, 129.8, 129.1, 127.9, 127.4, 126.8, 122.7, 119.6, 28.3, 21.6.

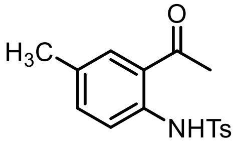

S2(c)

Following general procedure, **S1**(**c**) (922 mg, 6.2 mmol), pyridine (3 mL, 31 mmol), and TsCl (2.4 g, 12.4 mmol) in dry DCM (0.41 M). The crude residue was purified by silica gel column chromatography using EtOAc:Hexane (20:80, v:v) to afford a white color solid, 1.76 g, 94% yield. **^{1}H NMR** (400 MHz, $CDCl_3$) δ 11.21 (s, 1H), 7.72 – 7.67 (m, 2H), 7.60 (m, 1H), 7.55 (m, 1H), 7.26 (m, 1H), 7.20 m, 2H), 2.52 (s, 3H), 2.35 (s, 3H), 2.30 (s, 3H). **^{13}C{^{1}H} NMR** (101 MHz, $CDCl_3$) δ 202.4, 143.8, 137.6, 136.7, 135.7, 132.4, 132.1, 129.7, 127.4, 122.8, 119.7, 28.2, 21.6, 20.8. **HRMS** (ESI) *m/z*: $[M+Na]^+$Calcd for $C_{16}H_{17}NNaO_3S$ 326.0827; found: 326.0834. **IR** 3024, 2402, 1649, 1497, 1391, 1339, 1250, 1213, 1161, 1035, 921 cm^{-1}.

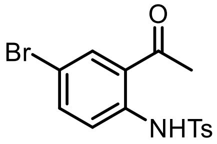

S2(d)

Following general procedure, **S1**(**d**) (500 mg, 2.34 mmol), pyridine (0.56 mL, 11.7 mmol), and TsCl (670 mg, 4.7 mmol) in dry DCM (0.16 M). The crude residue was purified by silica gel column chromatography using EtOAc:Hexane (15:85, v:v) to afford a white color solid, 649 mg, 75% yield. **^{1}H NMR** (400 MHz, $CDCl_3$) δ 11.28 (s, 1H), 7.87 (m, 1H), 7.73 – 7.69 (m, 2H), 7.61 (m, 1H), 7.53 (m, 1H), 7.23 (m, 2H), 2.54 (s, 3H), 2.37 (s, 3H). **^{13}C{^{1}H} NMR** (101 MHz, $CDCl_3$) δ 201.3, 144.3, 139.2, 137.6, 136.3, 134.4, 129.9, 127.3, 123.7, 121.0, 115.2, 28.2, 21.6. **HRMS** (ESI) *m/z*: $[M+H]^+$ Calcd for $C_{15}H_{15}BrNO_3S$ 367.9956; found: 367.9948. **IR** 1653, 1481, 1214, 1163, 1079, 908 cm^{-1}. **mp** 155 – 158 °C.

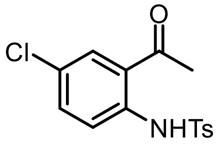

S2(e)

Following general procedure, **S1**(**e**) (500 mg, 2.9 mmol), pyridine (1.4 mL, 14.5 mmol), and TsCl (1.12 g, 5.8 mmol) in dry DCM (0.2 M). The crude residue was purified by silica gel column chromatography using EtOAc:Hexane (10:90, v:v) to afford a brown color solid, 779 mg, 82% yield. **^{1}H NMR** (400 MHz, $CDCl_3$) δ 11.25 (s, 1H), 7.73 – 7.70 (m, 2H), 7.70 – 7.65 (m, 2H), 7.40 (m, 1H), 7.23 (m, 2H), 2.54 (s, 3H), 2.37 (s, 3H). **^{13}C{^{1}H} NMR** (101 MHz, $CDCl_3$) δ 201.3, 144.2, 138.7, 136.4, 134.8, 131.5, 129.8, 128.0, 127.3, 123.5, 120.8, 28.2, 21.6. **HRMS**

(ESI) *m/z*: $[M+Na]^+$ Calcd for $C_{15}H_{14}ClNNaO_3S$ 346.0281; found: 346.0286. **IR** 3337, 3021, 1659, 1488, 1218, 1164 cm^{-1}. **mp** 145 – 148 °C.

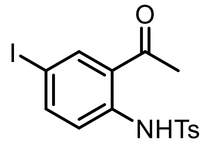

S2(f)

Following general procedure, **S1(f)** (987 mg, 3.8 mmol), pyridine (1.8 mL, 19 mmol), and TsCl (1.44 g, 7.6 mmol) in dry DCM (0.25 M). The crude residue was purified by silica gel column chromatography using EtOAc:Hexane (15:85, v:v) to afford a yellow color solid, 972 mg, 62% yield. **^{1}H NMR** (400 MHz, $CDCl_3$) δ 11.34 (s, 1H), 8.06 (m, 1H), 7.76 – 7.68 (m, 3H), 7.49 (m, 1H), 7.25 (m, 2H), 2.55 (s, 3H), 2.38 (s, 3H). **^{13}C{^{1}H} NMR** (101 MHz, $CDCl_3$) δ 201.2, 144.3, 143.4, 140.4, 139.8, 136.4, 129.9, 127.3, 124.0, 121.0, 85.1, 28.3, 21.6. **HRMS** (ESI) *m/z*: $[M+H]^+$ Calcd for $C_{15}H_{15}INO_3S$ 415.9817; found: 415.9819. **IR** 3018, 2398, 1654, 1482, 1379, 1212, 1161, 1084 cm^{-1}. **mp** 128 – 131 °C.

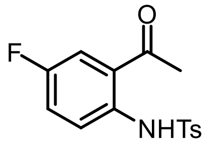

S2(g)

Following general procedure, **S1(g)** (322 mg, 2.1 mmol), pyridine (0.846 mL, 10.5 mmol), and TsCl (802 mg, 4.2 mmol) in dry DCM (0.14 M). The crude residue was purified by silica gel column chromatography using EtOAc:Hexane (20:80, v:v) to afford a white color solid, 528 mg, 82% yield. **^{1}H NMR** (400 MHz, $CDCl_3$) δ 10.99 (s, 1H), 7.69 (m, 1H), 7.66 – 7.62 (m, 2H), 7.42 (m, 1H), 7.22 – 7.15 (m, 3H), 2.48 (s, 3H), 2.35 (s, 3H). **^{13}C{^{1}H} NMR** (101 MHz, $CDCl_3$) δ 201.3, 201.3, 159.0, 156.5, 144.1, 136.2, 136.0, 135.9, 129.7, 127.2, 124.0, 123.9, 122.1, 122.0, 121.9, 121.9, 117.8, 117.6, 28.1, 21.5. **^{19}F NMR** (376 MHz, $CDCl_3$) δ -118.09. **HRMS** (ESI) *m/z*: $[M+H]^+$ Calcd for $C_{15}H_{15}FNO_3S$ 308.0757; found: 308.0763. **IR** 3439., 3050, 1651, 948cm^{-1}. **mp** 127 – 130 °C.

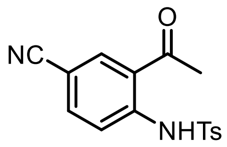

S2(h)

The compound was purified by silica gel column chromatography using EtOAc/Hexane (22:78 v:v) to afford light-yellow color solid, 358 mg, 16% yield. **^{1}H NMR** (400 MHz, $CDCl_3$) δ 11.82 (s, 1H), 8.11 (m, 1H), 7.81 – 7.75 (m, 3H), 7.69 – 7.64 (m, 1H), 7.29 (m, 2H), 2.64 (s, 3H), 2.39 (s, 3H). **^{13}C{^{1}H} NMR** (101 MHz, $CDCl_3$) δ 201.0, 144.9, 144.0, 137.6, 136.4, 136.1, 130.1, 127.4, 121.4, 118.5, 117.7, 105.6, 28.2, 21.7. **HRMS** (ESI) *m/z*: $[M+H]^+$ Calcd for $C_{16}H_{15}N_2O_3S$ 315.0804; found: 315.0800. **IR** 3323, 3023, 2229, 1751, 1063, 905 cm^{-1}. **mp** 162 – 165 °C.

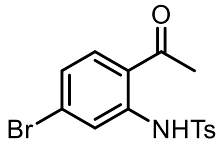

S2(i)

Following general procedure, **S1(i)** (430 mg, 2.0 mmol), pyridine (1.0 mL, 10 mmol), and TsCl (766 mg, 4 mmol) in dry DCM (0.13 M). The crude residue was purified by silica gel column chromatography using EtOAc:Hexane (7:93, v:v) to afford a yellow color solid, 440 mg, 60% yield. **^{1}H NMR** (400 MHz, $CDCl_3$) δ 11.53 (s, 1H), 7.89 (m, 1H), 7.76 (m, 2H), 7.63 (m, 1H), 7.27 (m, 2H), 7.18 (m, 1H), 2.54 (s, 3H), 2.38 (s, 3H). **^{13}C{^{1}H} NMR** (101 MHz, $CDCl_3$) δ 201.7, 144.4, 141.3, 136.4, 133.0, 130.0, 129.9, 127.4, 125.7, 121.8, 120.7, 28.2, 21.7. **HRMS** (ESI) *m/z*: $[M+H]^+$ Calcd for $C_{15}H_{15}BrNO_3S$ 367.9956; found: 367.9944. **mp** 171 – 174 °C.

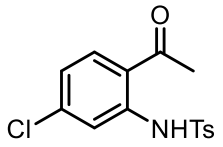

S2(j)

Following general procedure, **S1(j)** (842 mg, 5 mmol), pyridine (2 mL, 25 mmol), and TsCl (1.9 g, 10 mmol) in dry DCM (0.3 M). The crude residue was purified by silica gel column chromatography using EtOAc:Hexane (16:84, v:v) to afford a light pink color solid, 819 mg, 51% yield. **^{1}H NMR** (400 MHz, $CDCl_3$) δ 11.60 (s, 1H), 7.77 (m, 2H), 7.73 (m, 2H), 7.29 – 7.25 (m, 2H), 7.02

(m, 1H), 2.57 (s, 3H), 2.39 (s, 3H). **^{13}C{^{1}H} NMR** (101 MHz, $CDCl_3$) δ 201.5, 144.4, 141.4, 141.3, 136.4, 133.1, 129.9, 127.4, 122.7, 120.3, 118.7, 28.2, 21.6. **HRMS** (ESI) *m/z*: $[M+H]^+$ Calcd for $C_{15}H_{15}ClNO_3S$ 324.0461; found: 324.0464. **IR** 3285, 3016, 1753, 934 cm^{-1}.

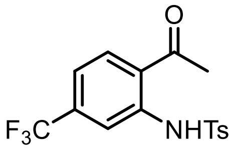

S2(k)

Following general procedure, **S1(k)** (669 mg, 3.3 mmol), pyridine (1.33 mL, 16.5 mmol), and TsCl (1.26 g, 6.6 mmol) in dry DCM (0.2 M). The crude residue was purified by silica gel column chromatography using EtOAc:Hexane (16:84, v:v) to afford a white color solid, 859 mg, 73% yield. **^{1}H NMR** (400 MHz, $CDCl_3$) δ 11.45 (s, 1H), 8.01 – 7.89 (m, 2H), 7.74 (m, 2H), 7.31 – 7.22 (m, 3H), 2.61 (s, 3H), 2.37 (s, 3H). **^{13}C{^{1}H} NMR** (101 MHz, $CDCl_3$) δ 201.9, 144.6, 140.5, 136.4, 136.1, 136.1, 135.8, 135.5, 132.6, 129.9, 127.4, 124.3, 124.0, 121.6, 119.0, 118.9, 118.9, 118.9, 115.9, 115.8, 115.8, 115.7, 28.4, 21.6. **^{19}F NMR** (376 MHz, $CDCl_3$) δ -63.81. **HRMS** (ESI) *m/z*: $[M+H]^+$ Calcd for $C_{16}H_{15}F_3NO_3S$ 358.0725; found: 358.0730. **IR** 3115, 3030, 1654, 1085, 932 cm^{-1}. **mp** 152 – 155 °C.

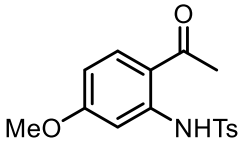

S2(l)

Following general procedure, **S1(l)** (280 mg, 1.6 mmol), pyridine (0.644 mL, 8 mmol), and TsCl (648 mg, 3.2 mmol) in dry DCM (0.12 M). The crude residue was purified by silica gel column chromatography using EtOAc:Hexane (20:80, v:v) to afford a yellow color solid color solid, 155 mg, 29% yield. **^{1}H NMR** (400 MHz, $CDCl_3$) δ 11.98 (s, 1H), 7.81 (m, 3H), 7.35 – 7.26 (m, 3H), 6.61 (m, 1H), 3.89 (s, 3H), 2.59 (s, 3H), 2.44 (s, 3H). **^{13}C{^{1}H} NMR** (101 MHz, $CDCl_3$) δ 200.8, 164.5, 144.0, 142.9, 136.7, 134.1, 129.8, 127.4, 115.6, 109.1, 102.8, 55.7, 27.9, 21.6. **HRMS** (ESI) *m/z*: $[M+H]^+$ Calcd for $C_{16}H_{18}NO_4S$ 320.0956; found: 320.0949. **IR** 3307, 3023, 1741, 1146, 957 cm^{-1}. **mp** 145 – 148 °C.

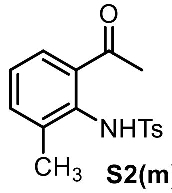

S2(m)

The compound was purified by silica gel column chromatography (70:30 ethyl acetate/hexane) to afford a white color solid, 125 mg, 43% yield. **^{1}H NMR** (400 MHz, $CDCl_3$) δ 8.98 (s, 1H), 7.43 (m, 1H), 7.39 – 7.35 (m, 1H), 7.35 – 7.29 (m, 2H), 7.19 (m, 1H), 7.16 – 7.12 (m, 2H), 2.56 (s, 3H), 2.36 (s, 3H), 1.92 (s, 3H). **^{13}C{^{1}H} NMR** (101 MHz, $CDCl_3$) δ 201.8, 143.7, 139.7, 136.4, 134.9, 134.8, 133.0, 129.3, 128.0, 127.5, 126.5, 27.7, 21.5, 19.5. **HRMS** (ESI) *m/z*: $[M+H]^+$ Calcd for $C_{16}H_{18}NO_3S$ 304.1007; found: 304.1015. **IR** 1656, 1275, 1158, 787 cm^{-1}. **mp** 142 – 145 °C.

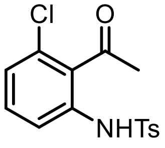

S2(n)

Following general procedure, **S1(n)** (509 mg, 3.0 mmol), pyridine (1.2 mL, 15 mmol), and TsCl (1.14 mg, 6 mmol) in dry DCM (0.2 M). The crude residue was purified by silica gel column chromatography using EtOAc:Hexane (16:84, v:v) to afford an orange color solid, 889 mg, 91% yield. **^{1}H NMR** (400 MHz, $CDCl_3$) δ 8.25 (s, 1H), 7.60 – 7.54 (m, 3H), 7.32 (m, 1H), 7.26 – 7.22 (m, 2H), 7.19 (m, 1H), 2.38 (s, 3H), 2.14 (s, 3H). **^{13}C{^{1}H} NMR** (101 MHz, $CDCl_3$) δ 202.8, 144.3, 136.2, 136.0, 132.0, 131.9, 131.5, 129.9, 127.3, 123.0, 31.6, 21.6. **HRMS** (ESI) *m/z*: $[M+H]^+$ Calcd for $C_{15}H_{15}ClNO_3S$ 324.0461; found: 324.0466. **IR** 3175, 3038, 1698, 961 cm^{-1}. **mp** 107 – 110 °C.

Synthesis of compounds **1a-1o**

Compound **S2** was dissolved in 20 mL dry THF. NaH (5 eq) was added portionwise at 0 °C and the solution stirred at room temperature for 10 minutes. Then, diethylcarbonate (2 eq) was added dropwise and the solution was stirred for 4 hours at 50 °C in an oil bath. The reaction mixture was quenched with 10 ml 1M HCl solution and extracted 3 times with ethyl acetate. The organic phase was dried over sodium sulfate and the crude was purified by silica gel column chromatography using EtOAc/Hexane as eluents.

Synthesis of compound **1o**: Compound **1a** (300 mg, 0.83 mmol) was dissolved in 8 mL methanol (0.1 M). Three drops of conc. HCl were added. The mixture was stirred at reflux in an oil bath overnight. Methanol was evaporated, and the mixture was extracted 3 times with DCM (3X15 mL) and 10 mL water. The organic layer was dried over sodium sulfate and concentrated under vacuum. The crude residue was purified by silica gel column chromatography using EtOAc:Hexane 35:65 to afford pink color solid, 225 mg, 78% yield. **^{1}H NMR** (400 MHz, $CDCl_3$) δ 11.18 (s, 1H), 7.78 – 7.68 (m, 4H), 7.50 – 7.45 (m, 1H), 7.27 – 7.20 (m, 2H), 7.05 (m, 1H), 3.95 (s, 2H), 3.74 (s, 3H), 2.36 (s, 3H). **^{13}C{^{1}H} NMR** (101 MHz, $CDCl_3$) δ 196.4, 167.4, 144.1, 140.7, 136.5, 135.7, 131.6, 129.8, 127.4, 122.7, 121.2, 119.0, 52.7, 46.8, 21.6. **HRMS** (ESI) *m/z*: $[M+Na]^+$ Calcd for $C_{17}H_{17}NNaO_5S$ 370.0725; found: 370.0721. **IR** 3110, 2954, 1649, 1488, 1291, 978 cm^{-1}.

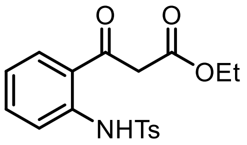

1(a)

Following general procedure, Ts-protected aminoacetophenone (200 mg, 0.7 mmol), NaH (84 mg, 3.5 mmol), and diethyl carbonate (0.16 mL, 1.4 mmol) in dry THF (0.04 M). The crude residue was purified by silica gel column chromatography using EtOAc:Hexane (30:70, v:v) to afford a brown color solid, 200 mg, 80% yield. **^{1}H NMR** (400 MHz, $CDCl_3$) δ 11.20 (s, 1H), 7.75 (m, 2H), 7.70 (m, 2H), 7.50 – 7.43 (m, 1H), 7.23 (m, 2H), 7.08 – 7.03 (m, 1H), 4.20 (q, *J* = 8 Hz, 2H), 3.93 (s, 2H), 2.36 (s, 3H), 1.24 (t, *J* = 8 Hz, 3H). **^{13}C{^{1}H} NMR** (101 MHz, $CDCl_3$) δ 196.6, 167.0, 144.1, 140.7, 136.5, 135.6, 131.6, 129.8, 127.4, 122.7, 121.3, 119.0, 61.8, 47.1, 21.6, 14.1. **HRMS** (ESI) *m/z*: $[M+H]^+$ Calcd for $C_{18}H_{20}NO_5S$ 362.1062; found: 362.1057. **IR** 1746, 1646, 1576, 1492, 1455, 1216, 1155, 1035, 996, 918 cm^{-1}. **mp** 90 – 93 °C.

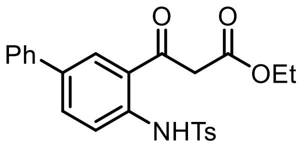

1(b)

Following general procedure, Ts-protected aminoacetophenone (417 mg, 1.1 mmol), NaH (132 mg, 5.5 mmol), and diethyl carbonate (0.28 mL, 2.2 mmol) in dry THF (0.06 M). The crude residue was purified by silica gel column chromatography using EtOAc:Hexane (30:70, v:v) to afford a yellow color solid, 491 mg, 98% yield. **^{1}H NMR** (400 MHz, $CDCl_3$) δ 11.17 (s, 1H), 7.92 (m, 1H), 7.81 – 7.77 (m, 3H), 7.70 (m, 1H), 7.50 – 7.35 (m, 5H), 7.27 (s, 1H), 7.25 (m, 1H), 4.21 (q, *J* = 8 Hz, 2H), 3.99 (s, 2H), 2.38 (s, 3H), 1.24 (t, *J* = 8 Hz, 3H). **^{13}C{^{1}H}**

NMR (101 MHz, $CDCl_3$) δ 196.6, 167.0, 144.2, 139.8, 136.6, 135.8, 134.2, 130.1, 129.9, 129.2, 128.0, 127.5, 126.8, 121.6, 119.5, 61.9, 47.4, 21.7, 14.2. **HRMS** (ESI) *m/z*: $[M+H]^+$ Calcd for $C_{24}H_{24}NO_5S$ 438.1375; found: 438.1375. **IR** 1744, 1653, 1481, 1333, 1292, 1218, 1159, 1088, 1023, 915 cm^{-1}. **mp** 113 – 116 °C.

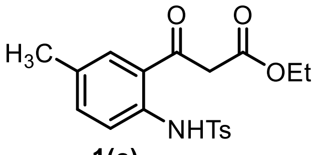

1(c)

Following general procedure, Ts-protected aminoacetophenone (1.75 g, 5.8 mmol), NaH (696 mg, 29 mmol), and diethyl carbonate (1.05 mL, 11.6 mmol) in dry THF (0.3 M). The crude residue was purified by silica gel column chromatography using EtOAc:Hexane (30:70, v:v) to afford a light brown color solid, 1.56 g, 72% yield. **^{1}H NMR** (400 MHz, $CDCl_3$) δ 10.98 (s, 1H), 7.74 – 7.70 (m, 2H), 7.62 (m, 1H), 7.47 (m, 1H), 7.29 (m, 1H), 7.22 (m, 2H), 4.20 (q, *J* = 8 Hz, 2H), 3.90 (s, 2H), 2.36 (s, 3H), 2.29 (s, 3H), 1.25 (t, *J* = 8 Hz, 3H). **^{13}C{^{1}H} NMR** (101 MHz, $CDCl_3$) δ 196.5, 167.1, 143.9, 138.2, 136.6, 136.4, 132.5, 131.6, 129.8, 127.4, 121.6, 119.5, 61.8, 47.1, 21.6, 20.8, 14.2. **HRMS** (ESI) *m/z*: $[M+H]^+$ Calcd for $C_{19}H_{22}NO_5S$ 376.1219; found: 376.1212. **IR** 1734, 1648, 1496, 1412, 1331, 1155, 1033 cm^{-1}. **mp** 124 – 127 °C.

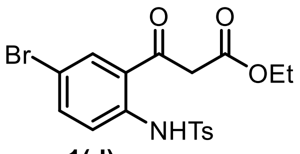

1(d)

Following general procedure, Ts-protected aminoacetophenone (400 mg, 1.08 mmol), NaH (129 mg, 5.4 mmol), and diethyl carbonate (0.189 mL, 2.16 mmol) in dry THF (0.05 M). The crude residue was purified by silica gel column chromatography using EtOAc:Hexane (25:75, v:v) to afford a brown color solid, 438 mg, 92% yield. **^{1}H NMR** (400 MHz, $CDCl_3$) δ 11.04 (s, 1H), 7.80 (m, 1H), 7.73 (m, 2H), 7.63 (m, 1H), 7.56 (m, 1H), 7.24 (m, 2H), 4.21 (q, *J* = 8 Hz, 2H), 3.90 (s, 2H), 2.38 (s, 3H), 1.25 (d, *J* = 8 Hz, 3H). **^{13}C{^{1}H} NMR** (101 MHz, $CDCl_3$) δ 195.6, 166.5, 144.5, 139.7, 138.3, 136.2, 134.1, 130.0, 127.4, 122.7, 120.8, 115.2, 62.0, 47.1, 21.7, 14.1. **HRMS** (ESI) *m/z*: $[M+H]^+$ Calcd for $C_{18}H_{19}BrNO_5S$ 440.0167; found: 440.0163. **IR** 1739, 1664, 1485, 1388, 1318, 1214, 1163, 1088, 1028 cm^{-1}. **mp** 92 – 95 °C.

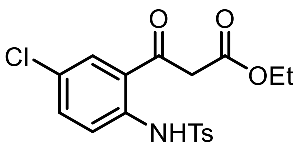

1(e)

Following general procedure, Ts-protected aminoacetophenone (779 mg, 2.4 mmol), NaH (288 mg, 12 mmol), and diethyl carbonate (0.44 mL, 4.8 mmol) in dry THF (0.12 M). The crude residue was purified by silica gel column chromatography using EtOAc:Hexane (15:85, v:v) to afford an orange color solid, 500 mg, 53% yield. **^{1}H NMR** (400 MHz, $CDCl_3$) δ 11.01 (s, 1H), 7.75 – 7.64 (m, 4H), 7.43 (m, 1H), 7.24 (m, 2H), 4.21 (q, *J* = 8 Hz, 2H), 3.89 (s, 2H), 2.38 (s, 3H), 1.26 (t, *J* = 8 Hz, 3H). **^{13}C{^{1}H} NMR** (101 MHz, $CDCl_3$) δ 195.7, 166.5, 144.4, 139.2, 136.2, 135.4, 131.1, 129.9, 128.0, 127.4, 122.4, 120.7, 62.0, 47.1, 21.7, 14.1. **HRMS** (ESI) *m/z*: $[M+H]^+$ Calcd for $C_{18}H_{19}ClNO_5S$ 396.0673; found: 396.0669. **IR** 3105, 1736, 1651, 1592, 1475, 1406, 1327, 1158, 1091 cm^{-1}. **mp** 93 – 96 °C.

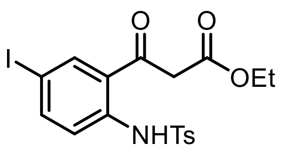

1(f)

Following general procedure, Ts-protected aminoacetophenone (400 mg, 0.96 mmol), NaH (115 mg, 4.8 mmol), and diethyl carbonate (0.175 mL, 1.92 mmol) in dry THF (0.05 M). The crude residue was purified by silica gel column chromatography using EtOAc:Hexane (15:85, v:v) to afford a red color oil, 436 mg, 93% yield. **^{1}H NMR** (400 MHz, $CDCl_3$) δ 11.07 (s, 1H), 7.97 (m, 1H), 7.77 – 7.68 (m, 3H), 7.49 (m, 1H), 7.25 (m, 2H), 4.21 (q, *J* = 8 Hz, 2H), 3.90 (s, 2H), 2.38 (s, 3H), 1.26 (t, *J* = 8 Hz, 3H). **^{13}C{^{1}H} NMR** (101 MHz, $CDCl_3$) δ 195.5, 166.5,

144.5, 144.0, 140.3, 140.0, 136.2, 129.9, 127.4, 123.0, 120.8, 84.9, 62.0, 47.1, 21.7, 14.2. **HRMS** (ESI) *m/z*: $[M+Na]^+$ Calcd for $C_{18}H_{18}INNaO_5S$ 509.9848; found: 509.9858. **IR** 1737, 1653, 1481, 1216, 1161, 1086, 1031, 915 cm^{-1}.

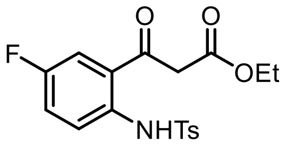

1(g)

Following general procedure, Ts-protected aminoacetophenone (528 mg, 1.7 mmol), NaH (206 mg, 8.5 mmol), and diethyl carbonate (0.416 mL, 3.4 mmol) in dry THF (0.09 M). The crude residue was purified by silica gel column chromatography using EtOAc:Hexane (18:82, v:v) to afford an orange color solid, 358 mg, 55% yield. **^{1}H NMR** (400 MHz, $CDCl_3$) δ 10.81 (s, 1H), 7.75 (m, 1H), 7.72 – 7.68 (m, 2H), 7.40 (m, 1H), 7.27 – 7.22 (m, 3H), 4.22 (q, *J* = 8 Hz, 2H), 3.87 (s, 2H), 2.39 (s, 3H), 1.27 (t, *J* = 78 Hz, 3H). **^{13}C{^{1}H} NMR** (101 MHz, $CDCl_3$) δ 195.6, 195.6, 166.5, 158.9, 156.4, 144.3, 136.7, 136.6, 136.1, 129.8, 127.3, 122.9, 122.7, 121.9, 121.8, 117.5, 117.3, 61.9, 47.1, 21.6, 14.1. **^{19}F NMR** (376 MHz, $CDCl_3$) δ -117.88. **HRMS** (ESI) *m/z*: $[M+H]^+$ Calcd for $C_{18}H_{19}FNO_5S$ 380.0968; found: 380.0972. **IR** 3439, 3107, 1750, 1603, 1150, 893 cm^{-1}. **mp** 83 – 86 °C.

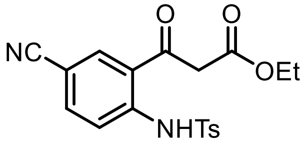

1(h)

Following general procedure, Ts-protected aminoacetophenone (350 mg, 1.1 mmol), NaH (134 mg, 5.5 mmol), and diethyl carbonate (0.266 mL, 2.2 mmol) in dry THF (0.06 M). The crude residue was purified by silica gel column chromatography using EtOAc:Hexane (17:83, v:v) to afford an orange color oil, 209 mg, 49% yield. **^{1}H NMR** (400 MHz, $CDCl_3$) δ 11.52 (s, 1H), 8.04 (m, 1H), 7.82 – 7.76 (m, 3H), 7.68 (m, 1H), 7.29 (m, 2H), 4.22 (q, *J* = 8 Hz, 2H), 3.98 (s, 2H), 2.39 (s, 3H), 1.27 (t, *J* = 8 Hz, 3H). **^{13}C{^{1}H} NMR** (101 MHz, $CDCl_3$) δ 195.6, 166.3, 145.1, 144.3, 138.0, 136.2, 135.9, 130.2, 127.5, 120.5, 118.5, 117.5, 105.7, 62.2, 46.9, 21.7, 14.1. **HRMS** (ESI) *m/z*: $[M+H]^+$ Calcd for $C_{19}H_{19}N_2O_5S$ 387.1015; found: 387.1007. **IR** 3314, 3115, 2231, 1739, 1654, 1159, 900 cm^{-1}. **mp** 97 – 100 °C.

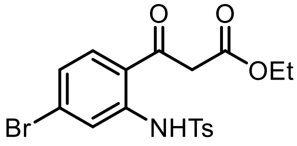

1(i)

Following general procedure, Ts-protected aminoacetophenone (438 mg, 1.1 mmol), NaH (142 mg, 5.5 mmol), and diethyl carbonate (0.266 mL, 2.2 mmol) in dry THF (0.06 M). The crude residue was purified by silica gel column chromatography using EtOAc:Hexane (20:80, v:v) to afford an orange color solid, 385 mg, 74% yield. **^{1}H NMR** (400 MHz, $CDCl_3$) δ 11.26 (s, 1H), 7.90 (m, 1H), 7.77 (m, 2H), 7.56 (m, 1H), 7.28 (m, 2H), 7.18 (m, 1H), 4.19 (q, *J* = 8 Hz, 2H), 3.90 (s, 2H), 2.38 (s, 3H), 1.25 (t, *J* = 8 Hz, 3H). **^{13}C{^{1}H} NMR** (101 MHz, $CDCl_3$) δ 195.9, 166.7, 144.5, 141.7, 136.2, 132.7, 130.8, 130.0, 127.4, 125.8, 121.7, 119.7, 61.9, 47.1, 21.7, 14.1.

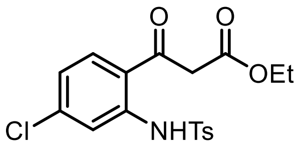

1(j)

Following general procedure, Ts-protected aminoacetophenone (813 mg, 2.5 mmol), NaH (301 mg, 12.5 mmol), and diethyl carbonate (0.610 mL, 5 mmol) in dry THF (0.13 M). The crude residue was purified by silica gel column chromatography using EtOAc:Hexane (10:90, v:v) to afford an orange color solid, 353 mg, 36% yield. **^{1}H NMR** (400 MHz, $CDCl_3$) δ 11.31 (s, 1H), 7.80 – 7.75 (m, 2H), 7.73 (m, 1H), 7.64 (m, 1H), 7.29 – 7.26 (m, 2H), 7.01 (m, 1H), 4.19 (q, *J* = 8 Hz, 2H), 3.91 (s, 2H), 2.39 (s, 3H), 1.24 (t, *J* = 8 Hz, 3H). **^{13}C{^{1}H} NMR** (101 MHz, $CDCl_3$) δ 195.7, 166.7, 144.5, 142.1, 141.9, 136.2, 132.8, 130.0, 127.4, 122.8, 119.3, 118.6,

61.9, 47.2, 21.7, 14.1. **HRMS** (ESI) *m/z*: $[M+Na]^+$ Calcd for $C_{18}H_{18}ClNNaO_5S$ 418.0492; found: 418.0500. **IR** 3117, 2983, 1741, 1656, 1140, 910 cm^{-1}. **mp** 89 – 92 °C.

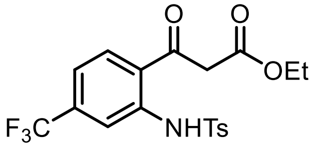

1(k)

Following general procedure, Ts-protected aminoacetophenone (859 mg, 2.4 mmol), NaH (288 mg, 12 mmol), and diethyl carbonate (0.582 mL, 4.8 mmol) in dry THF (0.12 M). The crude residue was purified by silica gel column chromatography using EtOAc:Hexane (10:90, v:v) to afford an orange color solid, 618 mg, 60% yield. **^{1}H NMR** (400 MHz, $CDCl_3$) δ 11.19 (s, 1H), 8.00 (m, 1H), 7.84 (m, 1H), 7.79 – 7.75 (m, 2H), 7.30 – 7.25 (m, 3H), 4.20 (q, *J* = 8 Hz, 2H), 3.96 (s, 2H), 2.38 (s, 3H), 1.25 (t, *J* = 8 Hz, 3H). **$^{13}C\{^1H\}$ NMR** (101 MHz, $CDCl_3$) δ 196.2, 166.5, 144.7, 141.1, 136.7, 136.4, 136.0, 132.3, 130.0, 127.5, 123.0, 121.5, 118.9, 118.9, 115.8, 115.7, 62.1, 47.3, 21.7, 14.1. **^{19}F NMR** (376 MHz, $CDCl_3$) δ -63.93. **HRMS** (ESI) *m/z*: $[M+H]^+$ Calcd for $C_{19}H_{19}F_3NO_5S$ 430.0936; found: 430.0944. **IR** 3288, 3109, 1748, 1658, 1069, 939 cm^{-1}. **mp** 82 – 85 °C.

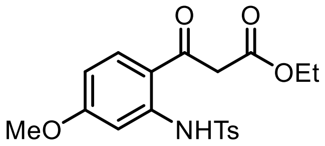

1(l)

Following general procedure, Ts-protected aminoacetophenone (147 mg, 0.46 mmol), NaH (55 mg, 2.3 mmol), and diethyl carbonate (0.11 mL, 0.92 mmol) in dry THF (0.02 M). The crude residue was purified by silica gel column chromatography using EtOAc:Hexane (25:75, v:v) to afford an amorphous orange color solid, 105 mg, 58% yield. **^{1}H NMR** (400 MHz, $CDCl_3$) δ 11.60 (s, 1H), 7.75 – 7.70 (m, 2H), 7.62 (m, 1H), 7.24 – 7.19 (m, 2H), 7.13 (m, 1H), 6.51 (m, 1H), 4.16 (q, *J* = 8 Hz, 2H), 3.86 (s, 2H), 3.78 (s, 3H), 2.33 (s, 3H), 1.21 (t, *J* = 8 Hz, 3H). **$^{13}C\{^1H\}$ NMR** (101 MHz, $CDCl_3$) δ 194.8, 167.2, 164.9, 144.1, 143.2, 136.3, 133.9, 129.7, 127.3, 114.5, 109.1, 102.7, 61.5, 55.6, 46.7, 21.5, 14.0. **HRMS** (ESI) *m/z*: $[M+H]^+$ Calcd for $C_{19}H_{22}NO_6S$ 392.1168; found: 392.1172. **IR** 3258, 1738, 1634, 1158, 891 cm^{-1}.

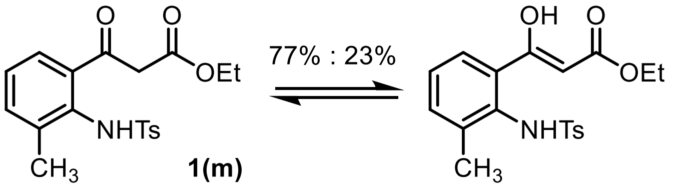

1(m)

Following general procedure, Ts-protected aminoacetophenone (475 mg, 1.56 mmol), NaH (187 mg, 7.8 mmol), and diethyl carbonate (0.380 mL, 3.12 mmol) in dry THF (0.08 M). The crude residue was purified by silica gel column chromatography using EtOAc:Hexane (15:85, v:v) to afford a pink color oil, 460 mg, 78% yield. **^{1}H NMR** (400 MHz, $CDCl_3$) δ 12.52 (s, 0.3H), 8.74 (s, 1H), 7.61 (s, 0.25H), 7.49 (m, 1H), 7.42 (m, 1H), 7.39 – 7.34 (m, 3H), 7.24 (s, 0.5H), 7.19 (m, 2H), 7.08 – 7.05 (m, 0.59H), 7.00 (m, 0.3H), 4.59 (s, 0.3H), 4.23 – 4.17 (m, 0.89H), 4.19 – 4.13 (m, 2H), 3.27 (s, 2H), 2.63 (s, 0.95H), 2.55 (s, 3H), 2.40 (s, 3H), 2.36 (s, 0.98H), 1.34 (t, *J* = 8 Hz, 1H), 1.25 (t, *J* = 8 Hz, 3H). **$^{13}C\{^1H\}$ NMR** (101 MHz, $CDCl_3$) δ 195.7, 172.7, 171.4, 166.6, 143.9, 143.8, 140.6, 140.1, 137.1, 135.5, 135.3, 135.1, 134.3, 133.0, 132.2, 131.8, 129.6, 129.5, 127.9, 127.8, 127.7, 127.4, 126.7, 126.4, 91.1, 61.6, 60.7, 46.7, 21.6, 21.5, 19.7, 14.3, 14.2. **HRMS** (ESI) *m/z*: $[M+H]^+$ Calcd for $C_{19}H_{22}NO_5S$ 376.1219; found: 376.1211. **IR** 3239, 2994, 1724, 1687, 1162, 895 cm^{-1}. **mp** 109 – 112 °C.

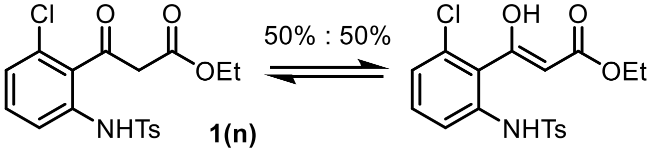

1(n)

Following general procedure, Ts-protected aminoacetophenone (889 mg, 2.7 mmol), NaH (329 mg, 13.5 mmol), and diethyl carbonate (0.664 mL, 5.4 mmol) in dry THF (0.14 M). The crude residue was purified by silica gel column chromatography using EtOAc:Hexane (15:85, v:v) to afford an amorphous blue color solid, 138 mg, 13% yield. **^{1}H NMR** (400 MHz, $CDCl_3$) (Keto-Enol 1:1) δ 12.49 (s, 1H), 8.26 (s, 1H), 7.70 – 7.66 (m, 2H), 7.60 (m, 2H), 7.54 – 7.50 (m, 2H), 7.38 (s, 1H), 7.31 (m, 2H), 7.25 (m, 2H), 7.22 (m, 1H), 7.19 – 7.14 (m, 2H), 7.13 (m, 1H), 4.75 (s, 1H), 4.27 (q, *J* = 8 Hz, 2H), 4.21 (q, *J* = 8 Hz, 2H), 3.73 (s, 2H), 2.39 (s, 3H), 2.38 (s, 3H), 1.37 (t, *J* = 8 Hz, 3H), 1.28 (t, *J* = 8, 3H). **^{13}C{^{1}H} NMR** (101 MHz, $CDCl_3$) δ 197.0, 172.2, 167.3, 165.9, 144.3, 136.6, 136.5, 136.2, 136.0, 132.7, 132.3, 131.5, 131.2, 129.9, 129.8, 127.6, 127.2, 126.5, 126.2, 122.5, 120.9, 96.6, 62.1, 61.1, 49.6, 21.7, 21.7, 14.3, 14.2. **HRMS** (ESI) *m/z*: $[M+Na]^+$ Calcd for $C_{18}H_{18}ClNNaO_5S$ 418.0492; found: 418.0502. **IR** 3281, 2917, 1647, 1638, 1155, 943 cm^{-1}.

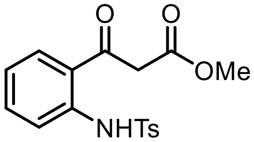

1(o)

The crude residue was purified by silica gel column chromatography using EtOAc:Hexane 35:65 to afford pink color solid, 225 mg, 78% yield. **^{1}H NMR** (400 MHz, $CDCl_3$) δ 11.18 (s, 1H), 7.78 – 7.68 (m, 4H), 7.50 – 7.45 (m, 1H), 7.27 – 7.20 (m, 2H), 7.05 (m, 1H), 3.95 (s, 2H), 3.74 (s, 3H), 2.36 (s, 3H). **^{13}C{^{1}H} NMR** (101 MHz, $CDCl_3$) δ 196.4, 167.4, 144.1, 140.7, 136.5, 135.7, 131.6, 129.8, 127.4, 122.7, 121.2, 119.0, 52.7, 46.8, 21.6. **HRMS** (ESI) *m/z*: $[M+Na]^+$ Calcd for $C_{17}H_{17}NNaO_5S$ 370.0725; found: 370.0721. **IR** 3110, 2954, 1649, 1488, 1291, 978 cm^{-1}. **mp** 96 – 99 °C.

Synthesis of compounds **2a-2p** in DCM as solvent

Compound **1a-1n** was dissolved in 12 mL dry DCM (0.02 M). PIFA (2 eq) was added portionwise at room temperature. The reaction was allowed to stir at room temperature overnight. The mixture was extracted 3 times with 10 mL water and DCM (3X30 mL) and dried over sodium sulfate. The product was purified by silica gel column chromatography using EtOAc/Hexane as eluents.

Large scale synthesis of compound **2a**

Compound **1(a)** (2g, 5.5 mmol) was dissolved in 50 mL dry DCM (0.1 M). PIFA (4.76 g, 11 mmol) was added portionwise at room temperature. The reaction was allowed to stir at room temperature overnight. The mixture was extracted 3 times with 15 mL water and DCM (3X30 mL) and dried over sodium sulfate. The product was purified by silica gel column chromatography using EtOAc/Hexane (20:80, v:v) to afford a brown color solid, 1.3 g, 63% yield.

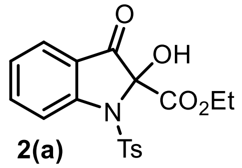

2(a)

Following general procedure, **1**(**a**) (100 mg, 0.27 mmol) and PIFA (238 mg, 0.54 mmol) in dry DCM (0.02 M). The crude residue was purified by silica gel column chromatography using EtOAc:Hexane (20:80, v:v) to afford a brown color solid, 101 mg, 97% yield. **^{1}H NMR** (400 MHz, $CDCl_3$) δ 7.98 (m, 2H), 7.70 (m, 1H), 7.63 – 7.51 (m, 2H), 7.31 (m, 2H), 7.17 – 7.11 (bs, 1H), 5.33 (s, 1H), 4.43 (m, 1H), 4.31 (m, 1H), 2.40 (s, 3H), 1.29 (t, *J* = 8 Hz, 3H). **^{13}C{^{1}H} NMR** (101 MHz, $CDCl_3$) δ 191.2, 167.3, 152.4, 145.1, 138.3, 136.1, 129.9, 128.0, 125.9, 123.9, 120.3, 87.3, 64.5, 21.7, 14.0. **HRMS** (ESI) *m/z*: $[M+Na]^+$ Calcd for $C_{18}H_{17}NNaO_6S$ 398.0674; found: 398.0668. **mp** 108 – 111 °C.

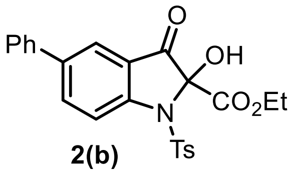

2(b)

Following general procedure, **1**(**b**) (100 mg, 0.23 mmol) and PIFA (197 mg, 0.46 mmol) in dry DCM (0.02 M). The crude residue was purified by silica gel column chromatography using EtOAc:Hexane (20:80, v:v) to afford an amorphous yellow color solid, 82 mg, 80% yield. **^{1}H NMR** (400 MHz, $CDCl_3$) δ 8.01 (m, 2H), 7.90 (m, 1H), 7.85 (m, 1H), 7.64 (m, 1H), 7.53 – 7.49 (m, 2H), 7.46 – 7.41 (m, 2H), 7.39 – 7.31 (m, 3H), 5.42 (s, 1H), 4.45 (m, 1H), 4.33 (m, 1H), 2.41 (s, 3H), 1.31 (t, *J* = 8 Hz, 3H). **^{13}C{^{1}H} NMR** (101 MHz, $CDCl_3$) δ 191.3, 167.2, 151.5, 145.2, 138.8, 137.5, 137.3, 136.1, 130.0, 129.1, 128.0, 126.8, 123.8, 120.8, 114.1, 87.8, 64.6, 21.7, 14.0. **HRMS** (ESI) *m/z*: $[M+Na]^+$ Calcd for $C_{24}H_{21}NNaO_6S$ 474.0987; found: 474.0998. **IR** 3031, 1761, 1725, 1611, 1470, 1355, 1261, 1157, 1084, 958 cm^{-1}.

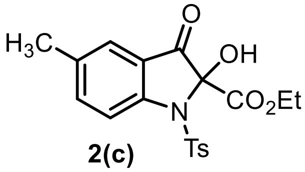

2(c)

Following general procedure, **1**(**c**) (100 mg, 0.27 mmol) and PIFA (229 mg, 0.54 mmol) in dry DCM (0.02 M). The crude residue was purified by silica gel column chromatography using EtOAc:Hexane (15:85, v:v) to afford a yellow color solid, 64 mg, 62% yield. **^{1}H NMR** (400 MHz, $CDCl_3$) δ 7.96 (m, 2H), 7.44 (m, 3H), 7.30 (m, 2H), 5.33 (s, 1H), 4.41 (m, 1H), 4.30 (m, 1H), 2.39 (s, 3H), 2.32 (s, 3H), 1.28 (t, *J* = 8 Hz, 3H). **^{13}C{^{1}H} NMR** (101 MHz, $CDCl_3$) δ 191.3, 167.3, 150.5, 145.0, 139.4, 136.2, 133.9, 129.9, 128.0, 125.5, 120.3, 113.6, 87.5, 64.4, 21.7, 20.6, 13.9. **HRMS** (ESI) *m/z*: $[M+Na]^+$ Calcd for $C_{19}H_{19}NNaO_6S$ 412.0831; found: 412.0843 **IR** 2926, 2848, 2304, 1761, 1724, 1617, 1487, 1362, 1247, 1141, 916, 672 cm^{-1}. **mp** 109 – 112 °C.

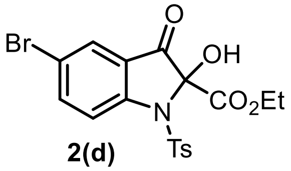

2(d)

Following general procedure, **1**(**d**) (100 mg, 0.23 mmol) and PIFA (195 mg, 0.46 mmol) in dry DCM (0.02 M). The crude residue was purified by silica gel column chromatography using EtOAc:Hexane (20:80, v:v) to afford a light orange color solid, 100 mg, 97% yield. **^{1}H NMR** (400 MHz, $CDCl_3$) δ 7.98 – 7.93 (m, 2H), 7.82 – 7.78 (m, 1H), 7.68 (m, 1H), 7.50 – 7.45 (m, 1H), 7.32 (m, 2H), 5.37 (s, 1H), 4.42 (m, 1H), 4.31 (m, 1H), 2.41 (s, 3H), 1.29 (t, *J* = 8 Hz, 3H). **^{13}C{^{1}H} NMR** (101 MHz, $CDCl_3$) δ 190.1, 166.8, 151.2, 145.4, 140.8, 135.8, 130.0, 128.3, 128.0, 121.8, 116.8, 115.4, 87.6, 64.7, 21.7, 14.0. **HRMS** (ESI) *m/z*: $[M+H]^+$ Calcd for $C_{18}H_{17}BrNO_6S$ 453.9960; found: 453.9954. **IR** 2926, 2853, 1759, 1730, 1593, 1453, 1364, 1247, 1150, 1128, 1079, 951 cm^{-1}. **mp** 114 – 117 °C.

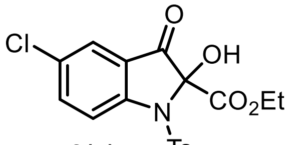

2(e)

Following general procedure, **1(e)** (100 mg, 0.25 mmol) and PIFA (218 mg, 0.5 mmol) in dry DCM (0.02 M). The crude residue was purified by silica gel column chromatography using EtOAc:Hexane (12:82, v:v) to afford an orange color solid, 76 mg, 73% yield. **^{1}H NMR** (400 MHz, $CDCl_3$) δ 7.95 (m, 2H), 7.64 (m, 1H), 7.57 – 7.51 (m, 2H), 7.31 (m, 2H), 5.40 (s, 1H), 4.42 (m, 1H), 4.31 (m, 1H), 2.40 (s, 3H), 1.29 (t, J = 8 Hz, 3H). **^{13}C{^{1}H} NMR** (101 MHz, $CDCl_3$) δ 190.2, 166.8, 150.7, 145.4, 138.0, 135.8, 130.0, 129.7, 128.0, 125.2, 121.4, 115.1, 87.7, 64.7, 21.7, 13.9. **HRMS** (ESI) *m/z*: $[M+Na]^+$ Calcd for $C_{18}H_{16}ClNNaO_6S$ 432.0284; found: 432.0286. **IR** 3358, 3009, 1729, 1738, 1364, 1150, 963 cm^{-1}. **mp** 117 – 120 °C.

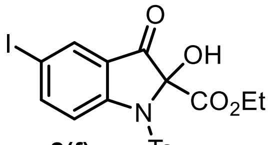

2(f)

Following general procedure, **1(f)** (100 mg, 0.21 mmol) and PIFA (176 mg, 0.42 mmol) in dry DCM (0.02 M). The crude residue was purified by silica gel column chromatography using EtOAc:Hexane (20:80, v:v) to afford a yellow color solid, 85 mg, 82% yield. **^{1}H NMR** (400 MHz, $CDCl_3$) δ 7.97 (m, 3H), 7.85 (m, 1H), 7.38 – 7.28 (m, 3H), 5.34 (s, 1H), 4.42 (m, 1H), 4.31 (m, 1H), 2.41 (s, 3H), 1.29 (t, J = 8 Hz, 3H). **^{13}C{^{1}H} NMR** (101 MHz, $CDCl_3$) δ 189.9, 166.8, 151.8, 146.4, 145.4, 135.8, 134.3, 130.0, 128.0, 122.3, 115.8, 87.3, 86.4, 64.7, 21.7, 14.0. **HRMS** (ESI) *m/z*: $[M+H]^+$ Calcd for $C_{18}H_{17}INO_6S$ 501.9821; found: 501.9823 **IR** 3235, 3040, 1752, 1728, 1591, 1450, 1357, 1252, 1128, 1078, 1007, 949 cm^{-1}. **mp** 125 – 128 °C.

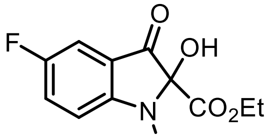

2(g)

Following general procedure, **1(g)** (100 mg, 0.26 mmol) and PIFA (226 mg, 0.52 mmol) in dry DCM (0.02 M). The crude residue was purified by silica gel column chromatography using EtOAc:Hexane (15:85, v:v) to afford a yellow color solid, 101 mg, 97% yield. **^{1}H NMR** (400 MHz, $CDCl_3$) δ 7.99 – 7.92 (m, 2H), 7.55 (m, 1H), 7.38 – 7.28 (m, 4H), 5.35 (s, 1H), 4.43 (m, 1H), 4.32 (m, 1H), 2.41 (s, 3H), 1.30 (t, J = 8 Hz, 3H). **^{13}C{^{1}H} NMR** (101 MHz, $CDCl_3$) δ 190.7, 190.7, 166.9, 160.3, 157.8, 148.7, 148.7, 145.3, 135.9, 130.0, 128.0, 125.8, 125.6, 121.3, 121.3, 115.3, 115.2, 111.6, 111.3, 87.9, 64.6, 21.7, 14.0. **^{19}F NMR** (376 MHz, $CDCl_3$) δ -118.01. **HRMS** (ESI) *m/z*: $[M+Na]^+$ Calcd for $C_{18}H_{16}FNNaO_6S$ 416.0580; found: 416.0589. **IR** 3372, 2928, 1739, 1729, 1477, 905 cm^{-1}. **mp** 131 – 134 °C.

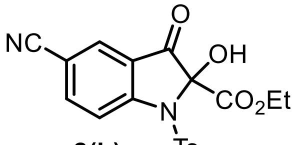

2(h)

Following general procedure, **1(h)** (100 mg, 0.26 mmol) and PIFA (222 mg, 0.52 mmol) in dry DCM (0.02 M). The crude residue was purified by silica gel column chromatography using EtOAc:Hexane (20:80, v:v) to afford an orange color oil, 90 mg, 87% yield. **^{1}H NMR** (400 MHz, $CDCl_3$) δ 8.00 – 7.93 (m, 3H), 7.84 (m, 1H), 7.68 (m, 1H), 7.36 – 7.31 (m, 2H), 4.44 (m, 1H), 4.31 (m, 1H), 2.41 (s, 3H), 1.29 (t, J = 8 Hz, 3H). **^{13}C{^{1}H} NMR** (101 MHz, $CDCl_3$) δ 189.7, 166.3, 154.4, 145.9, 141.0, 135.2, 130.2, 130.1, 128.0, 120.6, 117.3, 114.6, 107.6, 87.8, 64.9, 21.7, 13.9. **HRMS** (ESI) *m/z*: $[M+Na]^+$ Calcd for $C_{19}H_{16}N_2NaO_6S$ 423.0627; found: 423.0621. **IR** 3312, 3028, 2233, 1741, 1616., 1580, 900 cm^{-1}.

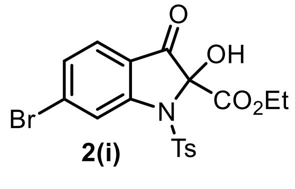

2(i)

Following general procedure, **1**(**i**) (100 mg, 0.22 mmol) and PIFA (195 mg, 0.44 mmol) in dry DCM (0.02 M). The crude residue was purified by silica gel column chromatography using EtOAc:Hexane (25:75, v:v) to afford an orange color solid, 95 mg, 92% yield. **^{1}H NMR** (400 MHz, $CDCl_3$) δ 7.99 – 7.94 (m, 2H), 7.76 (m, 1H), 7.54 (m, 1H), 7.37 – 7.31 (m, 2H), 7.28 (m, 1H), 5.39 (bs, 1H), 4.42 (m, 1H), 4.30 (m, 1H), 2.41 (s, 3H), 1.29 (t, J = 8 Hz, 3H). **^{13}C{^{1}H} NMR** (101 MHz, $CDCl_3$) δ 190.2, 166.8, 152.8, 145.4, 135.7, 134.0, 130.1, 128.0, 127.5, 126.6, 119.1, 117.2, 87.6, 64.6, 21.7, 13.9. **HRMS** (ESI) *m/z*: $[M+H]^+$ Calcd for $C_{18}H_{17}BrNO_6S$ 453.9960; found: 453.9954. **IR** 3434, 3109, 1722, 1589, 1153, 9512 cm^{-1}.

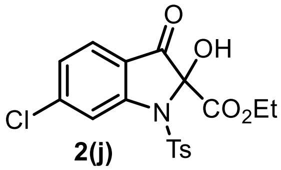

2(j)

Following general procedure, **1**(**j**) (100 mg, 0.25 mmol) and PIFA (218 mg, 0.5 mmol) in dry DCM (0.02 M). The crude residue was purified by silica gel column chromatography using EtOAc:Hexane (17:83, v:v) to afford an orange color solid, 78 mg, 75% yield. **^{1}H NMR** (400 MHz, $CDCl_3$) δ 7.97 (d, 2H), 7.64 – 7.60 (m, 1H), 7.59 – 7.57 (m, 1H), 7.34 (m, 2H), 7.12 (m, 1H), 4.43 (m, 1H), 4.31 (m, 1H), 2.42 (s, 3H), 1.29 (t, J = 8 Hz, 3H). **^{13}C{^{1}H} NMR** (101 MHz, $CDCl_3$) δ 189.9, 166.9, 152.9, 145.5, 145.1, 135.7, 130.1, 128.0, 126.7, 124.6, 118.7, 114.3, 87.8, 64.7, 21.7, 14.0. **HRMS** (ESI) *m/z*: $[M+H]^+$ Calcd for $C_{18}H_{17}ClNO_6S$ 410.0465; found: 410.0479. **IR** 3449, 3014, 1733, 1599, 1153, 955 cm^{-1}. **mp** 122 – 125 °C.

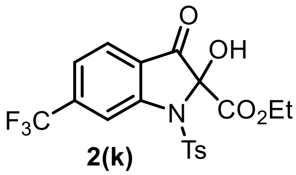

2(k)

Following general procedure, **1**(**k**) (100 mg, 0.23 mmol) and PIFA (200 mg, 0.46 mmol) in dry DCM (0.02 M). The crude residue was purified by silica gel column chromatography using EtOAc:Hexane (14:86, v:v) to afford an orange color solid, 102 mg, 98% yield. **^{1}H NMR** (400 MHz, $CDCl_3$) δ 8.00 – 7.95 (m, 2H), 7.85 – 7.78 (m, 2H), 7.39 (m, 1H), 7.34 (m, 2H), 5.46 (s, 1H), 4.44 (m, 1H), 4.32 (m, 1H), 2.42 (s, 3H), 1.30 (t, J = 8 Hz, 3H). **^{13}C{^{1}H} NMR** (101 MHz, $CDCl_3$) δ 190.8, 166.7, 152.2, 145.7, 139.6, 139.3, 139.0, 138.6, 135.5, 130.1, 128.0, 126.5, 122.6, 121.6, 120.7, 120.7, 111.1, 111.0, 111.0, 111.0, 87.6, 64.8, 21.7, 14.0. **^{19}F NMR** (376 MHz, $CDCl_3$) δ -63.49. **HRMS** (ESI) *m/z*: $[M+Na]^+$ Calcd for $C_{19}H_{16}F_3NNaO_6S$ 466.0548; found: 466.0557. **IR** 3420, 2985, 1748, 1604, 1157, 972 cm^{-1}. **mp** 125 – 128 °C.

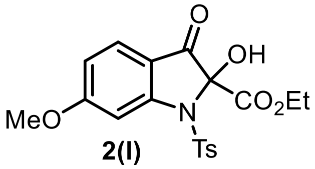

2(l)

Following general procedure, **1**(**l**) (100 mg, 0.25 mmol) and PIFA (220 mg, 0.5 mmol) in dry DCM (0.02 M). The crude residue was purified by silica gel column chromatography using EtOAc:Hexane (25:75, v:v) to afford a light yellow color solid, 67 mg, 65% yield. **^{1}H NMR** (400 MHz, $CDCl_3$) δ 7.97 (m, 2H), 7.61 (m, 1H), 7.31 (m, 2H), 7.00 (m, 1H), 6.65 (m, 1H), 5.34 (s, 1H), 4.42 (m, 1H), 4.30 (m, 1H), 3.87 (s, 3H), 2.40 (s, 3H), 1.29 (t, J = 8 Hz, 3H). **^{13}C{^{1}H} NMR** (101 MHz, $CDCl_3$) δ 188.8, 168.2, 167.4, 154.6, 145.1, 136.1, 129.9, 128.0, 127.6, 113.4, 111.6, 98.5, 88.1, 64.4, 56.1, 21.7, 14.0. **HRMS** (ESI) *m/z*: $[M+H]^+$ Calcd for $C_{19}H_{20}NO_7S$ 406.0960; found: 406.0956. **IR** 3396, 3034, 1724, 1604, 1585, 967. cm^{-1}. **mp** 133 – 136 °C.

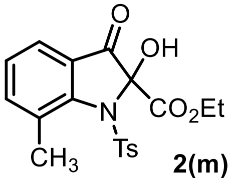

2(m)

Following general procedure, **1**(**m**) (100 mg, 0.26 mmol) and PIFA (229 mg, 0.52 mmol) in dry DCM (0.02 M). The crude residue was purified by silica gel column chromatography using EtOAc:Hexane (20:80, v:v) to afford a yellow color solid, 95 mg, 92% yield. **^{1}H NMR** (400 MHz, $CDCl_3$) δ 7.98 – 7.92 (m, 2H), 7.63 (m, 1H), 7.38 – 7.29 (m, 3H), 7.09 (m, 1H), 5.37 (s, 1H), 4.44 (m, 1H), 4.27 (m, 1H), 2.42 (s, 3H), 2.16 (s, 3H), 1.29 (t, J = 8 Hz, 3H). **^{13}C{^{1}H} NMR** (101 MHz, $CDCl_3$) δ 191.7, 167.2, 151.4, 144.6, 142.6, 138.5, 130.0, 127.1, 125.5, 124.6, 123.7, 122.5, 88.6, 64.3, 21.7, 21.7, 13.9. **HRMS** (ESI) *m/z*: $[M+H]^+$ Calcd for $C_{19}H_{20}NO_6S$ 390.1011; found: 390.1000. **IR** 3436, 3040,1753, 1722, 1148, 967 cm^{-1}. **mp** 131 – 134 °C.

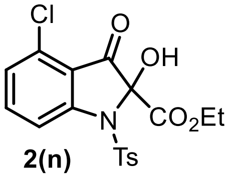

2(n)

Following general procedure, **1**(**n**) (100 mg, 0.24 mmol) and PIFA (218 mg, 0.48 mmol) in dry DCM (0.02 M). The crude residue was purified by silica gel column chromatography using EtOAc:Hexane (17:83, v:v) to afford a yellow color solid, 88 mg, 85% yield. **^{1}H NMR** (400 MHz, $CDCl_3$) δ 8.00 – 7.96 (m, 2H), 7.50 – 7.43 (m, 2H), 7.32 (m, 2H), 7.08 (m, 1H), 5.42 (s, 1H), 4.46 (m, 1H), 4.31 (m, 1H), 2.41 (s, 3H), 1.31 (t, J = 8 Hz, 3H). **^{13}C{^{1}H} NMR** (101 MHz, $CDCl_3$) δ 188.5, 166.9, 153.5, 145.4, 138.2, 135.7, 134.0, 130.0, 128.1, 125.2, 117.1, 111.9, 87.3, 64.7, 21.7, 14.0. **HRMS** (ESI) *m/z*: $[M+Na]^+$ Calcd for $C_{18}H_{16}ClNNaO_6S$ 432.0284; found: 432.0291. **IR** 3378, 2932, 1786, 1750, 1080, 957 cm^{-1}. **mp** 124 – 127 °C.

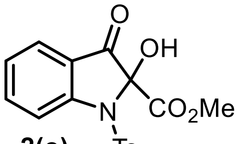

2(o)

Following general procedure, **1**(**o**) (100 mg, 0.28 mmol) and PIFA (247 mg, 0.56 mmol) in dry DCM (0.02 M). The crude residue was purified by silica gel column chromatography using EtOAc:Hexane (35:65, v:v) to afford a yellow color solid, 92 mg, 88% yield. **^{1}H NMR** (400 MHz, $CDCl_3$) δ 7.98 (m, 2H), 7.71 – 7.55 (m, 3H), 7.31 (m, 2H), 7.18 – 7.11 (m, 1H), 5.35 (s, 1H), 3.87 (s, 3H), 2.39 (s, 3H). **^{13}C{^{1}H} NMR** (101 MHz, $CDCl_3$) δ 191.0, 167.7, 152.3, 145.2, 138.4, 135.9, 129.9, 128.0, 125.9, 124.0, 120.2, 113.8, 87.3, 54.7, 21.7. **HRMS** (ESI) *m/z*: $[M+Na]^+$ Calcd for $C_{17}H_{15}NNaO_6S$ 384.0518; found: 384.0507. **IR** 2925, 1749, 1593, 1246, 951 cm^{-1}. **mp** 149 – 152 °C.

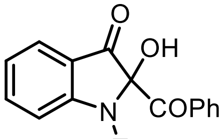

2(p)

Following general procedure, **1**(**p**) (26 mg, 0.07 mmol) and PIFA (58 mg, 0.14 mmol) in dry DCM (6 mM). The crude residue was purified by silica gel column chromatography using EtOAc:Hexane (15:85, v:v) to afford an amorphous yellow color solid, 16 mg, 60% yield. **^{1}H NMR** (400 MHz, $CDCl_3$) δ 8.02 – 7.95 (m, 2H), 7.86 – 7.76 (m, 3H), 7.73 – 7.68 (m, 2H), 7.60 – 7.54 (m, 1H), 7.40 – 7.33 (m, 2H), 7.29 (m, 2H), 7.25 – 7.21 (m, 1H), 6.47 (s, 1H), 2.40 (s, 3H). **^{13}C{^{1}H} NMR** (101 MHz, $CDCl_3$) δ 191.7, 191.0, 152.1, 145.2, 138.5, 135.7, 134.7, 131.7, 129.8, 129.2, 129.1, 128.4, 126.3, 124.4, 120.8, 114.6, 90.4, 21.7. **HRMS** (ESI) *m/z*: $[M+Na]^+$ Calcd for $C_{22}H_{17}NO_5SNa$ 430.0725; found: 430.0706. **IR** 3070, 2923, 1732, 1683, 1359, 753 cm^{-1}.

Synthesis of compounds **2q-2w** in CH_3CN as solvent

Compound **1a-1c**, **1e**, **1i-1k** was dissolved in 12 mL dry CH_3CN (0.02-0.03 M). PIFA (2 eq) was added portionwise at room temperature. The reaction was allowed to stir overnight. Then, 10 ml of water was added and the mixture stirred for 30 min. The mixture was extracted 3 times with water and DCM and dried over sodium sulfate. The product was purified by column chromatography with ethyl acetate and hexane as eluents.

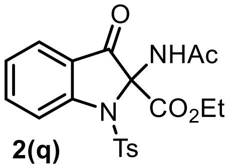

2(q)

Following general procedure, **1**(**a**) (140 mg, 0.39 mmol) and PIFA (333 mg, 0.78 mmol) in dry CH_3CN (0.03 M). The crude residue was purified by silica gel column chromatography using EtOAc:Hexane (40:60, v:v) to afford a white color solid, 105 mg, 65% yield. **^{1}H NMR** (400 MHz, $CDCl_3$) δ 7.79 – 7.72 (m, 4H), 7.63 (m, 1H), 7.36 (s, 1H), 7.28 (m, 2H), 7.17 (m, 1H), 4.37 (m, 1H), 4.27 – 4.18 (m, 1H), 2.39 (s, 3H), 1.76 (s, 3H), 1.24 (t, J = 8 Hz, 3H). **^{13}C{^{1}H} NMR** (101 MHz, $CDCl_3$) δ 188.9, 168.9, 164.9, 151.6, 144.9, 137.3, 137.2, 130.0, 126.6, 125.0, 123.6, 122.2, 113.6, 77.7, 64.3, 22.1, 21.7, 13.8. **HRMS** (ESI) *m/z*: $[M+Na]^+$ Calcd for $C_{20}H_{20}N_2NaO_6S$ 439.0940; found: 439.0948 **IR** 2249, 1759, 1728, 1666, 1602, 1458, 1357, 1214, 1153, 1081 cm^{-1}. **mp** 209 – 212 °C.

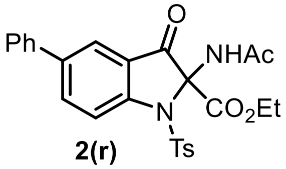

2(r)

Following general procedure, **1**(**b**) (130 mg, 0.29 mmol) and PIFA (256 mg, 0.58 mmol) in dry CH_3CN (0.02 M). The crude residue was purified by silica gel column chromatography using EtOAc:Hexane (40:60, v:v) to afford a yellow color solid, 81 mg, 55% yield. **^{1}H NMR** (400 MHz, $CDCl_3$) δ 7.94 (m, 1H), 7.86 (m, 2H), 7.80 (m, 2H), 7.57 – 7.53 (m, 2H), 7.49 – 7.41 (m, 3H), 7.38 – 7.33 (m, 1H), 7.31 – 7.28 (m, 2H), 4.39 (m, 1H), 4.25 (m, 1H), 2.39 (s, 3H), 1.77 (s, 3H), 1.27 (t, J = 8 Hz, 3H). **^{13}C{^{1}H} NMR** (101 MHz, $CDCl_3$) δ 188.9, 169.0, 164.8, 150.7, 145.0, 139.2, 137.1, 137.1, 136.3, 130.0, 129.0, 127.8, 126.9, 126.6, 123.0, 122.7, 113.9, 78.1, 64.3, 22.0, 21.6, 13.8. **HRMS** (ESI) *m/z*: $[M+H]^+$ Calcd for $C_{26}H_{25}N_2O_6S$ 493.1433; found: 493.1473. **IR** 3365, 3180, 1755, 1738, 1654, 1144, 961 cm^{-1}. **mp** 180 – 183 °C.

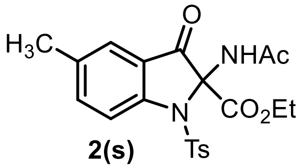

2(s)

Following general procedure, **1**(**c**) (130 mg, 0.35 mmol) and PIFA (298 mg, 0.7 mmol) in dry CH_3CN (0.03 M). The crude residue was purified by silica gel column chromatography using EtOAc:Hexane (40:60, v:v) to afford a white color solid, 80 mg, 54% yield. **^{1}H NMR** (400 MHz, $CDCl_3$) δ 7.77 – 7.73 (m, 2H), 7.65 (m, 1H), 7.52 (m, 1H), 7.43 (m, 1H), 7.37 (s, 1H), 7.28 – 7.24 (m, 2H), 4.35 (m, 1H), 4.22 (m, 1H), 2.38 (s, 3H), 2.35 (s, 3H), 1.74 (s, 3H), 1.24 (t, J = 8 Hz, 3H). **^{13}C{^{1}H} NMR** (101 MHz, $CDCl_3$) δ 188.9, 168.8, 165.0, 149.7, 144.7, 138.4, 137.3, 133.5, 129.9, 126.6, 124.8, 122.3, 113.4, 77.9, 64.2, 22.0, 21.6, 20.6, 13.8. **HRMS** (ESI) *m/z*: $[M+H]^+$ Calcd for $C_{21}H_{23}N_2O_6S$ 431.1277; found: 431.1290. **IR** 3309, 3034, 1753, 1662, 1620, 1150, 965 cm^{-1}. **mp** 210 – 213 °C.

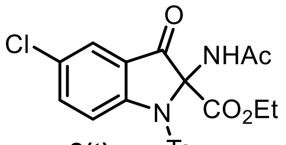

2(t)

Following general procedure, **1**(**e**) (140 mg, 0.35 mmol) and PIFA (304 mg, 0.7 mmol) in dry CH_3CN (0.03 M). The crude residue was purified by silica gel column chromatography using EtOAc:Hexane (40:60, v:v) to afford a white color solid, 92 mg, 58% yield. **^{1}H NMR** (400 MHz, $CDCl_3$) δ 7.73 – 7.67 (m, 3H), 7.65 – 7.63 (m, 1H), 7.53 (m, 1H), 7.35 (s, 1H), 7.25 (m, 2H), 4.33 (m, 1H), 4.20 (m, 1H), 2.37 (s, 3H), 1.70 (s, 3H), 1.21 (t, J = 8 Hz, 3H). **^{13}C{^{1}H} NMR** (101 MHz, $CDCl_3$) δ 187.8, 169.0, 164.4, 149.9, 145.2, 137.0, 136.9, 130.0, 129.3, 126.6, 124.5, 123.4, 114.9, 78.1, 64.4, 21.9, 21.7, 13.8. **HRMS** (ESI) *m/z*: $[M+H]^+$ Calcd for $C_{20}H_{20}ClN_2O_6S$ 451.0731; found: 451.0764. **IR** 3014, 2921, 1759, 1684, 1607, 1461, 1364, 1269, 1210, 1163, 1086, 1011, 886 cm^{-1}. **mp** 192 – 195 °C.

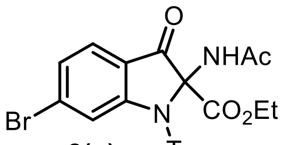

2(u)

Following general procedure, **1**(**i**) (130 mg, 0.29 mmol) and PIFA (254 mg, 0.58 mmol) in dry CH_3CN (0.02 M). The crude residue was purified by silica gel column chromatography using EtOAc:Hexane (35:65, v:v) to afford a white color solid, 68 mg, 46% yield. **^{1}H NMR** (400 MHz, $CDCl_3$) δ 8.00 (m, 1H), 7.77 – 7.73 (m, 2H), 7.59 – 7.55 (m, 1H), 7.35 – 7.28 (m, 4H), 4.37 (m, 1H), 4.23 (m, 1H), 2.41 (s, 3H), 1.73 (s, 3H), 1.26 (d, J = 8 Hz, 3H). **^{13}C{^{1}H} NMR** (101 MHz, $CDCl_3$) δ 187.9, 168.9, 164.5, 152.1, 145.2, 136.9, 132.7, 130.1, 127.2, 126.6, 125.9, 121.1, 117.0, 77.9, 64.5, 22.0, 21.7, 13.9. **HRMS** (ESI) *m/z*: $[M+H]^+$ Calcd for $C_{20}H_{20}BrN_2O_6S$ 495.0226; found: 495.0241. **IR** 3299, 3007, 1741, 1660, 1596, 955 cm^{-1}. **mp** 229 – 232 °C.

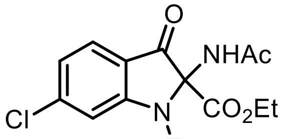

2(v)

Following general procedure, **1**(**j**) (130 mg, 0.33 mmol) and PIFA (282 mg, 0.66 mmol) in dry CH_3CN (0.03 M). The crude residue was purified by silica gel column chromatography using EtOAc:Hexane (40:60, v:v) to afford a light orange color solid, 114 mg, 77% yield. **^{1}H NMR** (400 MHz, $CDCl_3$) δ 7.81 (m, 1H), 7.75 (m, 2H), 7.65 (m, 1H), 7.34 (s, 1H), 7.33 – 7.28 (m, 2H), 7.15 (m, 1H), 4.37 (m, 1H), 4.23 (m, 1H), 2.41 (s, 3H), 1.73 (s, 3H), 1.25 (t, J = 8 Hz, 3H). **^{13}C{^{1}H} NMR** (101 MHz, $CDCl_3$) δ 187.6, 168.9, 164.5, 152.2, 145.2, 143.9, 136.9, 130.1, 126.6, 125.8, 124.3, 120.7, 114.1, 78.1, 64.4, 22.0, 21.7, 13.8. **HRMS** (ESI) *m/z*: $[M+H]^+$ Calcd for $C_{20}H_{20}ClN_2O_6S$ 451.0731; found: 451.0755. **IR** 3316, 3016, 1781, 1748, 1656, 1071, 967 cm^{-1}. **mp** 216 – 219 °C.

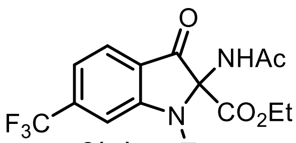

2(w)

Following general procedure, **1**(**k**) (130 mg, 0.3 mmol) and PIFA (260 mg, 0.6 mmol) in dry CH_3CN (0.03 M). The crude residue was purified by silica gel column chromatography using EtOAc:Hexane (30:70, v:v) to afford an orange color solid, 103 mg, 70% yield. **^{1}H NMR** (400 MHz, $CDCl_3$) δ 8.06 (s, 1H), 7.83 (m, 1H), 7.78 – 7.72 (m, 2H), 7.45 – 7.41 (m, 2H), 7.31 (m, 2H), 4.38 (m, 1H), 4.25 (m, 1H), 2.41 (s, 3H), 1.74 (s, 3H), 1.26 (t, J = 8 Hz, 3H). **^{13}C{^{1}H} NMR** (101 MHz, $CDCl_3$) δ 188.2, 169.2, 164.3, 151.3, 145.4, 138.7, 138.3, 138.0, 137.7, 136.7, 130.1, 126.6, 125.5, 124.6, 124.5, 121.9, 120.5, 120.5, 110.9, 110.8, 110.8, 78.0, 64.6, 21.8, 21.7, 13.8. **^{19}F NMR** (376 MHz, $CDCl_3$) δ -63.16. **HRMS** (ESI) *m/z*: $[M+H]^+$ Calcd for $C_{21}H_{20}F_3N_2O_6S$ 485.0994; found: 485.1038. **IR** 3288, 3019, 1744, 1709, 1654, 943 cm^{-1}. **mp** 203 – 206 °C.

4. Procedure for the Synthetic Transformations

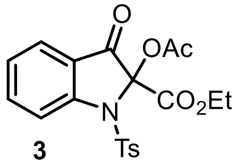

3

Compound **2a** (0.3 g, 0.8 mmol) was dissolved in 5 mL dry DCM (0.16 M). Triethylamine (4 mL) was added, and the mixture was stirred at room temperature for 10 minutes. Acetyl chloride (1.17 mL) was added, and the mixture was stirred at reflux in an oil bath for 2 hours. The crude mixture was extracted 3 times with water and DCM and dried over sodium sulfate. The product was purified by silica gel column chromatography using EtOAc/Hexane (25:75 v:v) to afford a yellow color solid, 233 mg, 69 % yield. **^{1}H NMR** (400 MHz, $CDCl_3$) δ 7.86 – 7.81 (m, 3H), 7.71 (m, 1H), 7.65 (m, 1H), 7.31 (m, 2H), 7.19 (m, 1H), 4.33 (m, 1H), 4.22 (m, 1H), 2.41 (s, 3H), 1.88 (s, 3H), 1.25 (t, J = 8 Hz, 3H). **^{13}C{^{1}H} NMR** (101 MHz, $CDCl_3$) δ 188.2, 168.0, 162.5, 151.5, 145.3, 137.6, 137.0, 130.1, 126.8, 125.1, 123.9, 121.7, 113.7, 87.9, 63.6, 21.7, 20.0, 13.8. **HRMS** (ESI) *m/z*: $[M+Na]^+$ Calcd for $C_{20}H_{19}NNaO_7S$ 440.0780; found: 440.0781. **IR** 3115.44, 1764, 1642, 1603, 1087, 950 cm^{-1}. **mp** 131 – 134 °C.

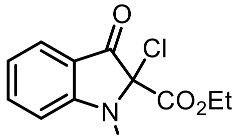

4

Imidazole (72 mg, 1 mmol, 8 eq) was dissolved in 3 ml dry DCM (0.33 M). Thionyl chloride (2.5 eq, 0.32 mmol, 25 µL) was added at 0 °C. The mixture was stirred at that temperature for 10 minutes and then compound **2a** (50 mg, 0.13 mmol) dissolved in 1 mL dry DCM was added dropwise. The mixture was stirred at 0 °C for 1 hour. The crude mixture was extracted 3 times with water and DCM and dried over sodium sulfate. The product was purified by silica gel column chromatography using EtOAc/Hexane (18:82 v:v) to afford an amorphous yellow color solid, 21 mg, 40% yield. **^{1}H NMR** (400 MHz, $CDCl_3$) δ 8.07 – 8.01 (m, 2H), 7.78 (m, 1H), 7.67 (m, 1H), 7.60 – 7.56 (m, 1H), 7.36 (m, 2H), 7.24 – 7.19 (m, 1H), 4.44 (m, 1H), 4.35 (m, 1H), 2.43 (s, 3H), 1.34 (t, J = 8 Hz, 3H). **^{13}C{^{1}H} NMR** (101 MHz, $CDCl_3$) δ 187.2, 162.4, 151.7, 145.8, 138.5, 135.6, 130.1, 128.4, 126.7, 124.5, 119.6, 114.2, 79.8, 64.7. **HRMS** (ESI) *m/z*: $[M+Na]^+$ Calcd for $C_{18}H_{16}ClNNaO_5S$ 416.0335; found: 416.0342. **IR** 2922, 1746, 1177, 950 cm^{-1}.

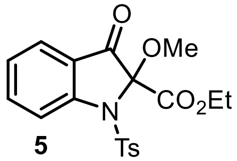

5

Compound **2a** (0.8 g, 2.1 mmol) was dissolved in CH_3CN:DMF (2:1) mixture (0.21 M). Cs_2CO_3 (3.5 eq, 7.4 mmol, 2.43 g) was added followed by dropwise addition of dimethylsulfate (8 eq, 17 mmol, 1.6 mL). The mixture was stirred at room temperature overnight. Solvents were evaporated and the product was purified by silica gel column chromatography using EtOAc/Hexane (20:80 v:v) to afford a yellow color solid, 678 mg, 81% yield. **^{1}H NMR** (400 MHz, $CDCl_3$) δ 8.03 – 7.97 (m, 2H), 7.84 – 7.79 (m, 1H), 7.71 – 7.64 (m, 2H), 7.34 (m, 2H), 7.16 (m, 1H), 4.37 – 4.29 (m, 1H), 4.29 – 4.21 (m, 1H), 2.94 (s, 3H), 2.42 (s, 3H), 1.26 (t, J = 8 Hz, 3H). **^{13}C{^{1}H} NMR** (101 MHz, $CDCl_3$) δ 191.2, 164.1, 153.3, 145.2, 138.6, 136.5, 130.0, 127.7, 125.4, 123.8, 120.9, 113.9, 93.0, 63.2, 52.8, 21.7, 14.0. **HRMS** (ESI) *m/z*: $[M+H]^+$ Calcd for $C_{19}H_{20}NO_6S$ 390.1011; found: 390.1000. **IR** 3028, 1731, 1587, 1157, 950 cm^{-1}. **mp** 112 – 115 °C.

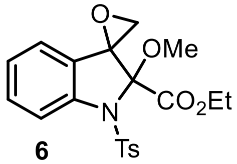

6

TMSOI (367 mg, 1.6 mmol, 5 eq) was dissolved in 3 ml dry DMSO (0.5 M). NaH (48 mg, 2 mmol) was added and the mixture was stirred at room temperature for 5 minutes. Compound **5** (130 mg, 0.3 mmol) dissolved in 1 mL dry DMSO was added dropwise. The mixture was stirred at room

temperature for 3 hours. The crude mixture was extracted 3 times with NH_4Cl and ethyl acetate and dried over sodium sulfate. The product was purified by silica gel column chromatography using EtOAc/Hexane (40:60 v:v) to afford an amorphous yellow color solid, 50 mg, 37% yield. **^{1}H NMR** (400 MHz, $CDCl_3$) δ 8.06 – 8.01 (m, 2H), 7.34 (m, 4H), 7.06 – 6.98 (m, 2H), 4.37 – 4.29 (m, 2H), 3.53 (m, 1H), 3.46 (m, 1H), 3.03 (s, 3H), 2.41 (s, 3H), 1.31 (t, *J* = 8 Hz, 3H). **^{13}C{^{1}H} NMR** (101 MHz, $CDCl_3$) δ 165.0, 144.8, 144.4, 136.4, 131.4, 129.8, 128.1, 123.4, 123.2, 122.6, 112.2, 97.5, 63.8, 62.7, 51.8, 51.8, 41.0, 21.7, 14.1. **HRMS** (ESI) *m/z*: $[M+H]^+$ Calcd for $C_{20}H_{22}NO_6S$ 404.1168; found: 404.1171. **IR** 2923, 1765, 1677, 1355, 1163, 1016 cm^{-1}.

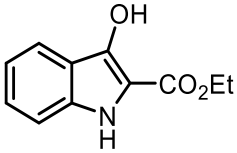

Compound **5** (188 mg, 0.48 mmol) and magnesium turnings (586 mg, 24 mmol, 50 eq) were dissolved in 12 mL dry MeOH (0.04 M) under N_2 atmosphere. The suspension was placed in an ultrasonicator at room temperature and sonicated for 30 minutes, with the flask being manually shaken every minute for the first 5 min to ensure thorough mixing. The suspension was poured over ethyl acetate with vigorous shaking to prevent a thick gel from forming. The crude mixture was extracted 3 times with ammonium chloride solution and ethyl acetate and dried over sodium sulfate. The product was purified by silica gel column chromatography using EtOAc/Hexane (9:91 v:v) to afford a purple color solid, 60 mg, 61% yield. **^{1}H NMR** (400 MHz, $CDCl_3$) δ 7.83 (s, 1H), 7.73 (m, 1H), 7.33 (m, 1H), 7.25 (m, 1H), 7.08 (m, 1H), 4.42 (q, *J* = 8 Hz, 2H), 1.41 (t, *J* = 8 Hz, 3H). **^{13}C{^{1}H} NMR** (101 MHz, $CDCl_3$) δ 163.5, 147.5, 135.4, 127.3, 120.2, 119.8, 117.9, 112.0, 108.2, 60.8, 14.6. **HRMS** (ESI) *m/z*: $[M+H]^+$ Calcd for $C_{11}H_{12}NO_3$ 206.0817; found: 206.0825. **IR** 3507, 3337, 2920, 1674, 1617, 1327, 1232, 1012, 742 cm^{-1}. **mp** 93 – 96 °C.

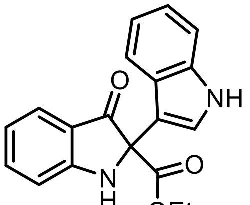

7

The deprotected indole from previous step (30 mg, 0.15 mmol) was dissolved in 12 mL dry DCM (1.3 mM). PIFA (62 mg, 0.15 mmol, 1 eq) was added. The solution was stirred at room temperature for 5 minutes and indole (42 mg, 0.36 mmol, 2.5 eq) was added. The solution was stirred at room temperature for 1 hour and extracted 3 times with water and DCM and dried over sodium sulfate. Product was purified by silica gel column chromatography using EtOAc/Hexane (20:80 v:v) to afford 43 mg of yellow solid, 43 mg, 92% yield. **^{1}H NMR** (400 MHz, $CDCl_3$) δ 8.40 (s, 1H), 7.69 (m, 1H), 7.62 – 7.57 (m, 1H), 7.52 (m, 1H), 7.33 – 7.28 (m, 2H), 7.17 (m, 1H), 7.08 (m, 1H), 6.99 (m, 1H), 6.95 – 6.90 (m, 1H), 5.73 (s, 1H), 4.27 (q, *J* = 8 Hz, 2H), 1.23 (t, *J* = 8 Hz, 3H). **^{13}C{^{1}H} NMR** (101 MHz, $CDCl_3$) δ 195.0, 168.5, 161.2, 137.9, 136.6, 125.5, 125.4, 123.7, 122.6, 120.3, 120.3, 119.9, 119.6, 113.6, 111.7, 111.6, 72.7, 63.1, 14.1. **HRMS** (ESI) *m/z*: $[M+H]^+$ Calcd for $C_{19}H_{17}N_2O_3$ 321.1239; found: 321.1257. **IR** 3390, 3348, 2917, 1726, 1676, 1232, 744 cm^{-1}. **mp** 189 – 192 °C.

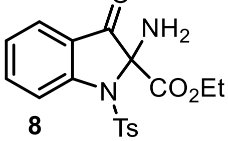

8

Compound **2q** (42 mg, 0.1 mmol) was dissolved in 10 mL Ethanol (0.01 M). 2 mL of conc. HCl were added, and the mixture was stirred at 100 °C overnight. Then, pH was adjusted to 7 by adding solid $NaHCO_3$ and was extracted 3 times with water and ethyl acetate. The organic layer was dried over sodium sulfate and the product was purified by silica gel column

chromatography using ethyl acetate and hexane as eluents (13:87, v:v). The desired product was obtained as white solid, 61% yield. **^{1}H NMR** (400 MHz, $CDCl_3$) δ 8.00 – 7.94 (m, 2H), 7.70 (m, 1H), 7.61 (m, 1H), 7.54 (m, 1H), 7.31 (m, 2H), 7.14 (m, 1H), 5.35 (s, 1H), 4.43 (m, 1H), 4.31 (m, 1H), 2.40 (s, 3H), 1.63 (s, 1H), 1.29 (t, *J* = 8 Hz, 3H). **^{13}C{^{1}H} NMR** (101 MHz, $CDCl_3$) δ 191.3, 167.2, 152.4, 145.1, 138.3, 136.1, 129.9, 128.0, 125.9, 123.9, 120.3, 113.8, 87.3, 64.5, 21.7, 14.0. **HRMS** (ESI) *m/z*: $[M+H]^+$ Calcd for $C_{18}H_{19}N_2O_5S$ 375.1015; found: 375.1009. **IR** 3435, 3410, 2988, 1599, 1456, 1249, 743 cm^{-1}. **mp** 132 – 135 °C.

Table S1: Hydrolysis Conditions:

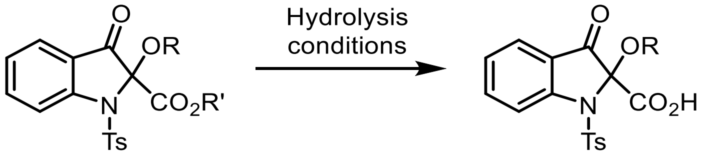

R, R'	Conditions	Yield
R = Me, R' = Et	LiOH (5 equiv), THF:H_2O (1:1)	--
R = Me, R' = Et	LiOH (1.5 equiv), THF:H_2O (1:1)	--
R = H, R' = Et	NaOH (1.2 equiv), 1,4-Dioxane:H_2O (2:1)	--
R = Me, R' = Et	CaCl2 (10 equiv) KOH (9 equiv), iPrOH:H_2O (1:1)	--
R = H, R' = Me	TMSOK (1.2 equiv), dry THF	--
R = H, R' = Et	LiCl (3.5 equiv), DMF	trace

5. Mechanistic Studies

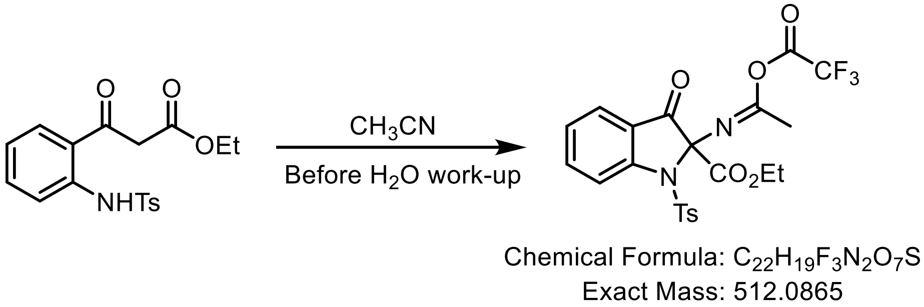

HRMS (ESI) *m/z*: $[M+H]^+$ Calcd for $C_{22}H_{20}F_3N_2O_7S$ 513.0944; found: 513.0952.

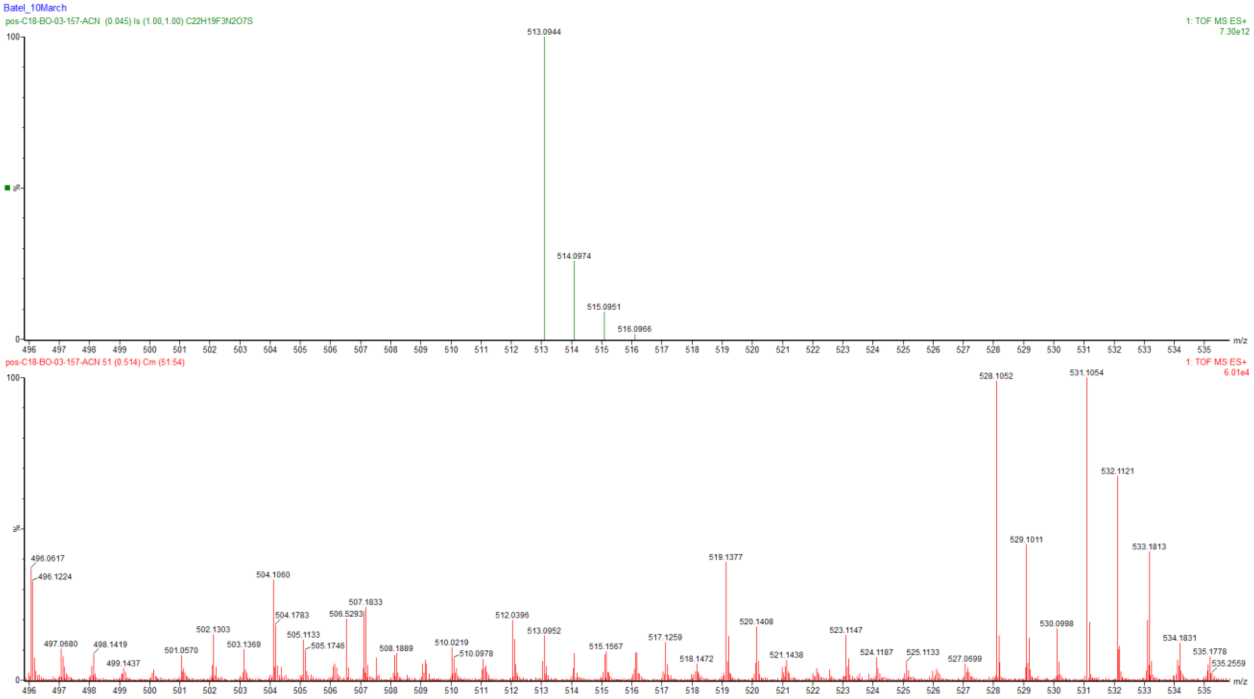

6. Computational Details

Geometries were optimized with Gaussian16 package using the B3LYP functional with D3 empirical corrections with Becke-Johnson damping. The electronic configuration of the systems was described with the 6-31G basis set for H, C, O, N, S and F while for I we adopted the quasi-relativistic LANL2DZ effective core potential with the associated valence basis set. All geometries were characterized as minimum or transition state through frequency calculations. Solvent effects were included with the PCM model using CH_2Cl_2 as the solvent. NBO analysis, as implemented

in Gaussian16 (version 3) (J. P. Foster and F. Weinhold, "Natural hybrid orbitals," *J. Am. Chem. Soc.*, **102** (1980) 7211-18. DOI: 10.1021/ja00544a007), was used to calculate partial charges.

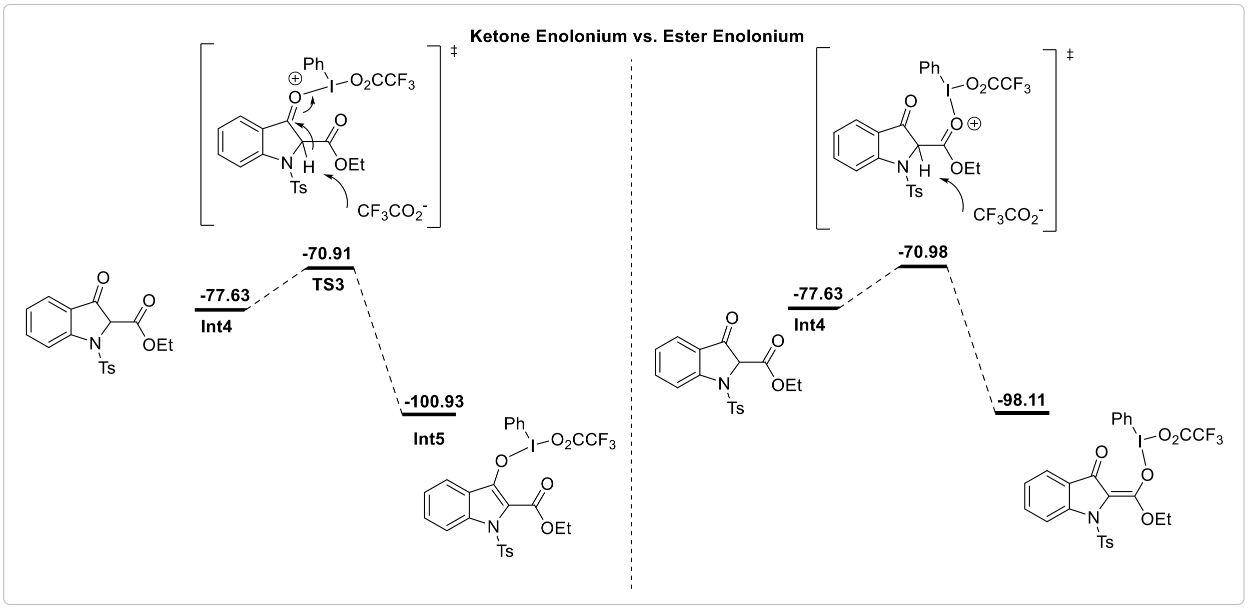

Species	Free Energy	Free Energy Correction	Number of Imaginary Frequency
**1a**	-1526.037413	0.286113	0
**PIFA**	-1294.988009	0.085091	0
**Iodobenzene**	-243.0089626	0.05968	0
**CF_3CO_2H**	-526.6454623	0.008231	0
**Int1**	-2821.091392	0.40499	0
**Int2**	-2294.414649	0.375271	0
**TS1**	-2821.076445	0.40058	1
**Int3**	-2294.43301	0.375056	0
**TS2**	-2820.651488	0.390212	1
**Int4**	-1524.820559	0.266365	0
**TS3**	-2819.859516	0.37628	1
**Int5**	-2293.206831	0.349825	0
**Int6**	-2293.222238	0.35247	0
**TS4**	-2819.418497	0.369997	1
**Int7**	-2050.252123	0.277118	0
**2a**	-1600.010452	0.269756	0

NBO analysis of **Int1**

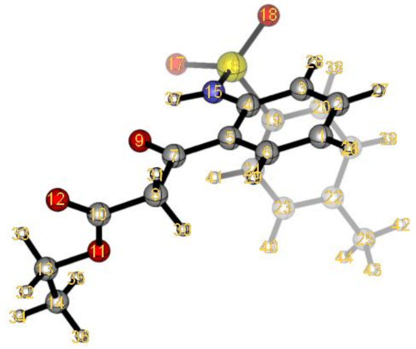

Atom	No	Charge
C	1	-0.27541
C	2	-0.19062
C	3	-0.29338
C	4	0.20458
C	5	-0.18545
C	6	-0.18688
C	7	0.55339
C	8	-0.64506
O	9	-0.59449
C	10	0.80490
O	11	-0.55516
O	12	-0.59840
C	13	-0.14325
C	14	-0.72758
N	15	-0.80025
S	16	1.87395
O	17	-0.82500
O	18	-0.83586
C	19	-0.24539
C	20	-0.22430
C	21	-0.23195
C	22	0.00396
C	23	-0.22664
C	24	-0.22672
C	25	-0.72438
H	26	0.26446
H	27	0.26700
H	28	0.26974
H	29	0.26578
H	30	0.30376

H	31	0.30515
H	32	0.24172
H	33	0.24462
H	34	0.25642
H	35	0.24916
H	36	0.24754
H	37	0.48552
H	38	0.28799
H	39	0.26868
H	40	0.26898
H	41	0.28333
H	42	0.25623
H	43	0.26444
H	44	0.26490

Cartesian coordinates of species reported in **Figure 2**

1a

C	-0.039731000	-3.030822000	-2.437119000
C	1.192034000	-3.003795000	-1.762692000
C	1.331422000	-2.336717000	-0.552752000
C	0.233720000	-1.659053000	0.019299000
C	-1.030952000	-1.679572000	-0.654074000
C	-1.129523000	-2.380516000	-1.876886000
C	-2.192245000	-0.988785000	-0.088359000
C	-3.495454000	-0.950991000	-0.884103000
O	-2.161629000	-0.423826000	1.037166000
C	-4.504772000	-0.044192000	-0.225770000
O	-4.175843000	1.268705000	-0.428278000
O	-5.495739000	-0.413529000	0.407142000
C	-4.990821000	2.281169000	0.282576000
C	-4.326407000	3.620682000	0.035909000
N	0.361091000	-0.958598000	1.203619000
S	1.909201000	-0.489297000	1.978366000
O	1.398930000	0.399896000	3.247411000
O	2.913725000	-1.759338000	2.210027000
C	2.669289000	0.562367000	0.639606000
C	3.964323000	0.273731000	0.235792000
C	4.535131000	1.081606000	-0.753588000
C	3.822106000	2.154487000	-1.312518000
C	2.513136000	2.411814000	-0.859777000
C	1.925239000	1.619637000	0.126398000
C	4.440005000	3.023872000	-2.380996000

H	-0.141644000	-3.562584000	-3.375564000
H	2.047861000	-3.524362000	-2.179105000
H	2.269840000	-2.373367000	-0.015359000
H	-2.083649000	-2.415208000	-2.388304000
H	-3.299736000	-0.597249000	-1.901165000
H	-3.922284000	-1.957478000	-0.941267000
H	-6.011370000	2.235342000	-0.109006000
H	-5.015810000	2.009072000	1.341114000
H	-4.888489000	4.415074000	0.539090000
H	-3.304085000	3.619553000	0.425223000
H	-4.290140000	3.846640000	-1.034194000
H	-0.475735000	-0.596473000	1.670193000
H	4.496685000	-0.558700000	0.680068000
H	5.544872000	0.876145000	-1.092632000
H	1.953523000	3.239863000	-1.282645000
H	0.917824000	1.812049000	0.476759000
H	5.460210000	2.706811000	-2.613394000
H	3.855755000	2.986103000	-3.308349000
H	4.476975000	4.072907000	-2.064078000

PIFA

C	-0.276630000	2.136781000	-0.981008000
C	0.145544000	3.459684000	-0.876391000
C	1.115687000	3.825291000	0.076288000
C	1.660974000	2.875924000	0.942928000
C	1.229764000	1.544476000	0.893210000
C	0.281151000	1.214958000	-0.080721000
I	-0.208391000	-0.832228000	-0.278165000
O	-2.276449000	-0.829234000	0.476487000
C	-3.176312000	-0.167506000	-0.270158000
O	-2.970290000	0.476831000	-1.296576000
C	-4.574201000	-0.311288000	0.357887000
F	-5.510448000	0.344916000	-0.403876000
F	-4.587501000	0.227560000	1.630336000
F	-4.931856000	-1.643214000	0.442934000
O	3.918833000	0.819531000	1.070723000
C	4.455188000	-0.052578000	0.314996000
O	5.568376000	-0.171218000	-0.242814000
C	3.446853000	-1.241666000	0.014454000
F	3.984088000	-2.357381000	-0.563178000
F	2.414268000	-0.787551000	-0.886310000
F	2.740342000	-1.651549000	1.143843000
H	-1.019720000	1.836650000	-1.707200000

H	-0.270155000	4.203507000	-1.546174000
H	1.451094000	4.854908000	0.127924000
H	2.444648000	3.135378000	1.642177000
H	1.686917000	0.810899000	1.540840000

Iodobenzene

C	1.217656000	1.266479000	0.000000000
C	1.210338000	2.666843000	0.000000000
C	0.000106000	3.368351000	0.000000000
C	-1.210164000	2.666943000	0.000000000
C	-1.217588000	1.266562000	0.000000000
C	0.000000000	0.581700000	0.000000000
I	-0.000046000	-1.570004000	0.000000000
H	2.155187000	0.724302000	0.000000000
H	2.153250000	3.203553000	0.000000000
H	0.000161000	4.452929000	0.000000000
H	-2.153050000	3.203701000	0.000000000
H	-2.155173000	0.724480000	0.000000000

CF_3CO_2H

C	0.932069000	-0.147752000	-0.000094000
O	1.576804000	1.047488000	-0.000227000
O	1.464456000	-1.250364000	0.000152000
C	-0.585386000	0.025350000	0.000015000
F	-0.985163000	1.341037000	0.000062000
F	-1.116342000	-0.591038000	1.119784000
F	-1.116492000	-0.591002000	-1.119704000
H	2.551807000	0.926446000	-0.000202000

Int1

C	2.511720000	5.479638000	-0.819547000
C	3.799880000	5.175683000	-0.351351000
C	4.116859000	3.879504000	0.049165000
C	3.162939000	2.852431000	-0.047965000
C	1.892296000	3.131021000	-0.619593000
C	1.567047000	4.467003000	-0.940543000
N	3.474577000	1.584026000	0.466945000
C	0.895150000	2.105599000	-0.975969000
O	-0.324890000	2.411755000	-1.074933000
C	1.356681000	0.702835000	-1.294256000
C	0.484371000	-0.114476000	-2.153543000
O	-0.790685000	-0.132926000	-2.190466000
O	1.150861000	-0.978257000	-2.924968000

C	0.391900000	-2.024842000	-3.690463000
C	0.042471000	-3.188840000	-2.779520000
I	-2.340746000	0.650786000	-0.679609000
O	-4.120599000	0.679866000	0.560839000
C	-4.234575000	1.792610000	1.290441000
O	-3.451542000	2.746386000	1.271169000
C	4.477924000	-0.992687000	0.513930000
C	4.954338000	-1.232173000	1.796725000
C	4.573749000	-2.421920000	2.416871000
C	3.725120000	-3.337420000	1.770625000
C	3.300029000	-3.063270000	0.458860000
C	3.675351000	-1.883082000	-0.188153000
C	3.209513000	-4.542287000	2.513116000
S	4.828795000	0.653222000	-0.259701000
O	6.263418000	1.250307000	0.245268000
O	4.547354000	0.551859000	-1.880189000
C	-5.489670000	1.749136000	2.164330000
F	-5.474924000	0.640127000	2.990715000
F	-5.579484000	2.874361000	2.947957000
F	-6.624832000	1.678183000	1.372569000
H	2.255922000	6.496457000	-1.094747000
H	4.546131000	5.958428000	-0.269084000
H	5.086112000	3.637680000	0.468411000
H	0.574154000	4.672092000	-1.322275000
H	2.793912000	1.001992000	1.002024000
H	2.375163000	0.683972000	-1.693531000
H	1.383698000	0.177431000	-0.296961000
H	-0.492133000	-1.549710000	-4.117522000
H	1.098575000	-2.293620000	-4.473905000
H	-0.442750000	-3.973608000	-3.370430000
H	0.943594000	-3.608494000	-2.323519000
H	-0.641760000	-2.884898000	-1.985557000
C	-3.127162000	-1.260170000	-1.235566000
H	5.585945000	-0.506242000	2.294277000
H	4.900163000	-2.622485000	3.430848000
H	2.651013000	-3.768834000	-0.049359000
H	3.350930000	-1.653372000	-1.196208000
H	3.980589000	-4.983230000	3.152783000
H	2.375174000	-4.233924000	3.154206000
H	2.842679000	-5.313792000	1.829740000
C	1.268057000	-0.985706000	2.182735000
O	1.482582000	0.051696000	1.440408000
O	1.754905000	-1.323527000	3.278229000
C	0.168466000	-1.908811000	1.600845000
F	-1.108220000	-1.491835000	2.014565000
F	0.118819000	-1.894744000	0.189797000

F	0.292592000	-3.235529000	1.962049000
C	-3.584535000	-1.429718000	-2.542472000
C	-4.063912000	-2.689522000	-2.917411000
C	-4.082261000	-3.739261000	-1.990970000
C	-3.621508000	-3.537187000	-0.685499000
C	-3.129086000	-2.286091000	-0.293169000
H	-3.565087000	-0.613493000	-3.253531000
H	-4.427623000	-2.843354000	-3.927194000
H	-4.457458000	-4.712732000	-2.286426000
H	-3.636614000	-4.348516000	0.033196000
H	-2.757227000	-2.129477000	0.708988000

Int2

C	-2.571409000	-4.130622000	-2.420999000
C	-3.462112000	-3.858467000	-1.374307000
C	-3.011643000	-3.189185000	-0.237710000
C	-1.666261000	-2.803050000	-0.127967000
C	-0.760807000	-3.076720000	-1.183144000
C	-1.236901000	-3.731248000	-2.326060000
C	0.651419000	-2.577031000	-1.168388000
O	1.048361000	-1.914473000	-2.186115000
C	1.458496000	-2.839461000	-0.021985000
C	2.705610000	-2.261662000	0.186616000
O	3.240880000	-1.239668000	-0.456938000
O	3.461675000	-2.780911000	1.195788000
I	1.925423000	0.444832000	-1.126668000
O	0.820257000	2.341465000	-1.110389000
C	3.039554000	1.424217000	0.410905000
C	4.416543000	1.583868000	0.257527000
C	5.132844000	2.193110000	1.293876000
C	4.466317000	2.630218000	2.445621000
C	3.083331000	2.457934000	2.568873000
C	2.348357000	1.843313000	1.546098000
C	-0.225497000	2.374347000	-1.934882000
O	-0.345569000	1.779992000	-3.008562000
C	4.694189000	-2.061007000	1.602472000
C	5.223024000	-2.793047000	2.819636000
N	-1.302789000	-2.065337000	1.037894000
C	-1.312558000	3.314726000	-1.407993000
F	-1.488073000	3.210321000	-0.036749000
F	-2.537312000	3.045017000	-1.997351000
F	-0.999557000	4.639942000	-1.685414000
C	-3.243451000	0.092525000	1.288568000
C	-3.719761000	-0.473871000	2.466145000
C	-5.053022000	-0.239141000	2.808044000

C	-5.882946000	0.549664000	1.989527000
C	-5.348707000	1.105811000	0.814255000
C	-4.018664000	0.883273000	0.448035000
C	-7.330222000	0.774328000	2.355296000
S	-1.478536000	-0.176936000	0.819833000
O	-0.409904000	0.408547000	1.920200000
O	-1.337295000	0.280373000	-0.745916000
H	-2.919614000	-4.640302000	-3.312706000
H	-4.500670000	-4.161621000	-1.448408000
H	-3.681162000	-2.953769000	0.580964000
H	-0.548789000	-3.901255000	-3.145759000
H	1.157218000	-3.608427000	0.678290000
H	4.922908000	1.235915000	-0.634136000
H	6.204220000	2.330149000	1.195139000
H	5.025728000	3.104998000	3.244608000
H	2.567085000	2.793793000	3.461284000
H	1.279092000	1.688393000	1.637832000
H	4.434864000	-1.020704000	1.815250000
H	5.394196000	-2.077405000	0.763465000
H	6.142958000	-2.312952000	3.171195000
H	4.489909000	-2.774685000	3.631356000
H	5.445877000	-3.836561000	2.578962000
H	-0.303079000	-2.122149000	1.268307000
H	-3.075305000	-1.087036000	3.083558000
H	-5.453721000	-0.669343000	3.719967000
H	-5.977034000	1.720909000	0.178881000
H	-3.595992000	1.303949000	-0.455533000
H	-7.971370000	0.004732000	1.905321000
H	-7.480139000	0.733203000	3.438446000
H	-7.685636000	1.745326000	1.997227000

TS1

C	2.504549000	5.464781000	-0.937019000
C	3.802620000	5.212051000	-0.471761000
C	4.142972000	3.943233000	-0.007557000
C	3.207868000	2.895852000	-0.041444000
C	1.920743000	3.119541000	-0.597214000
C	1.574694000	4.430070000	-0.984730000
N	3.585057000	1.664635000	0.533789000
C	0.900919000	2.070856000	-0.869718000
O	-0.326127000	2.403928000	-0.883069000
C	1.331144000	0.705788000	-1.189182000
C	0.505038000	-0.126117000	-1.998415000
O	-0.784801000	-0.107394000	-2.129344000
O	1.167046000	-1.073578000	-2.696653000

C	0.406567000	-2.054663000	-3.528641000
C	-0.093026000	-3.217076000	-2.688287000
I	-2.329776000	0.681338000	-0.689302000
O	-4.166861000	0.691354000	0.516872000
C	-4.279750000	1.767924000	1.290237000
O	-3.481299000	2.710455000	1.340469000
C	4.518231000	-0.955407000	0.512513000
C	4.997934000	-1.211551000	1.791213000
C	4.626126000	-2.413181000	2.396245000
C	3.786530000	-3.328027000	1.736582000
C	3.349331000	-3.032229000	0.432854000
C	3.716116000	-1.840043000	-0.198827000
C	3.310153000	-4.568690000	2.447791000
S	4.894285000	0.690031000	-0.260372000
O	6.349723000	1.247728000	0.222110000
O	4.571666000	0.592385000	-1.869703000
C	-5.552842000	1.708544000	2.137230000
F	-5.559245000	0.574082000	2.932505000
F	-5.654524000	2.808712000	2.955850000
F	-6.676410000	1.666040000	1.327254000
H	2.224722000	6.459453000	-1.265252000
H	4.536957000	6.009533000	-0.442424000
H	5.120264000	3.737785000	0.412779000
H	0.571208000	4.600283000	-1.354959000
H	2.905395000	1.071382000	1.022087000
H	2.379363000	0.605843000	-1.461253000
H	1.287348000	0.127704000	0.142030000
H	-0.409659000	-1.521576000	-4.017766000
H	1.151882000	-2.359180000	-4.262799000
H	-0.541655000	-3.969873000	-3.346265000
H	0.732108000	-3.684362000	-2.143001000
H	-0.849447000	-2.894700000	-1.971893000
C	-3.146009000	-1.211573000	-1.277156000
H	5.634202000	-0.493389000	2.294511000
H	4.967010000	-2.631390000	3.402119000
H	2.711614000	-3.738032000	-0.089329000
H	3.386249000	-1.601248000	-1.203195000
H	4.103542000	-5.011521000	3.058060000
H	2.478140000	-4.313965000	3.115162000
H	2.953394000	-5.325590000	1.743328000
C	1.231928000	-1.042895000	2.085797000
O	1.462249000	0.009452000	1.315686000
O	1.726005000	-1.269686000	3.190511000
C	0.161433000	-2.004682000	1.549087000
F	-1.101897000	-1.626169000	2.007927000
F	0.087447000	-1.996760000	0.151210000

F	0.372740000	-3.308055000	1.936170000
C	-3.502263000	-1.396458000	-2.612491000
C	-3.999701000	-2.645218000	-3.001612000
C	-4.135328000	-3.672688000	-2.060576000
C	-3.774748000	-3.458199000	-0.725863000
C	-3.268247000	-2.217008000	-0.320635000
H	-3.388199000	-0.599357000	-3.336102000
H	-4.284973000	-2.807929000	-4.035006000
H	-4.523570000	-4.637691000	-2.366874000
H	-3.881886000	-4.251842000	0.004881000
H	-2.980027000	-2.049747000	0.707349000

Int3

C	2.596899000	-3.653997000	1.562721000
C	2.379549000	-4.251006000	0.313603000
C	1.696888000	-3.554651000	-0.686297000
C	1.188583000	-2.273998000	-0.425136000
C	1.347438000	-1.697025000	0.854725000
C	2.088568000	-2.381001000	1.829199000
C	0.698355000	-0.415354000	1.241332000
C	-0.755431000	-0.247784000	0.836214000
O	1.244139000	0.386584000	2.021123000
C	-1.656601000	-0.109592000	2.051447000
O	-2.008950000	0.942771000	2.587241000
N	0.630464000	-1.446720000	-1.434544000
I	-1.238412000	1.430676000	-0.519642000
C	0.316478000	2.784613000	0.112998000
O	-3.007117000	-0.405172000	-0.559010000
C	-4.043474000	-0.020977000	-1.241217000
O	-4.129304000	0.956996000	-2.017611000
C	1.208805000	3.273221000	-0.845735000
C	2.164186000	4.221421000	-0.453577000
C	2.222115000	4.652273000	0.876465000
C	1.318709000	4.148565000	1.821165000
C	0.348861000	3.214411000	1.442245000
O	-1.981599000	-1.356833000	2.476357000
C	-4.417977000	-1.401506000	2.749971000
C	-3.085984000	-1.480800000	3.475662000
C	3.405021000	-0.437620000	-1.222568000
C	4.338827000	-1.466892000	-1.159655000
C	5.292604000	-1.414171000	-0.142400000
C	5.308658000	-0.355599000	0.785329000
C	4.352601000	0.667582000	0.666478000
C	3.390381000	0.636704000	-0.343662000
C	6.347627000	-0.308893000	1.878533000
S	2.050691000	-0.581632000	-2.479071000

O	2.504210000	-1.667647000	-3.617479000
O	1.505187000	0.892790000	-2.902109000
C	-5.308726000	-0.849318000	-0.962274000
F	-6.191819000	-0.850937000	-2.024069000
F	-5.992562000	-0.324062000	0.141626000
F	-5.028389000	-2.177805000	-0.655744000
H	3.151738000	-4.184305000	2.328347000
H	2.766470000	-5.243373000	0.110676000
H	1.593719000	-3.972179000	-1.682223000
H	2.229263000	-1.914502000	2.796991000
H	-1.112506000	-1.063284000	0.219573000
H	0.136908000	-1.956835000	-2.175160000
H	1.186956000	2.900919000	-1.863456000
H	2.862918000	4.609212000	-1.187016000
H	2.966494000	5.382507000	1.176106000
H	1.364192000	4.482374000	2.852069000
H	-0.353065000	2.815649000	2.164671000
H	-5.236084000	-1.587526000	3.455232000
H	-4.567676000	-0.415445000	2.304982000
H	-4.469531000	-2.140701000	1.947309000
H	-2.902276000	-2.455646000	3.927081000
H	-2.961896000	-0.688278000	4.216999000
H	4.310679000	-2.274575000	-1.879951000
H	6.029737000	-2.206183000	-0.062584000
H	4.339899000	1.485464000	1.377913000
H	2.646803000	1.416254000	-0.421754000
H	5.948419000	0.153384000	2.786583000
H	6.707075000	-1.310669000	2.132290000
H	7.217933000	0.282746000	1.565463000

TS2

C	-2.595815000	-1.066394000	4.227276000
C	-3.719940000	-1.399517000	3.454024000
C	-3.665749000	-1.376767000	2.056999000
C	-2.466180000	-0.993055000	1.443552000
C	-1.348288000	-0.621912000	2.212872000
C	-1.406631000	-0.673495000	3.610833000
C	-0.142022000	-0.149190000	1.514307000
O	0.961923000	-0.098635000	2.083517000
C	-0.322816000	0.290912000	0.059989000
C	-1.287821000	1.385860000	-0.254966000
O	-1.754568000	2.157246000	0.599036000
O	-1.515916000	1.462409000	-1.583960000
I	1.827062000	1.361768000	-0.489382000
O	4.535416000	1.661967000	-1.333199000

C	2.164890000	2.503427000	1.313657000
C	3.389664000	2.351742000	1.954101000
C	3.592043000	3.088770000	3.129784000
C	2.589025000	3.924716000	3.632692000
C	1.364931000	4.036001000	2.963575000
C	1.137849000	3.321357000	1.780293000
C	5.170293000	0.705688000	-0.777272000
O	5.249706000	0.387141000	0.450376000
C	-2.538575000	2.438992000	-2.065920000
C	-2.977841000	1.961342000	-3.432417000
N	-2.264556000	-0.973490000	0.044719000
C	5.852799000	-0.258851000	-1.772657000
F	4.882889000	-1.065386000	-2.406819000
F	6.747164000	-1.140238000	-1.179694000
F	6.539944000	0.389962000	-2.788894000
C	-0.101461000	-2.909999000	-0.348165000
C	0.342589000	-3.292413000	0.911755000
C	1.717563000	-3.246919000	1.161674000
C	2.626428000	-2.817258000	0.178912000
C	2.129827000	-2.470077000	-1.095369000
C	0.761586000	-2.517580000	-1.369818000
C	4.095793000	-2.692880000	0.481456000
S	-1.926413000	-2.763339000	-0.640217000
O	-2.676337000	-3.851907000	0.324478000
O	-2.188985000	-2.716009000	-2.250572000
H	-2.656478000	-1.098951000	5.310065000
H	-4.644977000	-1.684547000	3.943445000
H	-4.531154000	-1.609609000	1.446194000
H	-0.533907000	-0.384714000	4.184636000
H	-0.153549000	-0.423048000	-0.724564000
H	4.153878000	1.683040000	1.558144000
H	4.538777000	2.993728000	3.651157000
H	2.757508000	4.484685000	4.547155000
H	0.578794000	4.673234000	3.354709000
H	0.181651000	3.386433000	1.276167000
H	-2.055033000	3.421428000	-2.079355000
H	-3.361983000	2.432628000	-1.354381000
H	-2.133826000	1.904483000	-4.127351000
H	-3.445136000	0.980190000	-3.333093000
H	-3.722753000	2.656489000	-3.836516000
H	-3.123946000	-0.616871000	-0.523773000
H	-0.366132000	-3.608038000	1.667629000
H	2.088942000	-3.522495000	2.142718000
H	2.825267000	-2.144839000	-1.860743000
H	0.361273000	-2.255628000	-2.343564000
H	4.366748000	-1.650185000	0.698581000

H	4.375734000	-3.300197000	1.347798000
H	4.708470000	-2.998169000	-0.372018000
C	-6.361712000	1.120418000	-1.520204000
F	-5.904329000	2.425732000	-1.283313000
F	-6.313434000	0.940374000	-2.902484000
F	-7.696722000	1.097309000	-1.156152000
C	-5.496083000	0.086661000	-0.771555000
O	-4.265820000	0.136524000	-1.154076000
O	-6.043815000	-0.641512000	0.091199000

Int4

C	1.421138000	3.902044000	1.142395000
C	0.327391000	3.207487000	1.694476000
C	-0.085608000	1.964560000	1.204997000
C	0.635665000	1.420074000	0.139167000
C	1.746302000	2.098196000	-0.405348000
C	2.138904000	3.347039000	0.082500000
N	0.392411000	0.234329000	-0.581186000
C	1.515141000	-0.053118000	-1.500238000
C	2.322039000	1.291005000	-1.476426000
O	3.259098000	1.550812000	-2.238293000
C	2.433028000	-1.187708000	-1.055134000
O	2.511949000	-1.213766000	0.303854000
O	3.052892000	-1.915230000	-1.824833000
C	3.190256000	-2.372042000	0.939958000
C	3.096071000	-2.146116000	2.434580000
C	-2.196004000	-0.697627000	0.018485000
C	-2.636729000	-0.440873000	-1.274976000
C	-3.975872000	-0.089885000	-1.449649000
C	-4.853272000	-0.003550000	-0.351572000
C	-4.361255000	-0.285051000	0.933841000
C	-3.023289000	-0.640293000	1.132232000
C	-6.292768000	0.401671000	-0.556725000
S	-0.426602000	-1.196517000	0.275147000
O	-0.194744000	-1.248858000	1.893435000
O	-0.027016000	-2.476199000	-0.660125000
H	1.705083000	4.865991000	1.548170000
H	-0.213225000	3.646831000	2.526097000
H	-0.917863000	1.441059000	1.654176000
H	2.988730000	3.854532000	-0.359774000
H	1.162001000	-0.278613000	-2.505680000
H	2.657254000	-3.268441000	0.611649000
H	4.216971000	-2.408183000	0.566096000
H	3.543450000	-2.993070000	2.966794000
H	3.628694000	-1.234846000	2.723806000
H	2.048677000	-2.047253000	2.730370000

H	-1.957320000	-0.505362000	-2.115508000
H	-4.346252000	0.118076000	-2.448041000
H	-5.030007000	-0.231352000	1.786306000
H	-2.625188000	-0.866291000	2.114090000
H	-6.907874000	0.144267000	0.310065000
H	-6.376163000	1.485024000	-0.713058000
H	-6.722480000	-0.087344000	-1.437274000

TS3

C	-5.104702000	1.029914000	-1.428421000
C	-5.485787000	-0.313692000	-1.192830000
C	-4.572203000	-1.284689000	-0.789428000
C	-3.242387000	-0.879747000	-0.616098000
C	-2.847952000	0.456713000	-0.875914000
C	-3.782901000	1.427416000	-1.271280000
C	-1.435524000	0.537779000	-0.642126000
C	-0.949069000	-0.801990000	-0.261240000
O	-0.751229000	1.635189000	-0.756145000
C	-0.061372000	-0.835457000	0.931678000
O	0.669810000	0.127573000	1.252852000
N	-2.141481000	-1.647830000	-0.196479000
H	-0.097981000	-1.097367000	-1.307240000
I	1.506650000	1.786189000	-0.810051000
O	3.559616000	2.243138000	-1.400994000
C	1.075563000	2.592938000	-2.734834000
C	0.784152000	1.690254000	-3.757804000
C	0.516243000	2.210967000	-5.029267000
C	0.549260000	3.592364000	-5.250808000
C	0.847958000	4.471279000	-4.202860000
C	1.112677000	3.973767000	-2.922224000
C	4.462398000	1.324115000	-1.054163000
O	4.289080000	0.375465000	-0.284736000
O	-0.109527000	-1.983717000	1.632139000
C	0.779112000	-2.090362000	2.831675000
C	2.225890000	-2.348552000	2.446947000
C	-0.488572000	-4.103609000	-1.082265000
C	0.012026000	-4.609746000	0.109641000
C	1.287650000	-5.177894000	0.082251000
C	2.034516000	-5.228988000	-1.106600000
C	1.457894000	-4.747173000	-2.296142000
C	0.186921000	-4.174412000	-2.297463000
C	3.457859000	-5.722164000	-1.108763000
S	-2.187386000	-3.379544000	-1.119084000
O	-2.572782000	-3.087533000	-2.679743000
O	-3.209900000	-4.248368000	-0.196393000
C	5.796092000	1.588744000	-1.756305000

F	5.646421000	1.540269000	-3.131478000
F	6.739255000	0.654377000	-1.393459000
F	6.285950000	2.843858000	-1.426495000
O	2.174016000	-2.041193000	-0.628325000
C	1.900105000	-1.678399000	-1.779372000
O	0.754546000	-1.184510000	-2.214309000
C	2.961022000	-1.716111000	-2.879659000
F	4.071309000	-2.441763000	-2.508707000
F	3.381503000	-0.414862000	-3.179612000
F	2.473529000	-2.263131000	-4.058831000
H	-5.855824000	1.749195000	-1.733517000
H	-6.524719000	-0.595064000	-1.325703000
H	-4.868611000	-2.309390000	-0.605428000
H	-3.467438000	2.449565000	-1.443669000
H	0.765192000	0.621708000	-3.576425000
H	0.288447000	1.530988000	-5.842253000
H	0.344512000	3.985063000	-6.240690000
H	0.876761000	5.541170000	-4.376091000
H	1.349016000	4.645316000	-2.106549000
H	0.667617000	-1.165111000	3.401563000
H	0.330619000	-2.921012000	3.377337000
H	2.812237000	-2.500522000	3.361343000
H	2.312758000	-3.235707000	1.817714000
H	2.646326000	-1.508651000	1.892649000
H	-0.564096000	-4.548194000	1.022688000
H	1.716369000	-5.568022000	0.998858000
H	2.019553000	-4.792804000	-3.222345000
H	-0.263235000	-3.773931000	-3.196152000
H	4.137910000	-4.863451000	-1.176022000
H	3.698388000	-6.273965000	-0.196013000
H	3.659951000	-6.369682000	-1.968535000

Int5

C	-2.352289000	4.398177000	-2.341888000
C	-3.608071000	3.768490000	-2.246314000
C	-3.765740000	2.544539000	-1.584539000
C	-2.627525000	1.977023000	-1.017995000
C	-1.368311000	2.617294000	-1.081761000
C	-1.215481000	3.825808000	-1.762313000
N	-2.480516000	0.728107000	-0.348183000
C	-1.127725000	0.647678000	0.086730000
C	-0.427505000	1.769022000	-0.361256000
C	-0.580495000	-0.502828000	0.769348000
O	0.875239000	2.099120000	-0.259510000
O	0.531951000	-0.503649000	1.331389000
I	2.520422000	0.768680000	-0.776775000

O	4.375823000	-0.372442000	-1.158627000
C	4.324252000	-1.701511000	-1.264977000
O	3.327891000	-2.422280000	-1.331136000
O	-1.378530000	-1.616634000	0.699790000
C	-2.241884000	-3.229104000	2.304113000
C	-1.006670000	-2.784239000	1.540951000
C	-4.907243000	-0.751917000	0.365041000
C	-4.329572000	-1.824228000	-0.304696000
C	-5.186907000	-2.776189000	-0.862905000
C	-6.583907000	-2.657736000	-0.748213000
C	-7.116541000	-1.548646000	-0.068142000
C	-6.281770000	-0.581192000	0.496876000
C	-7.493563000	-3.712767000	-1.329878000
S	-3.827288000	0.549539000	1.137788000
O	-4.752299000	1.892599000	1.266718000
O	-3.019314000	0.061413000	2.472424000
C	3.317012000	1.086622000	1.250358000
C	4.640520000	0.746475000	1.494430000
C	5.123729000	0.970016000	2.792496000
C	4.294574000	1.518554000	3.775688000
C	2.969364000	1.848716000	3.474256000
C	2.454857000	1.632397000	2.188137000
C	5.749796000	-2.267420000	-1.306765000
F	6.429506000	-1.828603000	-2.431229000
F	5.747302000	-3.643333000	-1.317555000
F	6.471912000	-1.851698000	-0.191031000
H	-2.269106000	5.340450000	-2.872086000
H	-4.474141000	4.240045000	-2.697296000
H	-4.734591000	2.070497000	-1.503504000
H	-0.242759000	4.298658000	-1.831798000
H	-1.985533000	-4.068745000	2.960647000
H	-2.625886000	-2.403929000	2.908852000
H	-3.035569000	-3.554765000	1.624894000
H	-0.634129000	-3.547069000	0.852095000
H	-0.193885000	-2.471267000	2.198178000
H	-3.253714000	-1.899423000	-0.386670000
H	-4.764373000	-3.619292000	-1.399851000
H	-8.192595000	-1.438165000	0.016722000
H	-6.676880000	0.288860000	1.006619000
H	-8.474200000	-3.299698000	-1.584731000
H	-7.063933000	-4.156980000	-2.233176000
H	-7.656717000	-4.526537000	-0.610934000
H	5.271602000	0.314223000	0.731297000
H	6.151455000	0.709629000	3.019774000
H	4.679505000	1.686434000	4.775482000
H	2.319479000	2.267730000	4.234085000

H	1.431803000	1.869443000	1.942420000

Int6

C	-4.574040000	-3.794889000	-0.895165000
C	-4.739010000	-2.874510000	-1.948282000
C	-4.056796000	-1.652521000	-1.983948000
C	-3.186239000	-1.382664000	-0.927732000
C	-2.999131000	-2.302747000	0.123881000
C	-3.702938000	-3.509611000	0.156724000
N	-2.406803000	-0.218976000	-0.678731000
C	-1.510006000	-0.450714000	0.423632000
C	-2.050885000	-1.741484000	1.079391000
O	-1.713350000	-2.156139000	2.199196000
I	0.659436000	-1.034916000	-0.356318000
O	3.010437000	-1.311272000	-0.982232000
C	-1.370575000	0.712613000	1.382419000
O	-0.315686000	1.213715000	1.772407000
C	3.613319000	-0.164390000	-1.100924000
O	3.143698000	0.989861000	-0.945106000
C	-1.084135000	2.241550000	-1.299392000
C	0.301392000	2.350027000	-1.284777000
C	0.858506000	3.393794000	-0.545128000
C	0.042852000	4.310615000	0.139258000
C	-1.357759000	4.182184000	0.050646000
C	-1.941661000	3.138620000	-0.668533000
C	0.660049000	5.397790000	0.982364000
S	-1.838571000	0.791125000	-2.162028000
O	-3.194479000	1.188417000	-2.970084000
O	-0.662645000	-0.079842000	-2.905367000
C	1.495913000	-1.279352000	1.598928000
C	1.295721000	-2.472130000	2.288259000
C	1.899486000	-2.614819000	3.544439000
C	2.687202000	-1.585696000	4.067608000
C	2.876891000	-0.401559000	3.343515000
C	2.274010000	-0.233440000	2.094082000
O	-2.604136000	1.103462000	1.802778000
C	-4.086758000	2.729752000	2.769936000
C	-2.666389000	2.205567000	2.799377000
C	5.110193000	-0.319763000	-1.418577000
F	5.794053000	-0.784016000	-0.296986000
F	5.689742000	0.880906000	-1.785268000
F	5.336555000	-1.222285000	-2.444712000
H	-5.128936000	-4.725683000	-0.904574000
H	-5.420805000	-3.112858000	-2.757372000
H	-4.201649000	-0.943764000	-2.787809000
H	-3.560667000	-4.196678000	0.983056000

H	0.943929000	1.644730000	-1.792672000
H	1.938797000	3.463795000	-0.494182000
H	-1.994229000	4.903895000	0.552192000
H	-3.016411000	3.019606000	-0.729215000
H	-0.031775000	6.231107000	1.136692000
H	0.934952000	5.004622000	1.969933000
H	1.573182000	5.788311000	0.523060000
H	0.684979000	-3.265562000	1.881042000
H	1.751798000	-3.532231000	4.102829000
H	3.157636000	-1.706299000	5.037395000
H	3.493792000	0.393723000	3.746590000
H	2.409725000	0.673754000	1.521511000
H	-4.204886000	3.527780000	3.511105000
H	-4.329977000	3.134027000	1.782566000
H	-4.800688000	1.934150000	3.000131000
H	-2.387708000	1.782546000	3.768475000
H	-1.925539000	2.955999000	2.515598000

TS4

C	0.178471000	0.289906000	2.647379000
C	0.615902000	-1.039825000	2.732308000
C	1.709328000	-1.514320000	1.991491000
C	2.351102000	-0.605306000	1.155157000
C	1.914471000	0.726706000	1.055934000
C	0.831993000	1.187486000	1.797803000
N	3.484101000	-0.773229000	0.270683000
C	3.745209000	0.424286000	-0.388869000
C	2.736266000	1.448656000	0.077544000
O	2.627670000	2.617780000	-0.311216000
I	5.616238000	1.375326000	1.584739000
O	7.358696000	2.131328000	3.679671000
C	4.691751000	0.626210000	-1.516639000
O	5.173813000	1.713506000	-1.832632000
C	6.846701000	2.130942000	4.854875000
O	5.644363000	2.248069000	5.228311000
C	4.177777000	-3.066725000	-1.259214000
C	5.228798000	-3.805737000	-1.780675000
C	4.971916000	-4.584170000	-2.913795000
C	3.693972000	-4.609835000	-3.496993000
C	2.665822000	-3.842521000	-2.920959000
C	2.895856000	-3.060532000	-1.787229000
C	3.422751000	-5.422290000	-4.740649000
S	4.529651000	-2.104840000	0.295344000
O	4.090260000	-3.028430000	1.572737000

O	6.069172000	-1.546916000	0.307308000
C	4.996904000	3.425628000	1.657597000
C	4.649353000	3.979214000	2.889978000
C	4.241116000	5.318451000	2.912532000
C	4.180302000	6.066378000	1.729840000
C	4.530172000	5.479434000	0.509980000
C	4.947785000	4.143506000	0.460626000
O	4.938230000	-0.553569000	-2.147312000
C	5.138339000	-1.061132000	-4.522979000
C	5.880498000	-0.576500000	-3.290393000
C	7.913142000	1.999882000	5.970435000
F	8.764942000	0.915257000	5.766170000
F	7.370437000	1.839316000	7.239591000
F	8.727919000	3.135340000	6.032751000
C	2.051769000	0.149818000	-3.046287000
O	2.122606000	-0.097235000	-1.779975000
O	2.701669000	0.952722000	-3.753415000
C	1.035210000	-0.731260000	-3.811056000
F	0.345392000	-1.642515000	-3.007552000
F	1.680432000	-1.497064000	-4.789879000
F	0.074722000	0.025927000	-4.466419000
H	-0.665411000	0.619946000	3.242762000
H	0.104194000	-1.729372000	3.395218000
H	2.053638000	-2.531614000	2.085681000
H	0.521101000	2.221303000	1.702115000
H	6.215084000	-3.751641000	-1.335737000
H	5.775261000	-5.164703000	-3.355553000
H	1.685053000	-3.820504000	-3.379540000
H	2.125839000	-2.412418000	-1.390718000
H	2.650592000	-6.180141000	-4.559999000
H	3.062836000	-4.779913000	-5.553185000
H	4.324182000	-5.935727000	-5.088099000
H	4.734066000	3.394251000	3.803680000
H	3.977485000	5.772865000	3.862376000
H	3.859766000	7.103506000	1.759739000
H	4.473367000	6.049776000	-0.411242000
H	5.189811000	3.671758000	-0.482857000
H	5.838626000	-1.137795000	-5.364053000
H	4.696409000	-2.045813000	-4.348212000
H	4.339533000	-0.362870000	-4.781072000
H	6.277659000	0.432660000	-3.410426000
H	6.674123000	-1.257559000	-2.973838000

Int7

C	-4.329936000	2.362111000	1.494098000
C	-3.975203000	1.361648000	2.417428000
C	-2.885853000	0.505701000	2.212085000
C	-2.156536000	0.669686000	1.033749000
C	-2.517638000	1.657431000	0.091675000
C	-3.591545000	2.520020000	0.321585000
N	-0.985397000	-0.011277000	0.603546000
C	-0.652983000	0.373776000	-0.762817000
C	-1.595313000	1.619673000	-1.024169000
O	-1.474577000	2.346644000	-2.011137000
O	0.751812000	0.709621000	-0.936722000
C	-0.935245000	-0.726929000	-1.797161000
O	-0.095448000	-1.310564000	-2.474092000
C	1.259785000	1.763137000	-0.245471000
O	0.662481000	2.478583000	0.546284000
O	-2.269847000	-0.939872000	-1.805120000
C	-4.005557000	-2.603889000	-1.699712000
C	-2.797265000	-2.152126000	-2.493352000
C	1.255509000	-1.645497000	0.989487000
C	1.946230000	-0.868711000	1.913866000
C	3.316789000	-0.703154000	1.716217000
C	3.967447000	-1.299784000	0.618491000
C	3.221934000	-2.093717000	-0.268332000
C	1.847143000	-2.274986000	-0.095833000
C	5.428817000	-1.030169000	0.366997000
S	-0.580591000	-1.751087000	1.186396000
O	-1.177471000	-2.895279000	0.182420000
O	-1.000651000	-1.777168000	2.760529000
C	2.723279000	2.005365000	-0.615743000
F	3.288397000	0.986034000	-1.351187000
F	3.477488000	2.166192000	0.536704000
F	2.823184000	3.173470000	-1.350509000
H	-5.175045000	3.008957000	1.698016000
H	-4.556496000	1.249991000	3.326471000
H	-2.615605000	-0.251264000	2.933486000
H	-3.833947000	3.283455000	-0.408704000
H	-4.459918000	-3.476185000	-2.182847000
H	-3.699250000	-2.881944000	-0.688387000
H	-4.756009000	-1.809785000	-1.642614000
H	-3.037029000	-1.851980000	-3.516571000
H	-1.997554000	-2.894331000	-2.504875000
H	1.431844000	-0.407660000	2.748313000
H	3.884807000	-0.090739000	2.407170000
H	3.713580000	-2.553542000	-1.118138000
H	1.255051000	-2.848580000	-0.796419000

H	5.886776000	-1.819669000	-0.235427000
H	5.541605000	-0.085601000	-0.180042000
H	5.989274000	-0.939297000	1.302509000

2a

C	4.243597000	-0.807426000	2.202574000
C	3.471031000	-1.950230000	1.930135000
C	2.402627000	-1.932720000	1.024239000
C	2.122817000	-0.723185000	0.384503000
C	2.899358000	0.429848000	0.643901000
C	3.957751000	0.394604000	1.553396000
N	1.101204000	-0.387887000	-0.522389000
C	1.255833000	0.985550000	-1.063889000
C	2.404308000	1.546875000	-0.148865000
O	2.772087000	2.722646000	-0.170623000
O	1.721032000	1.038907000	-2.392719000
C	-1.621598000	-1.194723000	-0.476713000
C	-2.579012000	-0.434830000	-1.132949000
C	-3.760399000	-0.147507000	-0.440280000
C	-3.965962000	-0.612598000	0.869569000
C	-2.965798000	-1.394687000	1.480475000
C	-1.778714000	-1.694183000	0.811017000
C	-5.224292000	-0.260188000	1.624516000
S	-0.017487000	-1.531966000	-1.357548000
O	0.477456000	-3.054588000	-1.056725000
O	-0.175048000	-0.971902000	-2.892389000
C	-0.052235000	1.755049000	-0.881649000
O	-0.743926000	2.192385000	-1.801407000
O	-0.351062000	1.875021000	0.439818000
C	-1.870691000	2.278149000	2.257162000
C	-1.587411000	2.609765000	0.806432000
H	5.061477000	-0.864939000	2.911107000
H	3.708966000	-2.882683000	2.431311000
H	1.838449000	-2.826762000	0.801431000
H	4.535318000	1.294063000	1.734896000
H	1.041749000	0.640192000	-2.989374000
H	-2.398413000	-0.072617000	-2.137216000
H	-4.529068000	0.443831000	-0.926220000
H	-3.120762000	-1.771687000	2.485969000
H	-1.007682000	-2.299268000	1.272773000
H	-5.577850000	-1.102343000	2.227979000
H	-5.044158000	0.578846000	2.309551000
H	-6.028898000	0.035110000	0.945384000
H	-2.767995000	2.810747000	2.591952000
H	-2.035925000	1.203919000	2.377549000
H	-1.033685000	2.575752000	2.895058000

H	-1.397148000	3.673973000	0.641802000
H	-2.384582000	2.285398000	0.134457000

7. References

1. Sardar, B., Pal, D., Sarmah, R., & Srimani, D. (**2023**). Ruthenium-catalyzed dehydrogenative cyclization to synthesize polysubstituted 4-quinolones under solvent-free conditions. Chemical Communications, 59(60), 9267‑9270.
2. Ren, L., Lian, X.-L., & Gong, L.-Z. (**2013**). Brønsted Acid/Rhodium(II) Cooperative Catalytic Asymmetric Three-Component Aldol-Type Reaction for the Synthesis of 3-Amino Oxindoles. Chemistry – A European Journal, 19(10), 3315–3318.
3. Kobayashi Yuta; Konishi Hisatoshi, K. O. (**2009**). Synthesis of 4-Alkylidene-4H-3,1-benzoxazine Derivatives by Acid-Catalyzed Cyclization of 2-Isocyanophenyl Ketones in the Presence of a Vinyl Ether. Synthesis, **2009** (09), 1494–1498.
4. Ikeda, A., Omote, M., Kusumoto, K., Komori, M., Tarui, A., Sato, K., & Ando, A. (2016). A dramatic enhancing effect of InBr3 towards the oxidative Sonogashira cross-coupling reaction of 2-ethynylanilines. Organic & Biomolecular Chemistry, 14(6), 2127–2133.
5. Ji, M., Wang, X., Liu, J., Wu, X., & Zhu, C. (**2021**). Catalyst-free, radical-mediated intermolecular 1,2-arylheteroarylation of alkenes by cleaving inert C-C bond. Science China Chemistry, 64(10), 1703–1708.
6. Devine, W., Woodring, J. L., Swaminathan, U., Amata, E., Patel, G., Erath, J., Roncal, N. E., Lee, P. J., Leed, S. E., Rodriguez, A., Mensa-Wilmot, K., Sciotti, R. J., & Pollastri, M. P. (**2015**). Protozoan Parasite Growth Inhibitors Discovered by Cross-Screening Yield Potent Scaffolds for Lead Discovery. Journal of Medicinal Chemistry, 58(14), 5522–5537.
7. Lázaro-Milla, C., & Almendros, P. (**2021**). A Convenient Formal [4+2] Heterocylization Route to Bis(triflyl)tetrahydroquinolines. Chemistry – A European Journal, 27(54), 13534–13538.
8. Jin, G. H., Ha, S. K., Park, H. M., Kang, B., Kim, S. Y., Kim, H.-D., Ryu, J.-H., & Jeon, R. (**2008**). Synthesis of azaisoflavones and their inhibitory activities of NO production in activated microglia. Bioorganic & Medicinal Chemistry Letters, 18(14), 4092–4094.

9. Tanaka Kosho; Tabata Hidetsugu; Oshitari Tetsuta; Natsugari Hideaki; Takahashi Hideyo, R. M. (**2021**). Atropisomeric Properties of 9-Methyl-1,4-benzodiazepin-2-ones. Synthesis, 53(24), 4682–4688.
10. Hoffmann La Roche AG. (**2006**). Preparation of 5-chloro-4- alkyl-3,4-dihydro-quinazolin-2-ylamines as 5-HT5A receptor modulators (Patent No. US20060293349).
11. Wen, S.-S., Zhou, Z.-F., Xiao, J.-A., Li, J., Xiang, H., & Yang, H. (**2017**). Facile oxidative cyclization to access C2-quaternary 2-hydroxy-indolin-3-ones: synthetic studies towards matemone. New Journal of Chemistry, 41(20), 11503–11506.

8. NMR Spectra

^{1}H NMR spectra of **S1(b)** and **S1(c)**

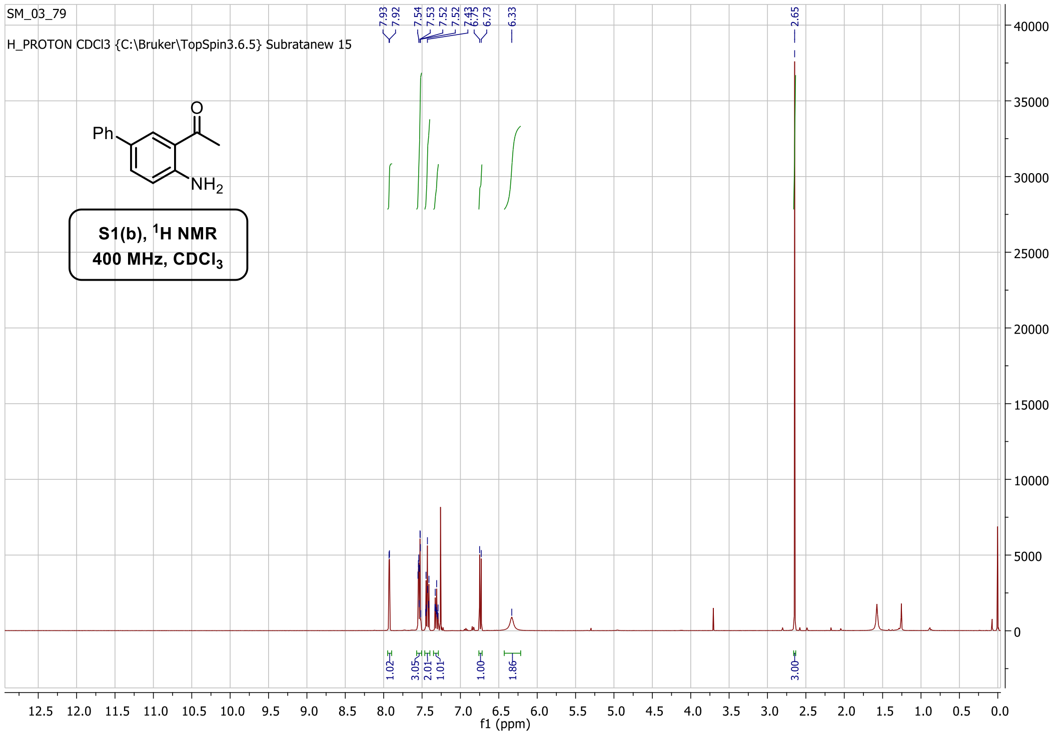

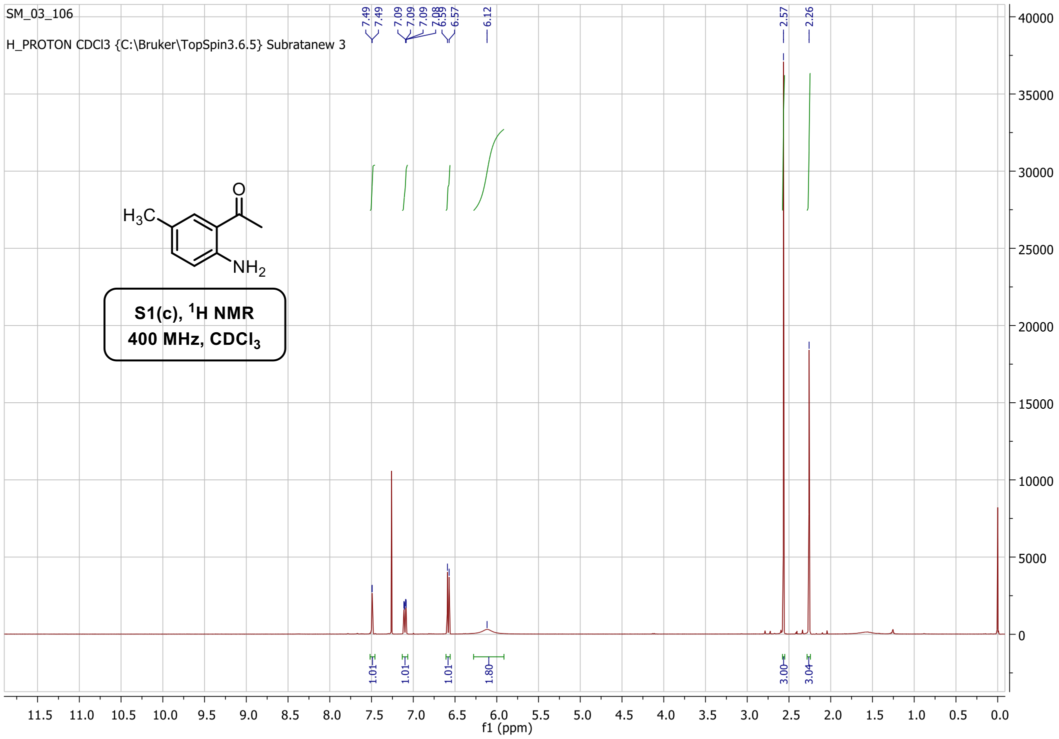

^{1}H NMR spectra of **S1(d)** and **S1(e)**

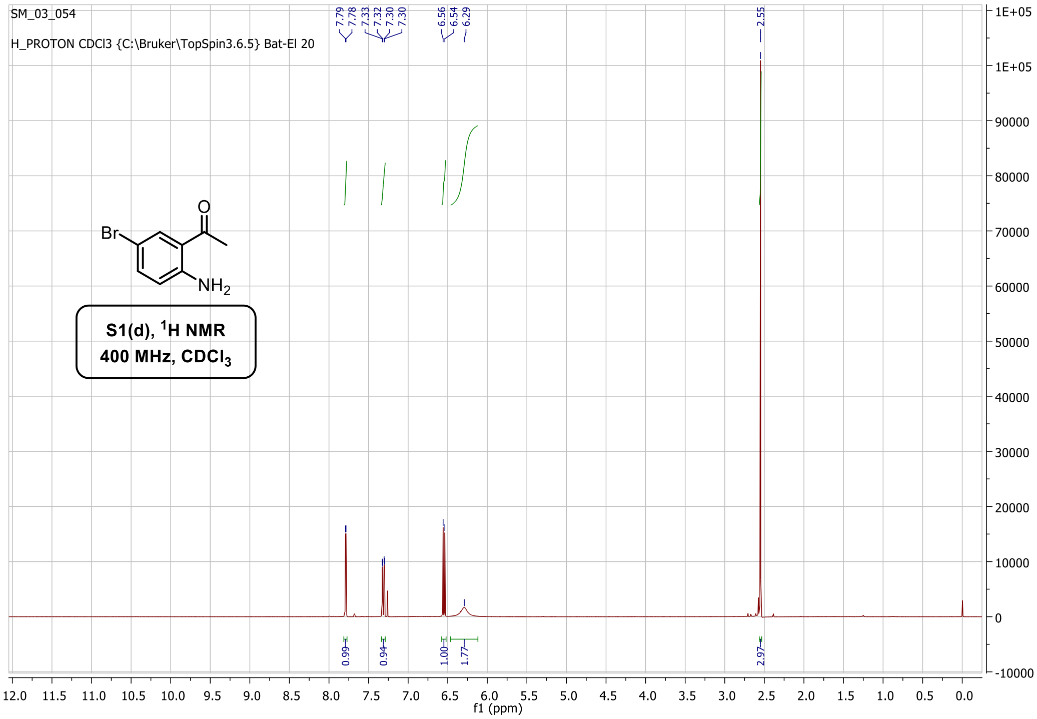

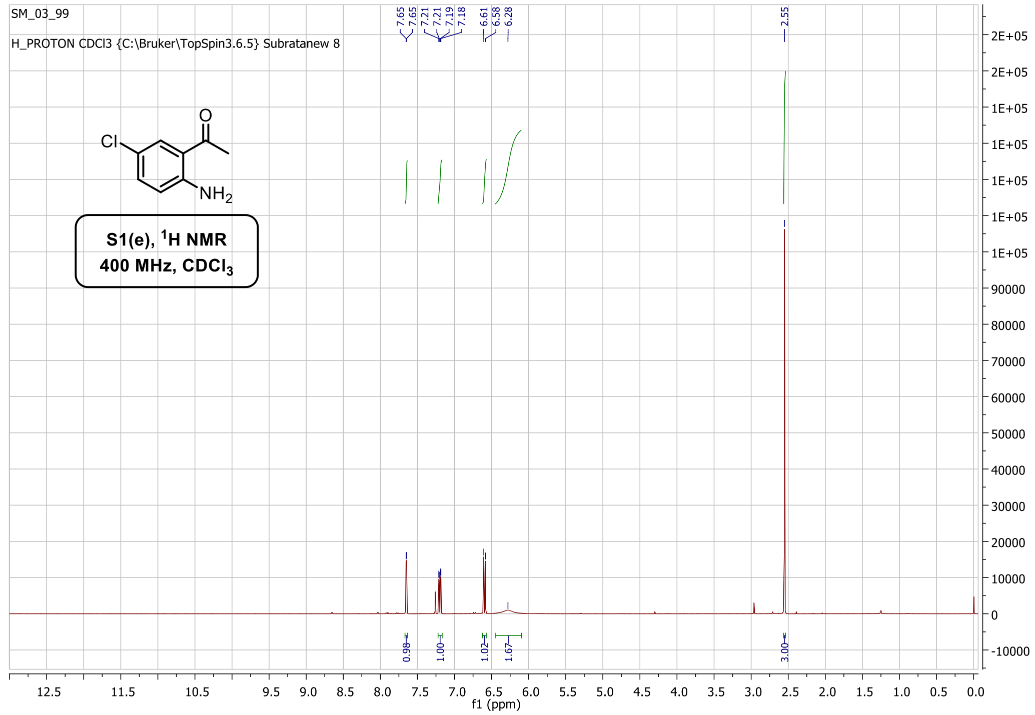

^{1}H NMR spectra of **S1(f)**

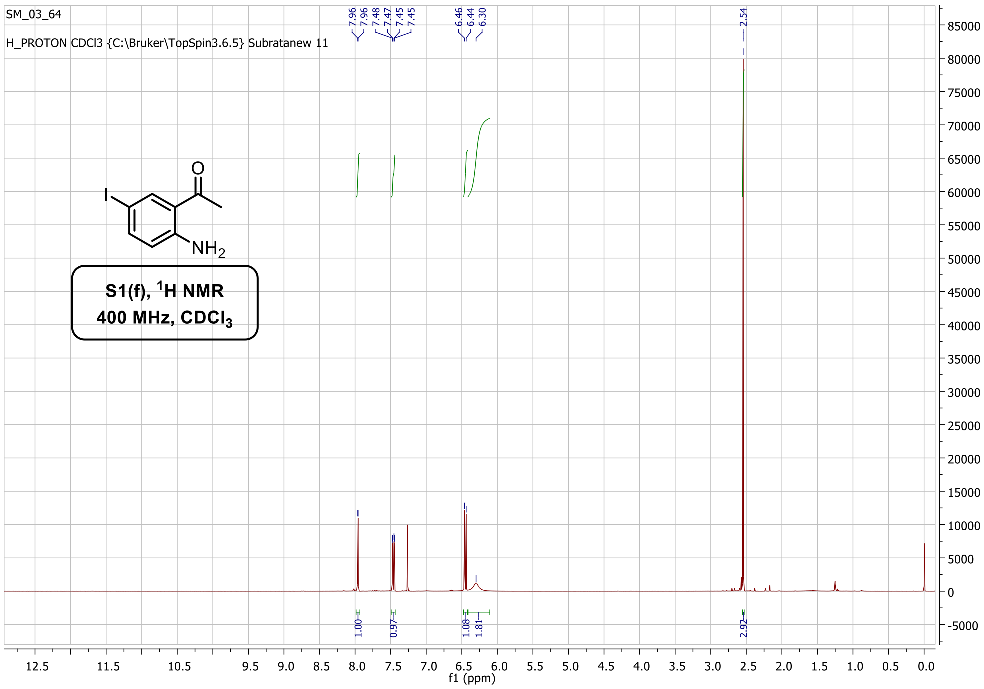

^{1}H and ^{19}F NMR spectra of **S1(g)**

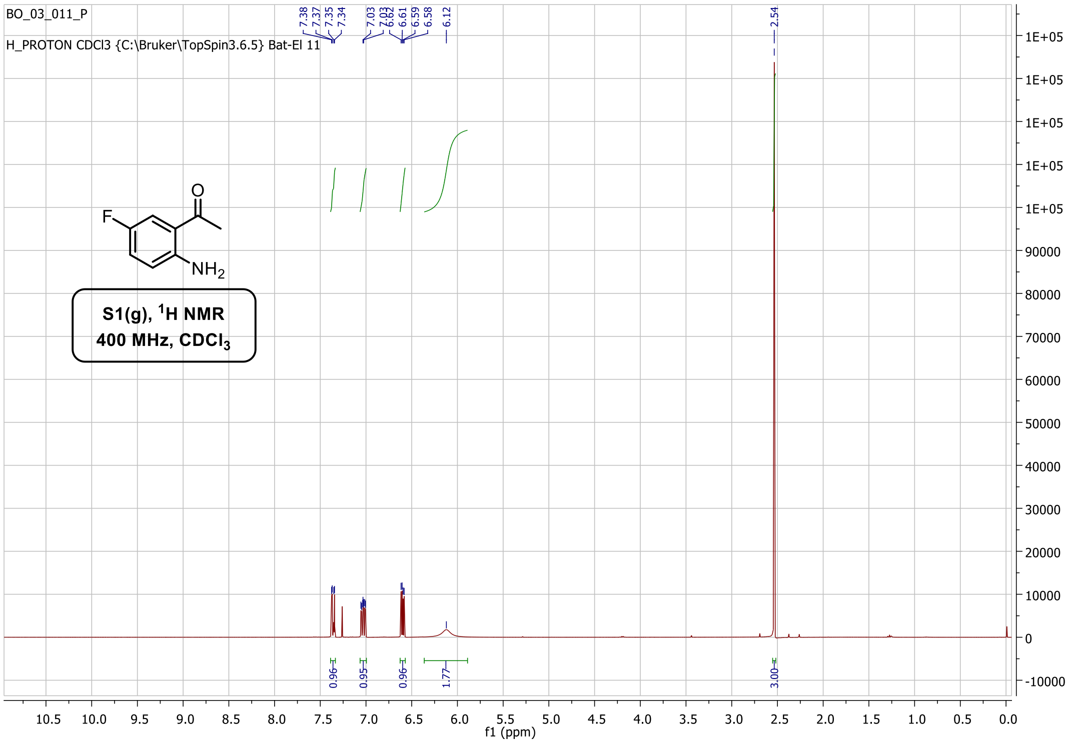

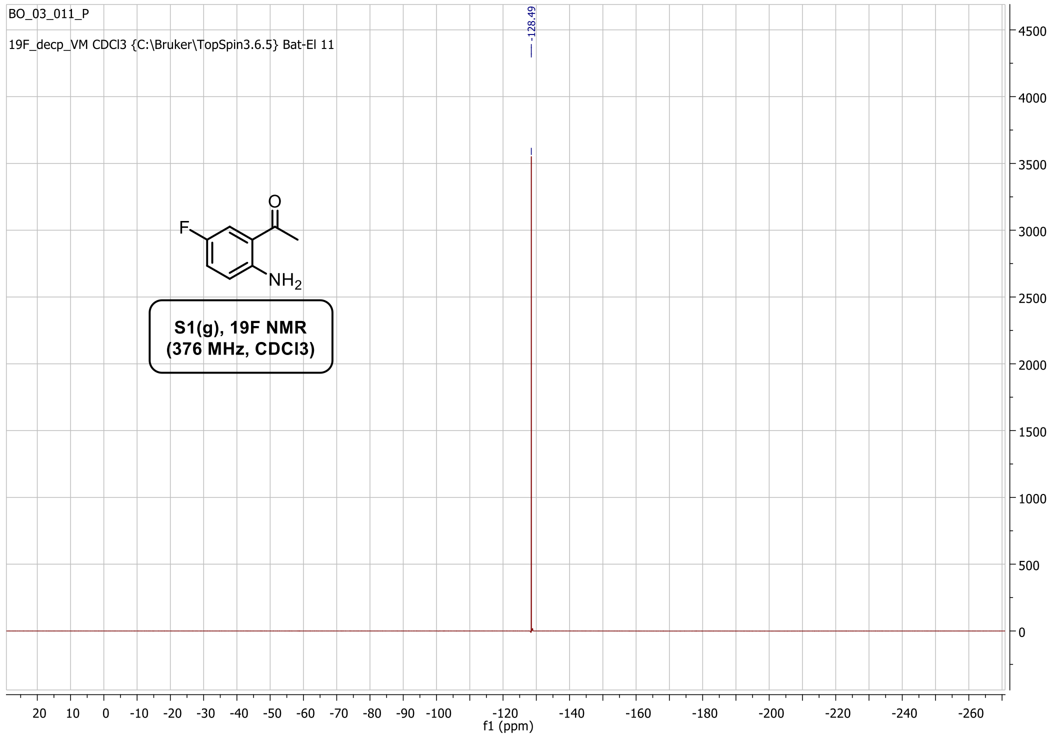

^{1}H NMR spectra of **S1(i)** and **S1(j)**

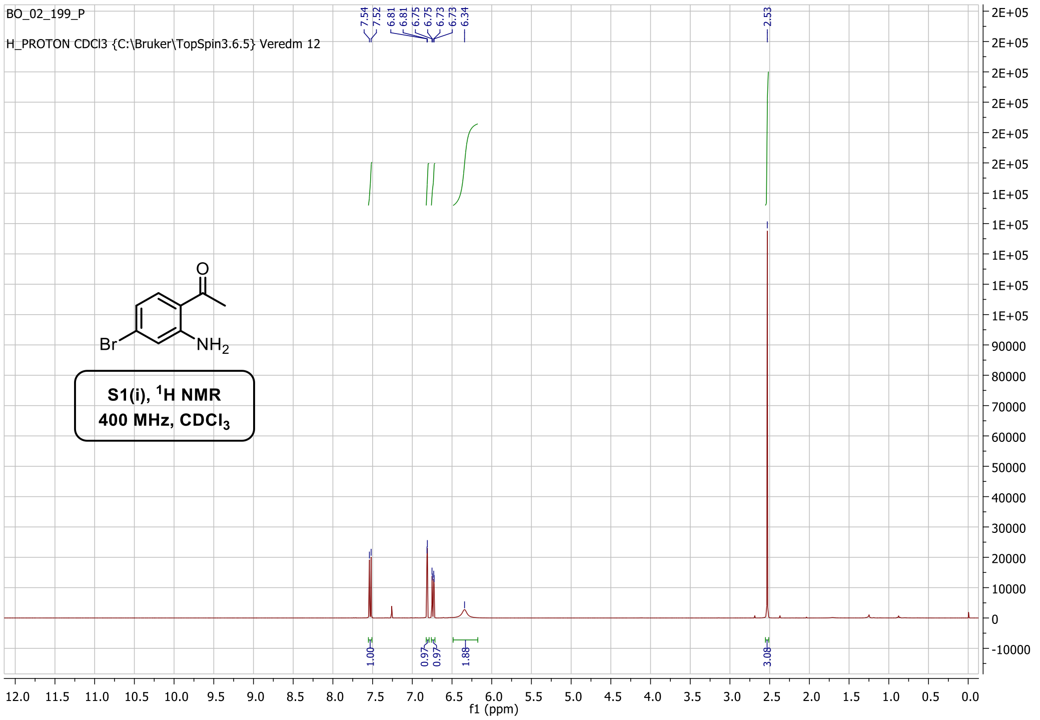

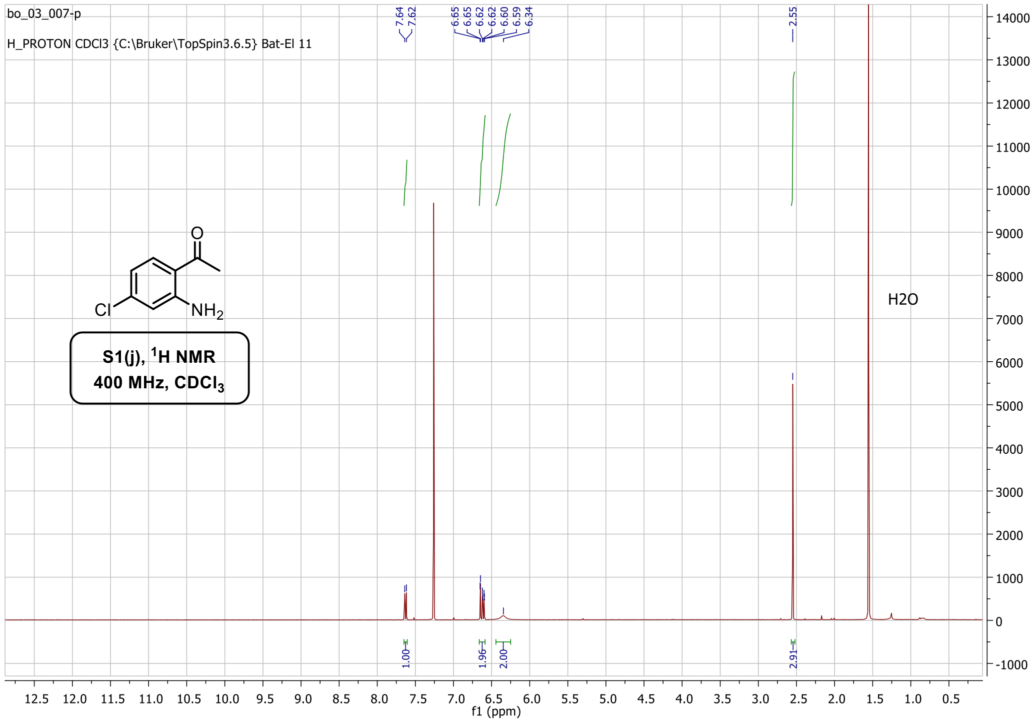

^{1}H and ^{19}F NMR spectra of **S1(k)**

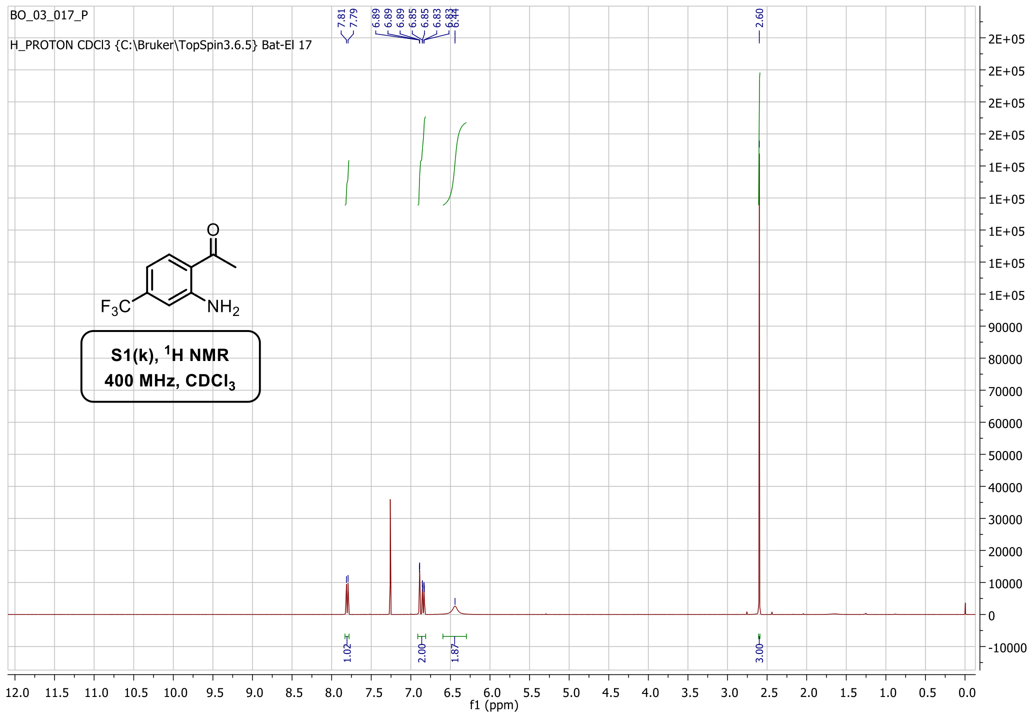

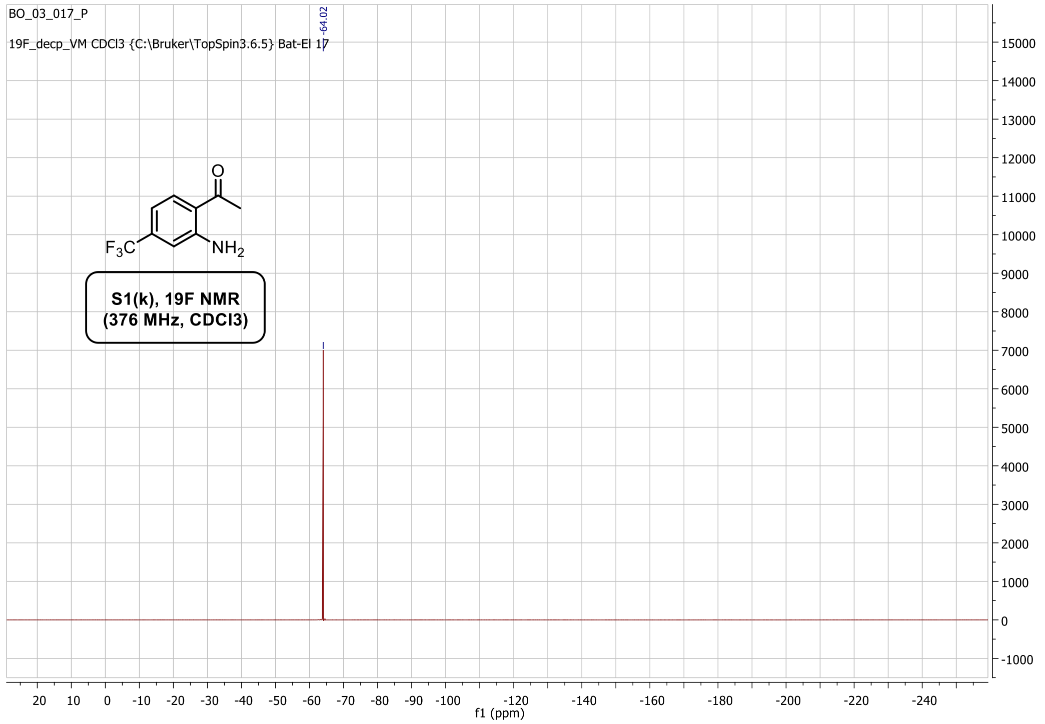

^{1}H NMR spectra of **S1(l)** and **S1(m)**

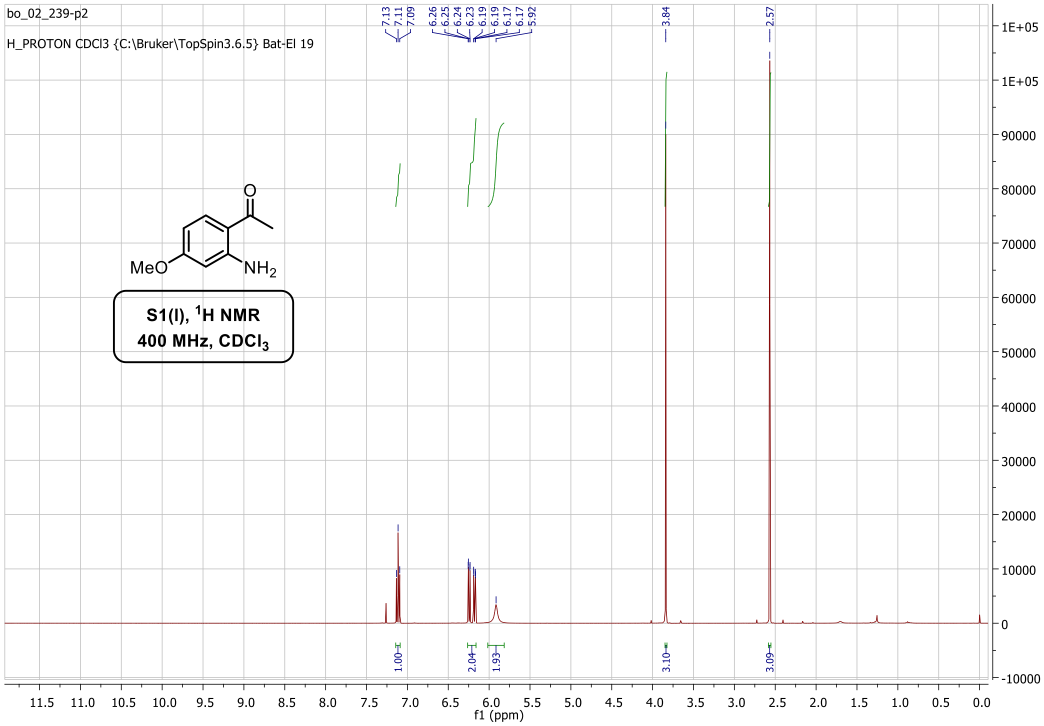

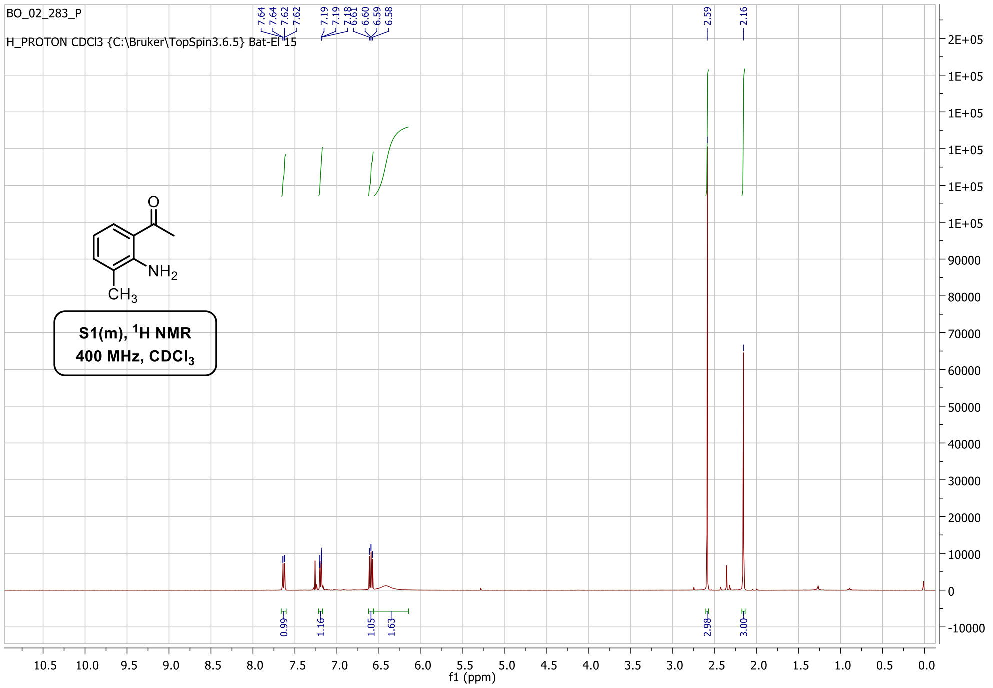

^{1}H NMR spectra of **S1(n)**

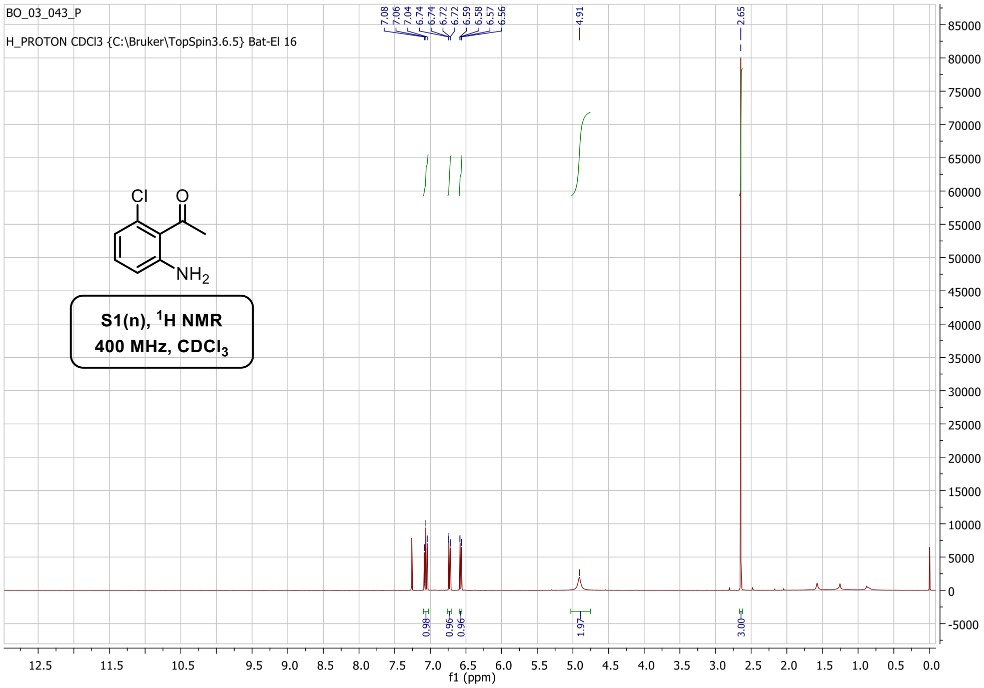

^{1}H and $^{13}C\{^{1}H\}$ NMR spectra of **S2(a)**

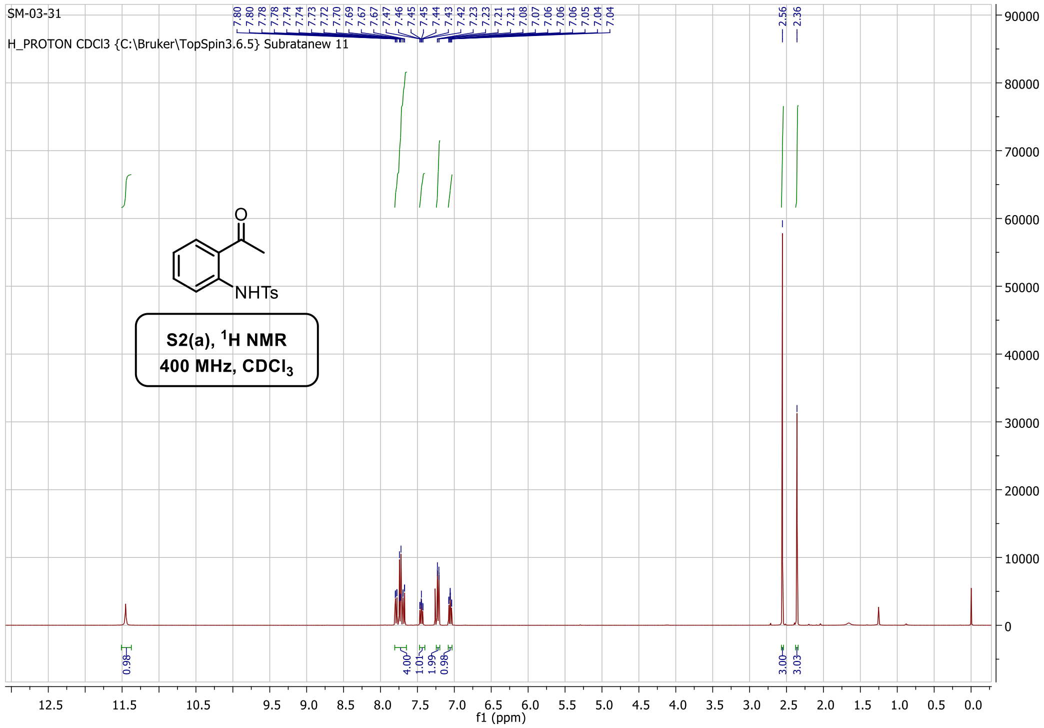

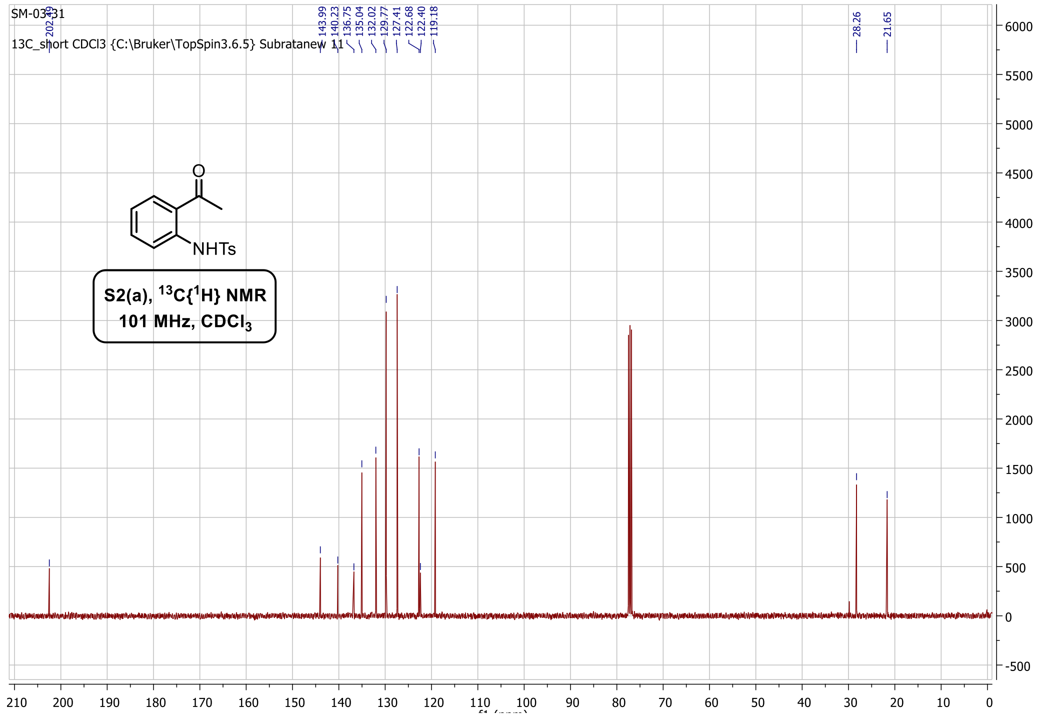

^{1}H and ^{13}C{^{1}H} NMR spectra of **S2(b)**

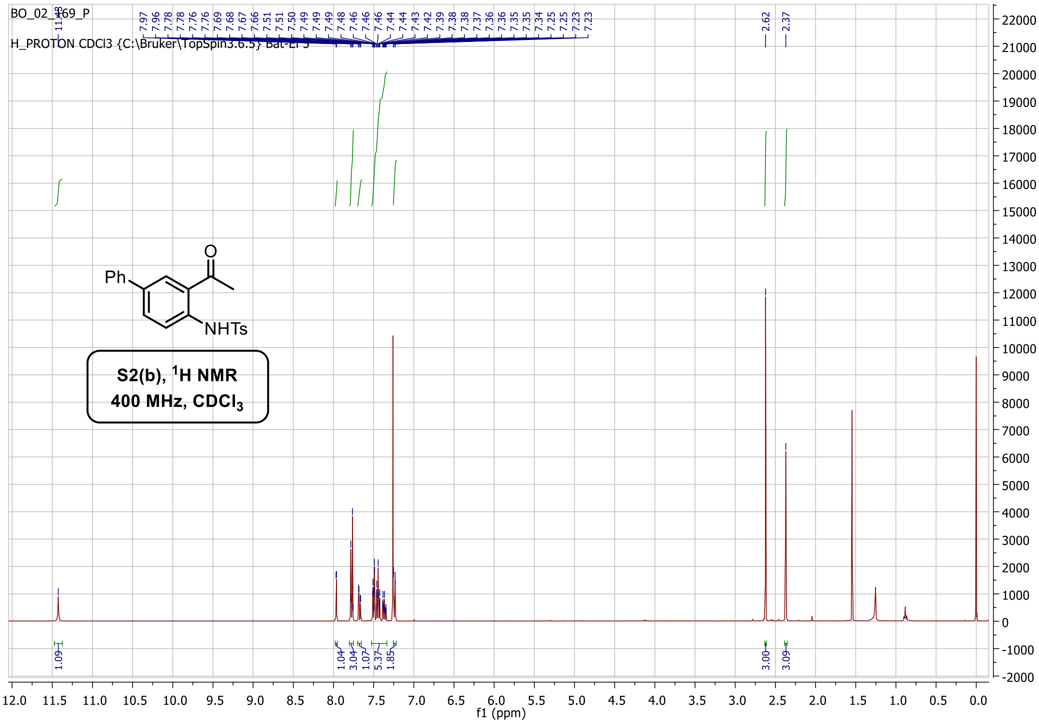

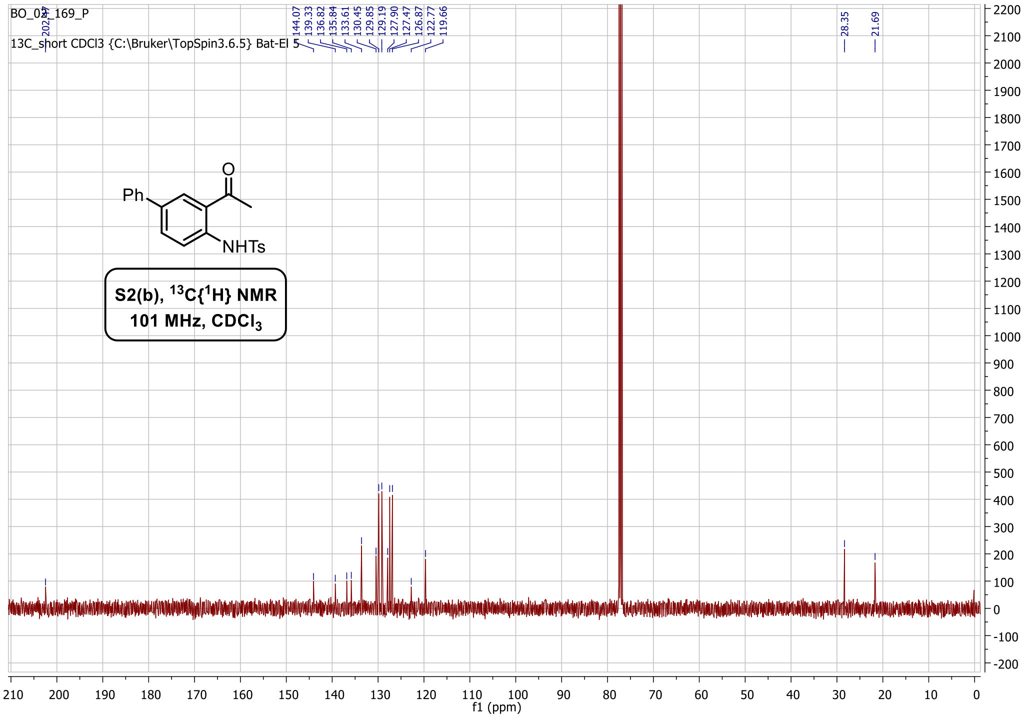

^{1}H and ^{13}C{^{1}H} NMR spectra of **S2(c)**

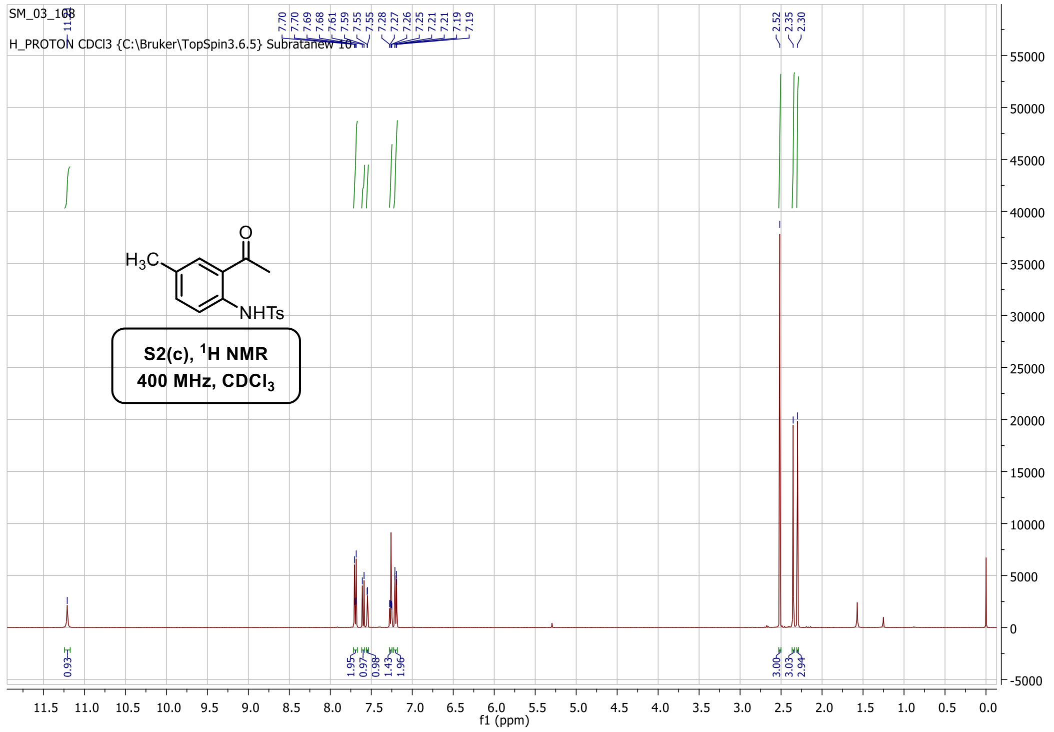

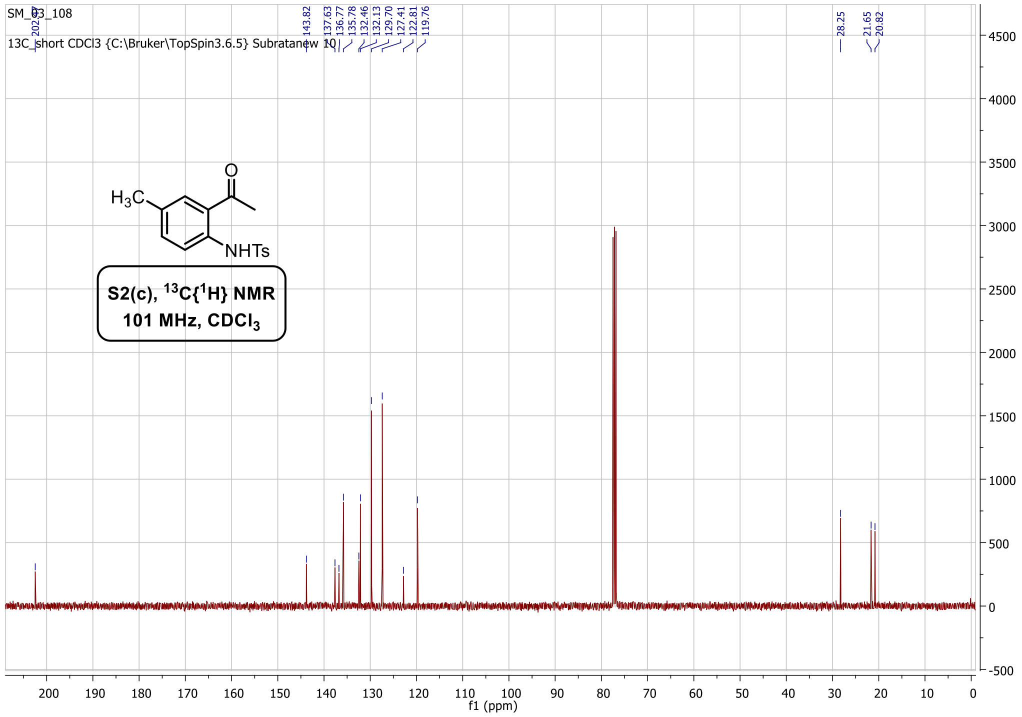

^{1}H and ^{13}C{^{1}H} NMR spectra of **S2(d)**

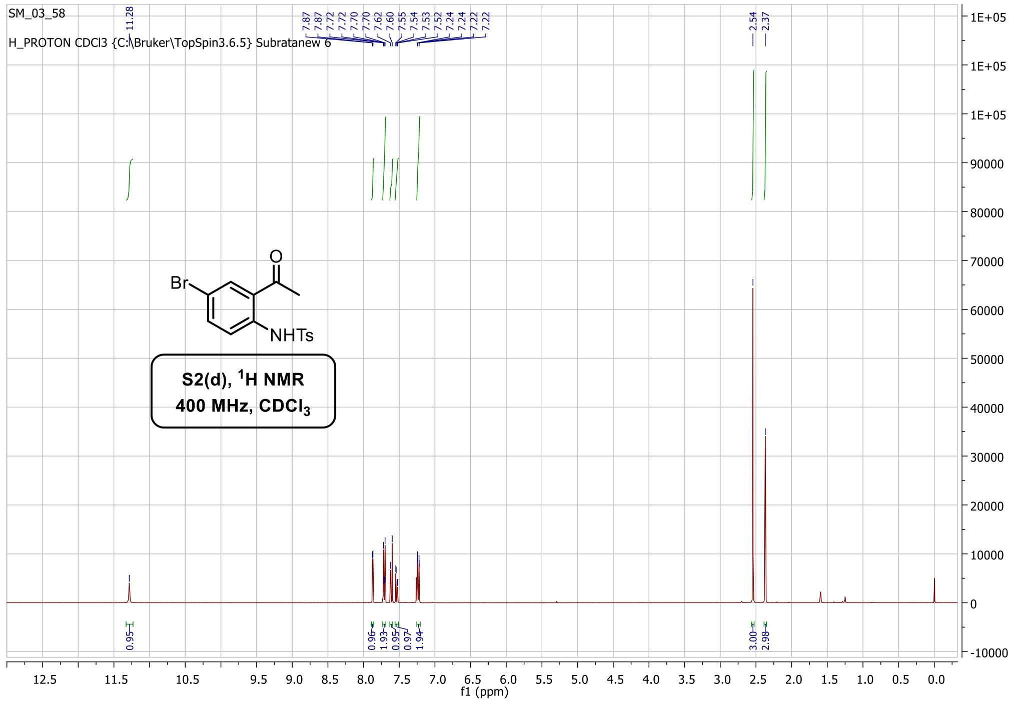

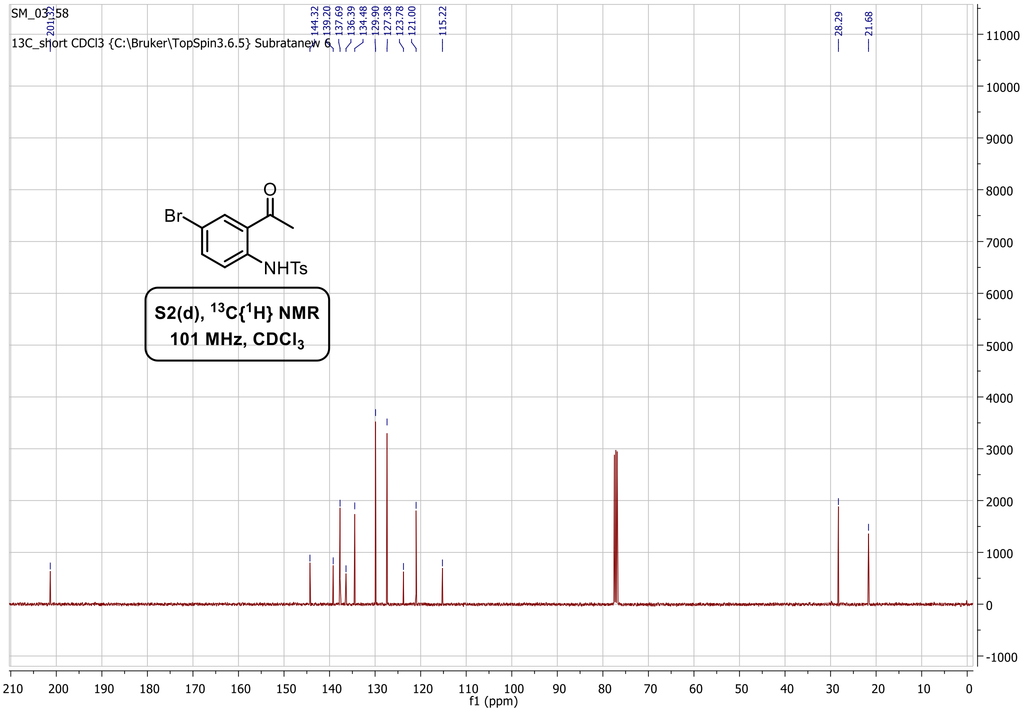

^{1}H and ^{13}C{^{1}H} NMR spectra of **S2(e)**

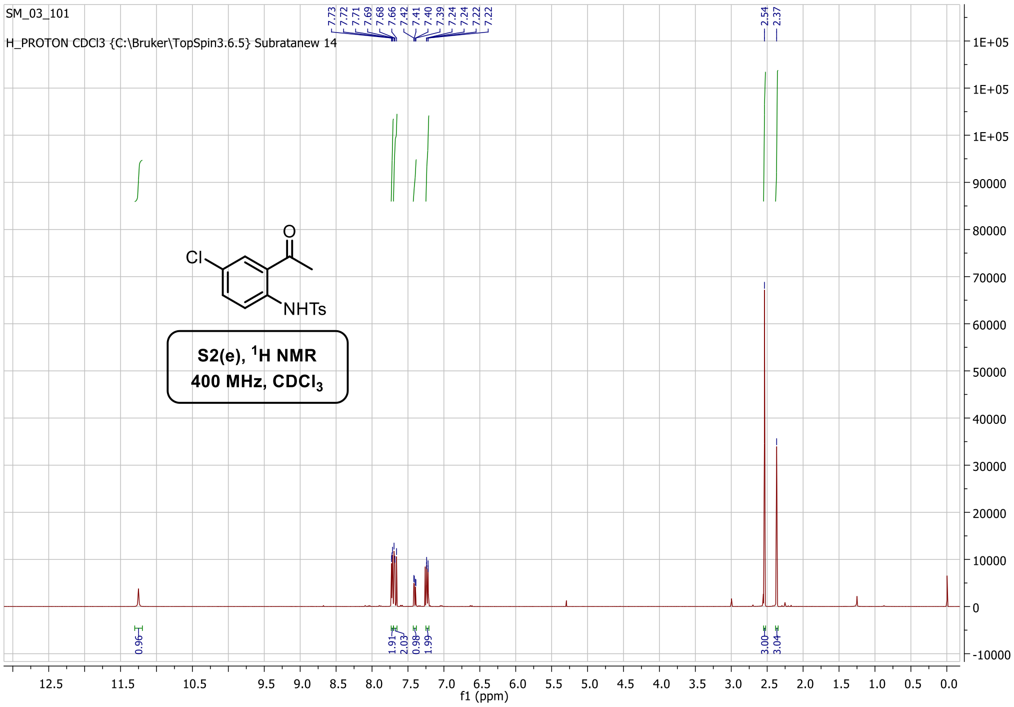

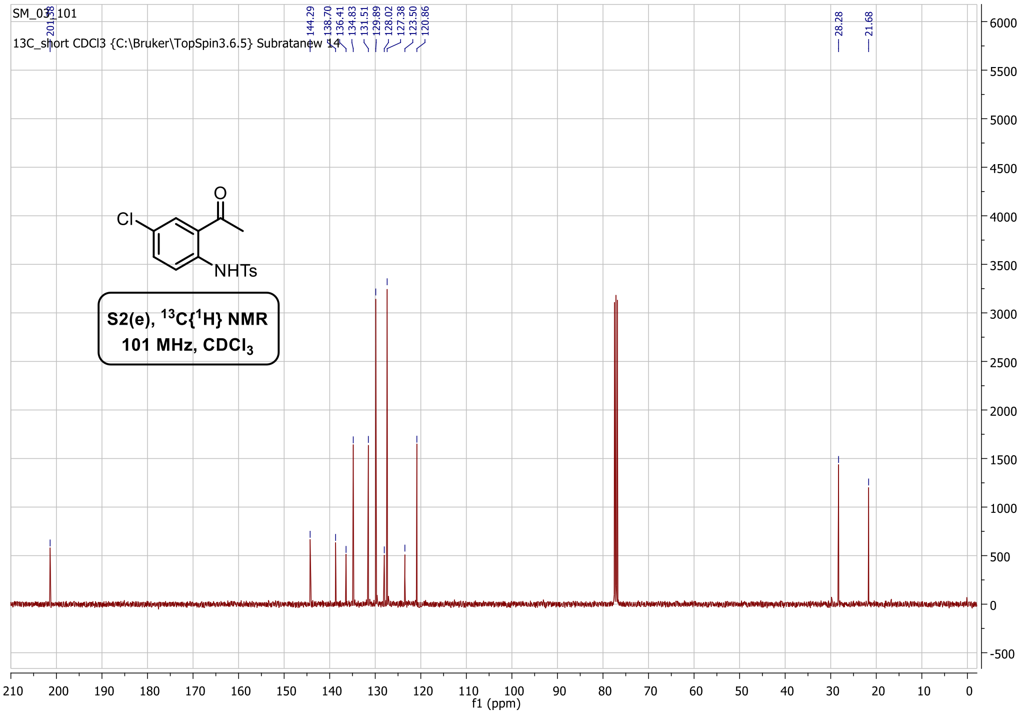

^1H and $^{13}C\{^1H\}$ NMR spectra of **S2(f)**

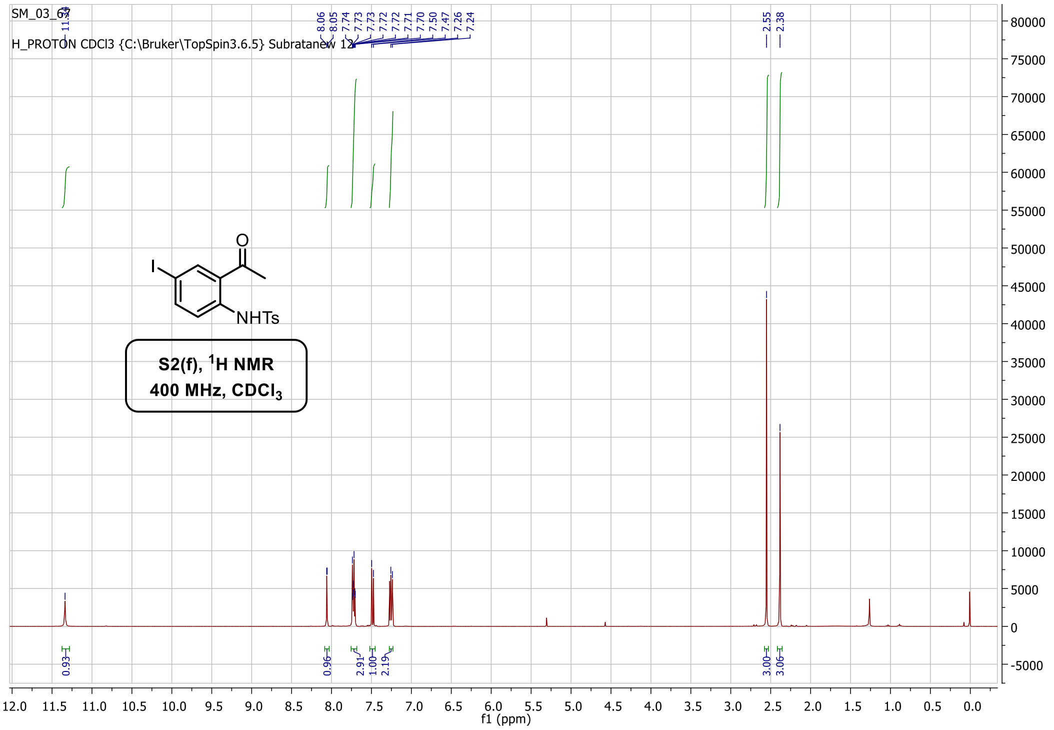

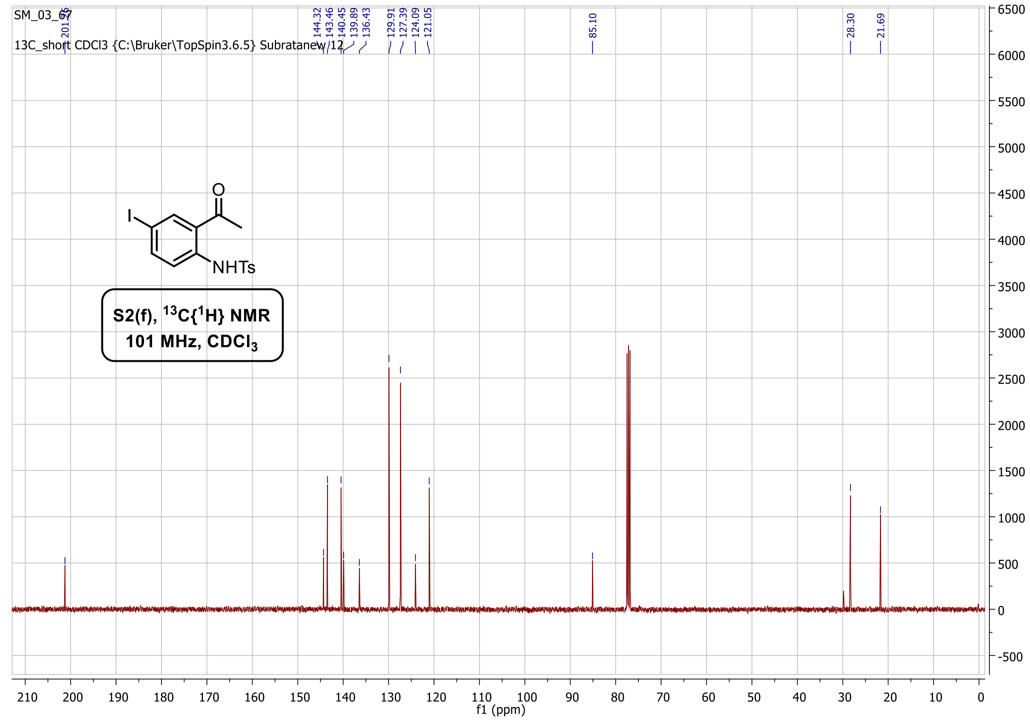

^{1}H, $^{13}C\{^{1}H\}$ and ^{19}F NMR spectra of **S2(g)**

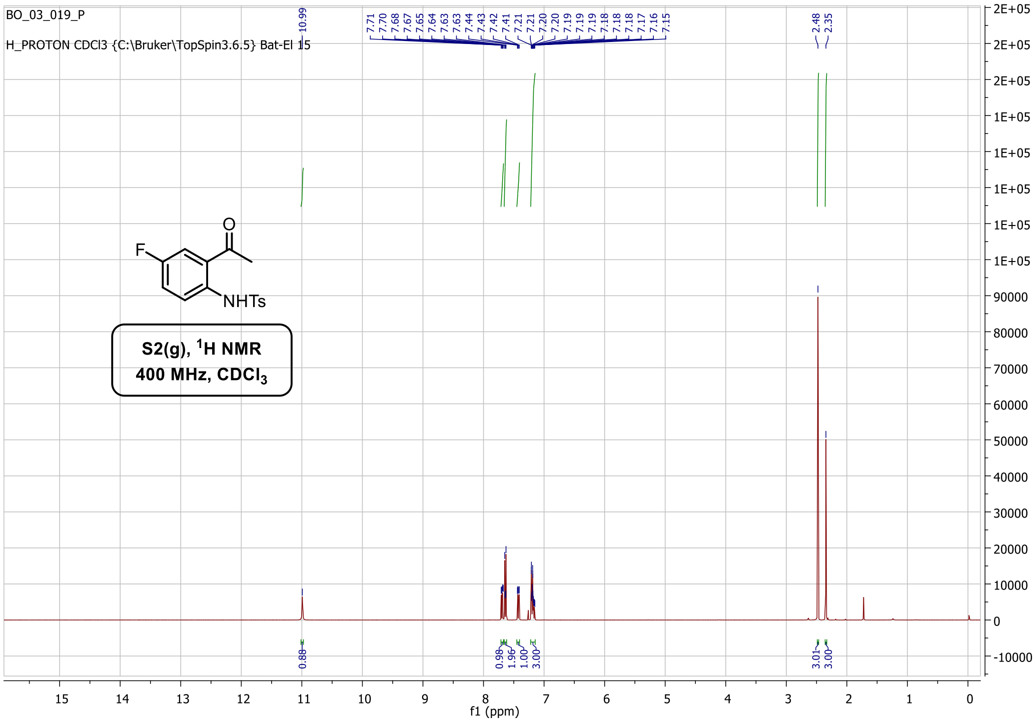

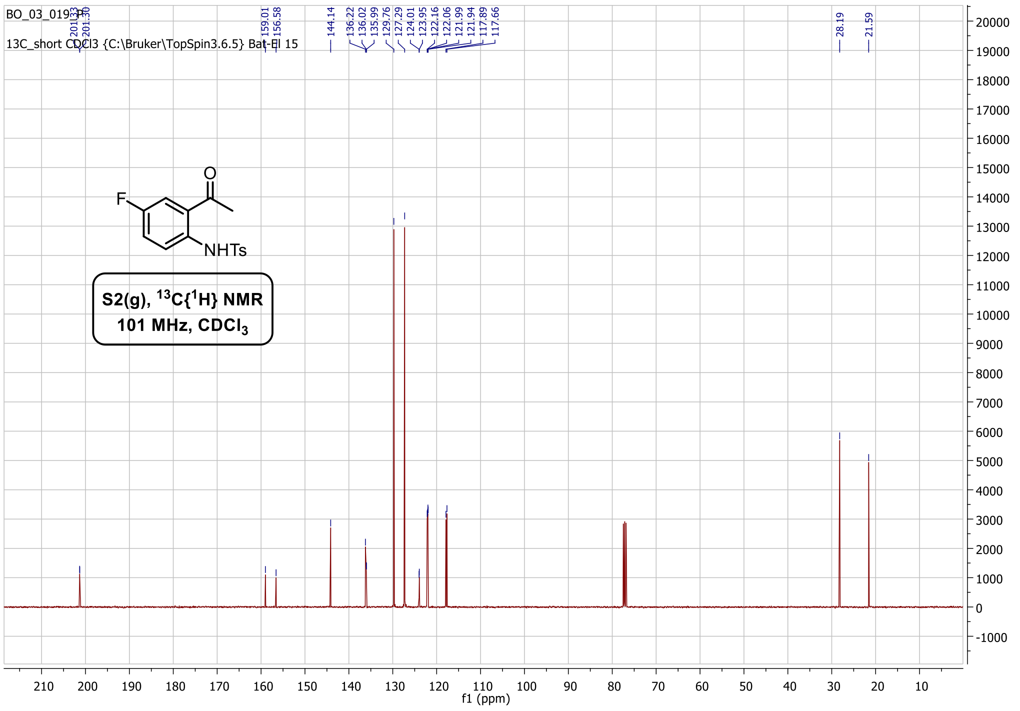

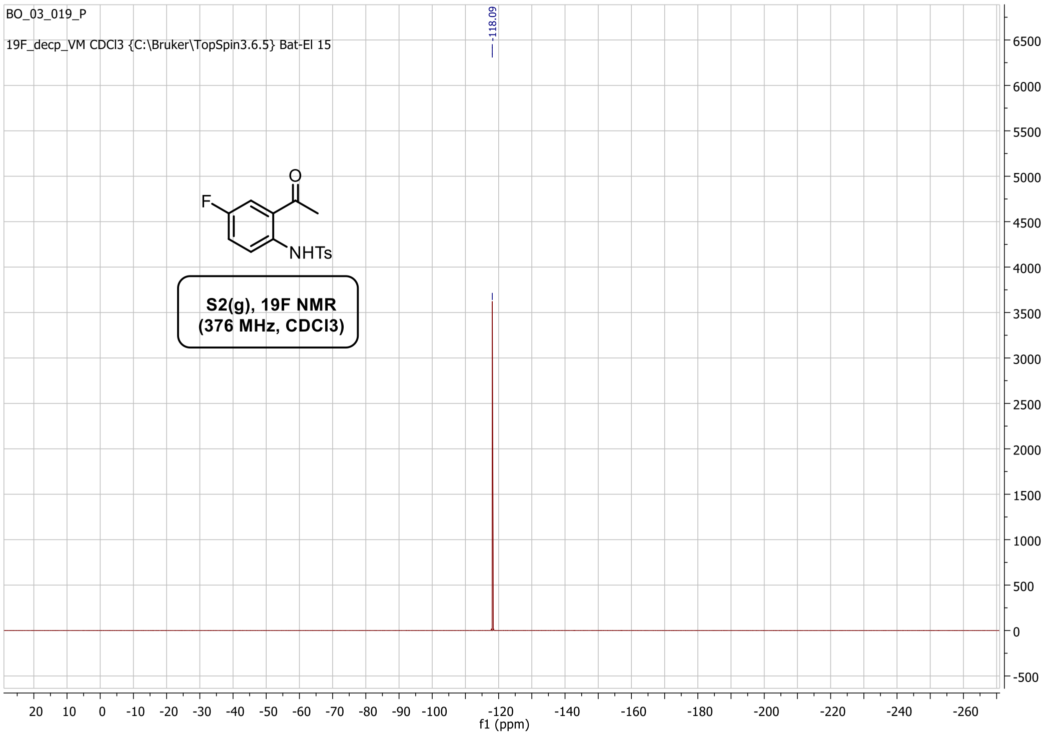
BO_03_019_P
19F_decp_VM CDCl3 {C:\Bruker\TopSpin3.6.5} Bat-El 15
-118.09
F
O
NHTs
S2(g), 19F NMR
(376 MHz, CDCl3)
f1 (ppm)

^{1}H and $^{13}C\{^{1}H\}$ NMR spectra of **S2(h)**

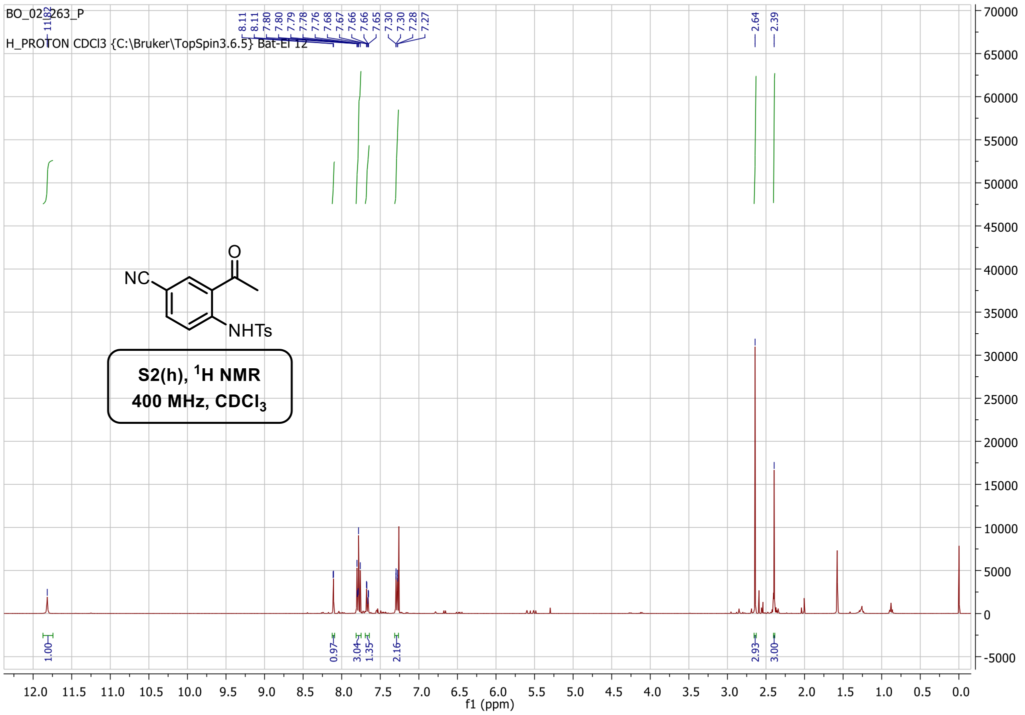

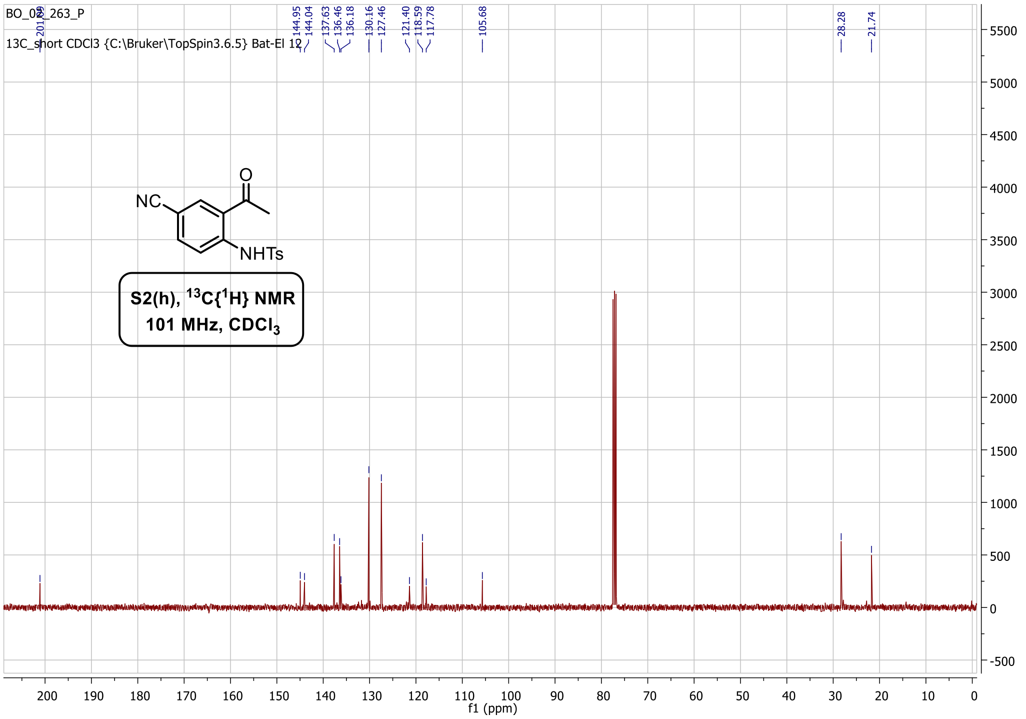

^{1}H and $^{13}C\{^{1}H\}$ NMR spectra of **S2(i)**

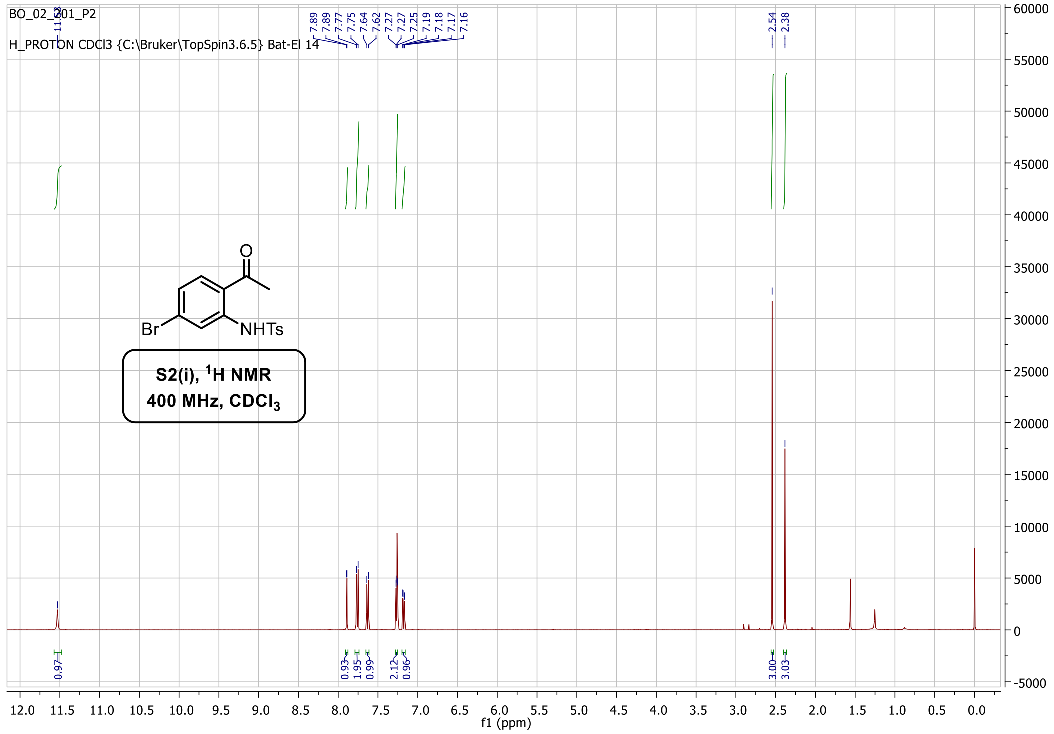

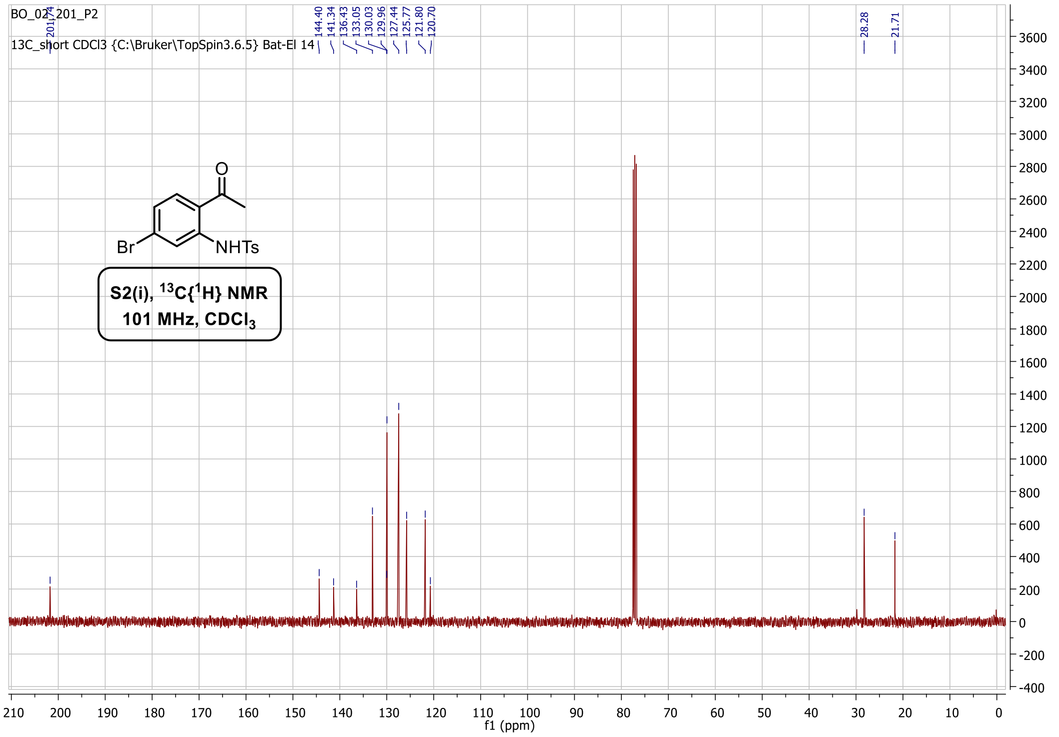

^{1}H and $^{13}C\{^{1}H\}$ NMR spectra of **S2(j)**

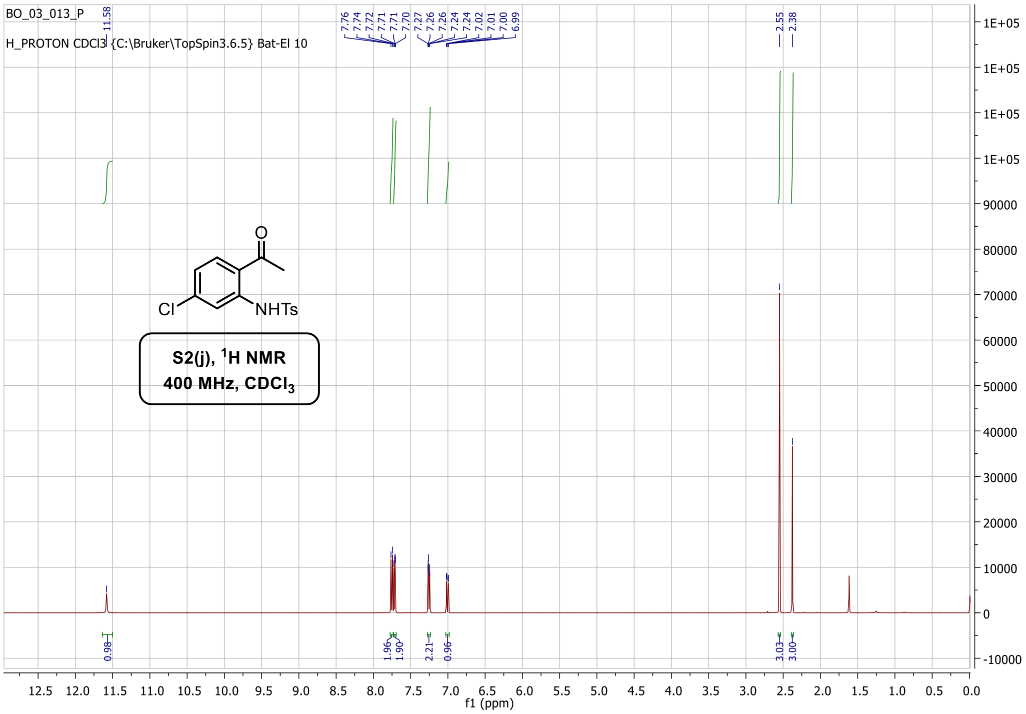

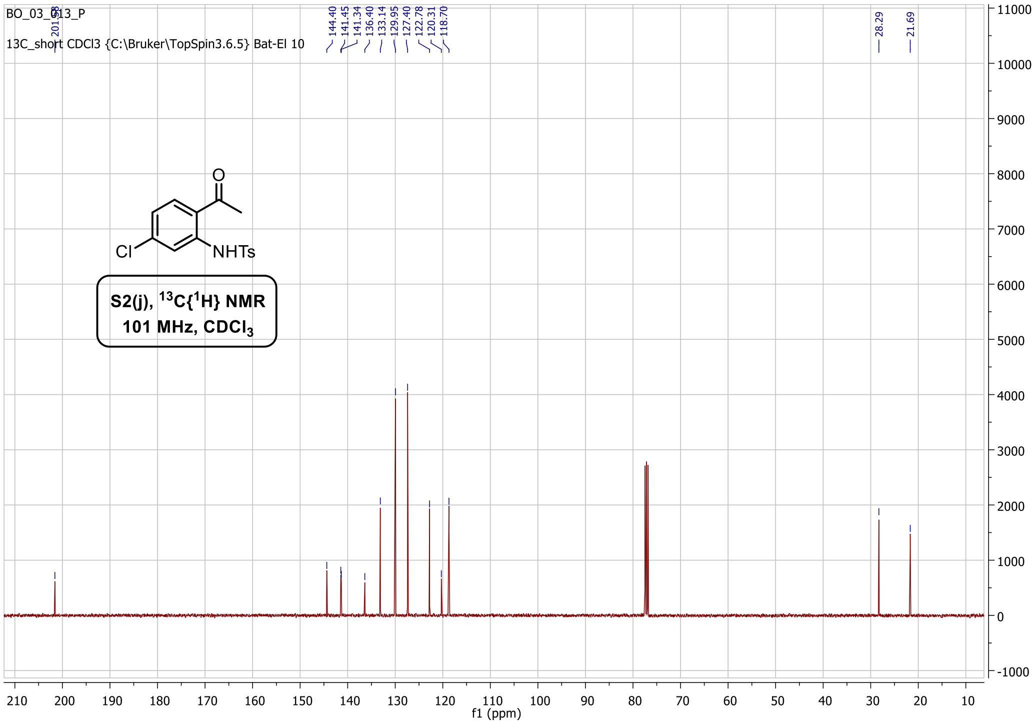

^{1}H, ^{13}C{^{1}H} and ^{19}F NMR spectra of **S2(k)**

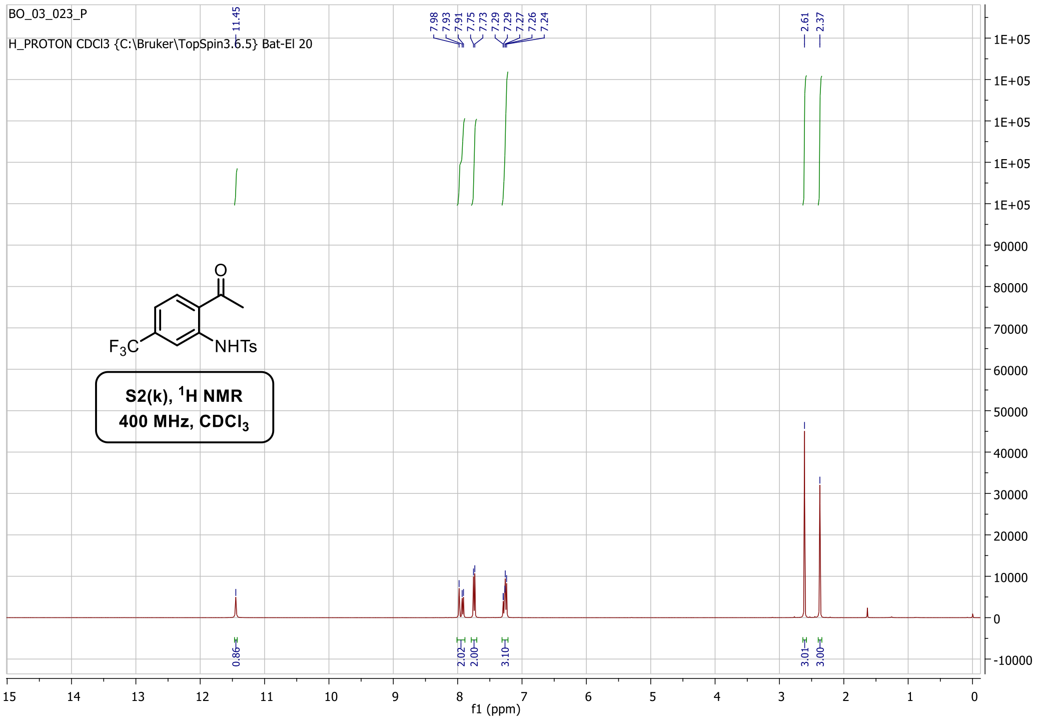

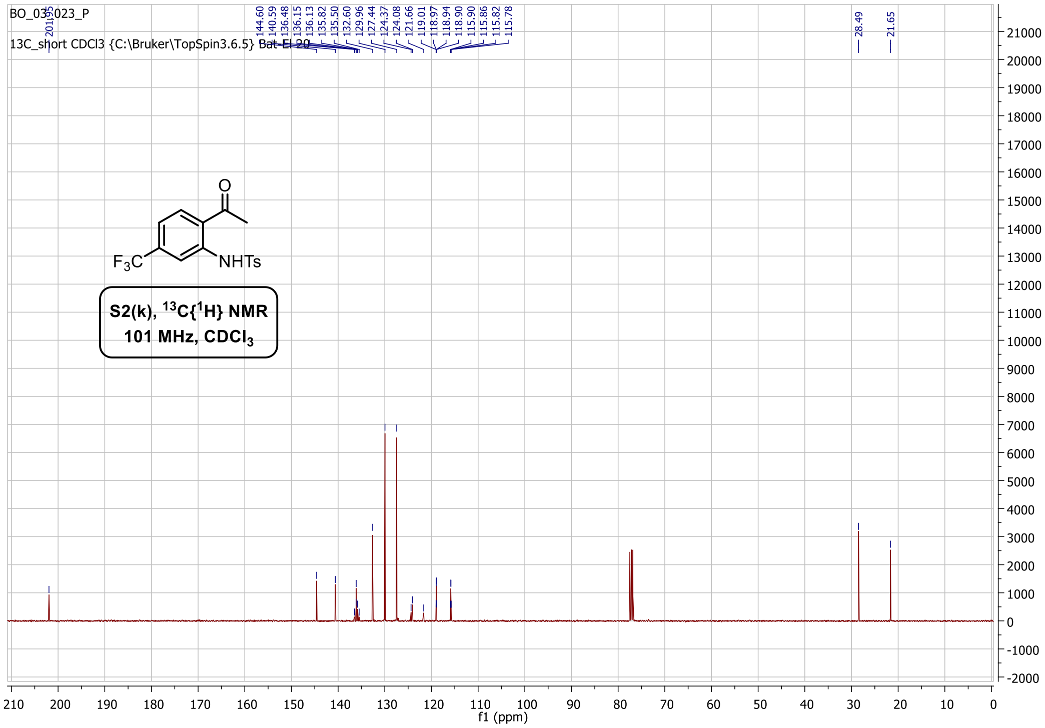

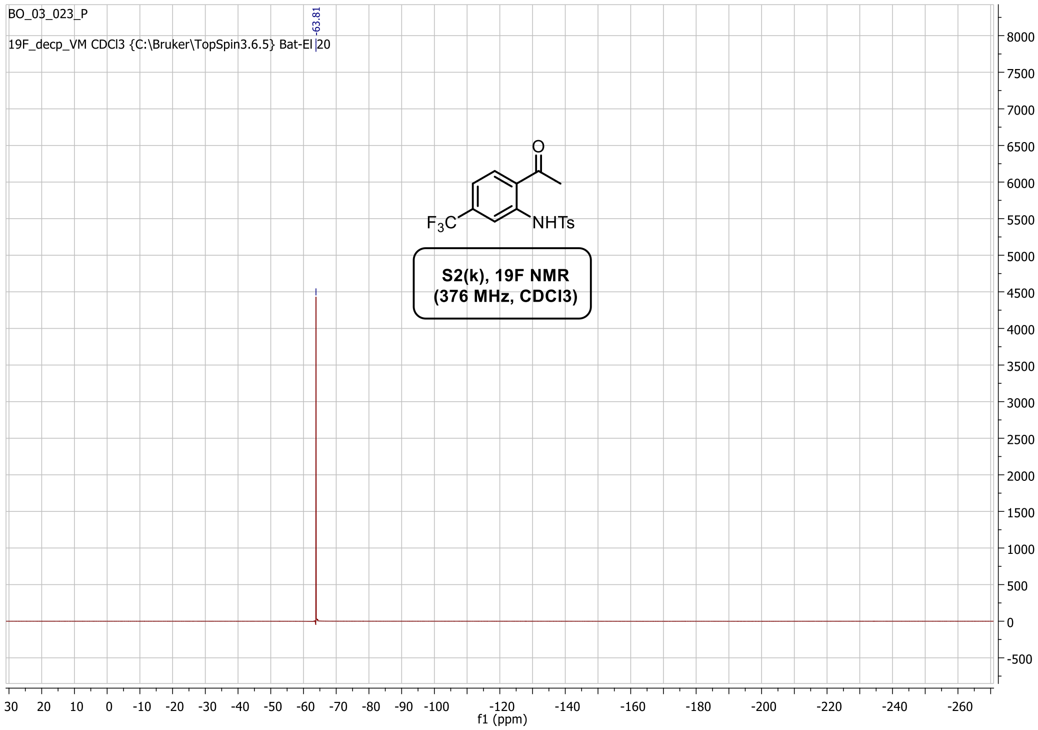

BO_03_023_P
19F_decp_VM CDCl3 {C:\Bruker\TopSpin3.6.5} Bat-El 20
-63.81
F3C
NHTs
O
S2(k), 19F NMR
(376 MHz, CDCl3)
f1 (ppm)

^1H and $^{13}C\{^1H\}$ NMR spectra of **S2(l)**

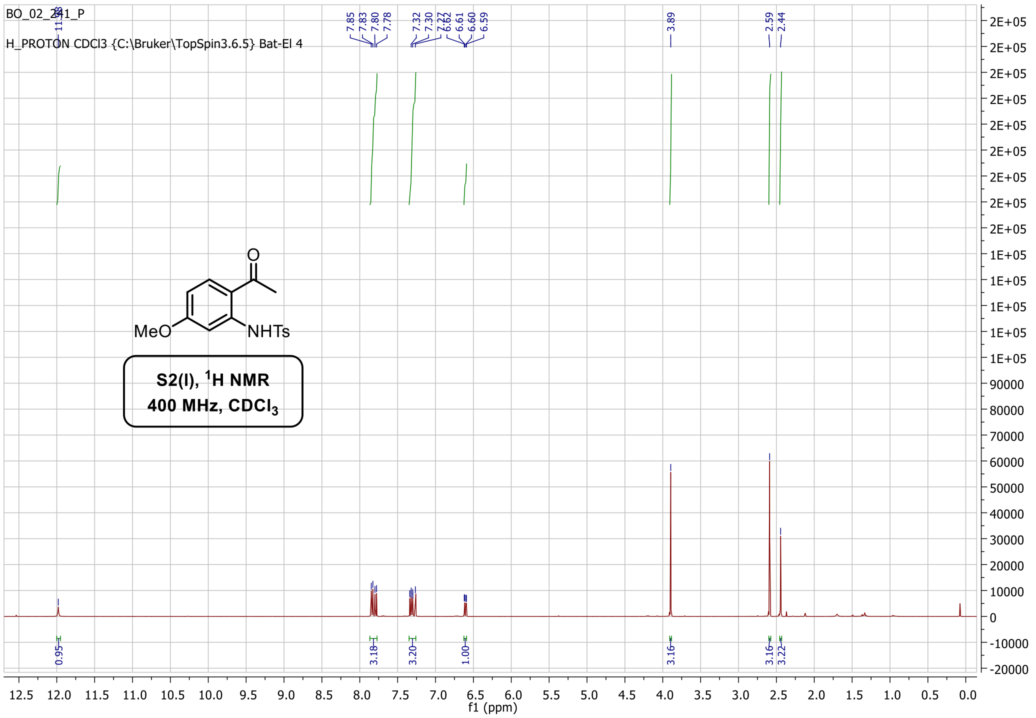

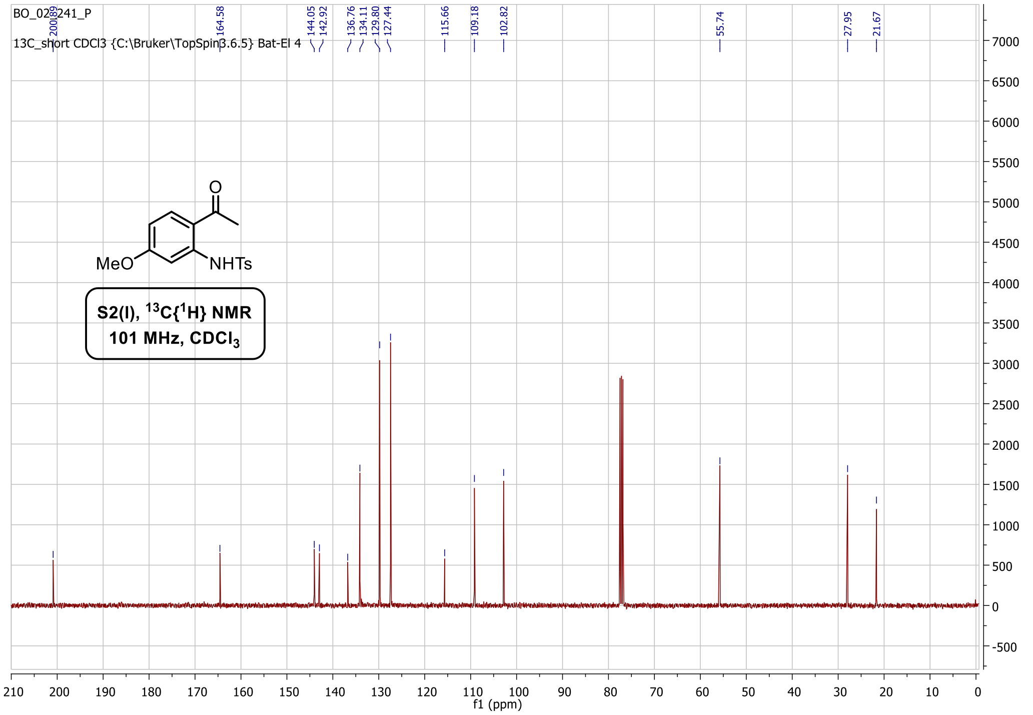

^{1}H and $^{13}C\{^{1}H\}$ NMR spectra of **S2(m)**

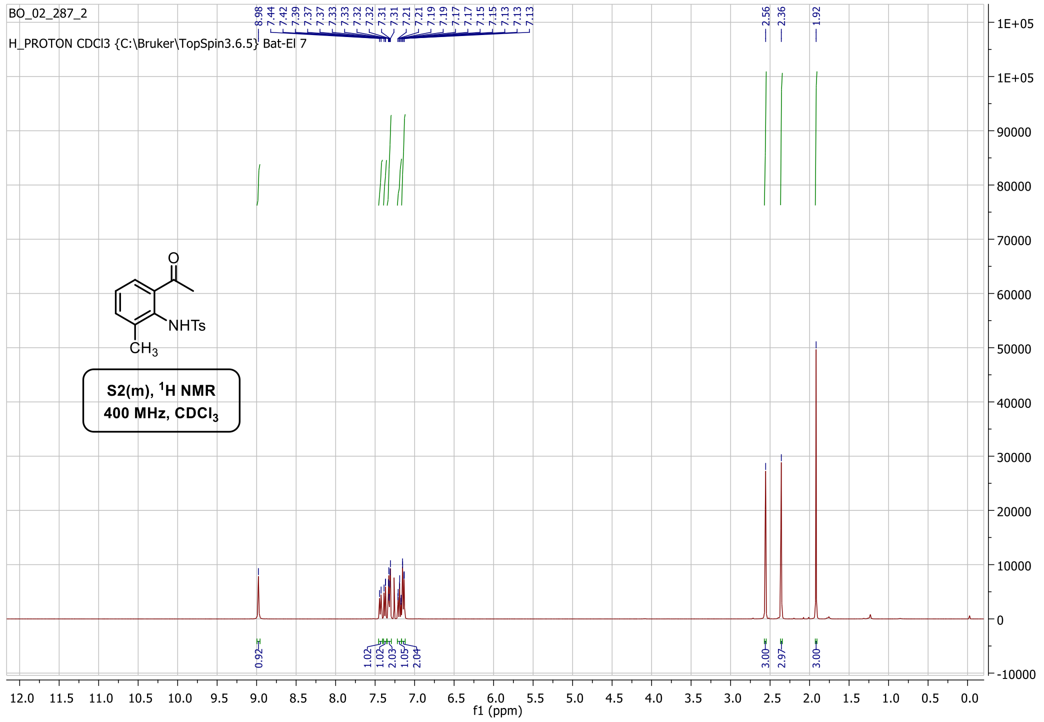

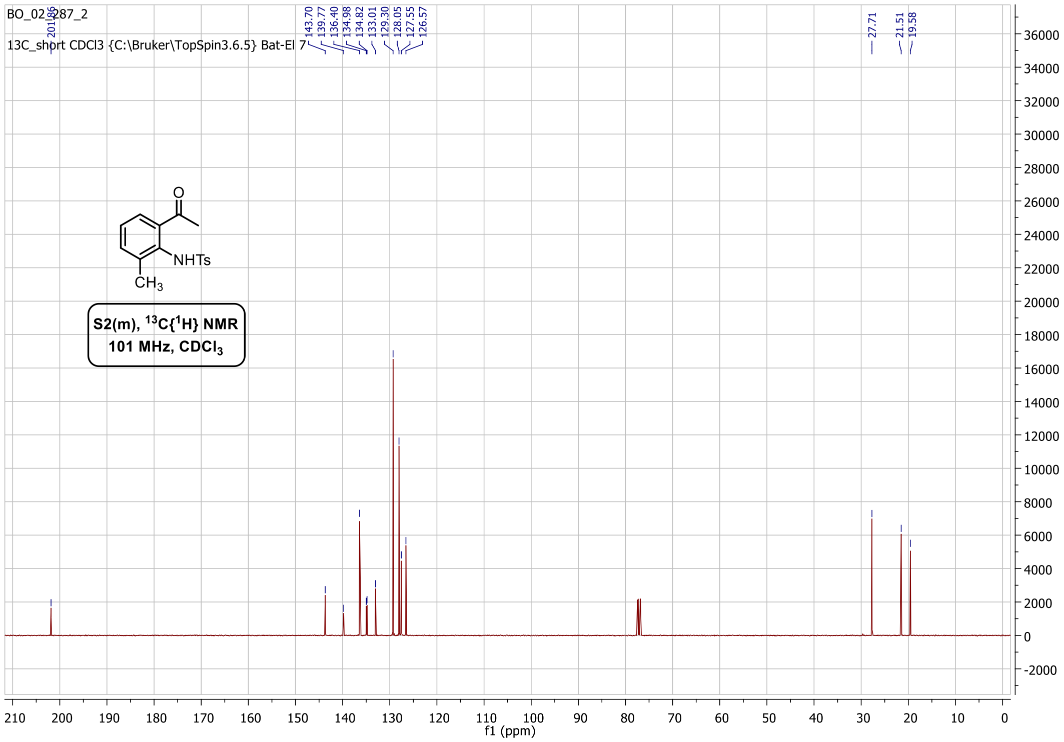

^{1}H and $^{13}C\{^{1}H\}$ NMR spectra of **S2(n)**

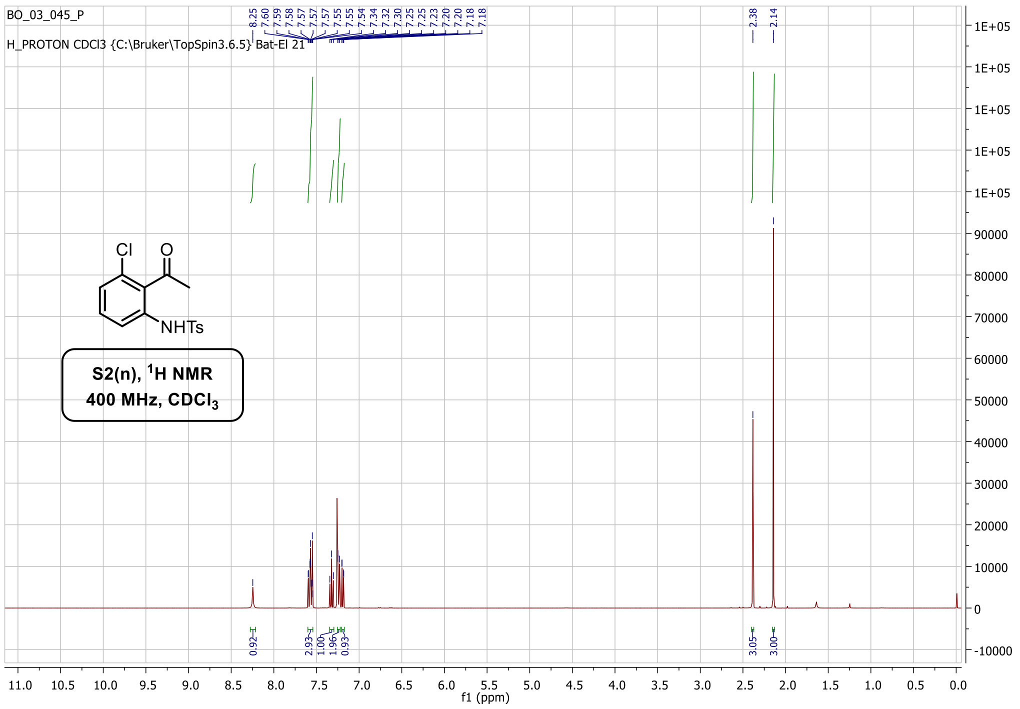

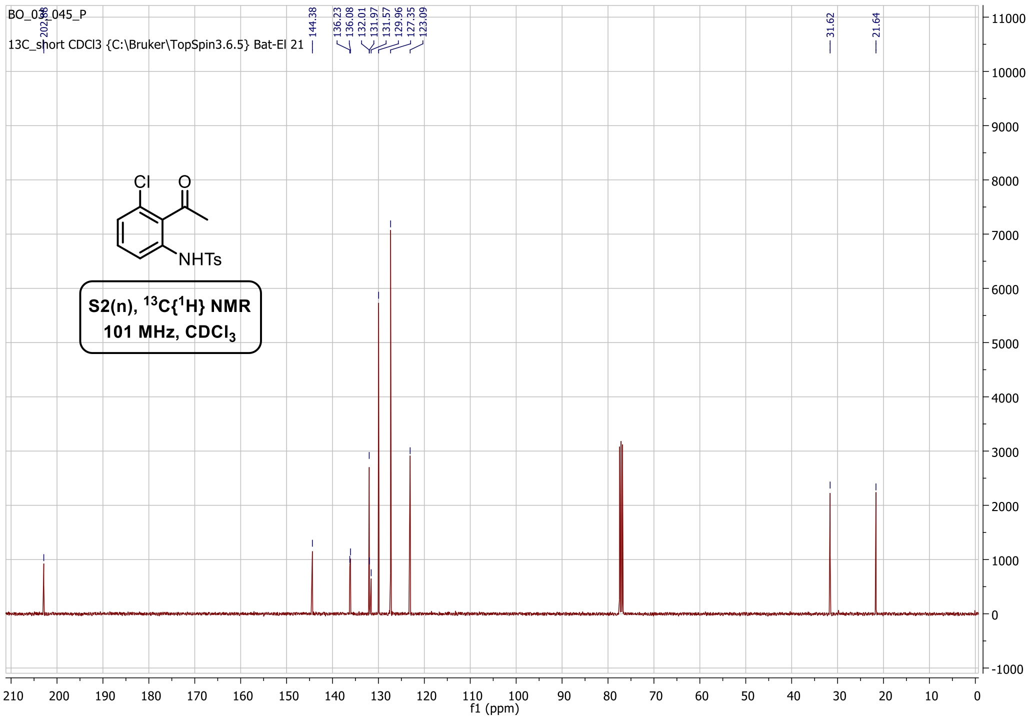

^{1}H and $^{13}C\{^{1}H\}$ NMR spectra of **1(a)**

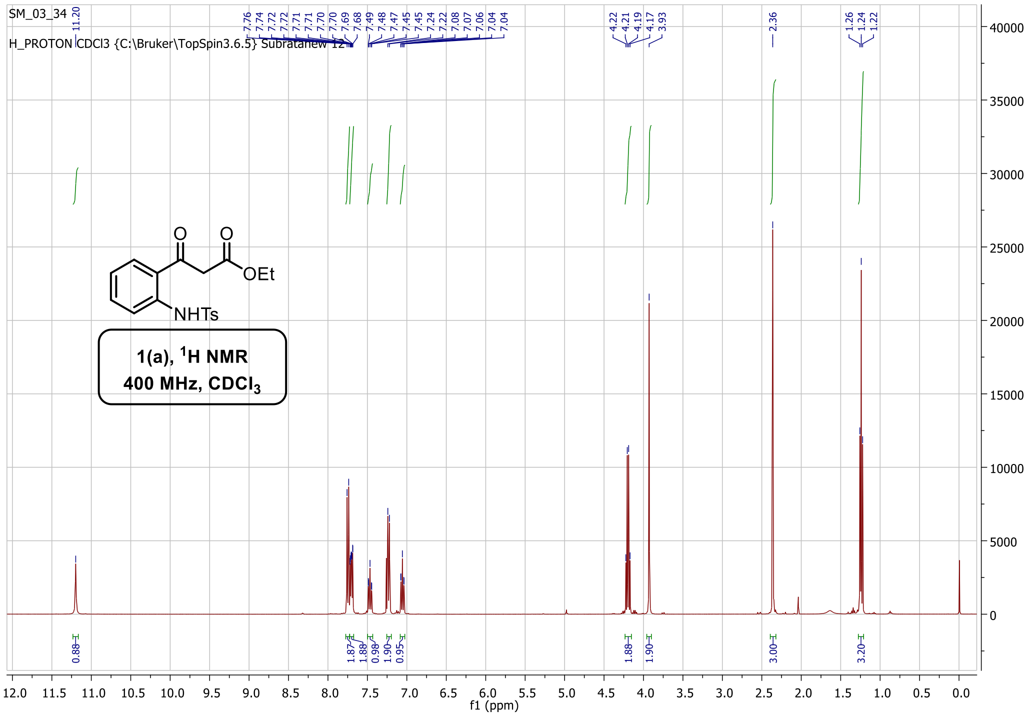

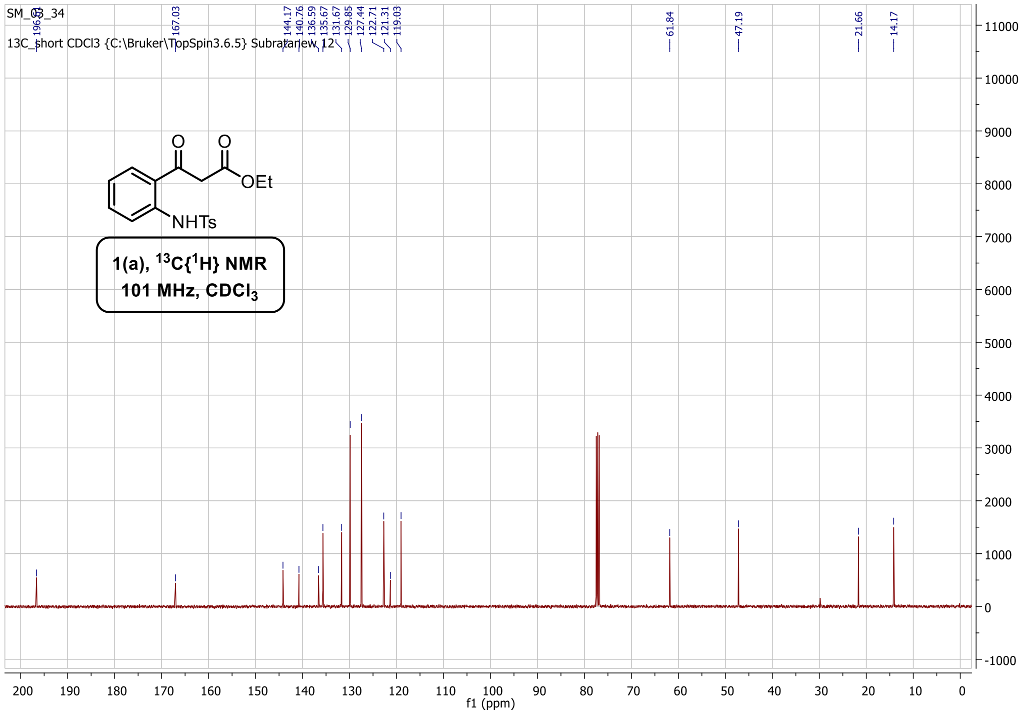

^1H and $^{13}C\{^1H\}$ NMR spectra of **1(b)**

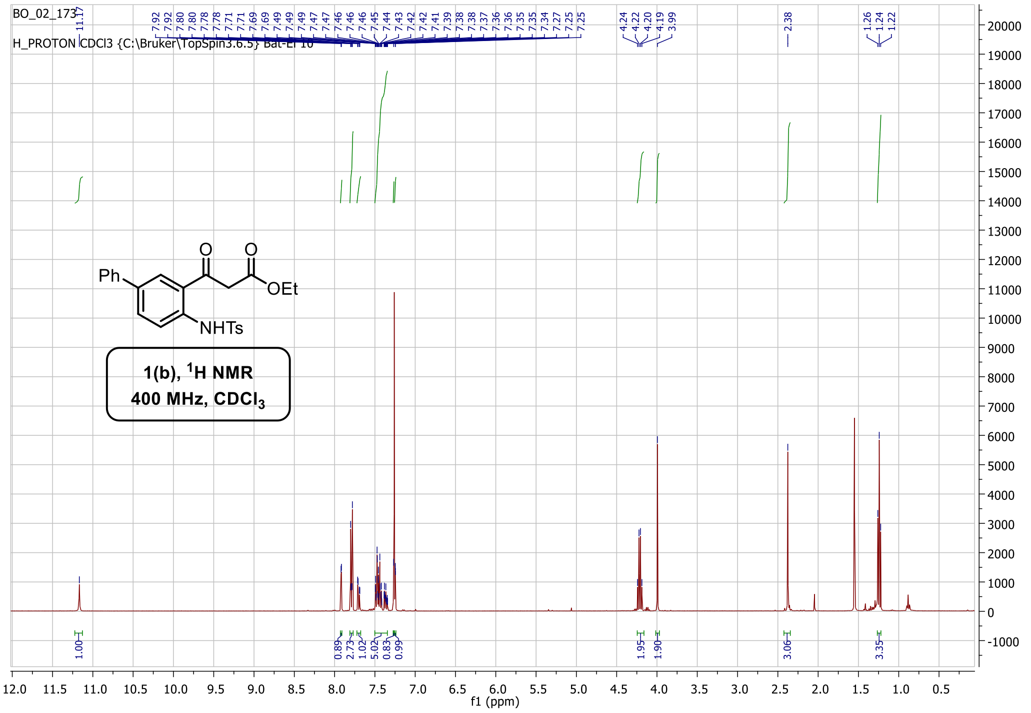

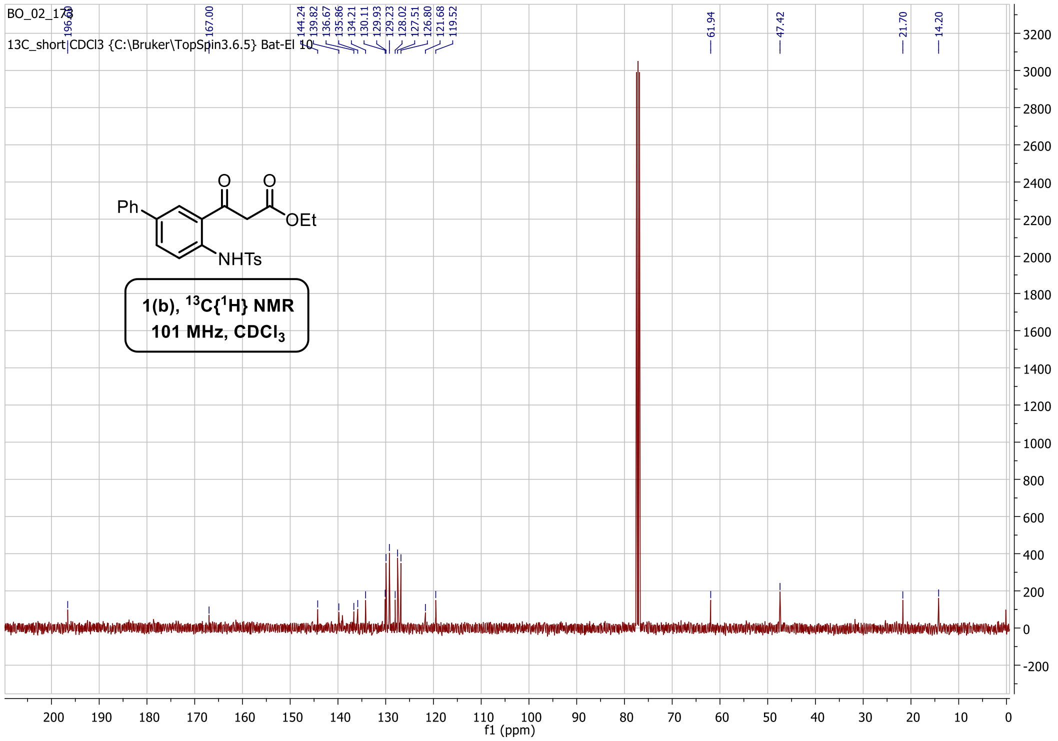

^{1}H and $^{13}C\{^{1}H\}$ NMR spectra of **1(c)**

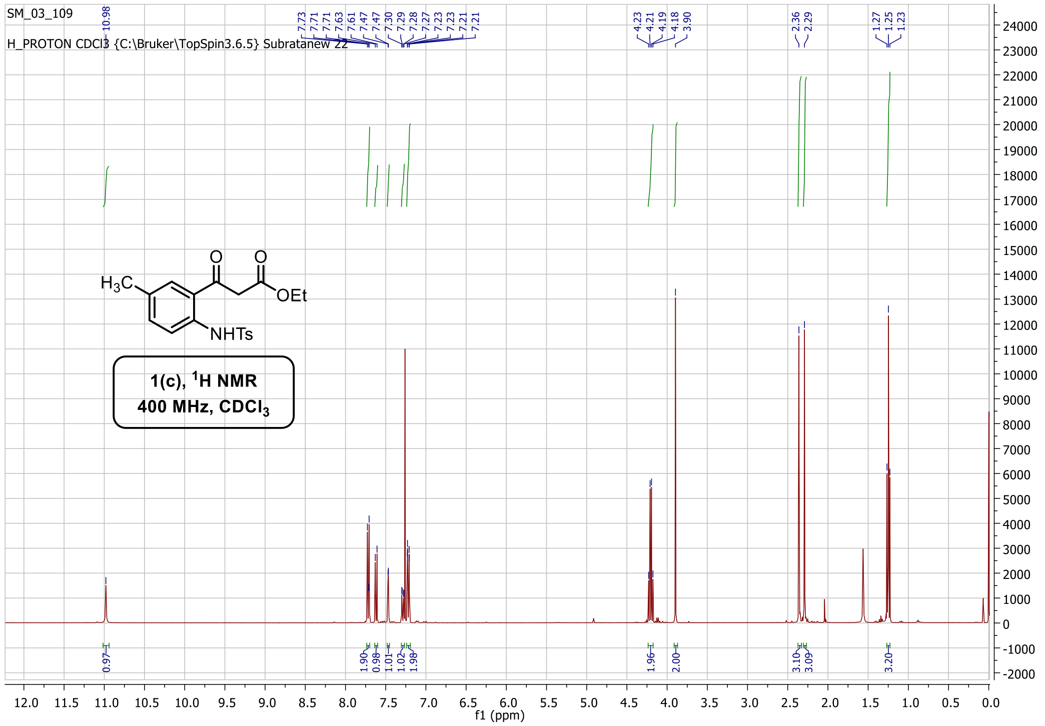

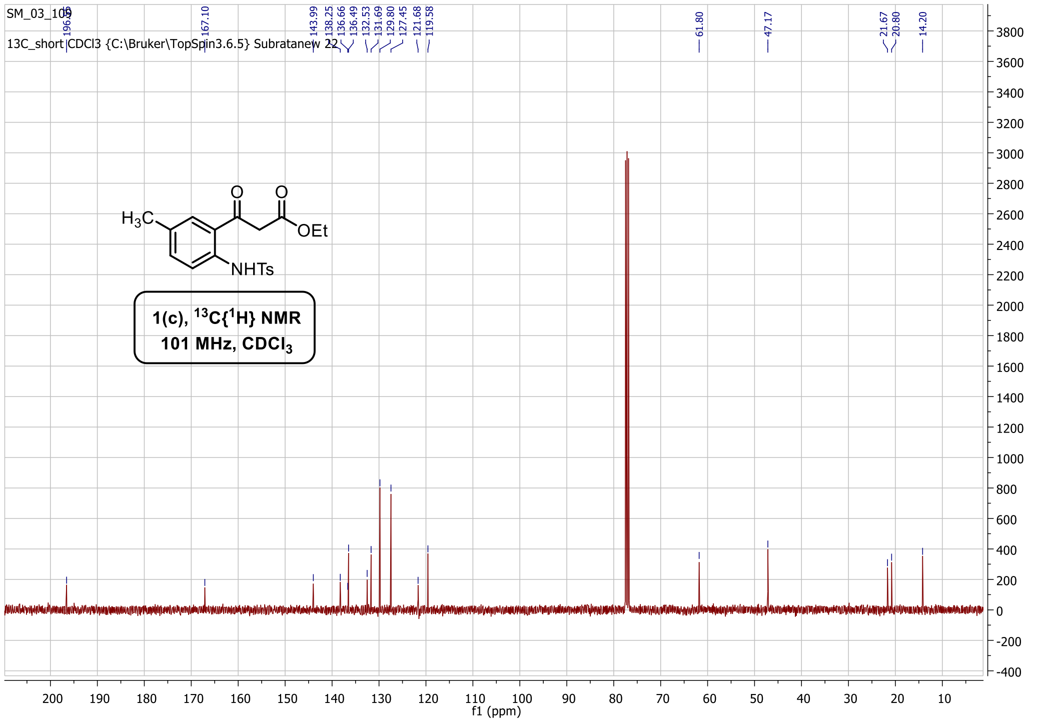

^{1}H and ^{13}C{^{1}H} NMR spectra of **1(d)**

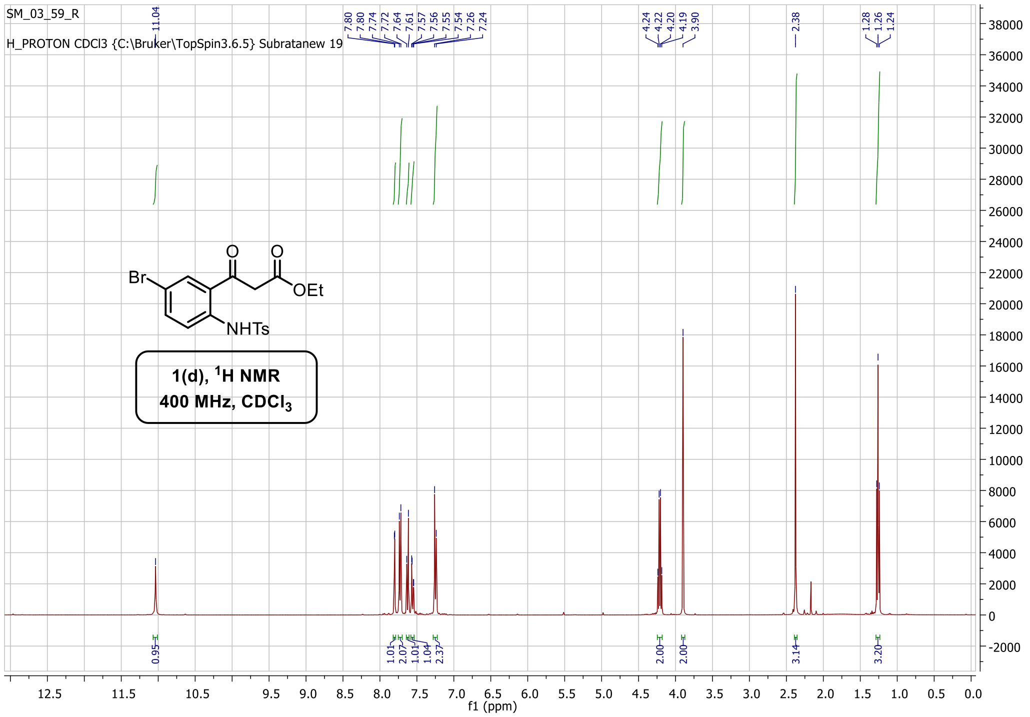

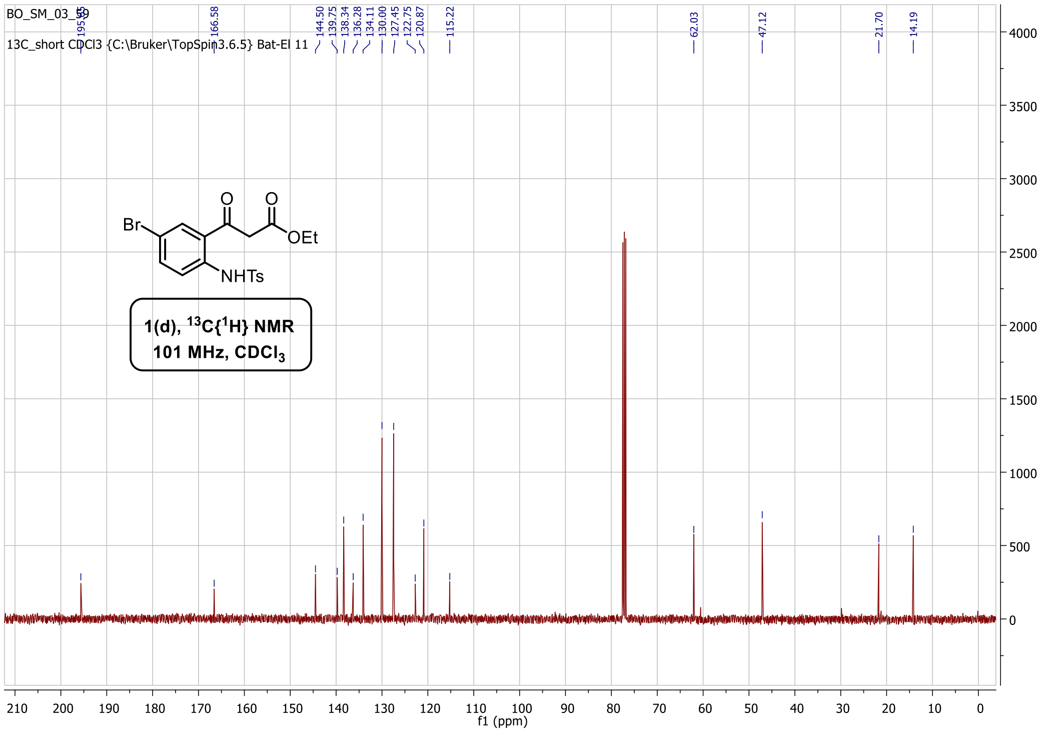

^{1}H and $^{13}C\{^{1}H\}$ NMR spectra of **1(e)**

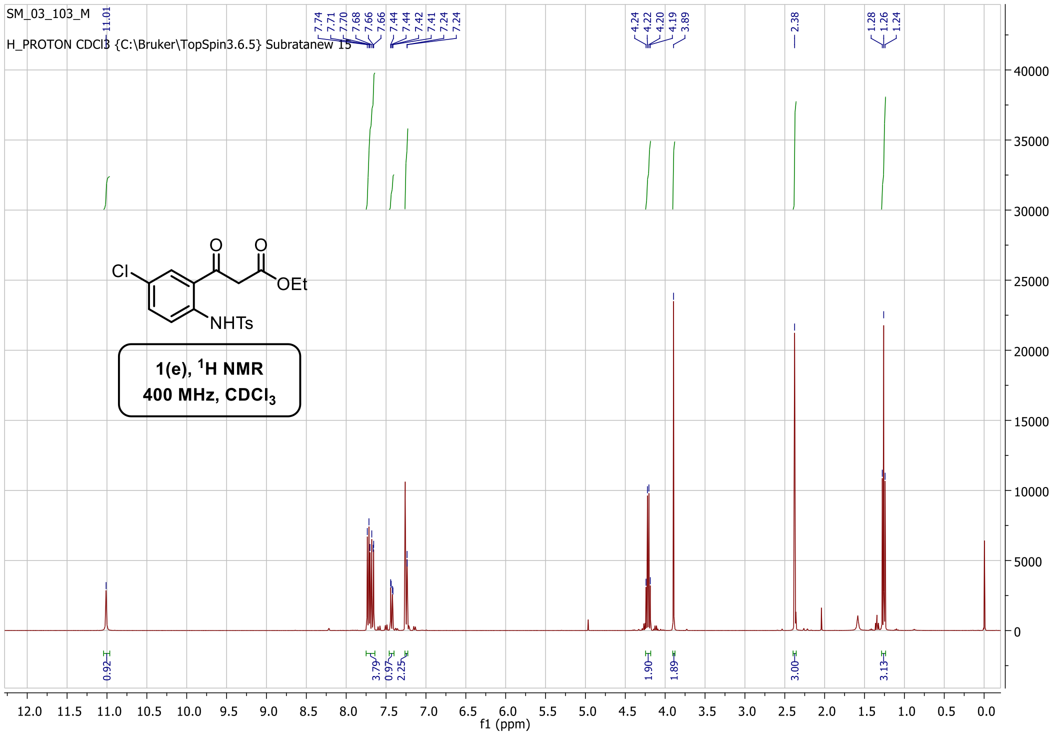

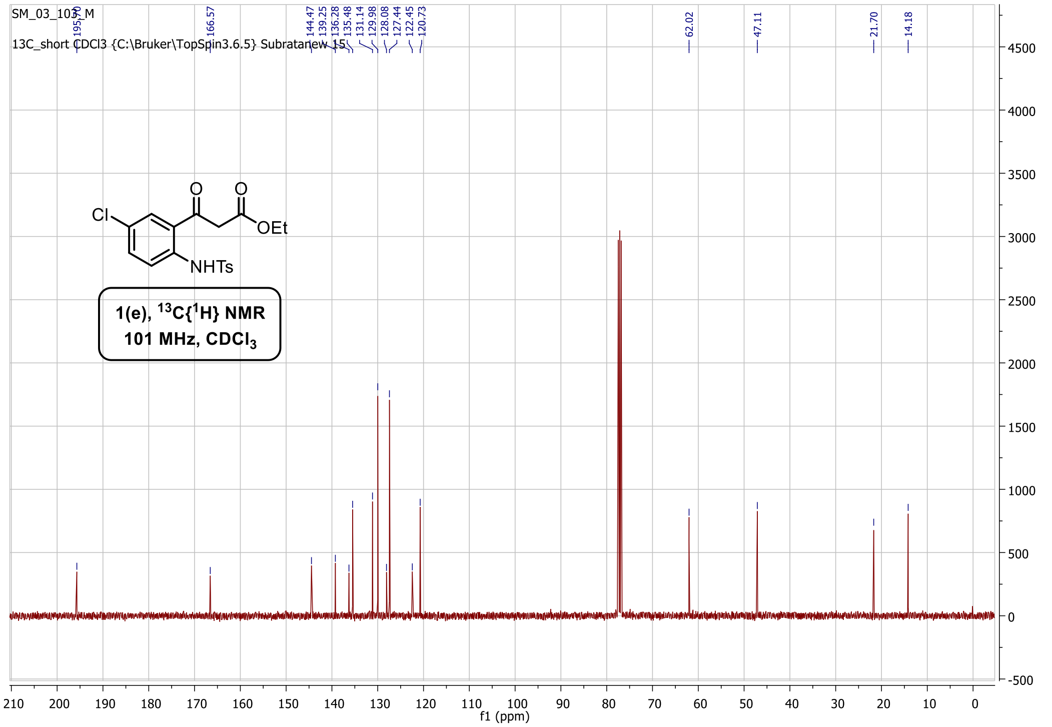

^{1}H and ^{13}C{^{1}H} NMR spectra of **1(f)**

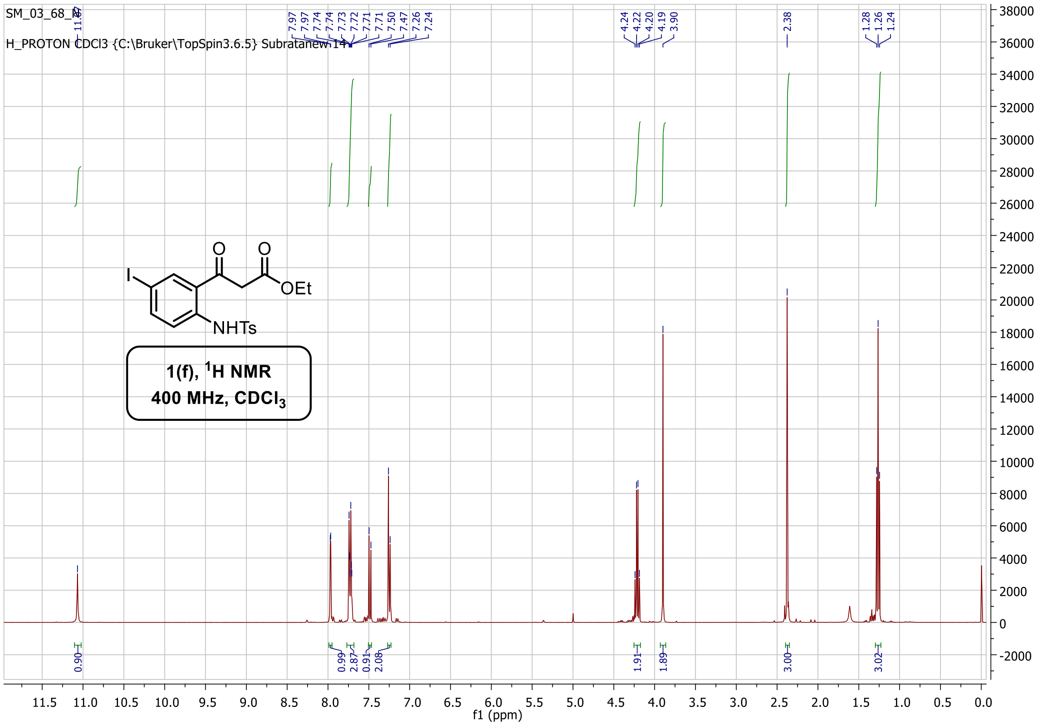

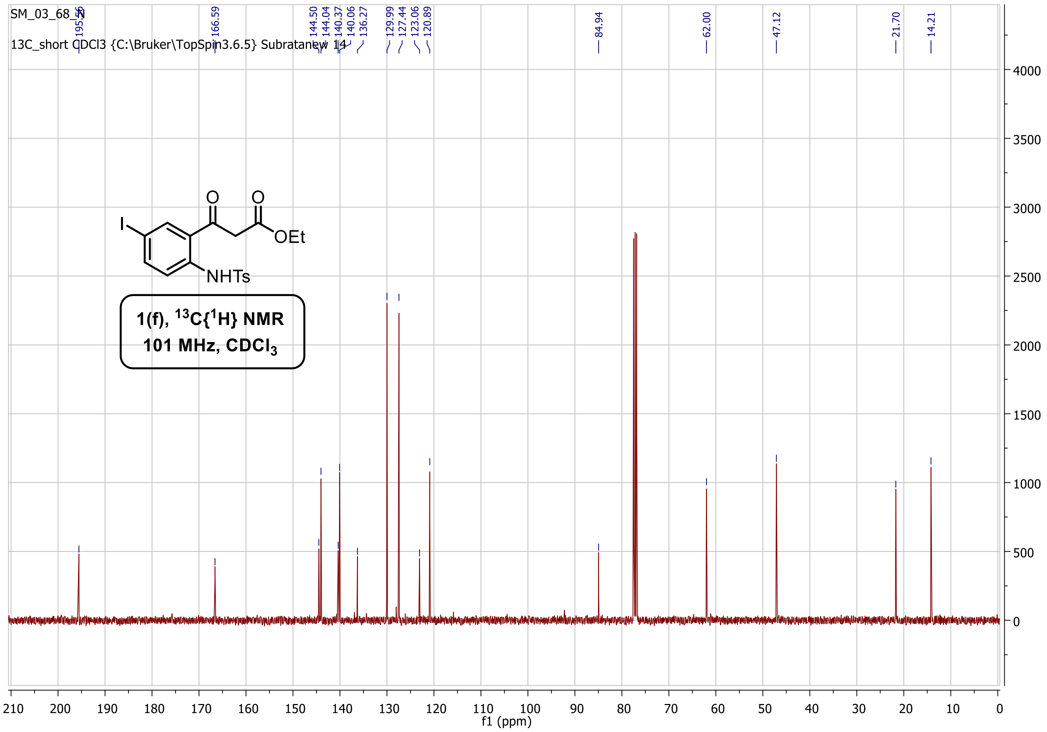

^{1}H, $^{13}C\{^{1}H\}$ and ^{19}F NMR spectra of **1(g)**

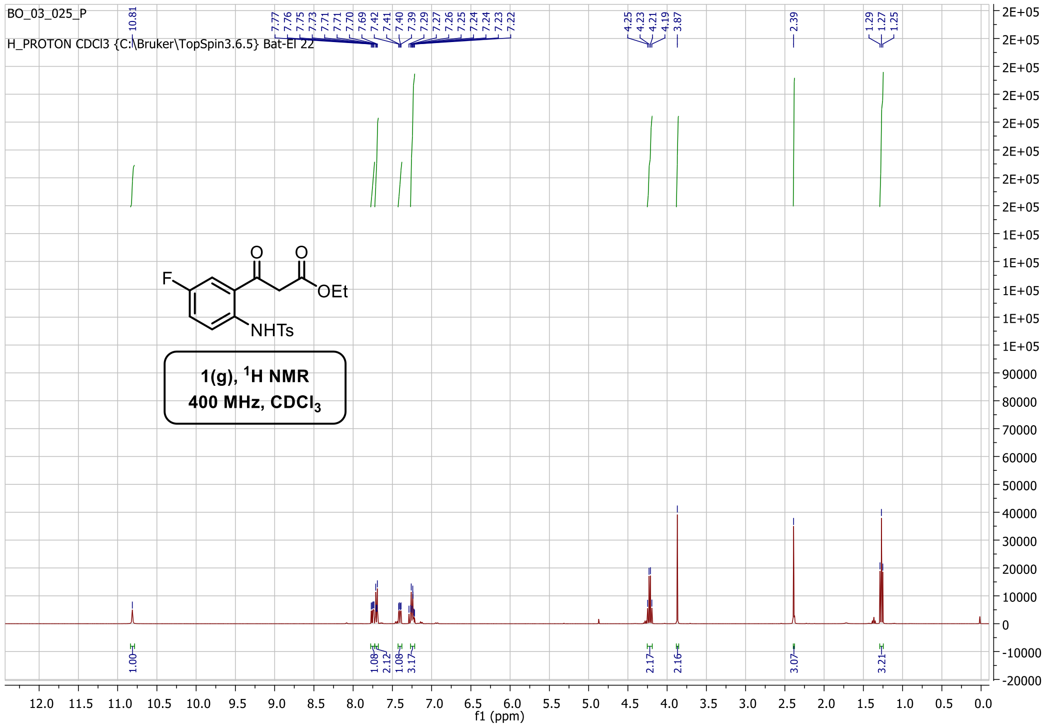

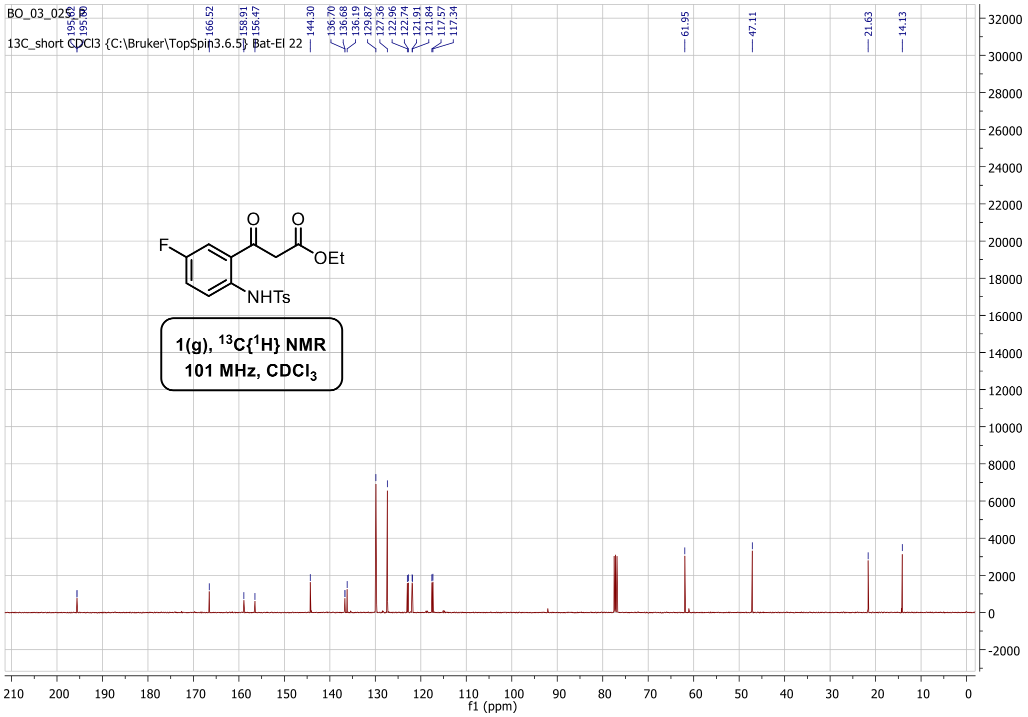

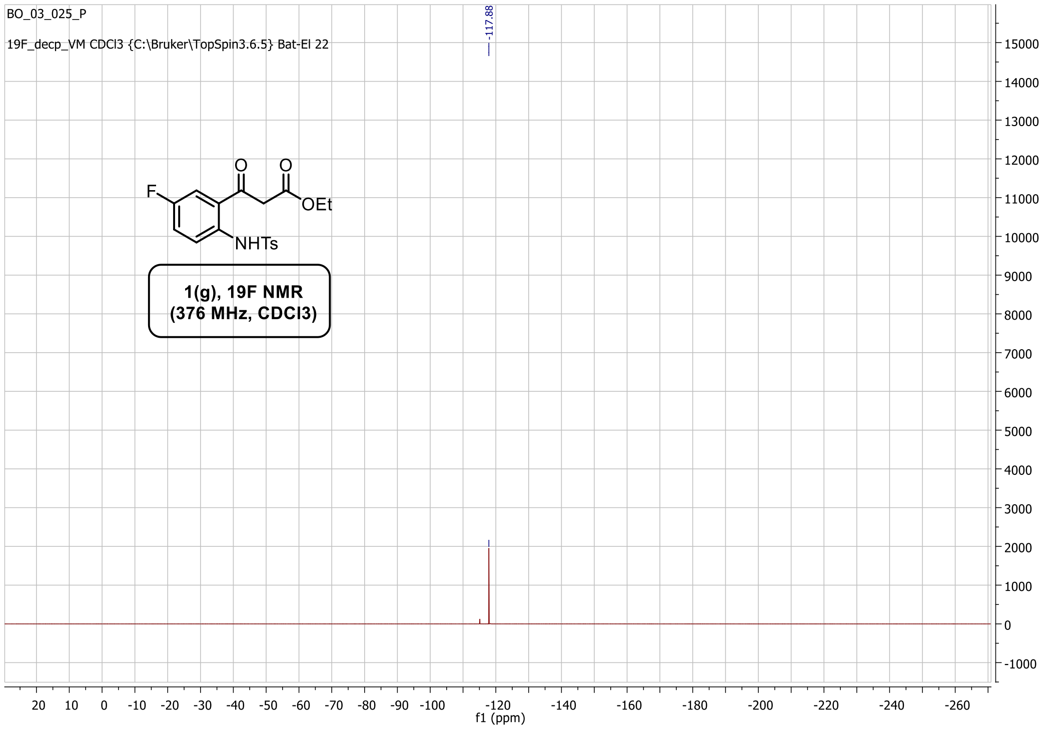

BO_03_025_P
19F_decp_VM CDCl3 {C:\Bruker\TopSpin3.6.5} Bat-El 22
-117.88
F
O
O
OEt
NHTs
1(g), 19F NMR
(376 MHz, CDCl3)
f1 (ppm)

^{1}H and $^{13}C\{^{1}H\}$ NMR spectra of **1(h)**

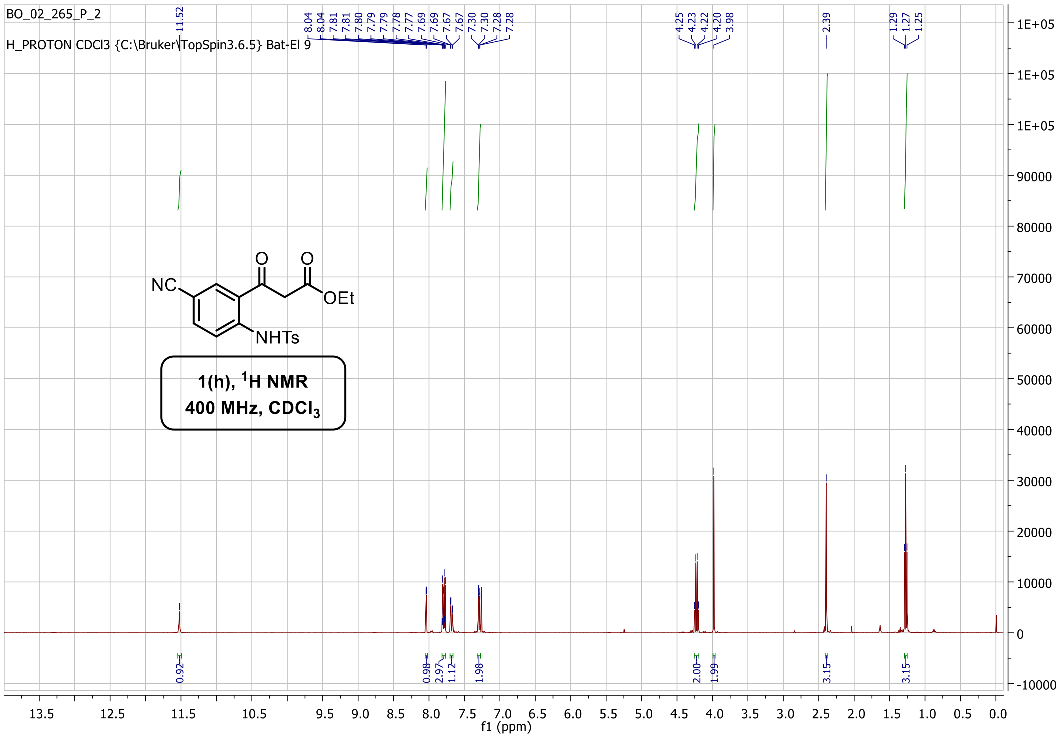

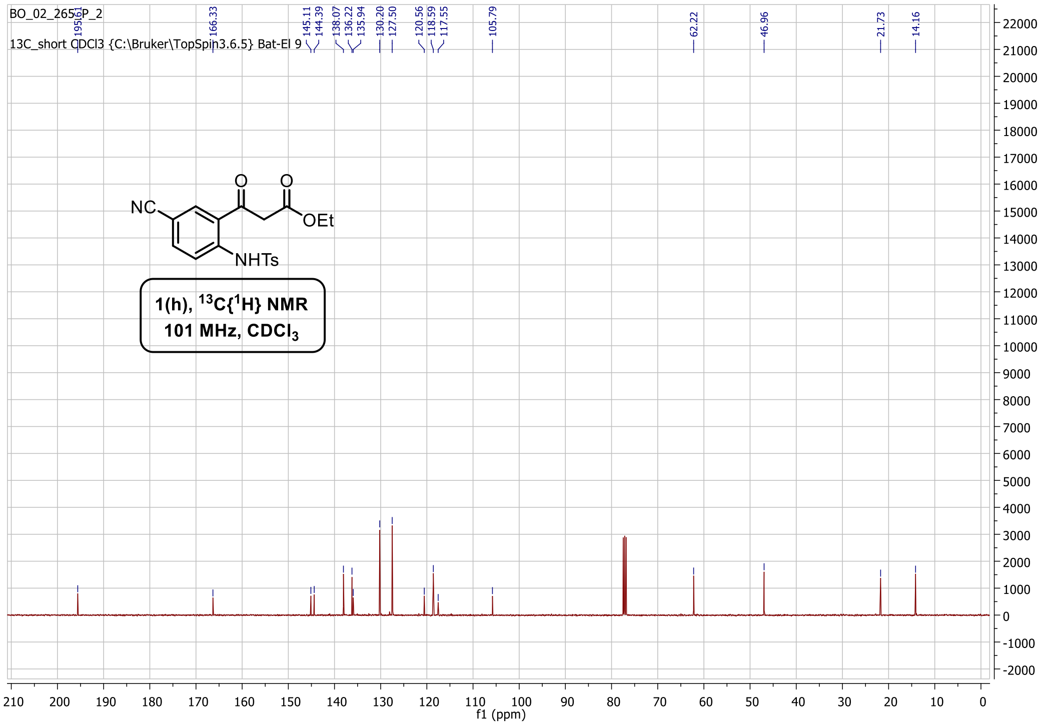

^{1}H and $^{13}C\{^{1}H\}$ NMR spectra of **1(i)**

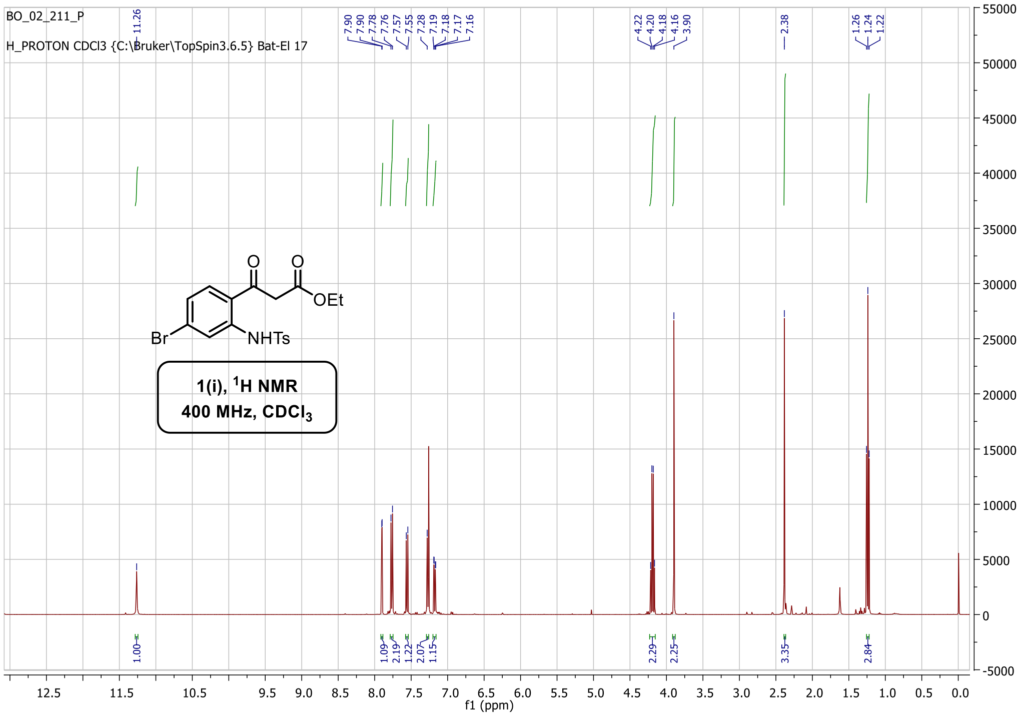

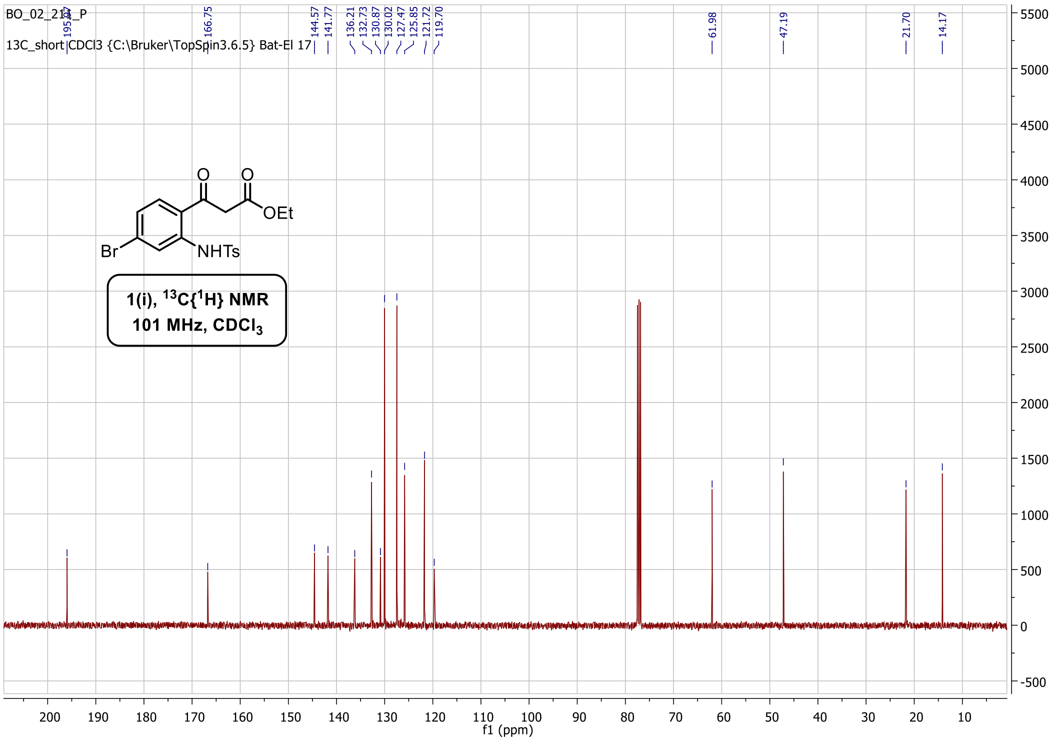

^{1}H and ^{13}C{^{1}H} NMR spectra of **1(j)**

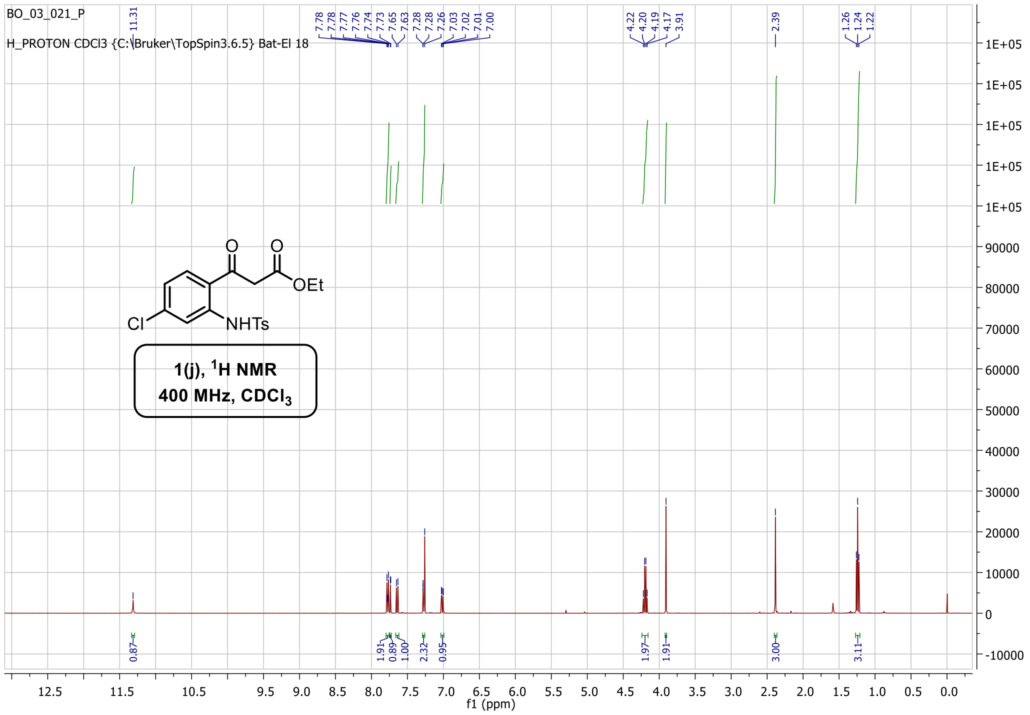

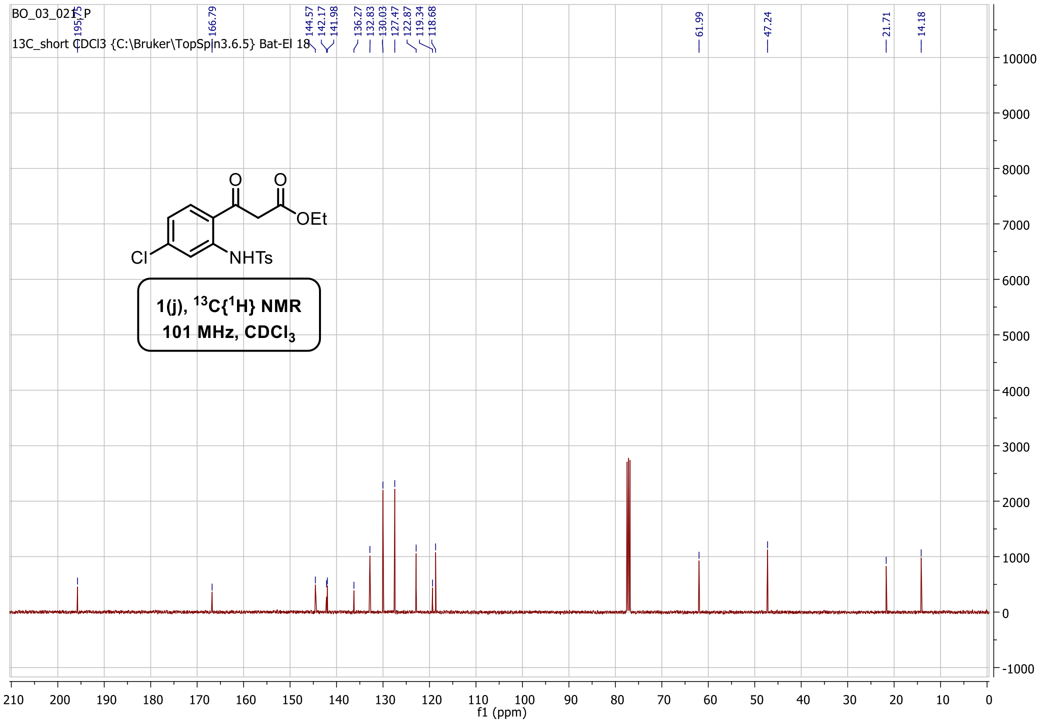

^{1}H, ^{13}C{^{1}H} and ^{19}F NMR spectra of **1(k)**

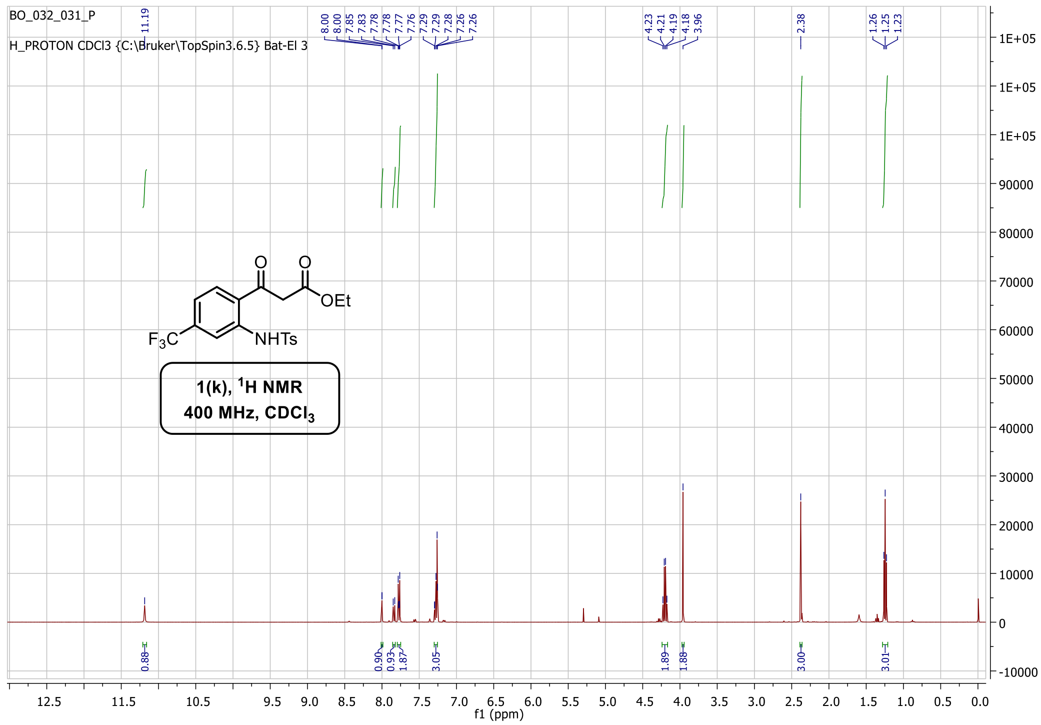

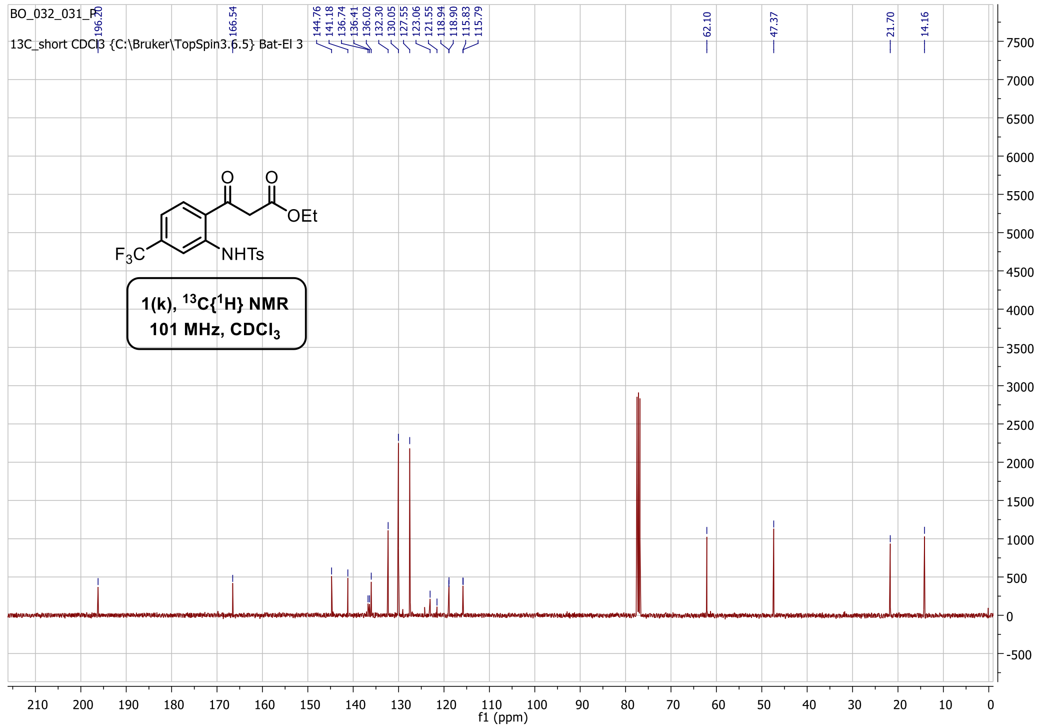

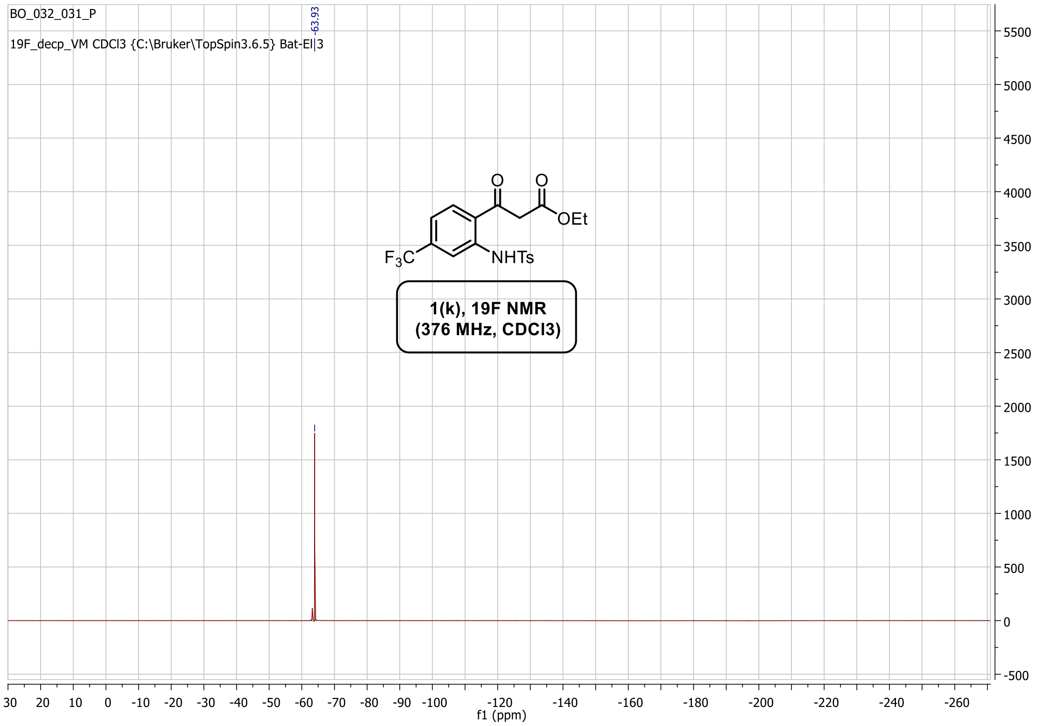
BO_032_031_P
19F_decp_VM CDCl3 {C:\Bruker\TopSpin3.6.5} Bat-El 3
-63.93
O
O
OEt
F3C
NHTs
1(k), 19F NMR
(376 MHz, CDCl3)
f1 (ppm)

^{1}H and $^{13}C\{^{1}H\}$ NMR spectra of **1(l)**

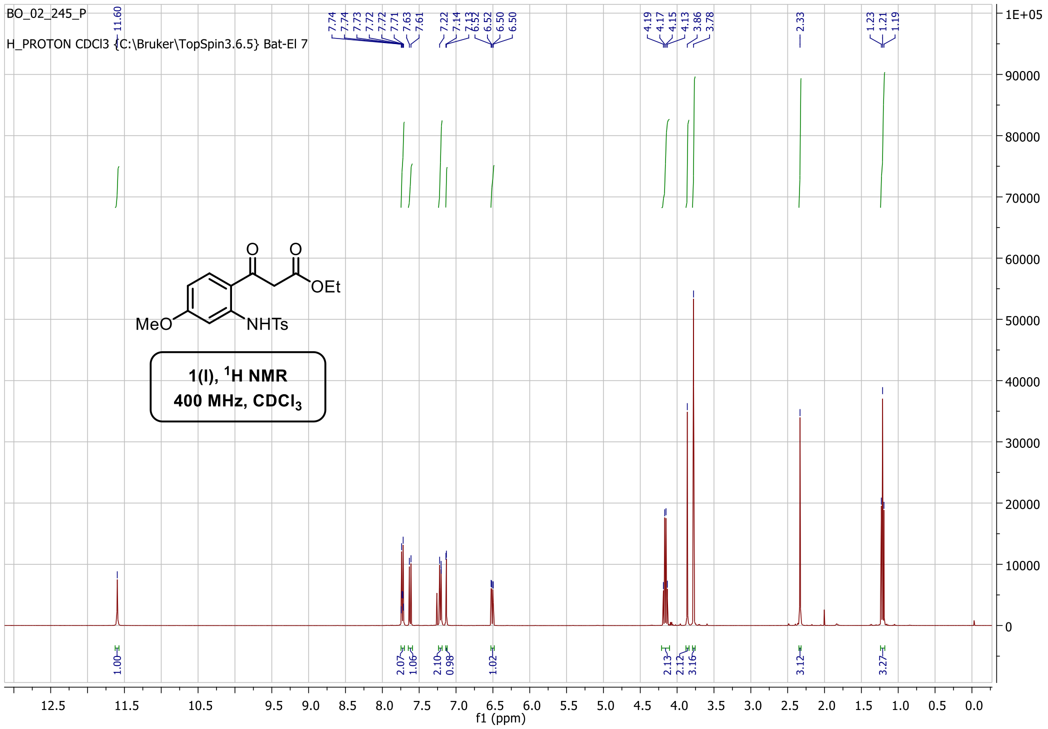

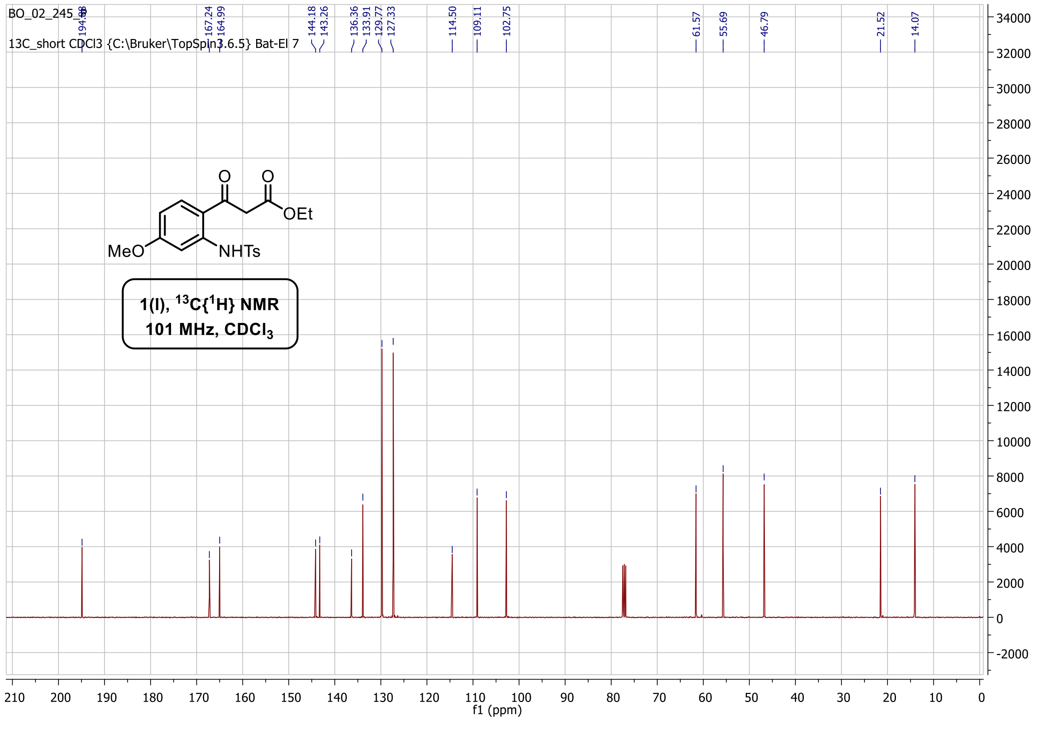

^{1}H and ^{13}C{^{1}H} NMR spectra of **1(m)**

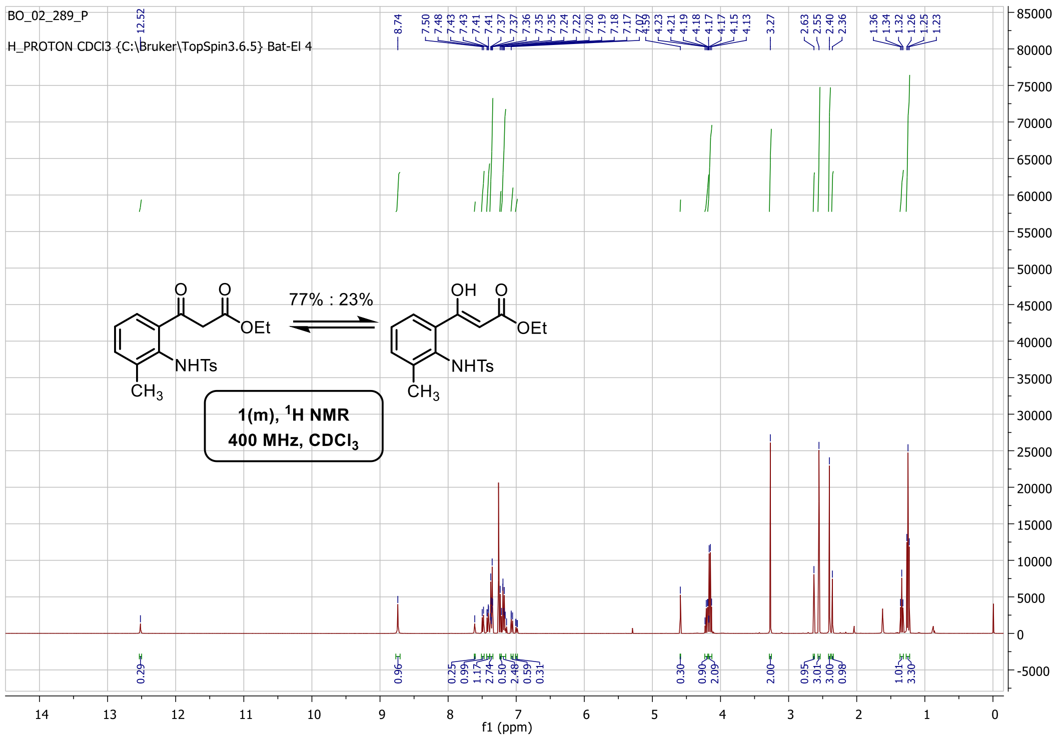

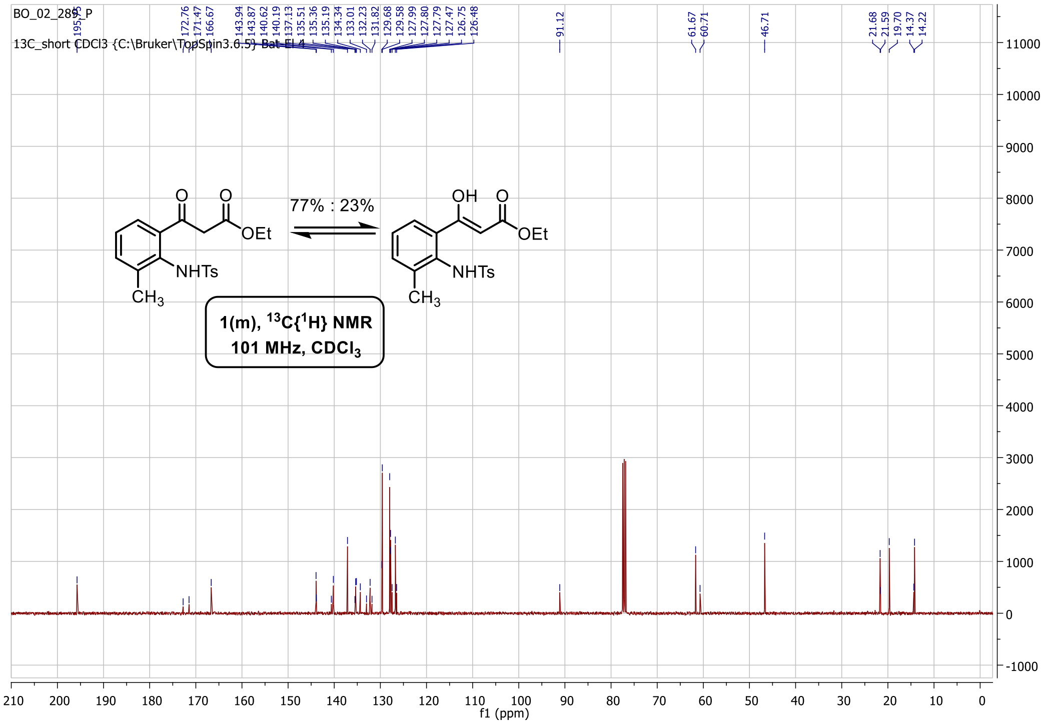

^{1}H and $^{13}C\{^{1}H\}$ NMR spectra of **1(n)**

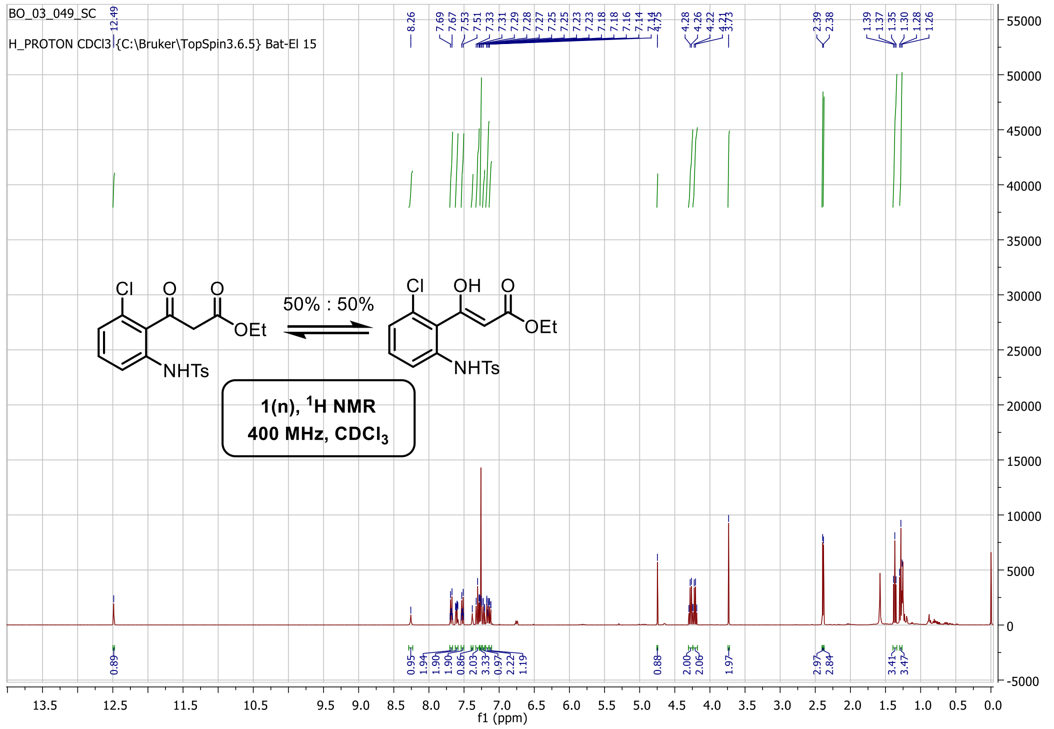

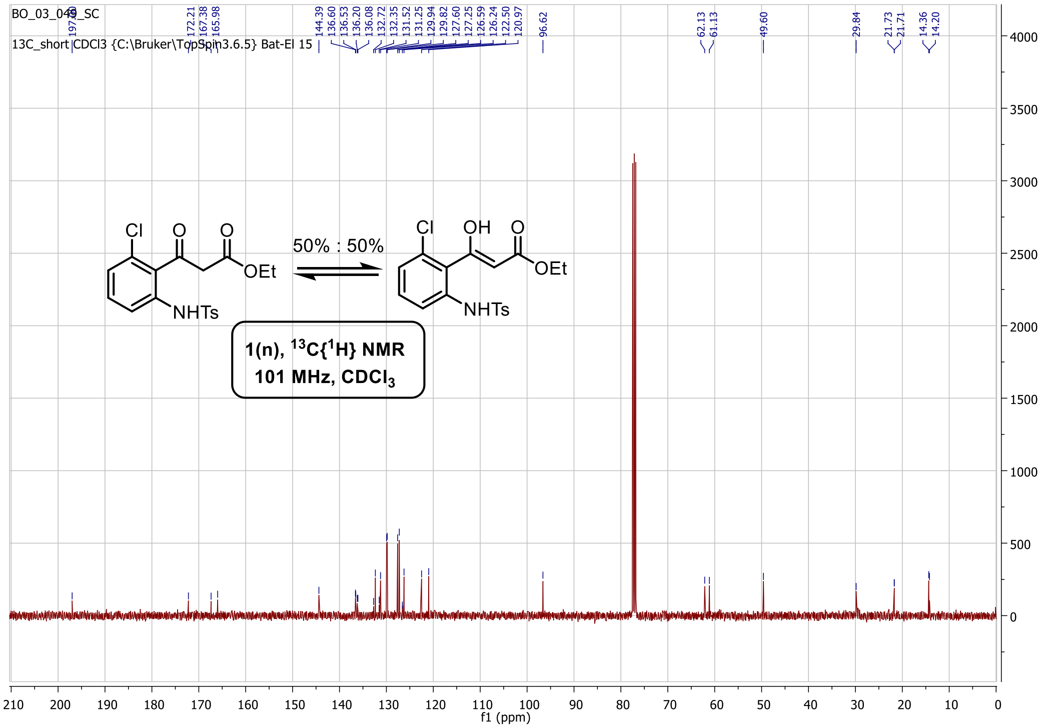

^{1}H and $^{13}C\{^{1}H\}$ NMR spectra of **1(o)**

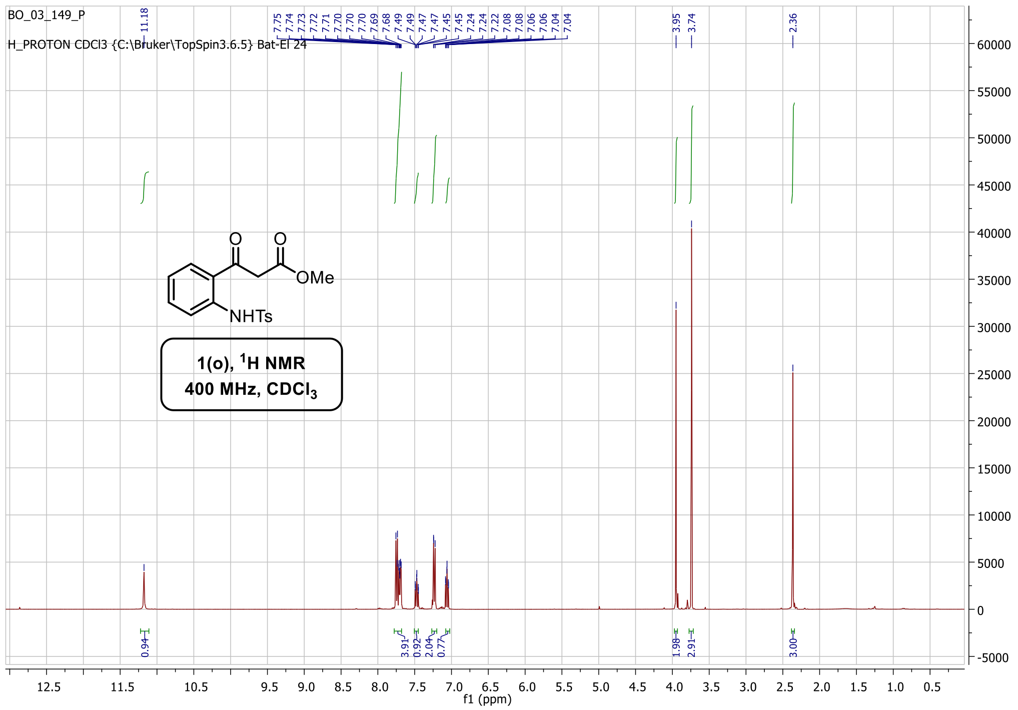

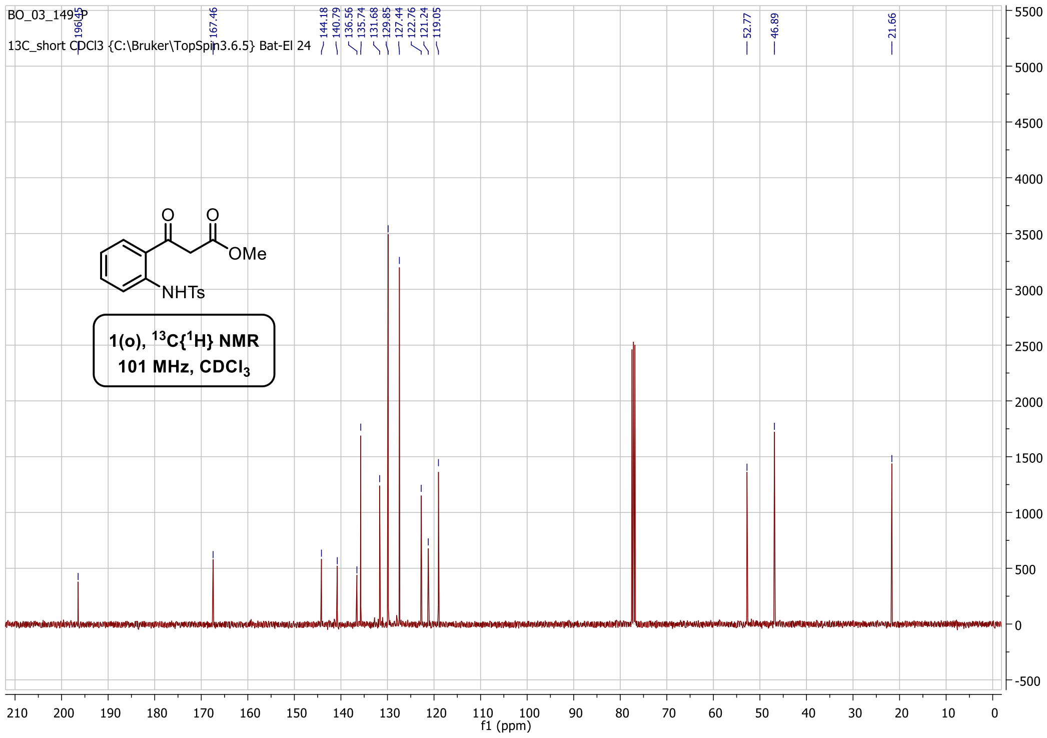

^{1}H and $^{13}C\{^{1}H\}$ NMR spectra of **1(p)**

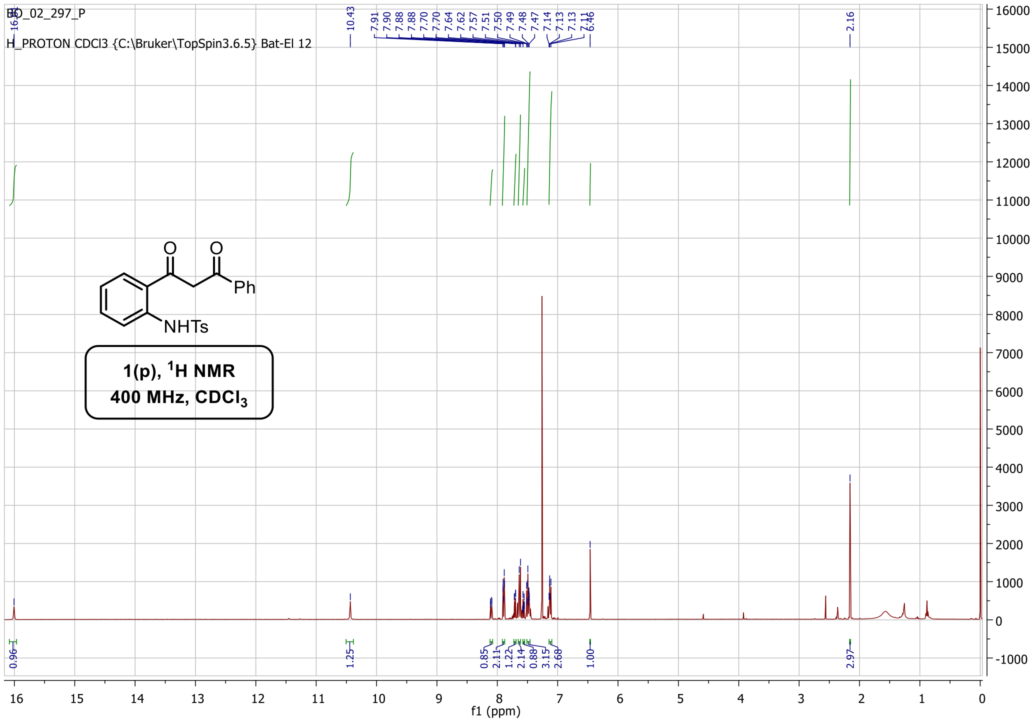

^{1}H and ^{13}C{^{1}H} NMR spectra of **2(a)**

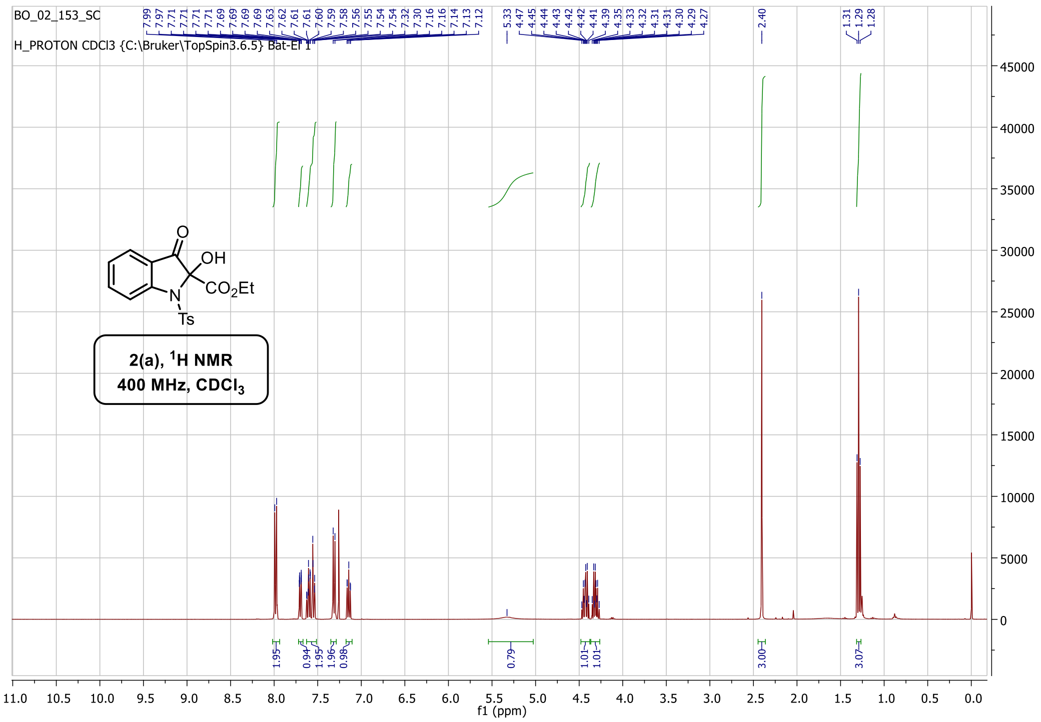

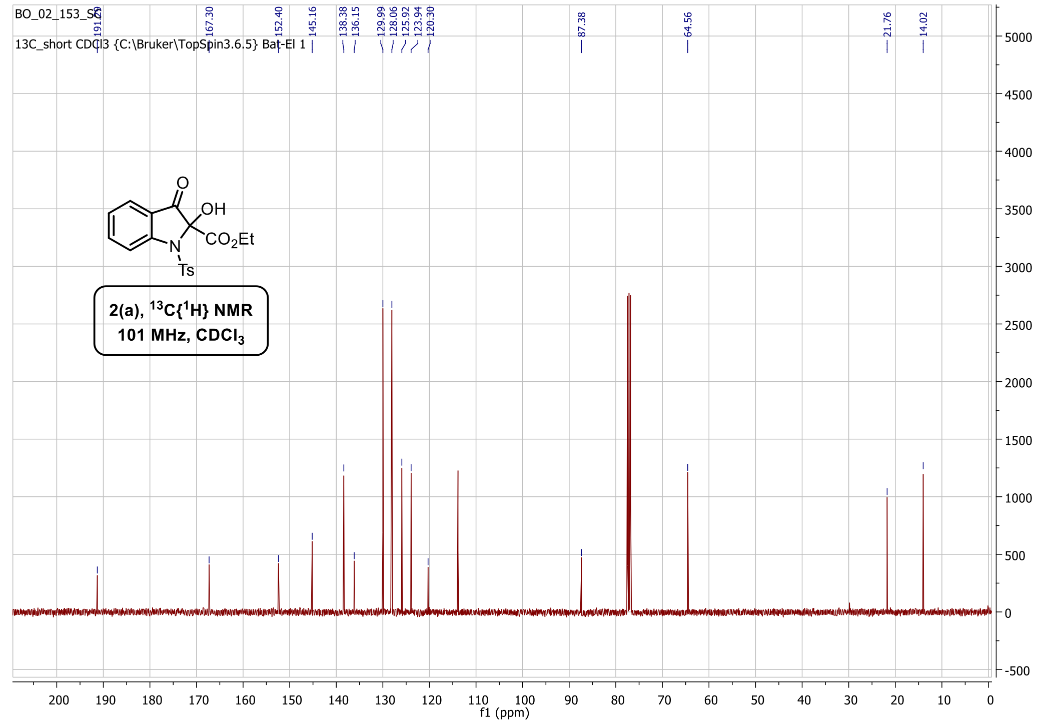

^{1}H and $^{13}C\{^{1}H\}$ NMR spectra of **2(b)**

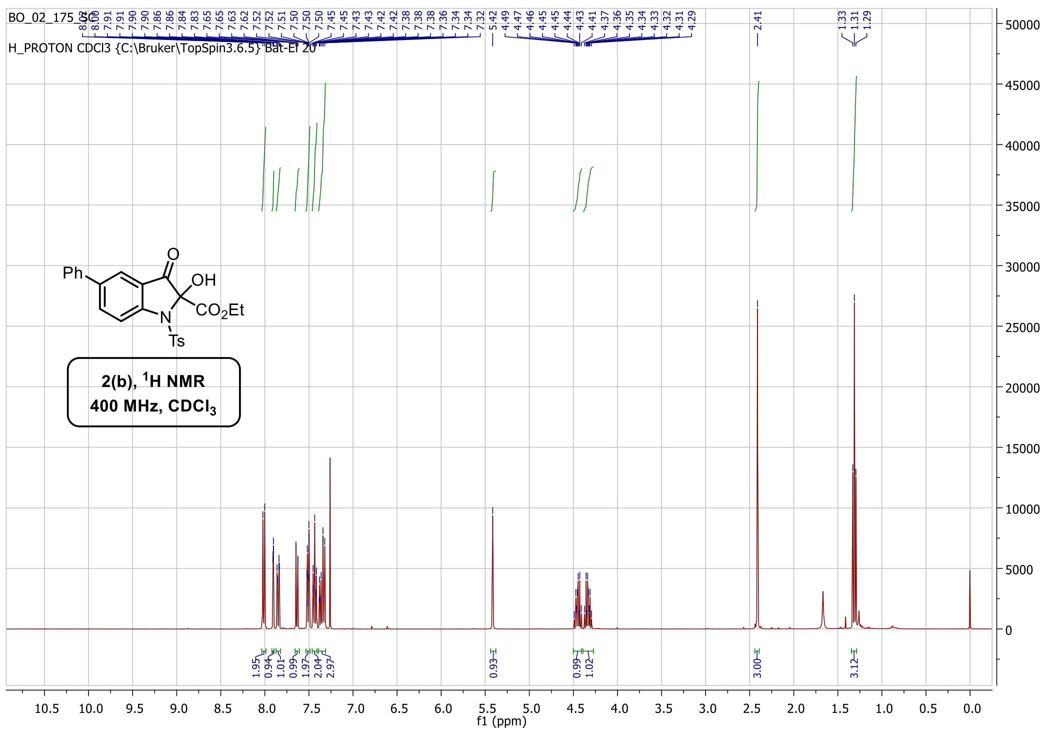

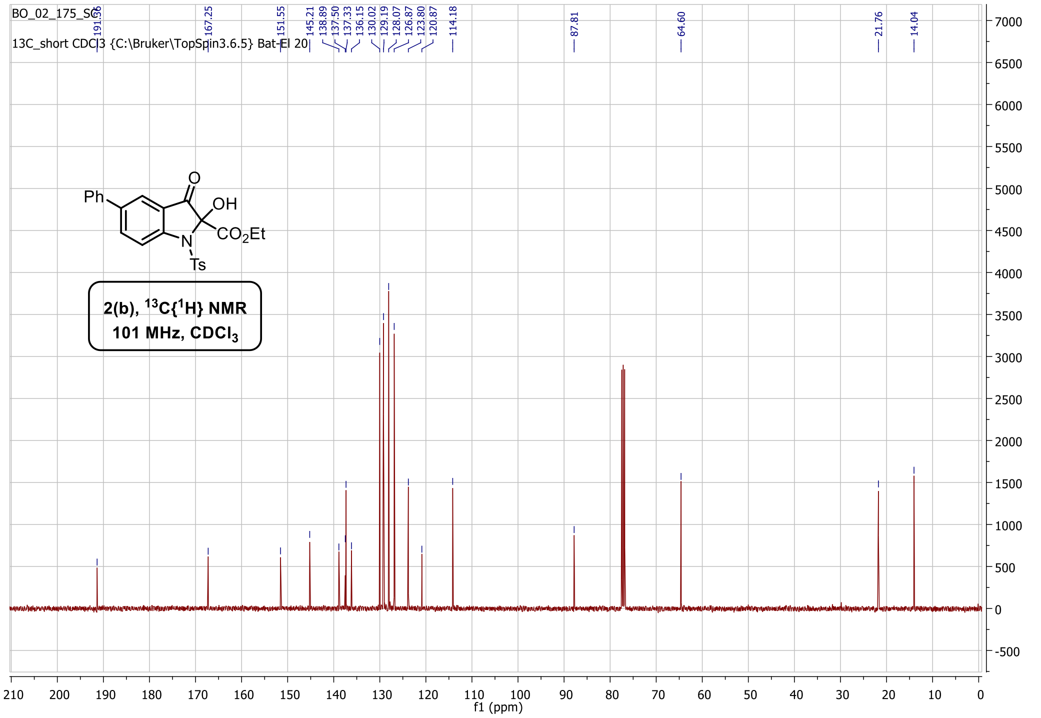

^{1}H and ^{13}C{^{1}H} NMR spectra of **2(c)**

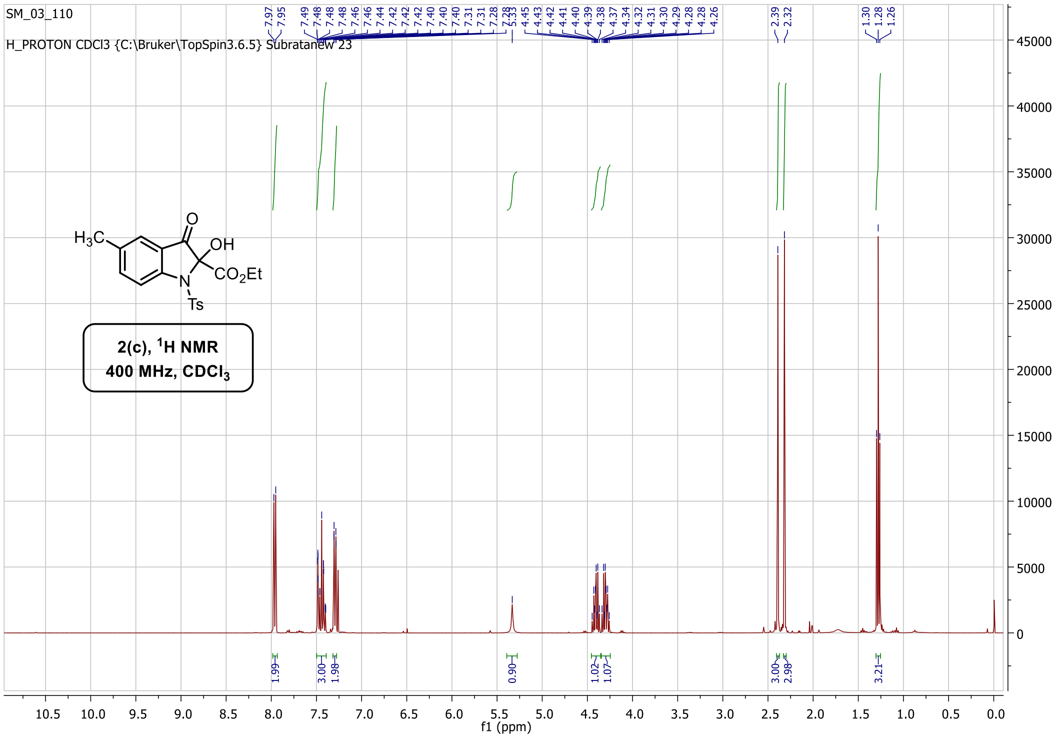

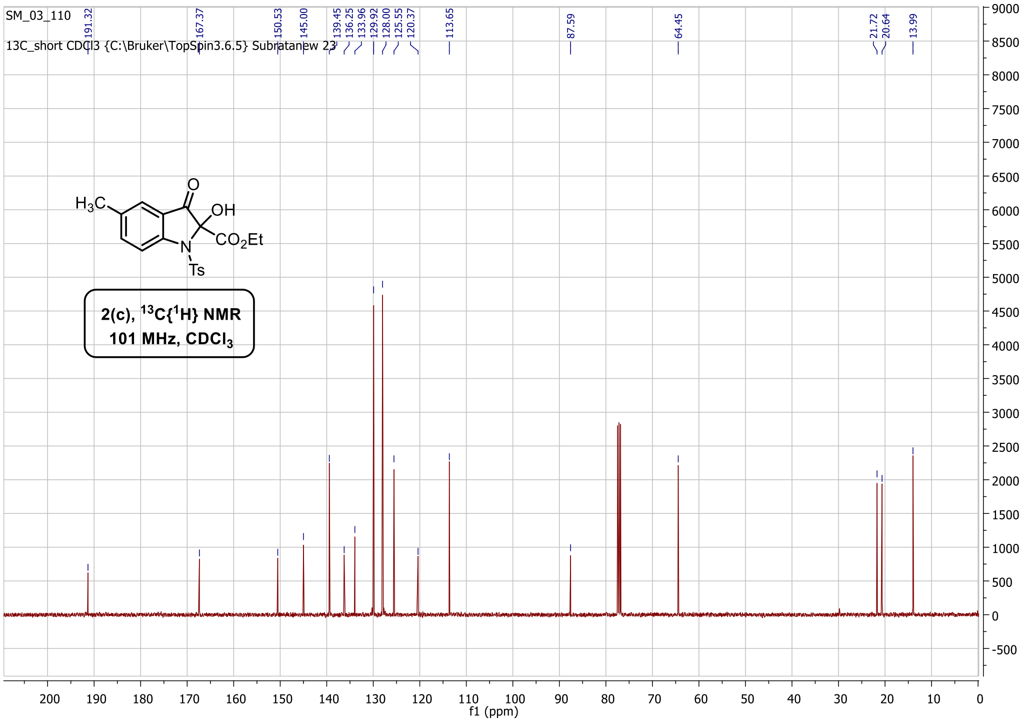

^{1}H and $^{13}C\{^{1}H\}$ NMR spectra of **2(d)**

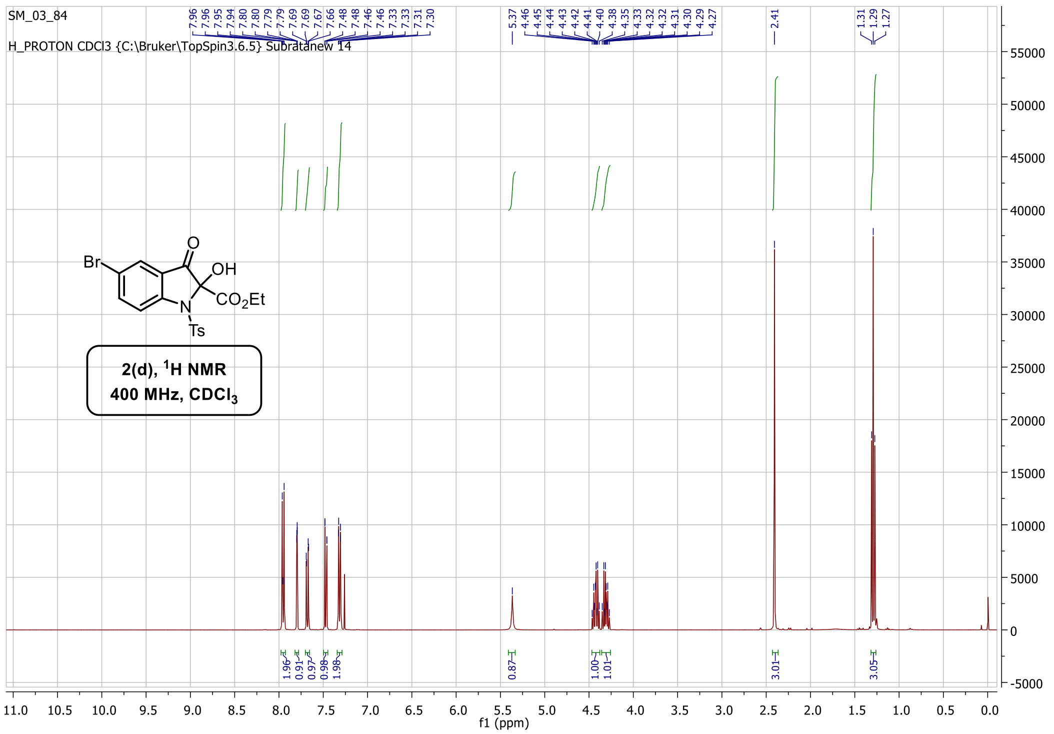

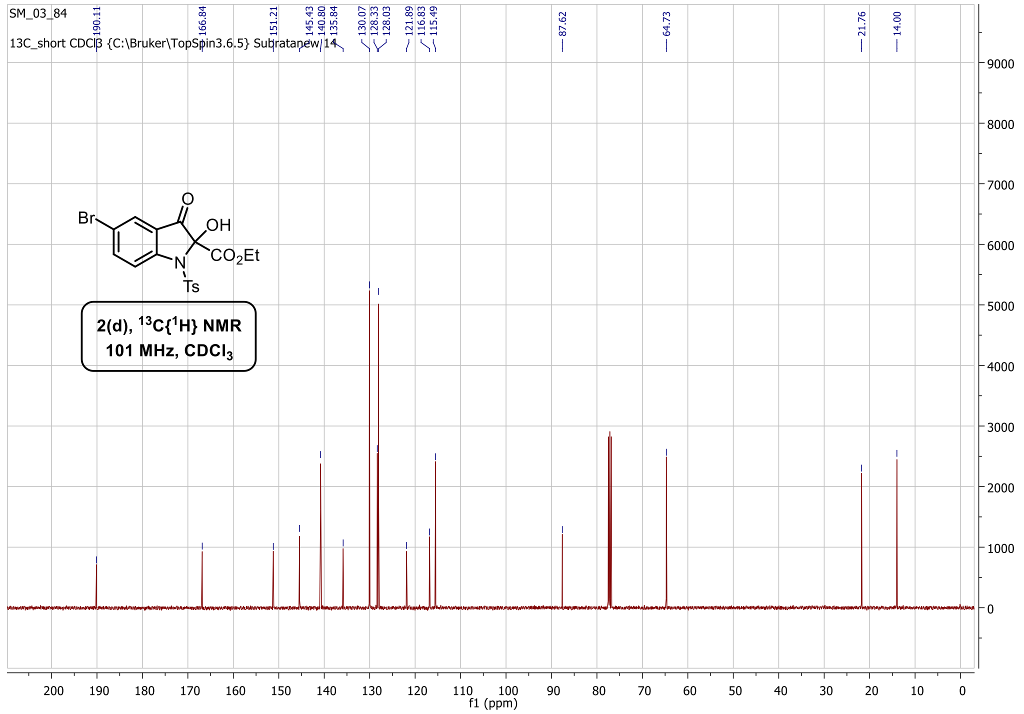

^{1}H and $^{13}C\{^{1}H\}$ NMR spectra of **2(e)**

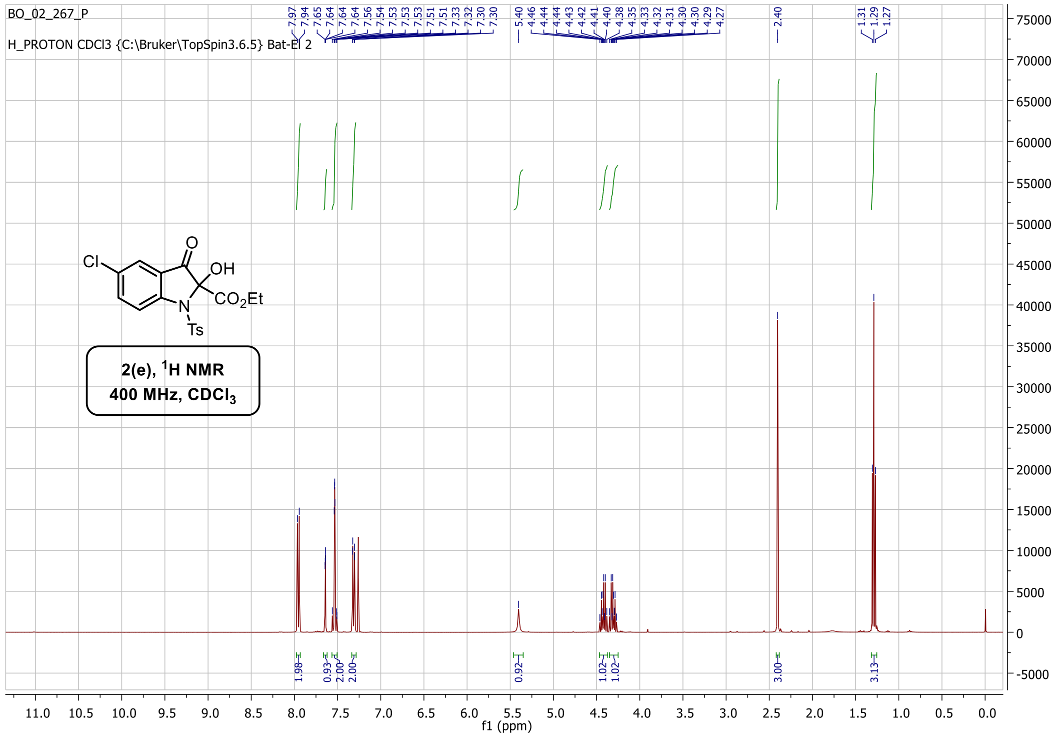

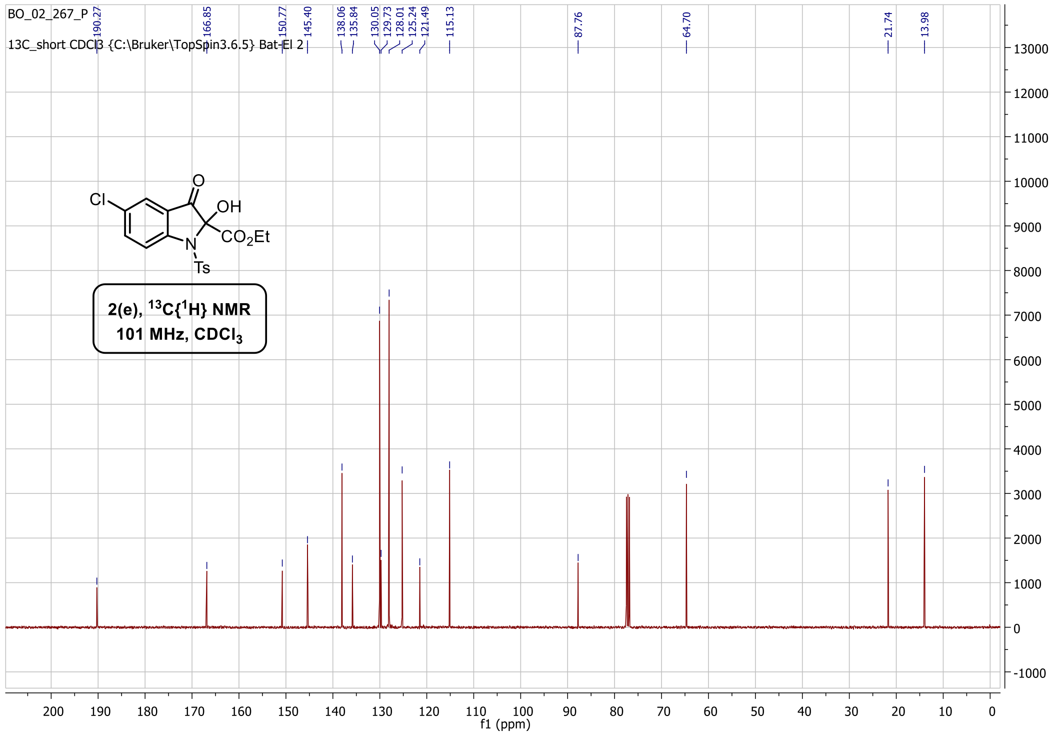

^{1}H and ^{13}C{^{1}H} NMR spectra of **2(f)**

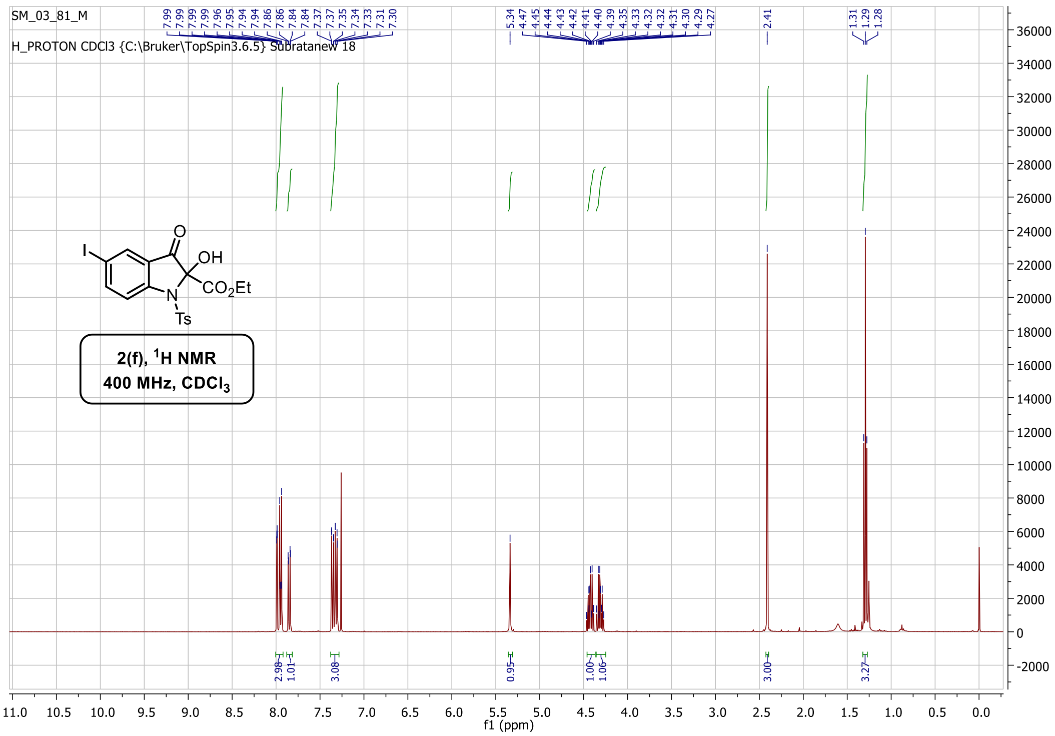

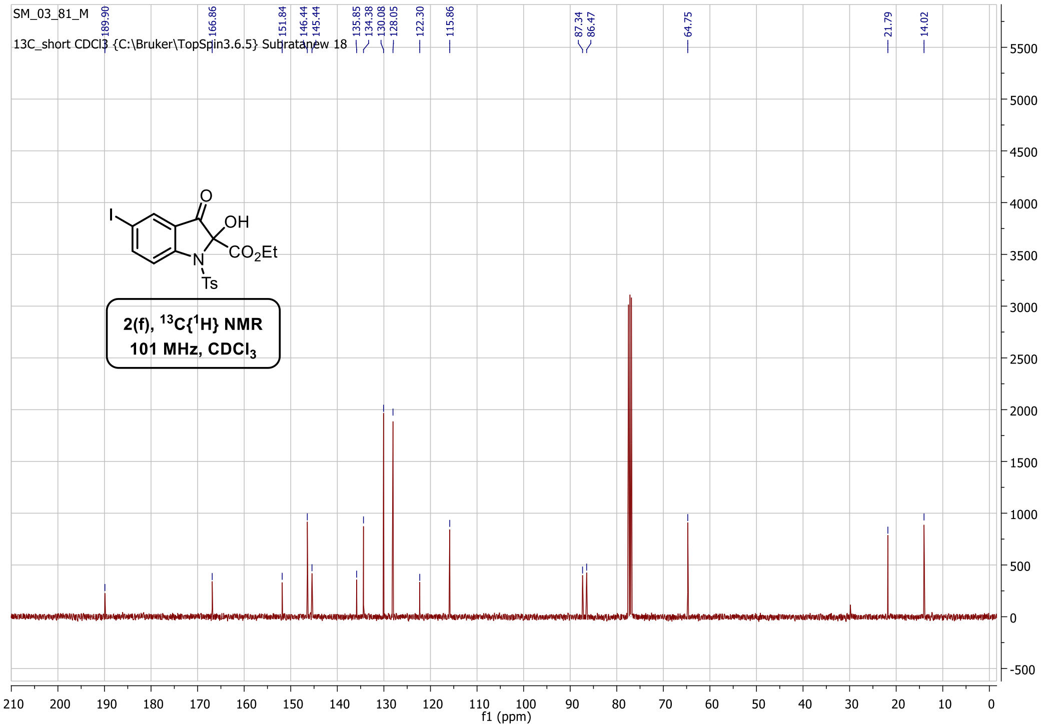

^{1}H, $^{13}C\{^{1}H\}$ and ^{19}F NMR spectra of **2(g)**

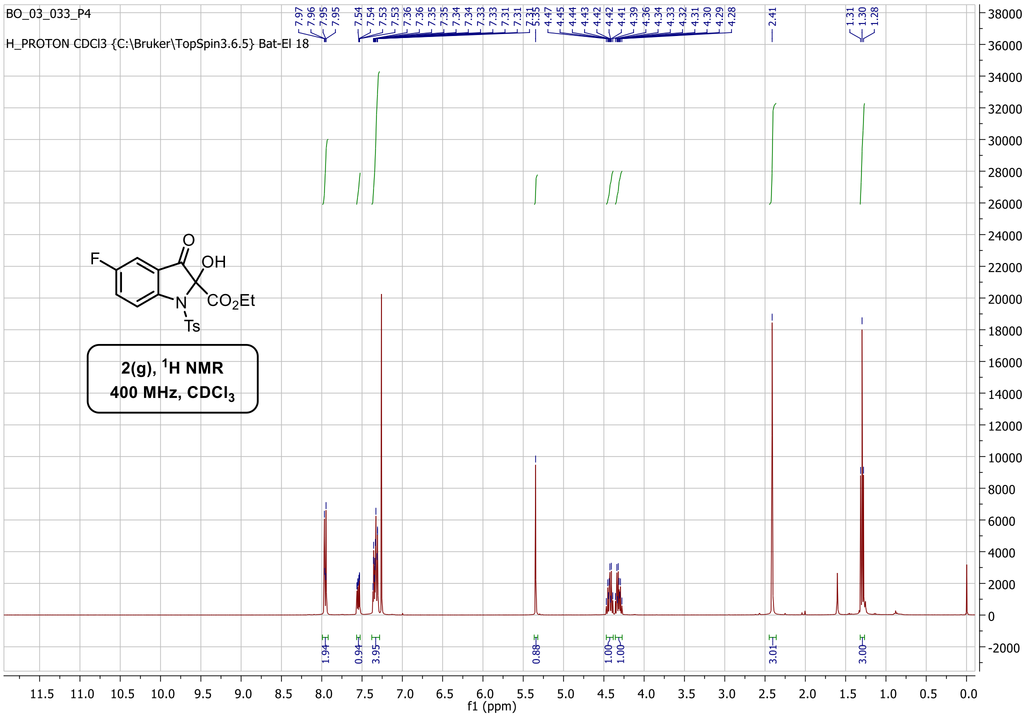

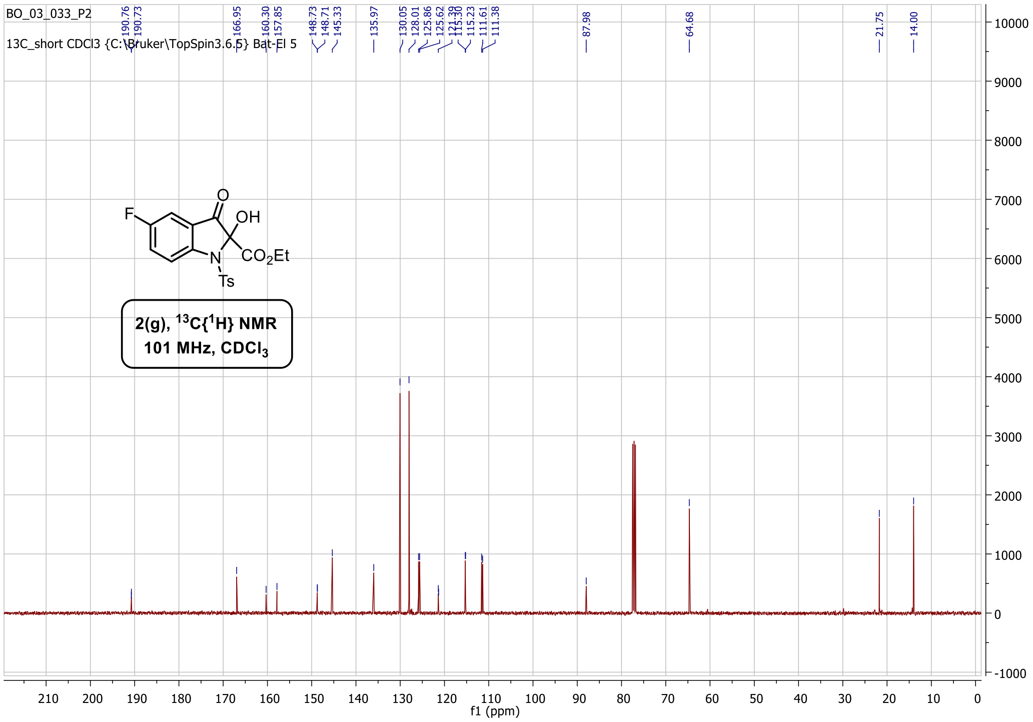

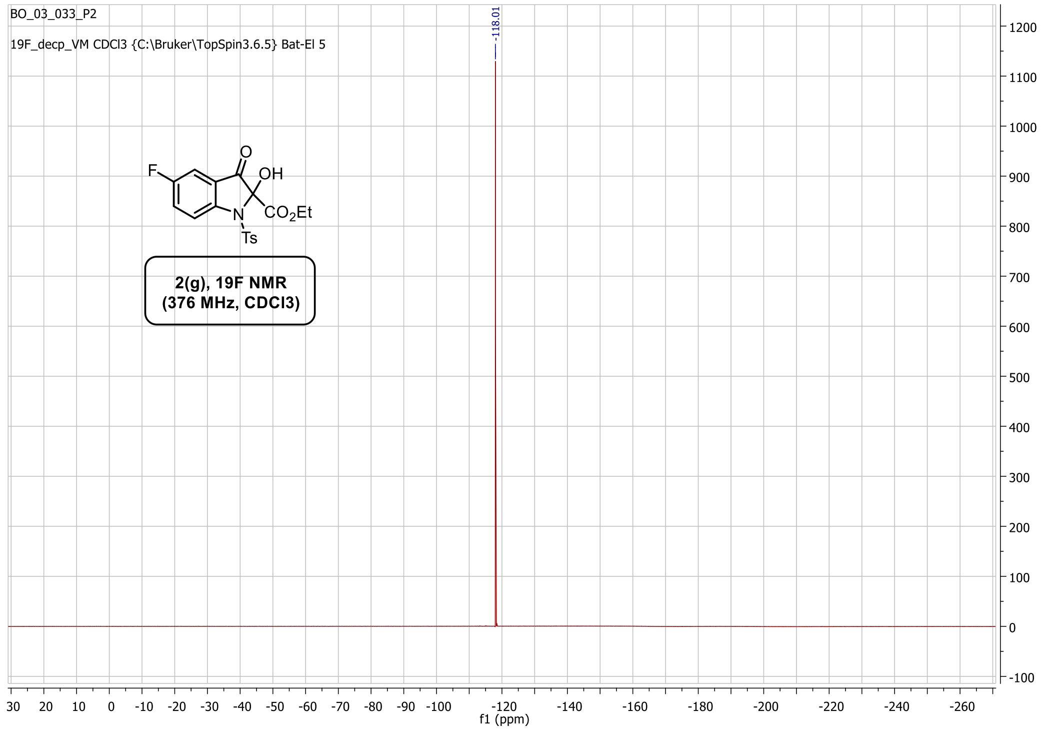
BO_03_033_P2
19F_decp_VM CDCl3 {C:\Bruker\TopSpin3.6.5} Bat-El 5
-118.01
2(g), 19F NMR
(376 MHz, CDCl3)
f1 (ppm)

^1H and $^{13}C\{^1H\}$ NMR spectra of **2(h)**

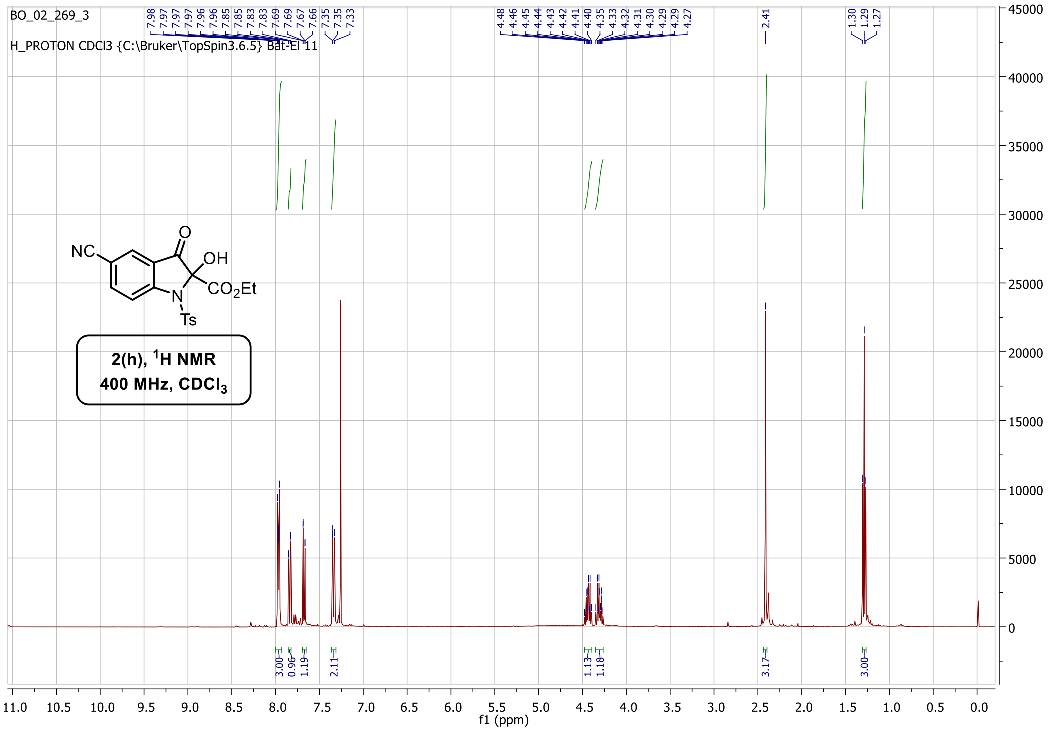

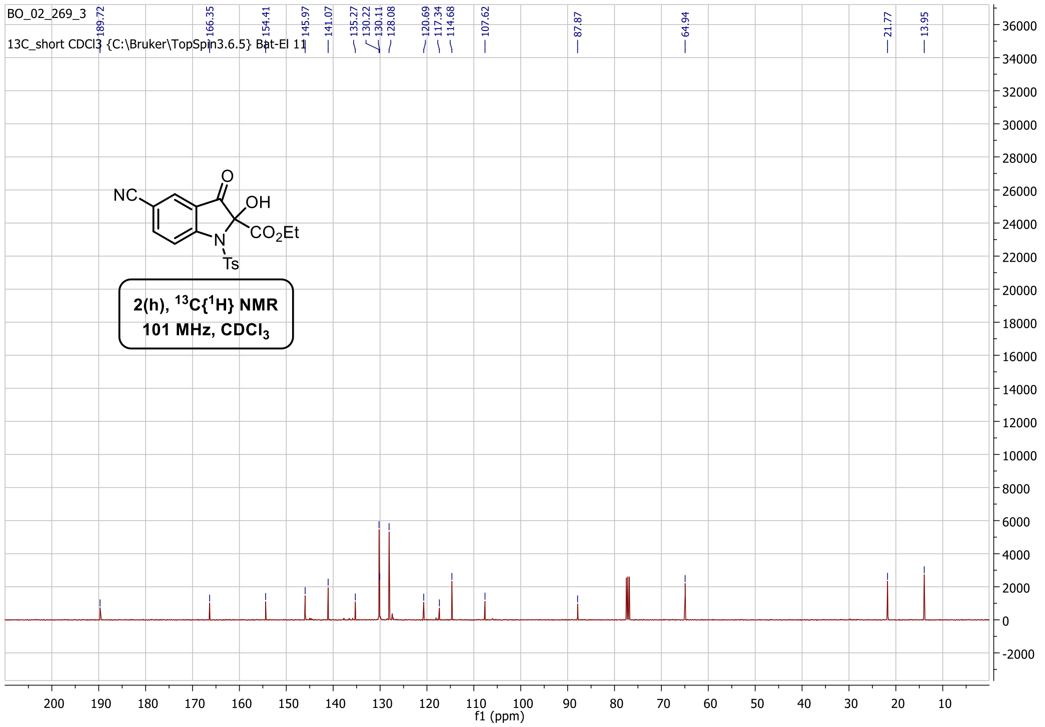

^{1}H and ^{13}C{^{1}H} NMR spectra of **2(i)**

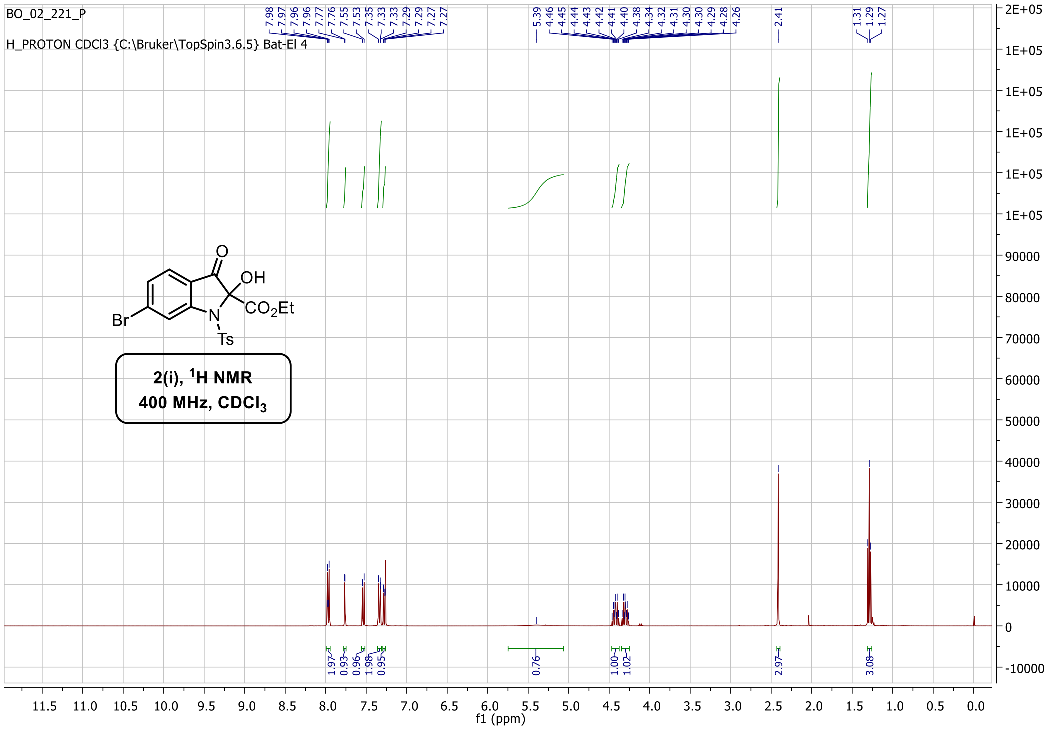

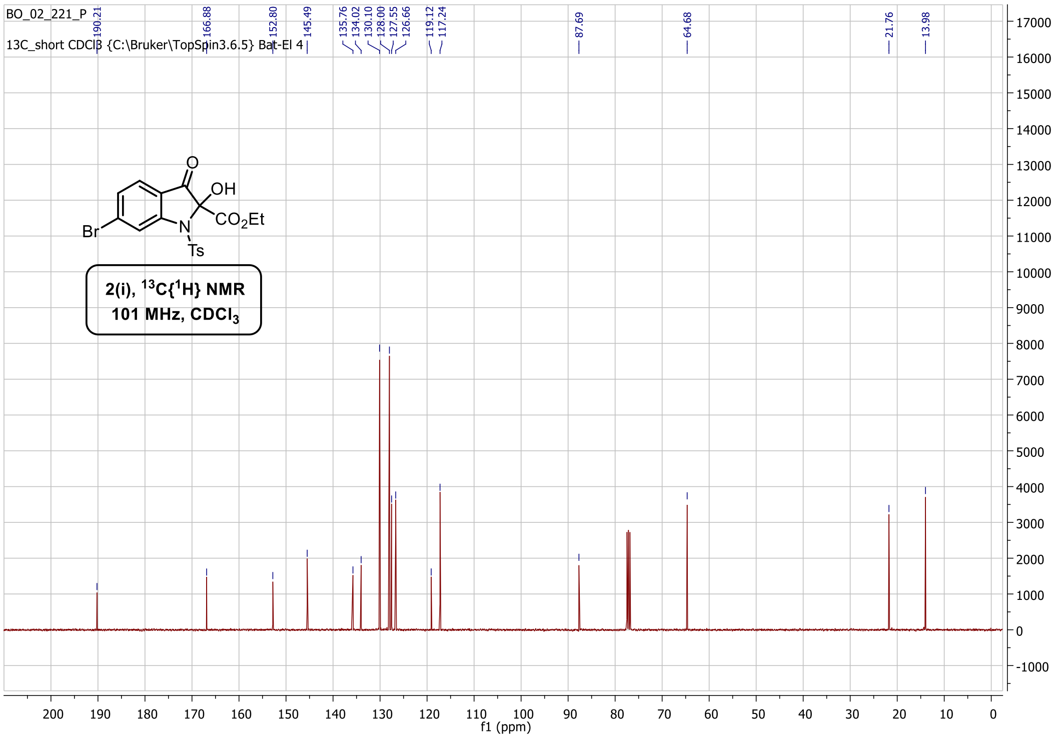

^{1}H and ^{13}C{^{1}H} NMR spectra of **2(j)**

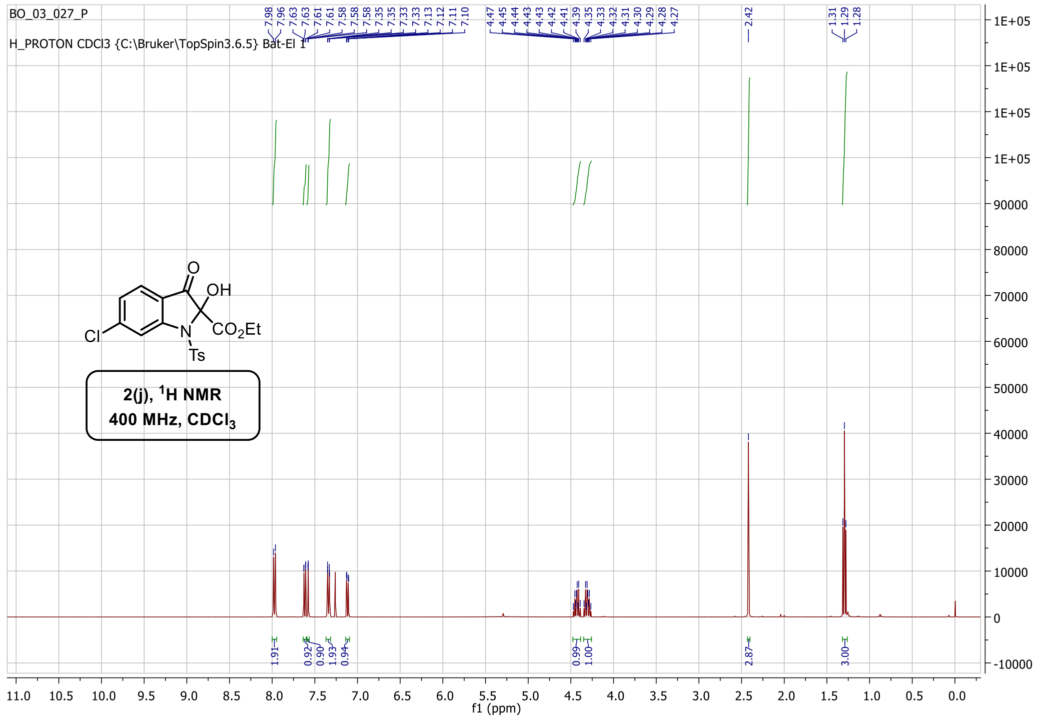

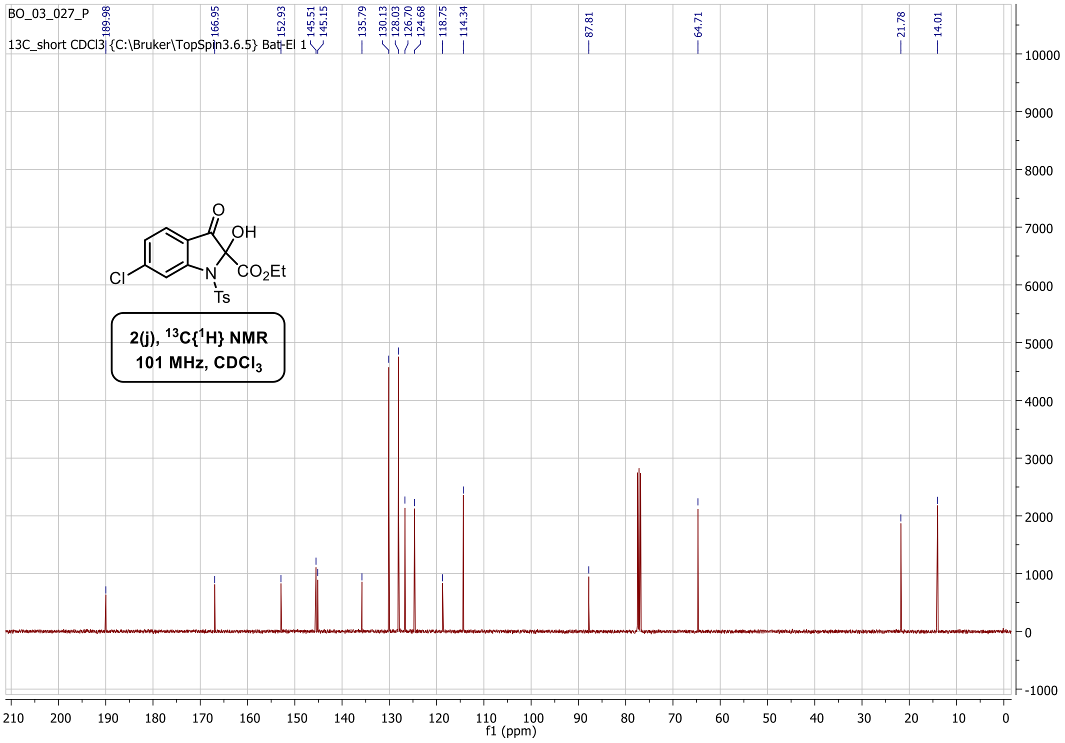

^{1}H, $^{13}C\{^{1}H\}$ and ^{19}F NMR spectra of **2(k)**

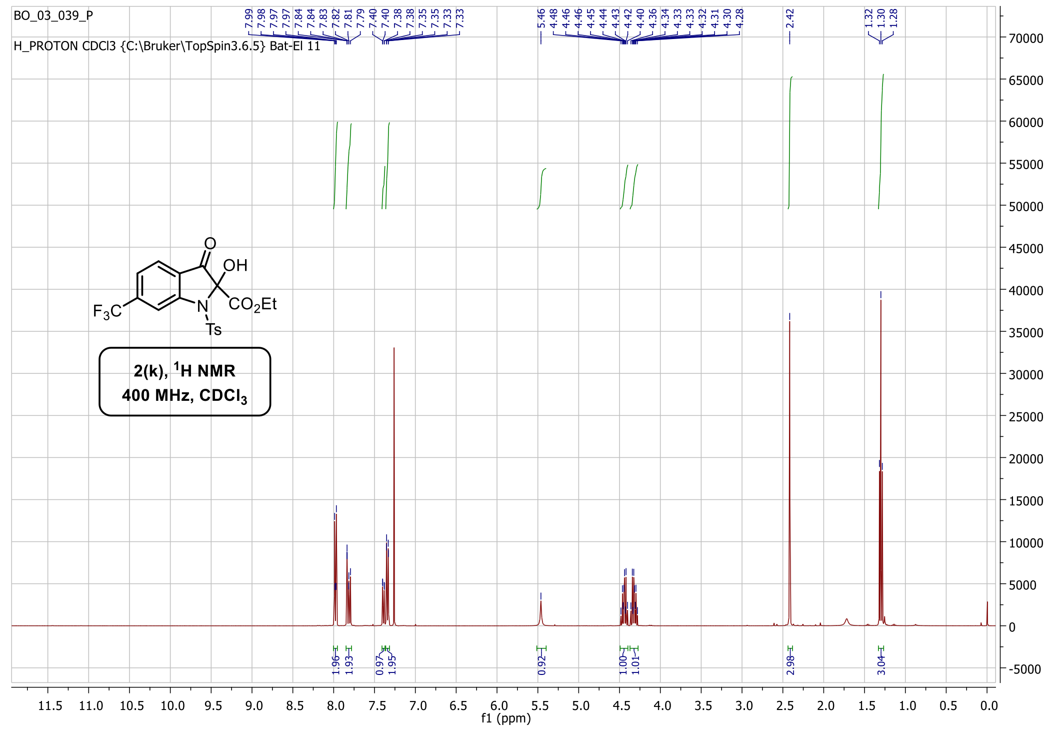

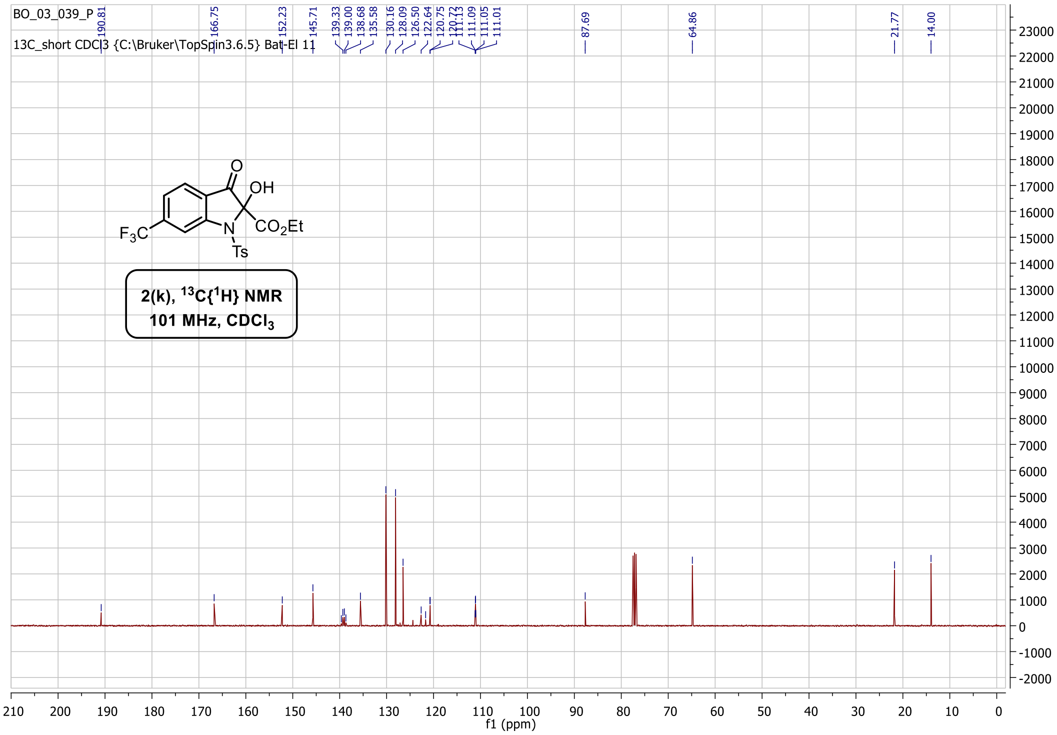

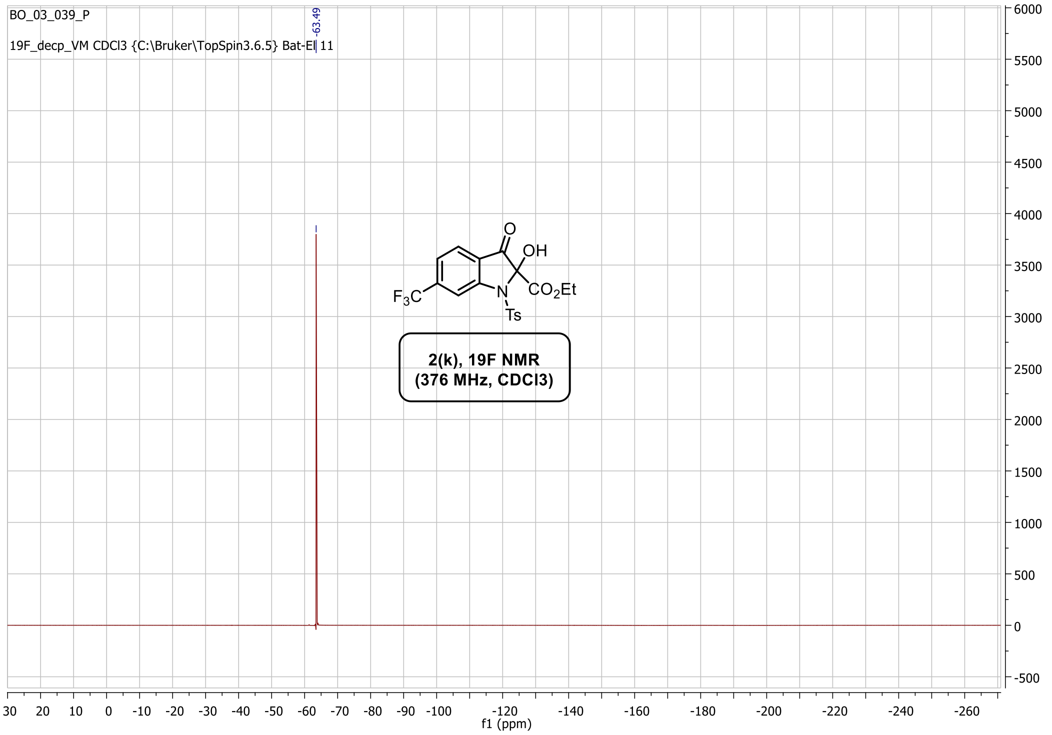
BO_03_039_P
19F_decp_VM CDCl3 {C:\Bruker\TopSpin3.6.5} Bat-El 11
-63.49
2(k), 19F NMR
(376 MHz, CDCl3)
f1 (ppm)

^{1}H and ^{13}C{^{1}H} NMR spectra of **2(l)**

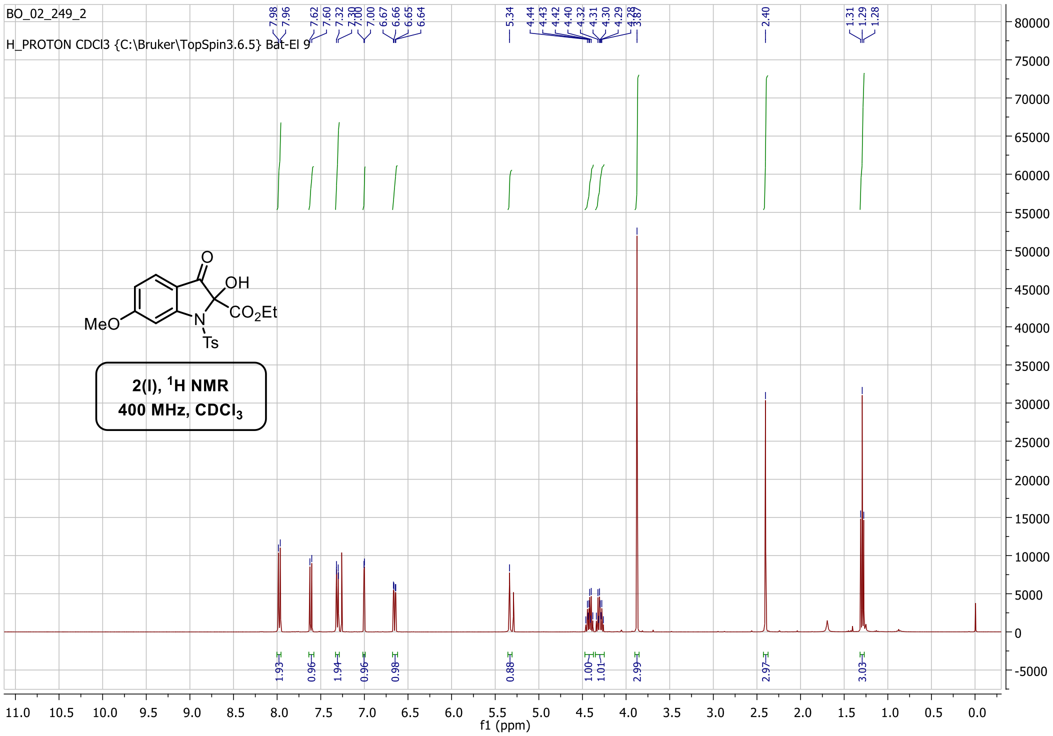

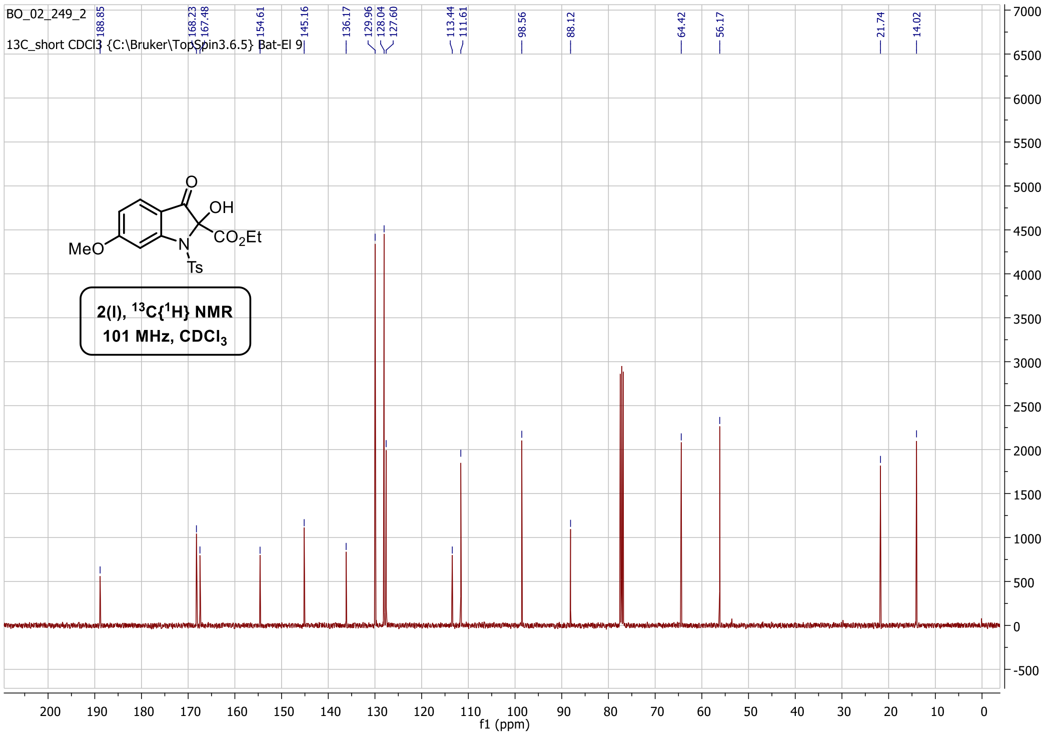

^{1}H and $^{13}C\{^{1}H\}$ NMR spectra of **2(m)**

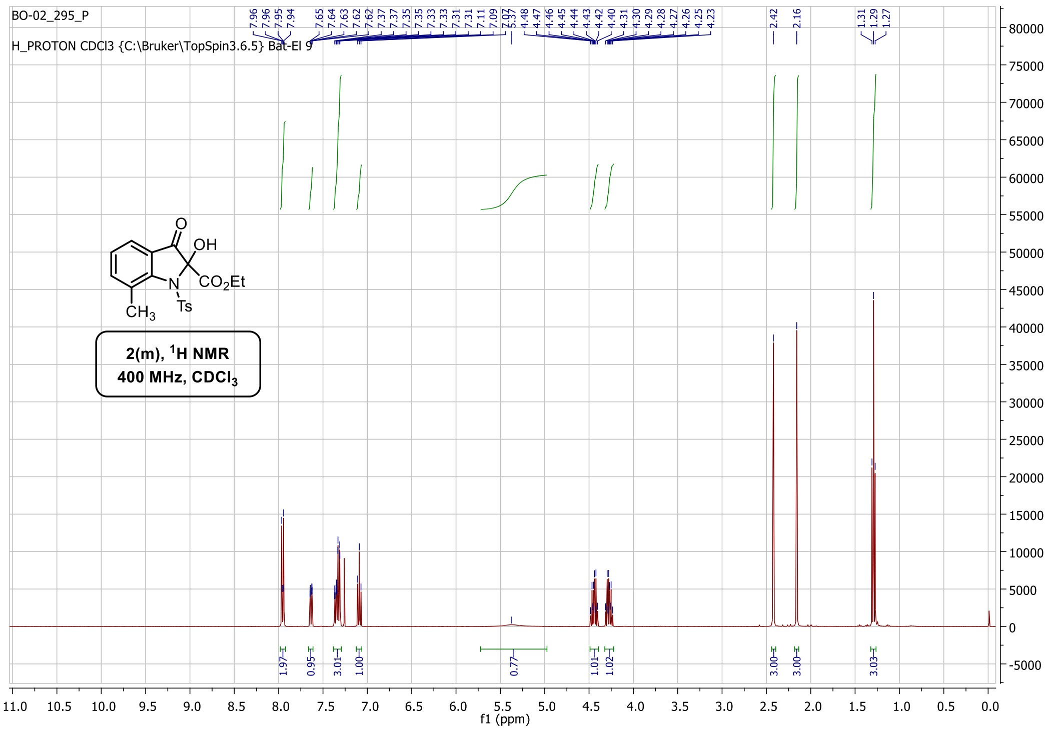

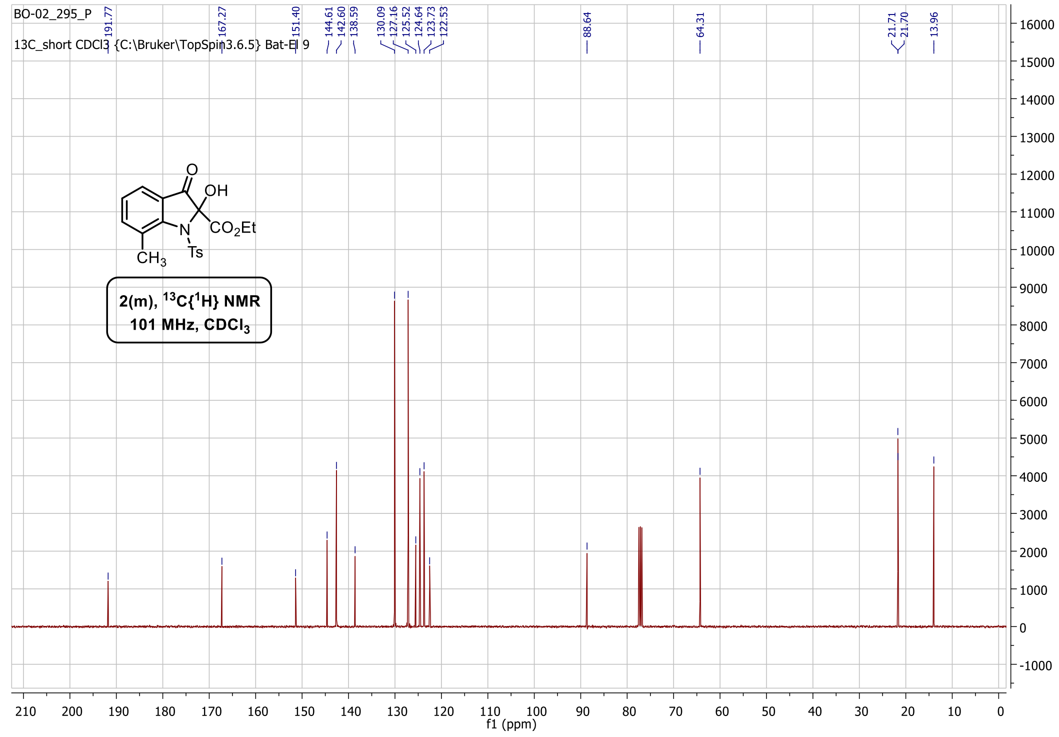

^{1}H and ^{13}C{^{1}H} NMR spectra of **2(n)**

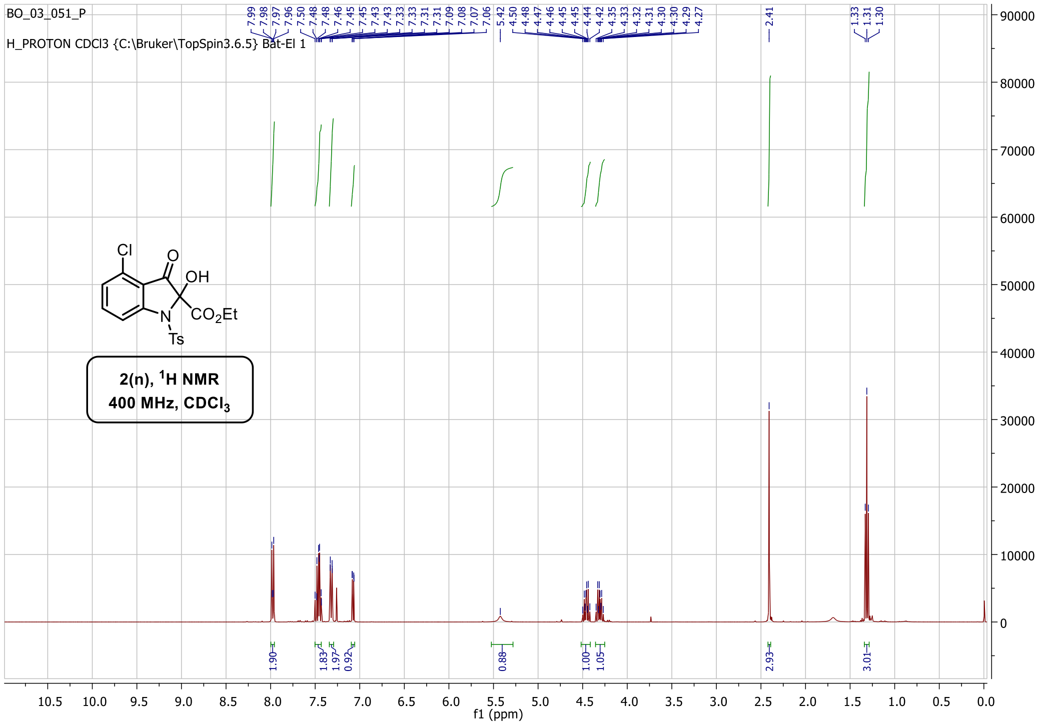

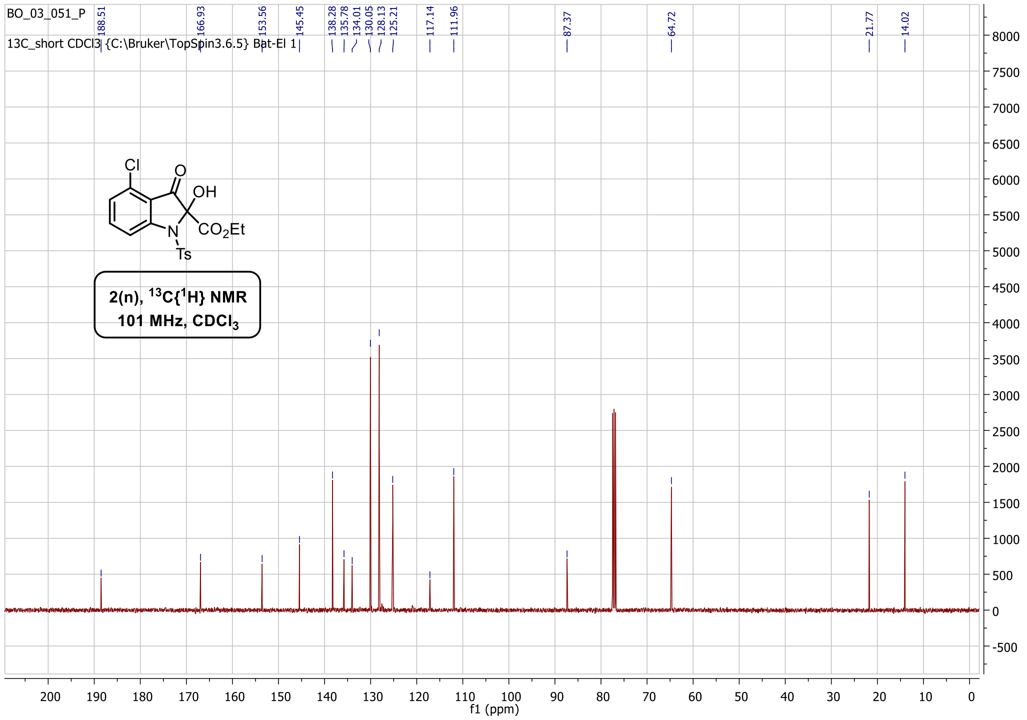

^{1}H and ^{13}C{^{1}H} NMR spectra of **2(o)**

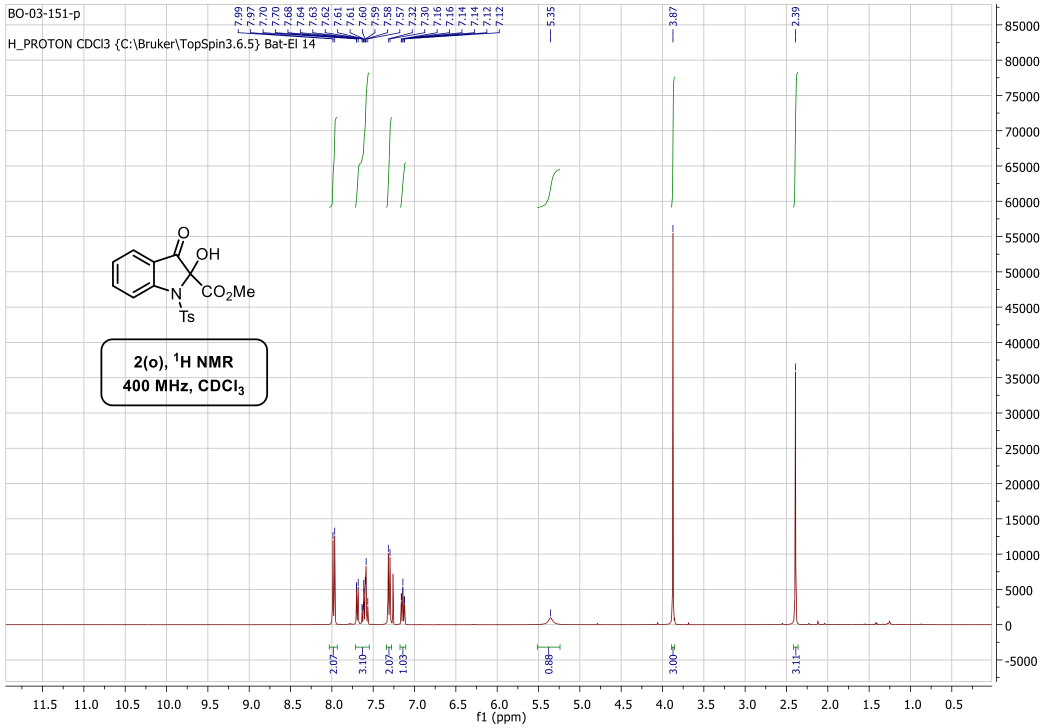

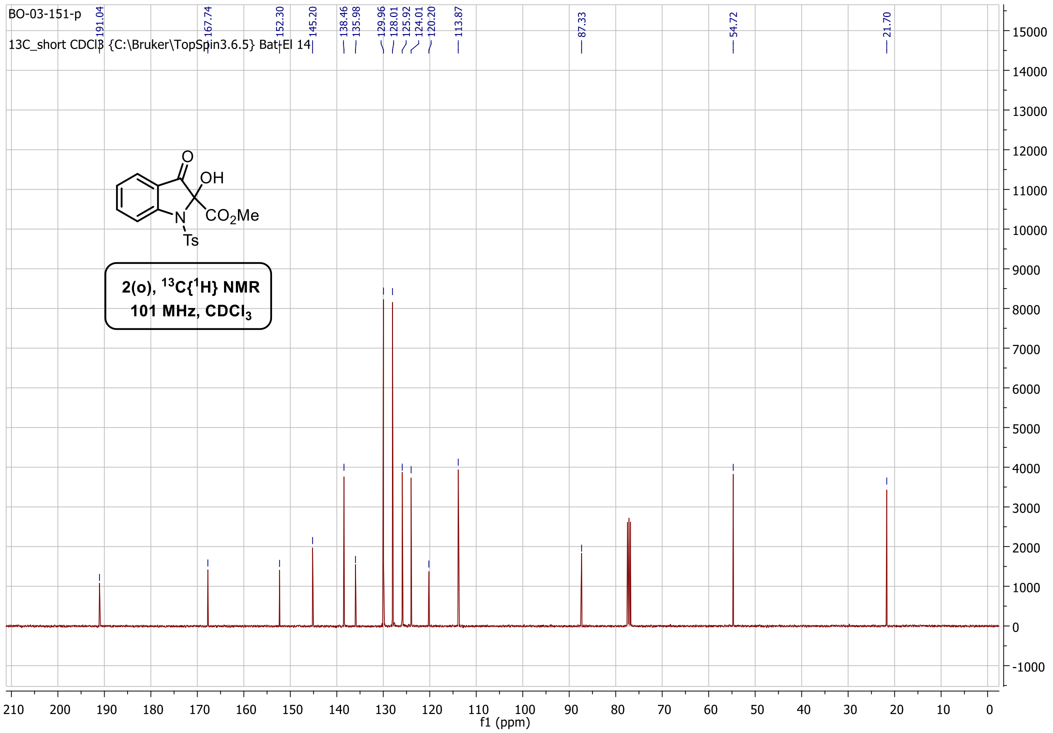

^{1}H and $^{13}C\{^{1}H\}$ NMR spectra of **2(p)**

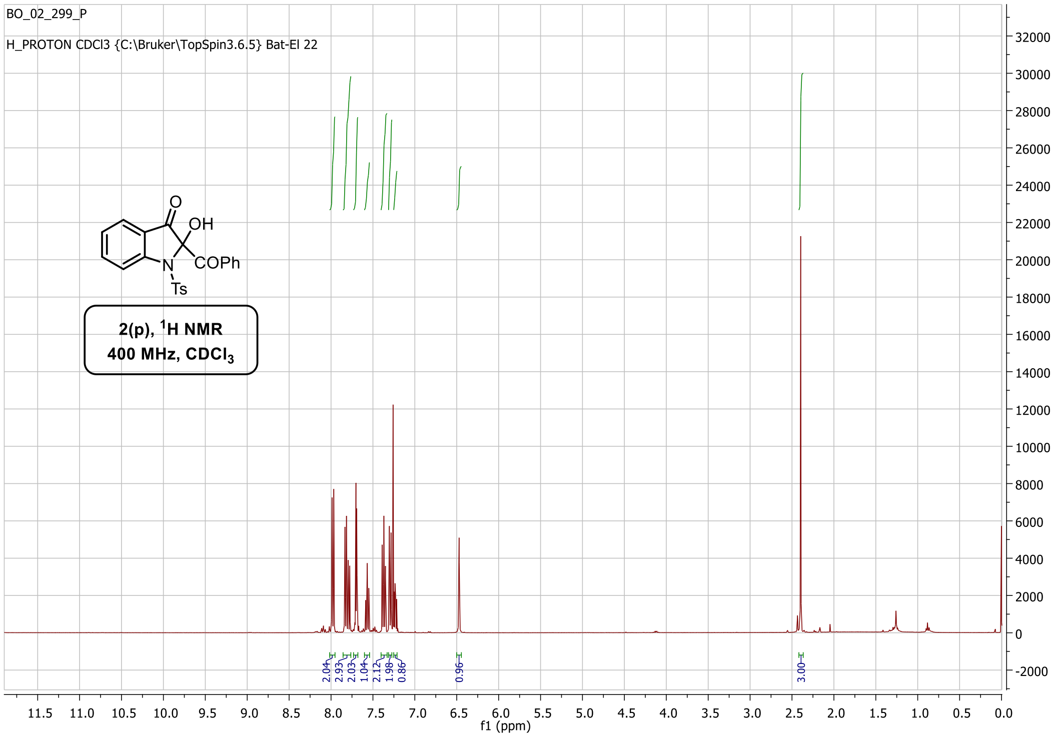

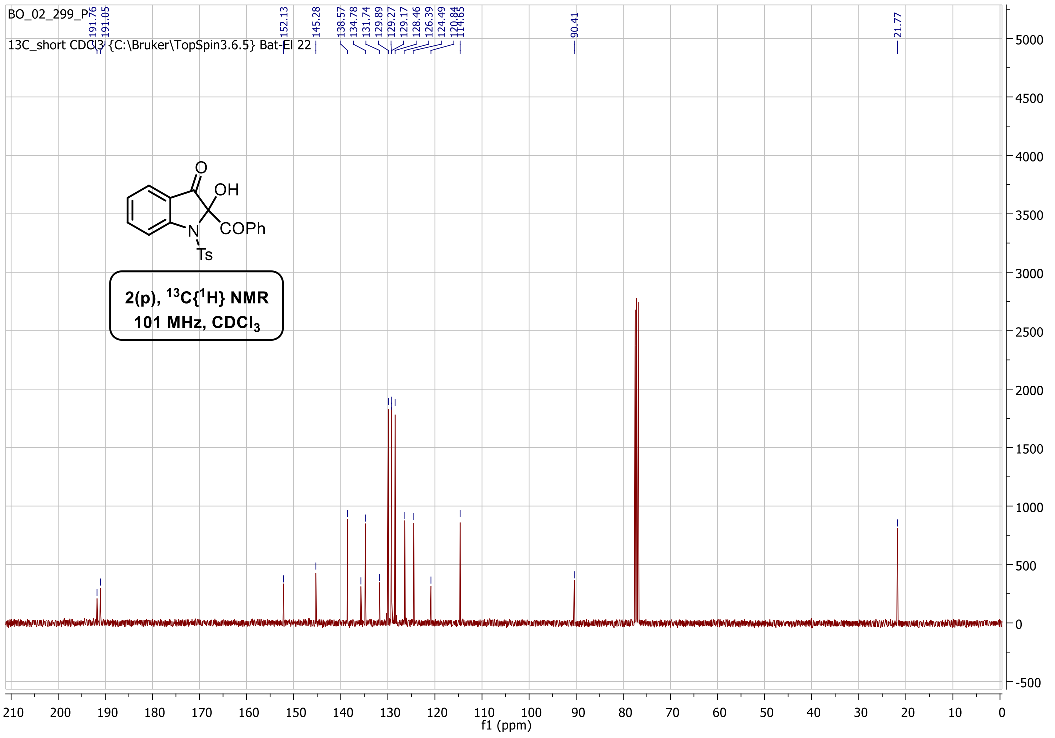

^{1}H and ^{13}C{^{1}H} NMR spectra of **2(q)**

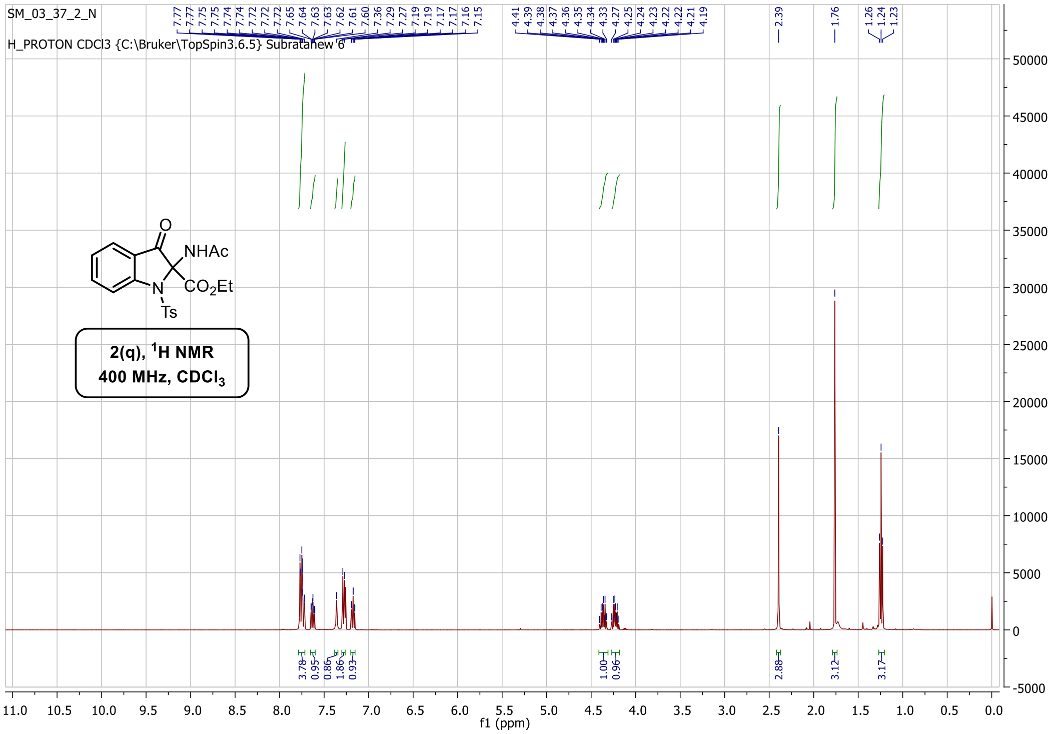

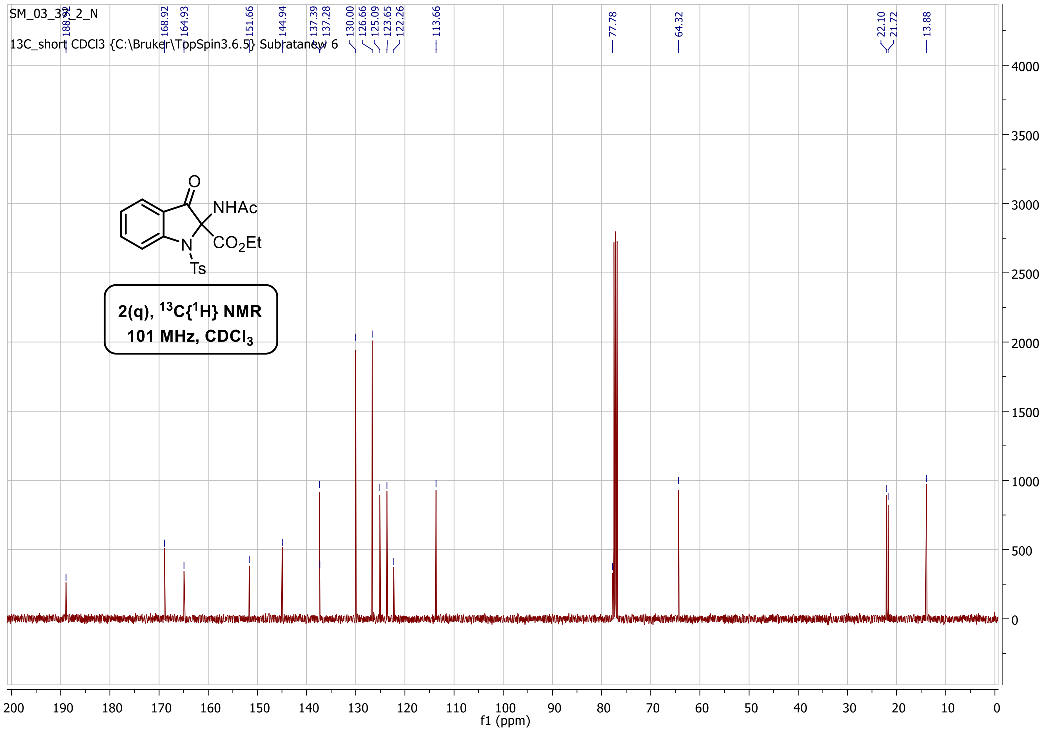

^1H and $^{13}C\{^1H\}$ NMR spectra of **2(r)**

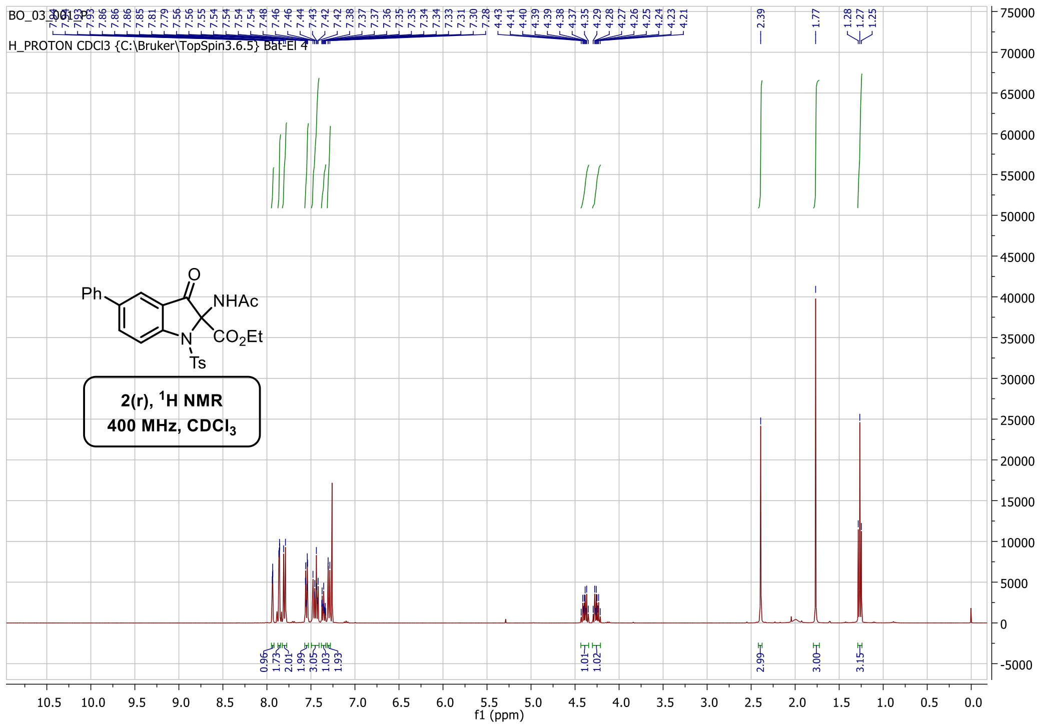

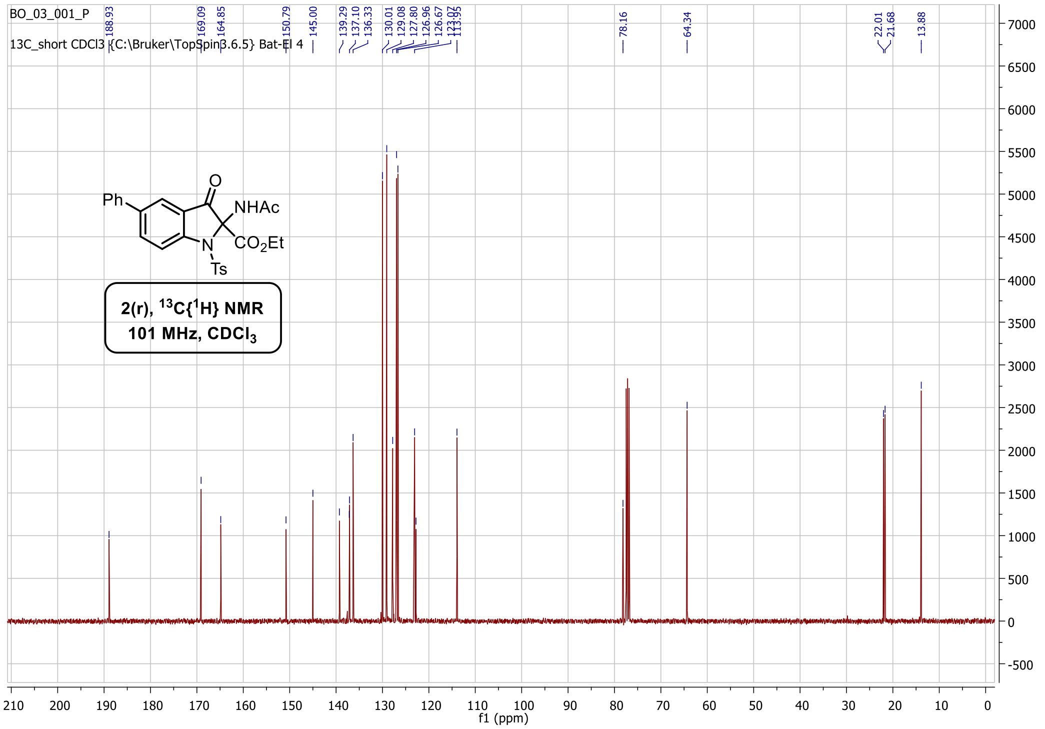

^{1}H and $^{13}C\{^{1}H\}$ NMR spectra of **2(s)**

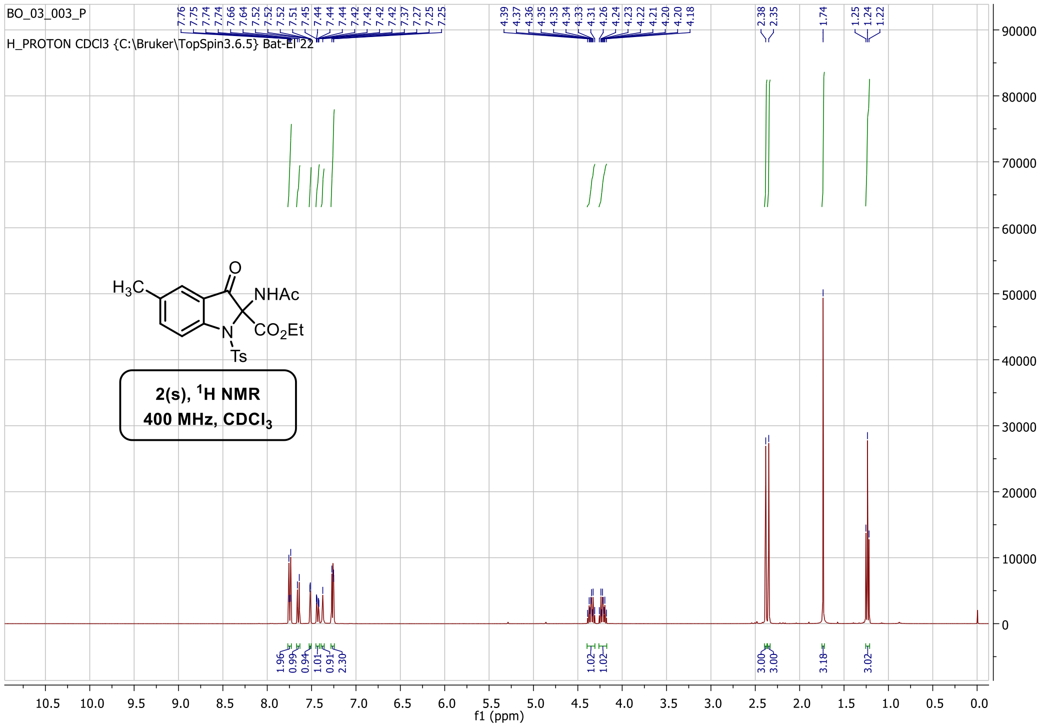

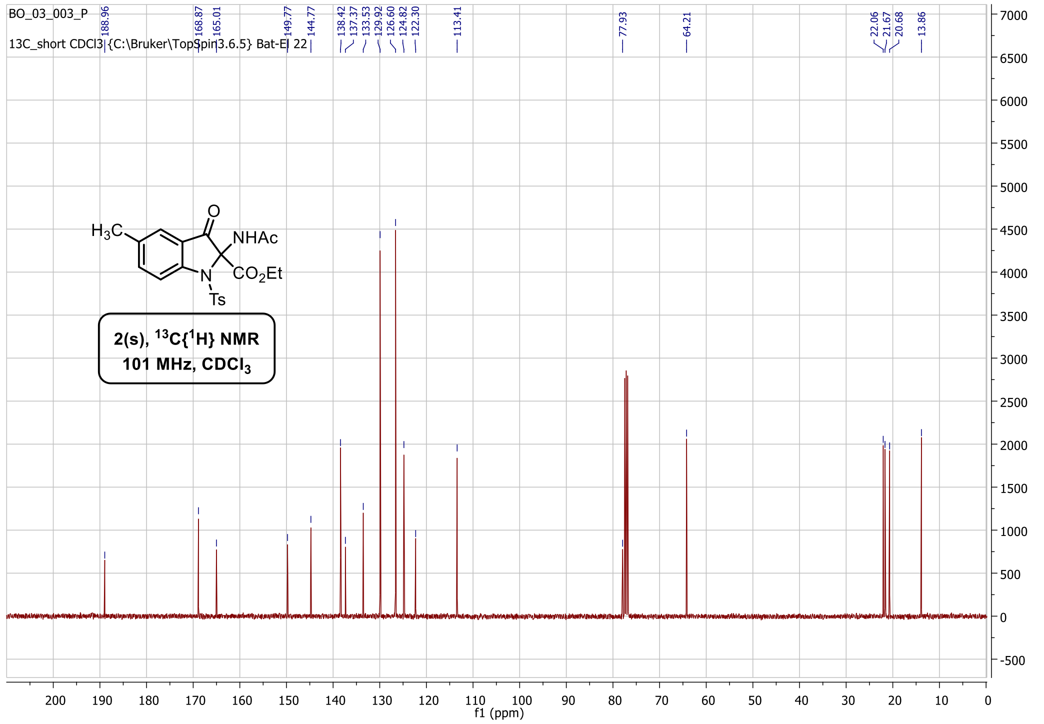

^{1}H and $^{13}C\{^{1}H\}$ NMR spectra of **2(t)**

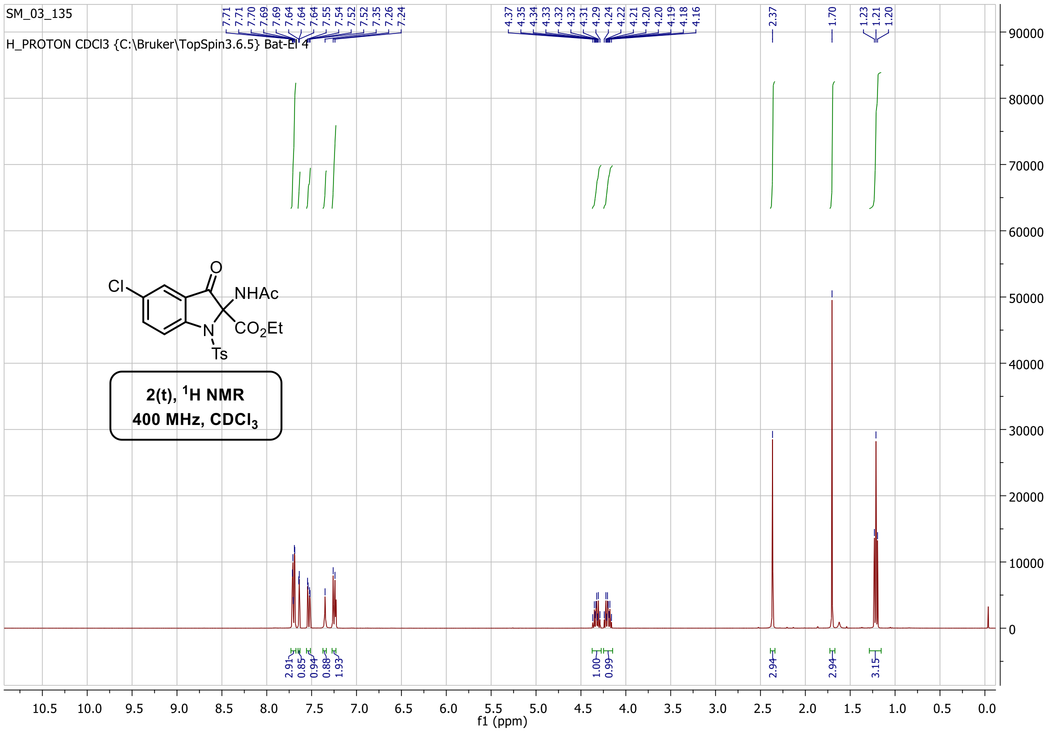

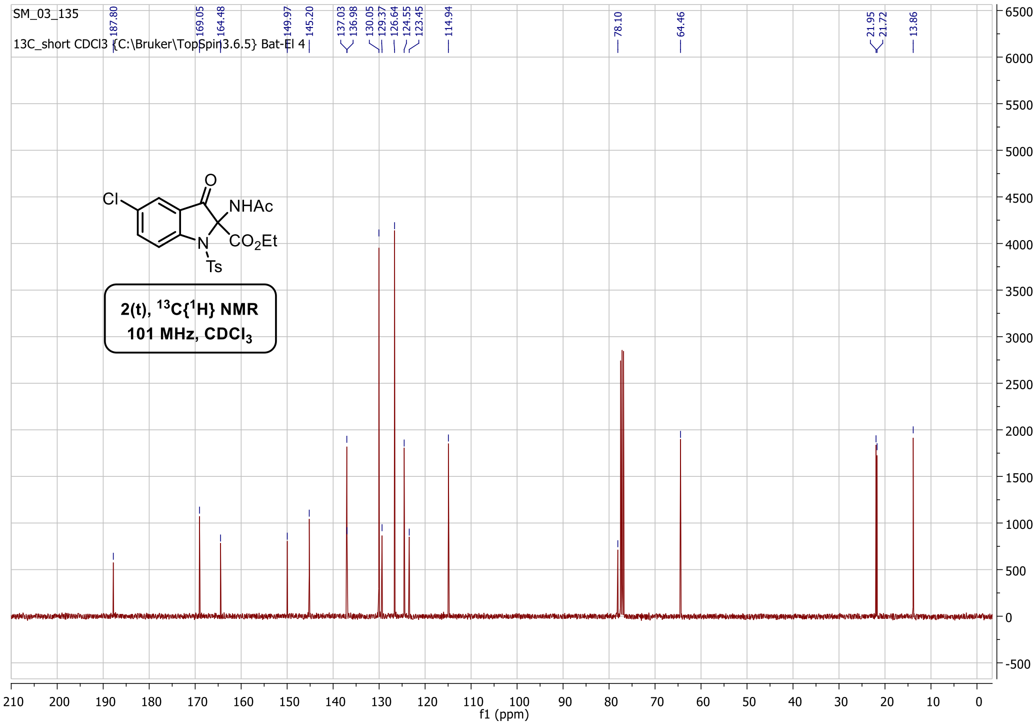

^{1}H and $^{13}C\{^{1}H\}$ NMR spectra of **2(u)**

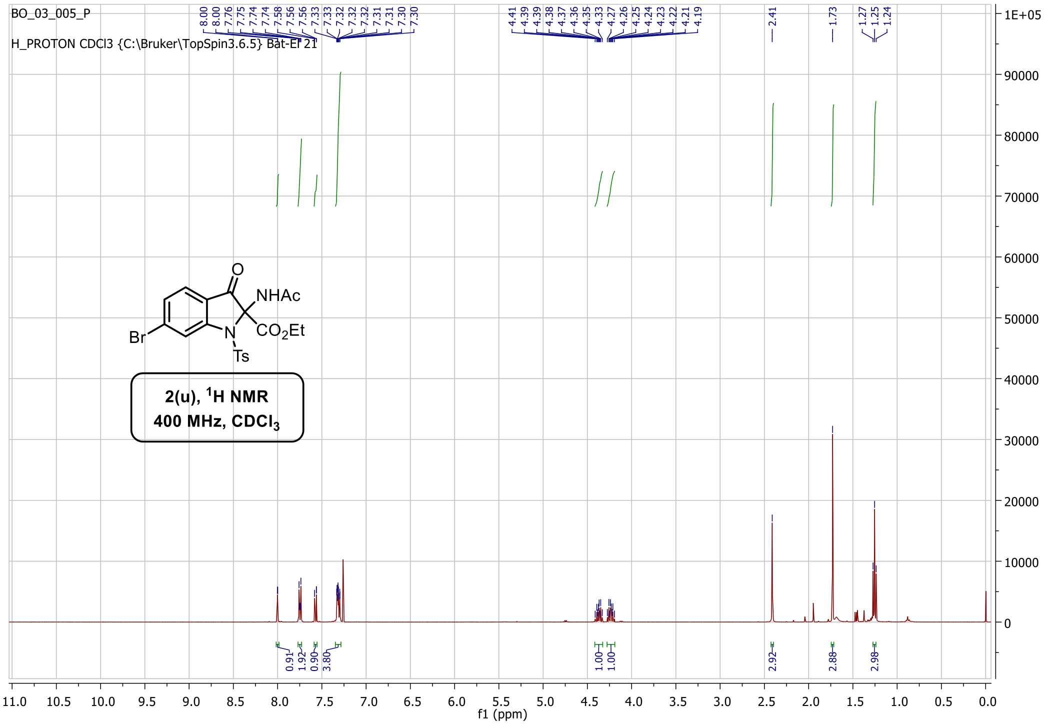

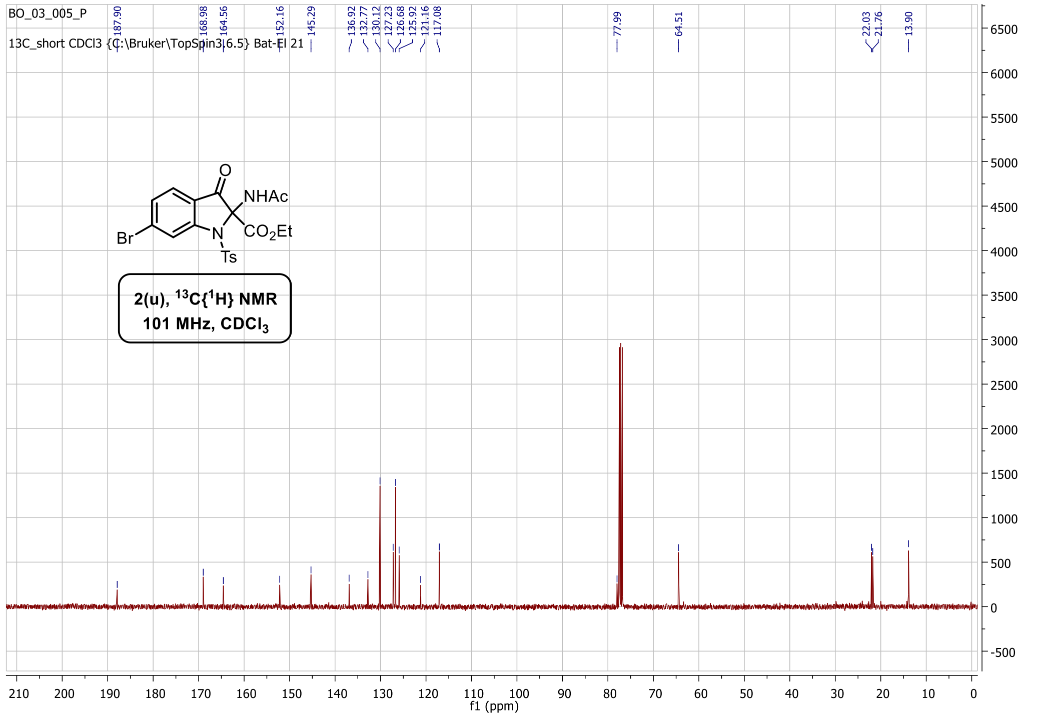

^1H and $^{13}C\{^1H\}$ NMR spectra of **2(v)**

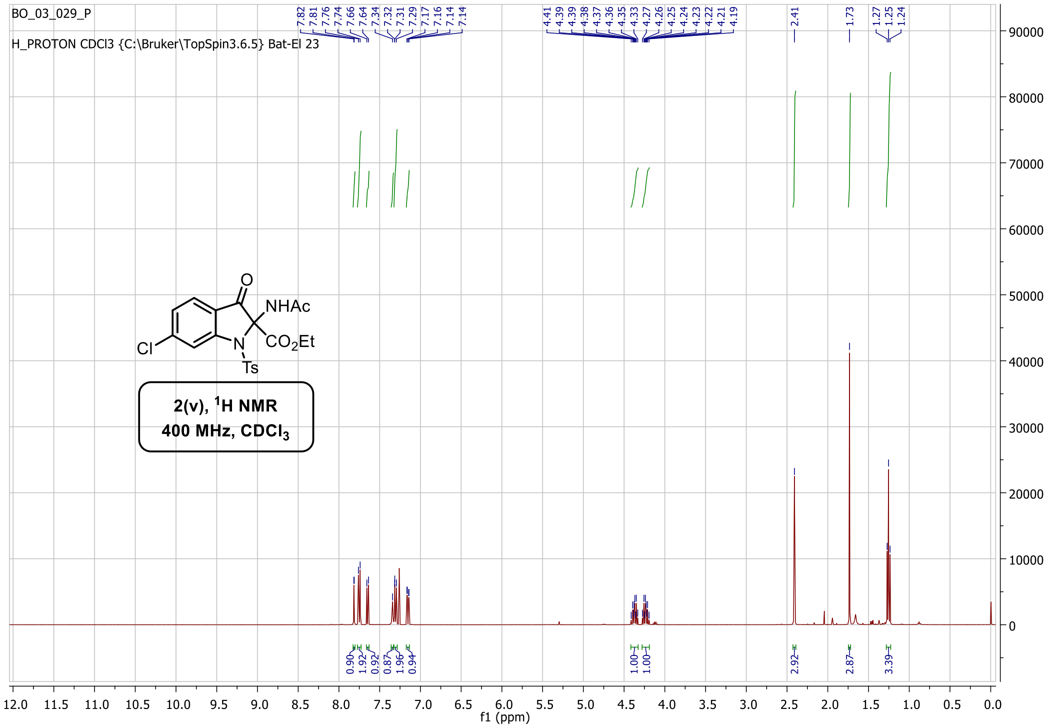

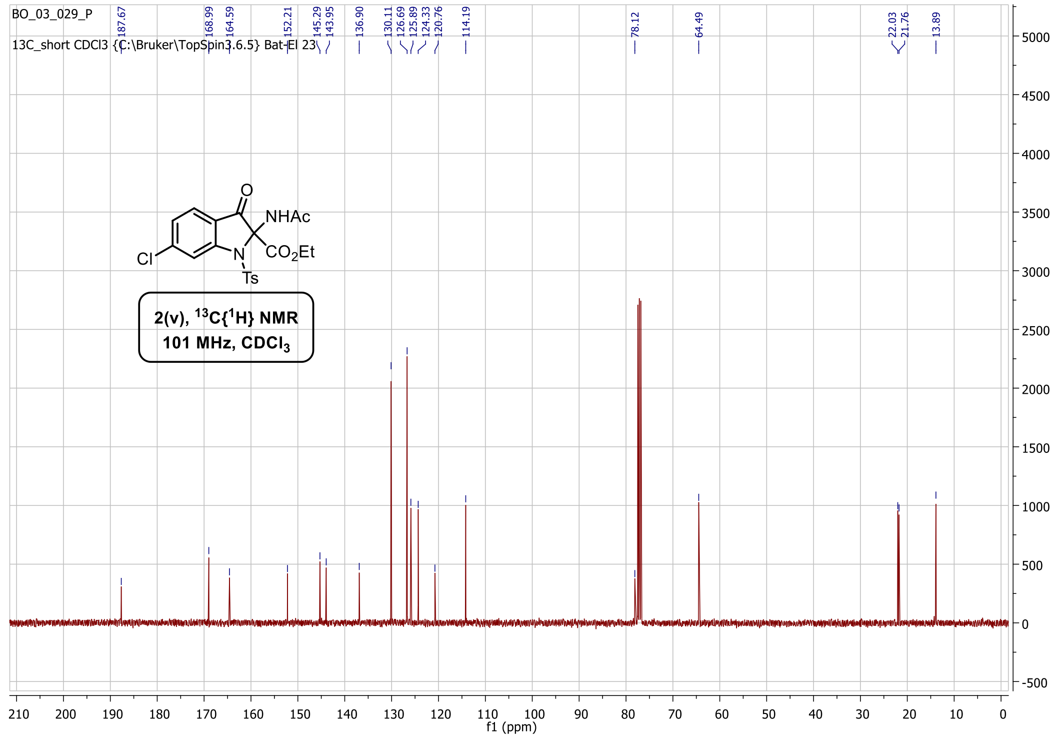

^1H, $^{13}C\{^1H\}$ and ^{19}F NMR spectra of **2(w)**

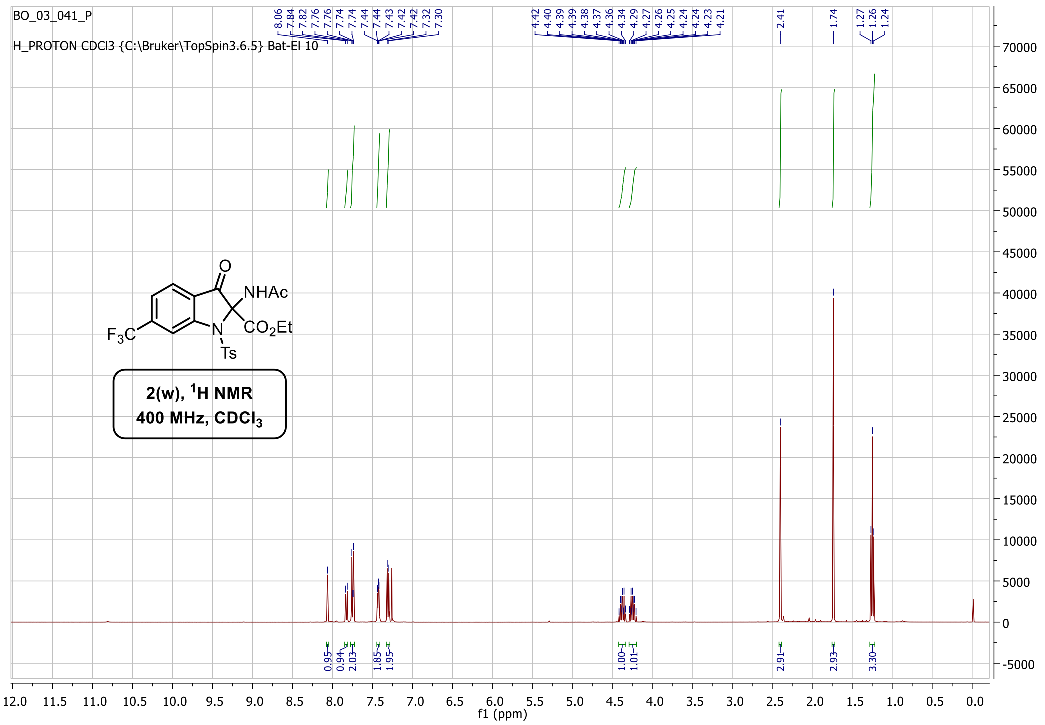

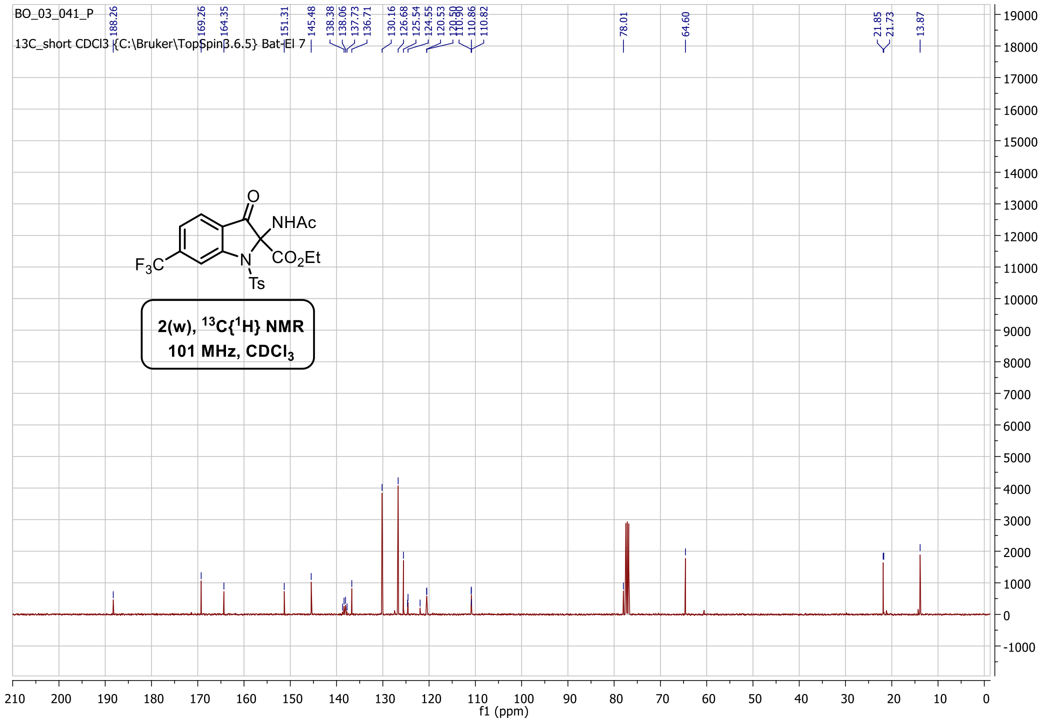

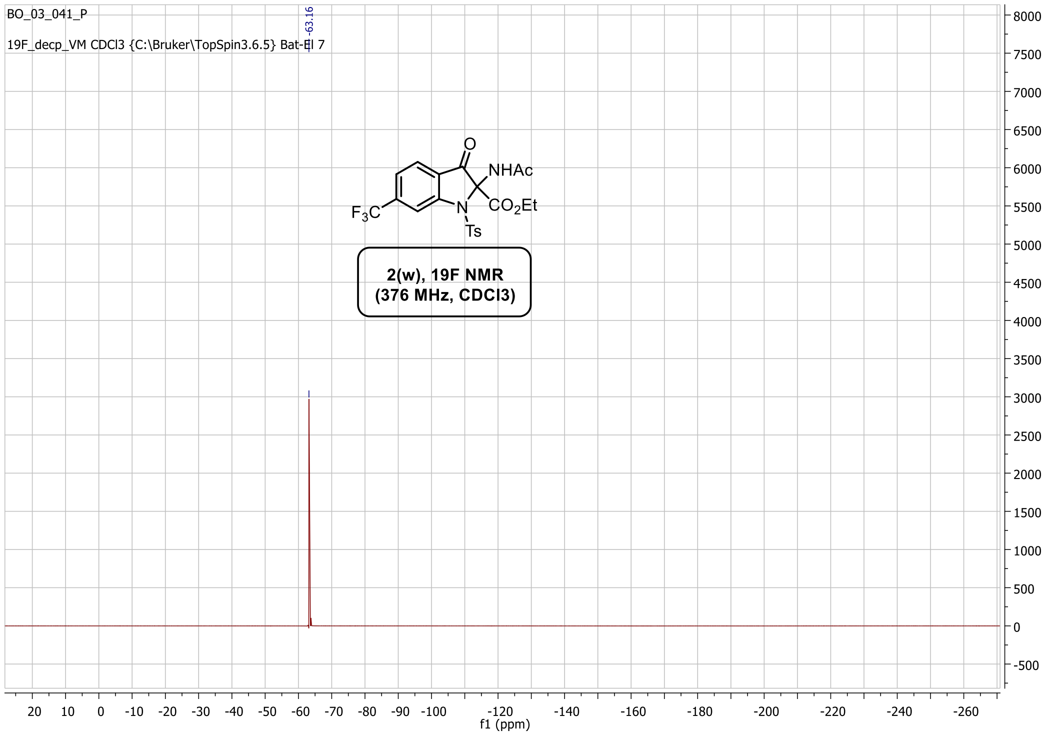
BO_03_041_P
19F_decp_VM CDCl3 {C:\Bruker\TopSpin3.6.5} Bat-El 7
-63.16
NHAc
CO_2Et
F_3C
N
Ts
O
2(w), 19F NMR
(376 MHz, CDCl3)
f1 (ppm)

^{1}H and $^{13}C\{^{1}H\}$ NMR spectra of **3**

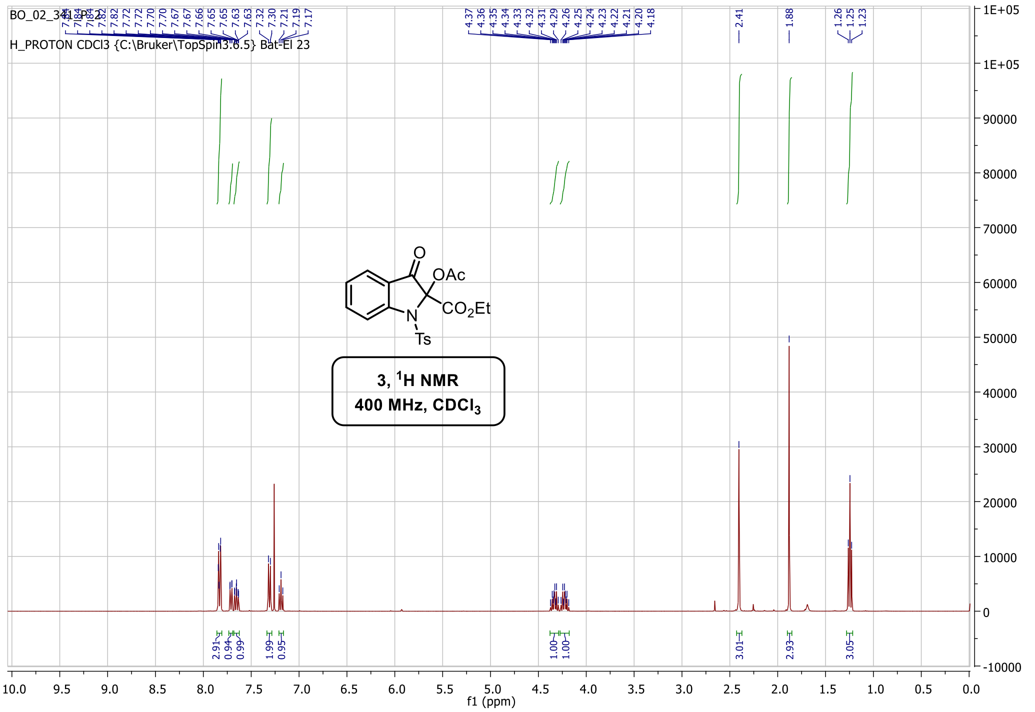

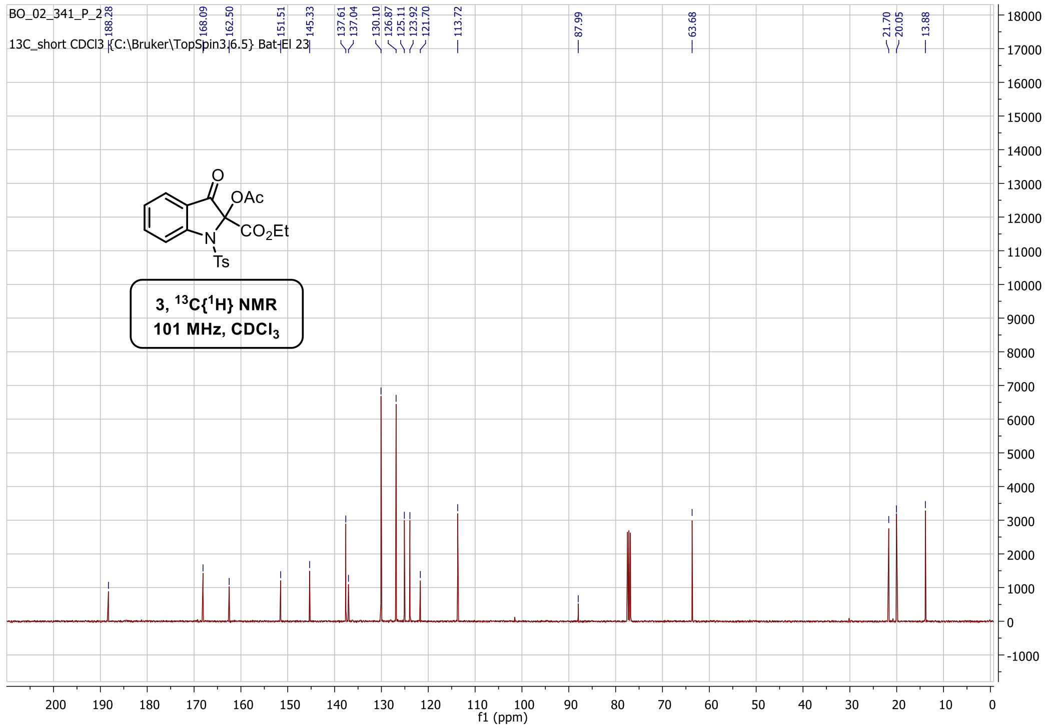

^{1}H and $^{13}C\{^{1}H\}$ NMR spectra of **4**

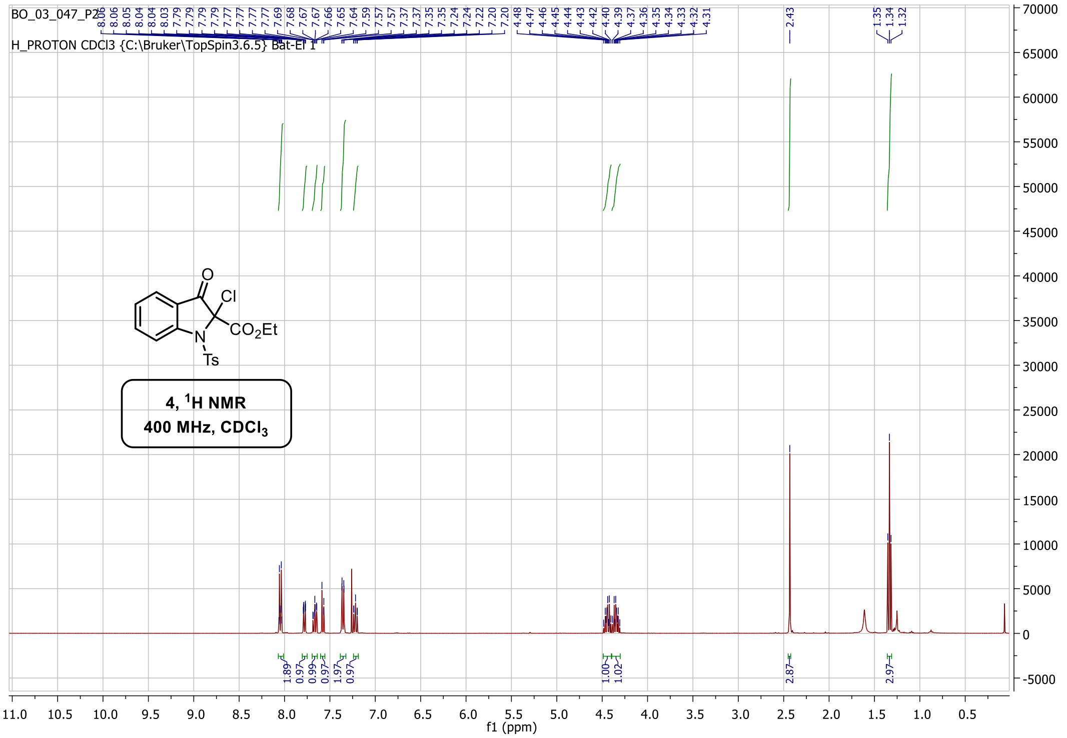

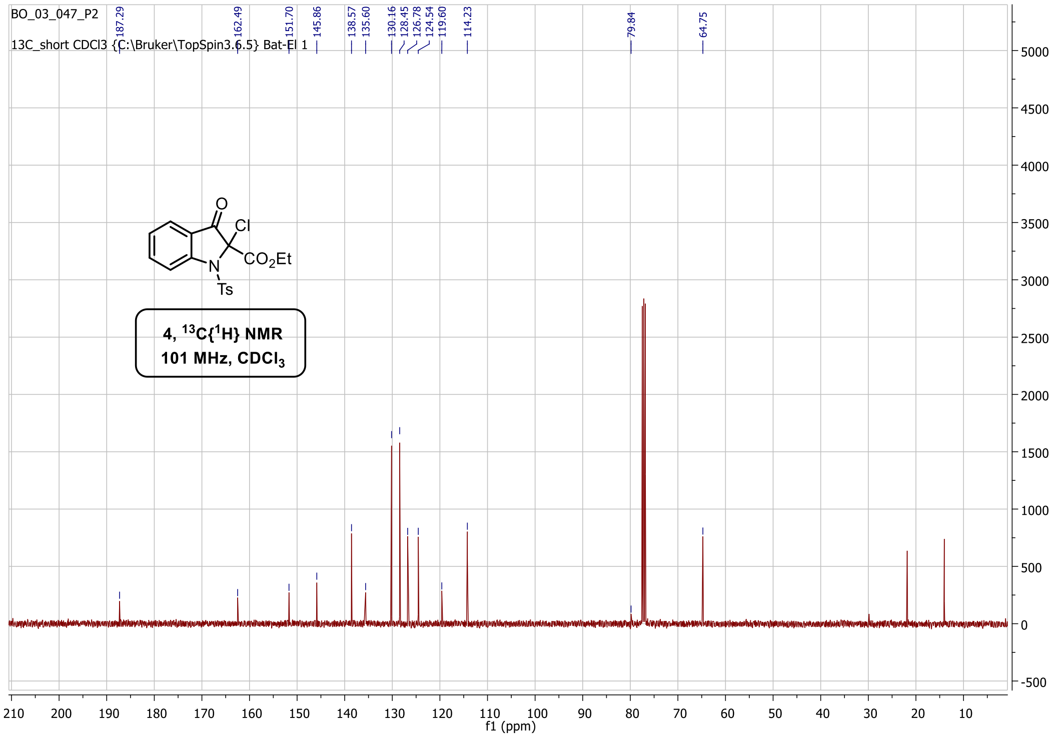

^{1}H and $^{13}C\{^{1}H\}$ NMR spectra of **5**

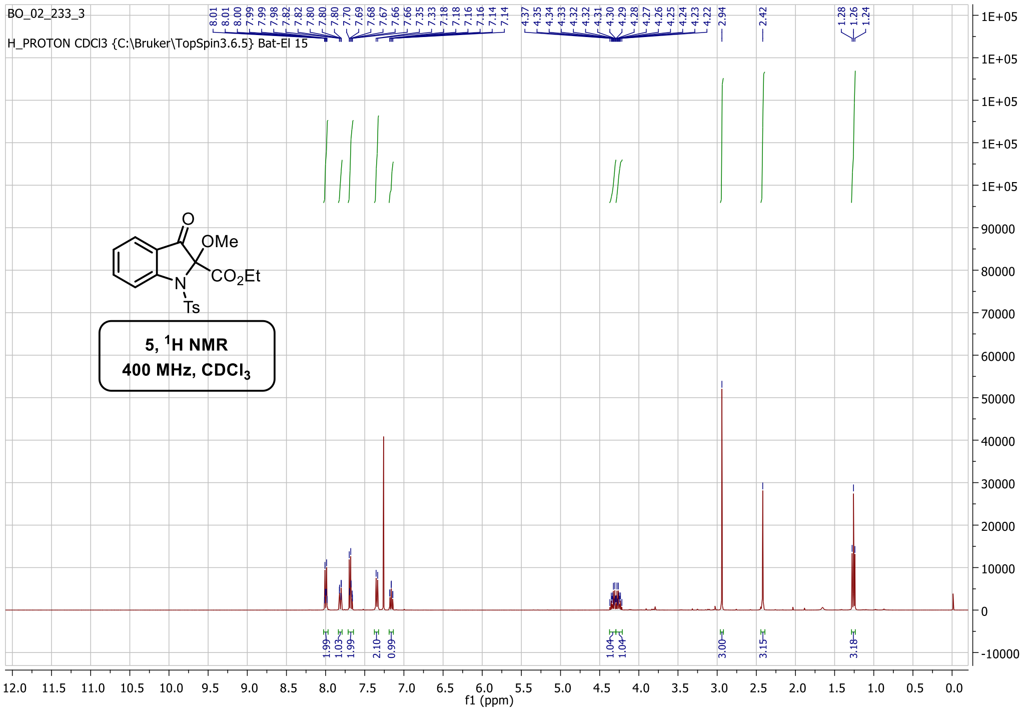

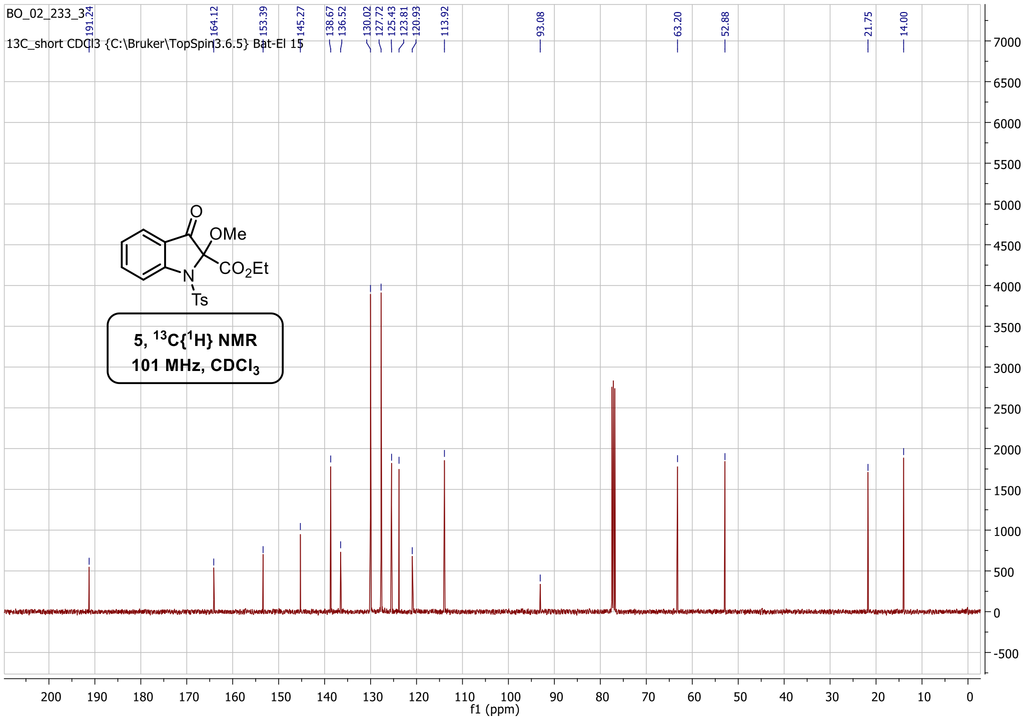

^{1}H and $^{13}C\{^{1}H\}$ NMR spectra of **6**

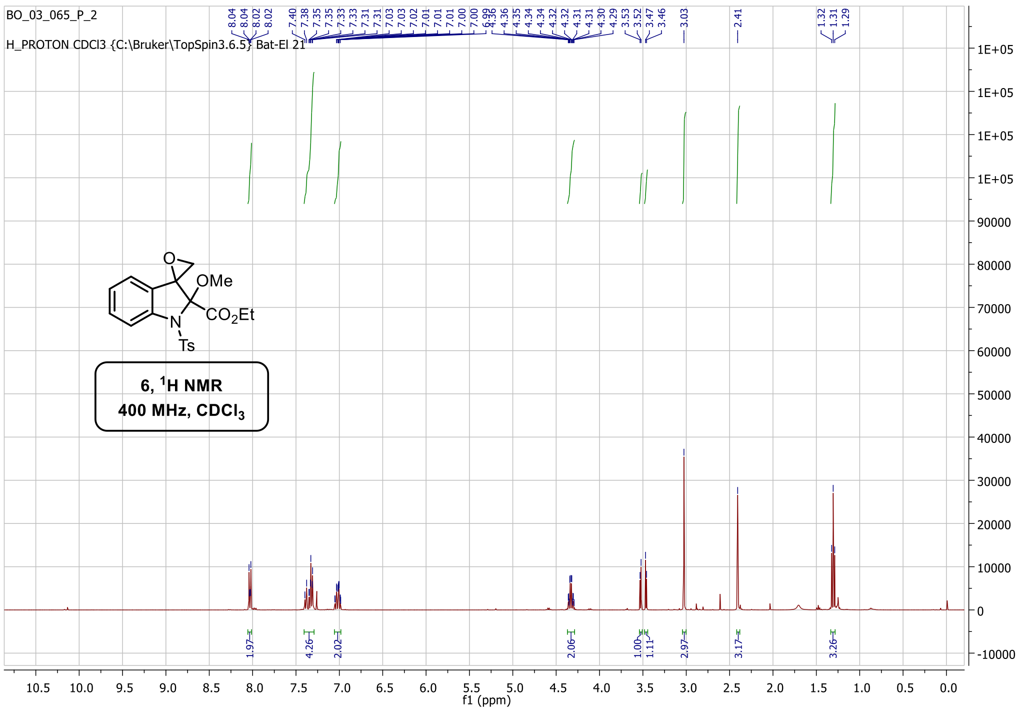

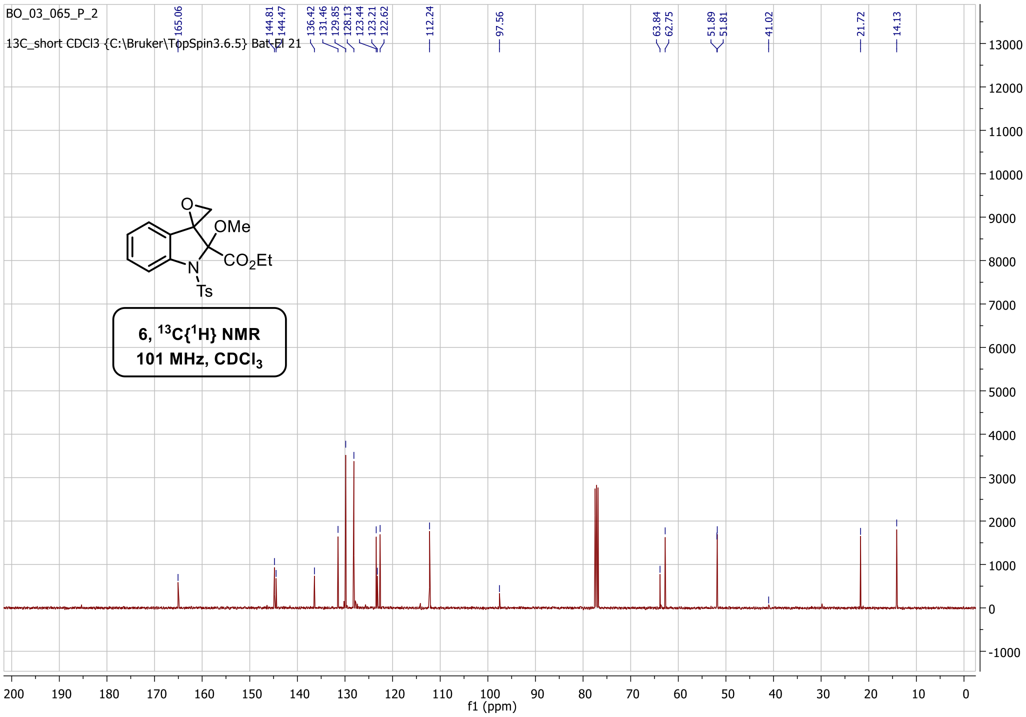

^{1}H and ^{13}C{^{1}H} NMR spectra of **Deprotected 1a**

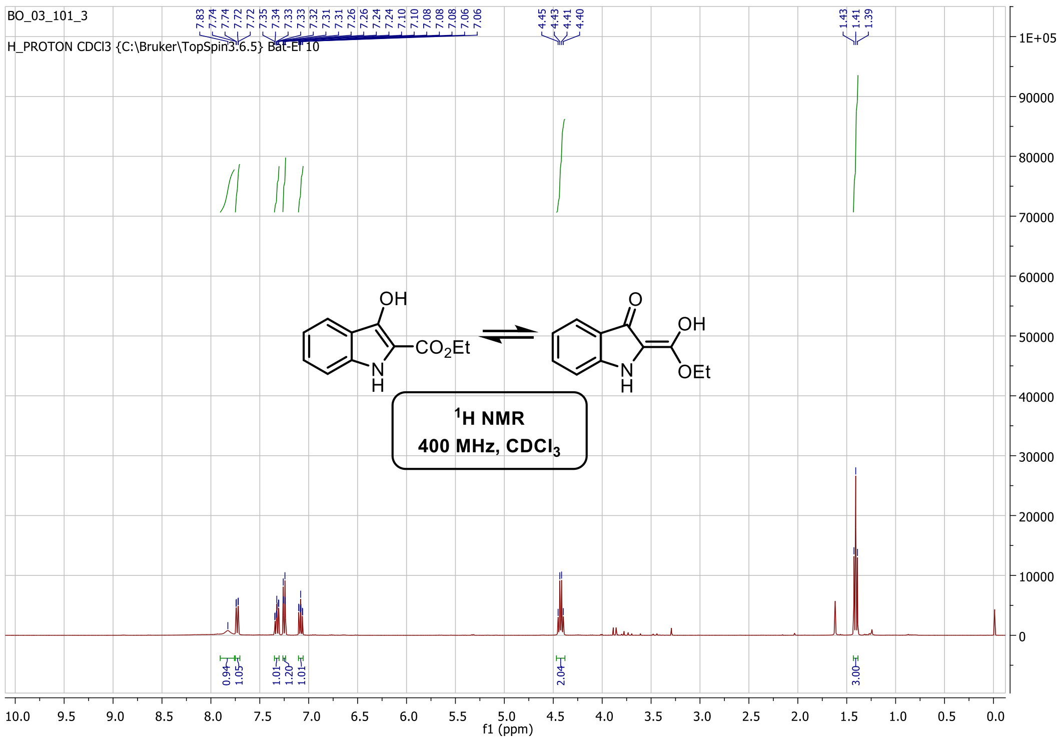

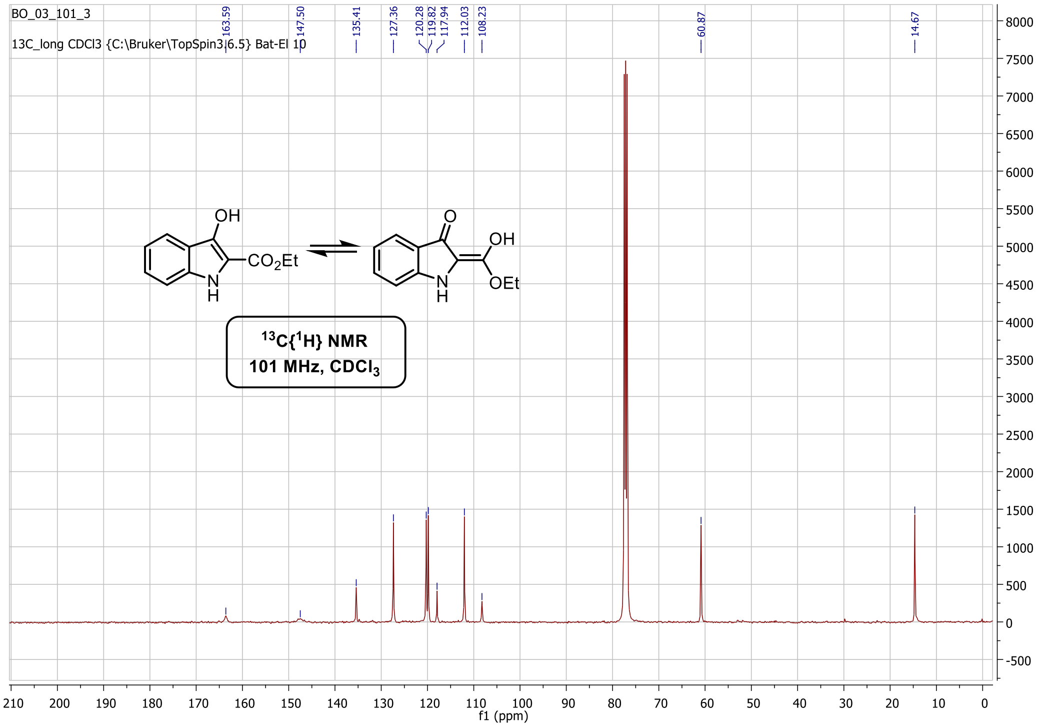

^{1}H and ^{13}C{^{1}H} NMR spectra of **7**

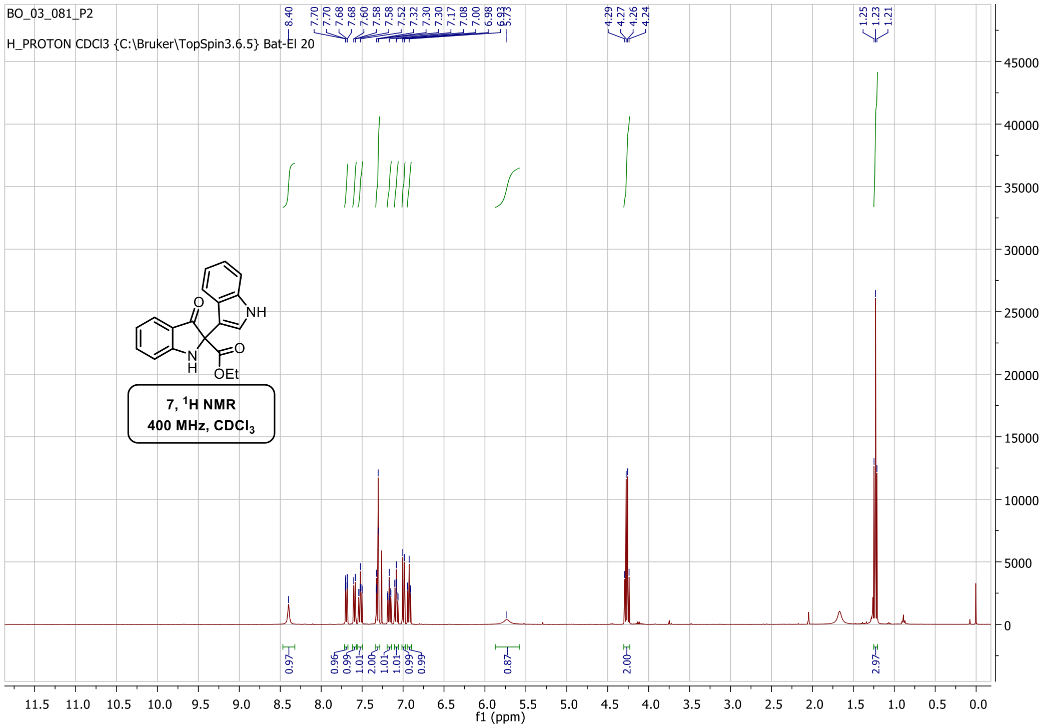

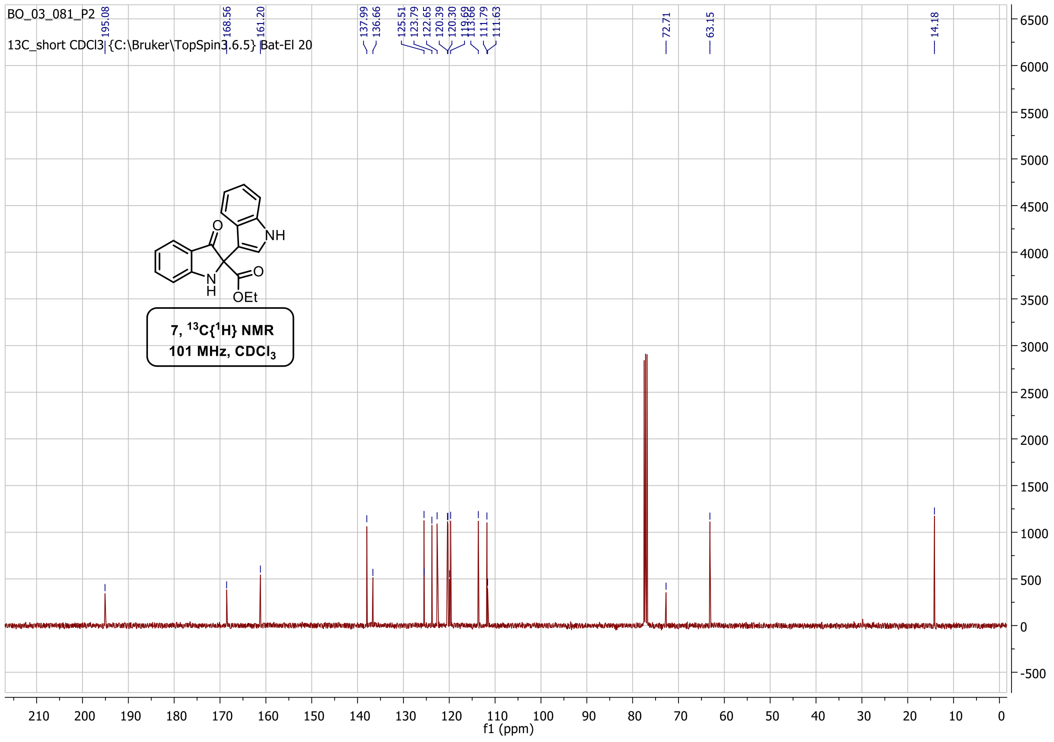

^{1}H and $^{13}C\{^{1}H\}$ NMR spectra of **8**

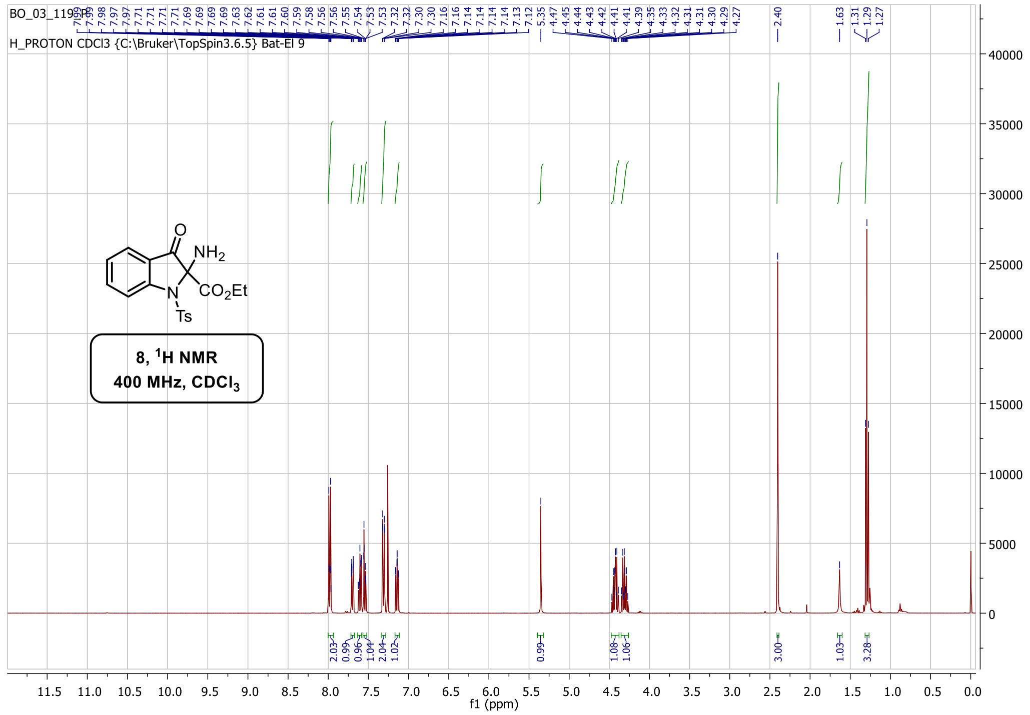

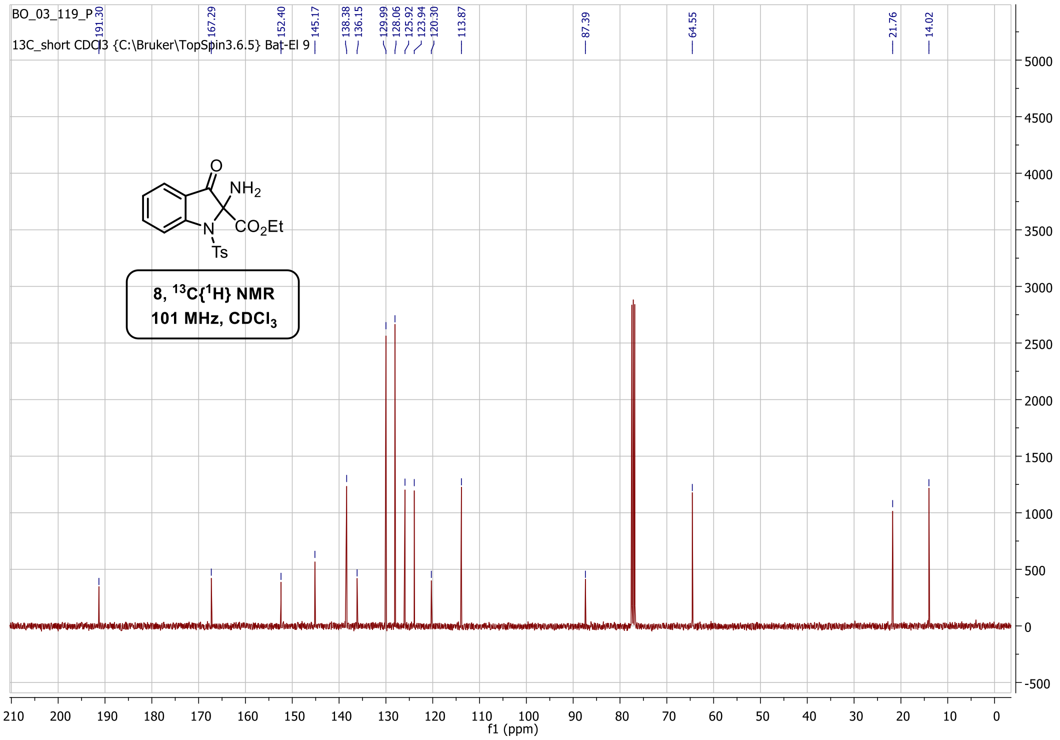

Supplement: Supplementary file 1 [file jo5c00873_si_001.pdf]
